# Synthesis of *gem*-di(boryl)cyclopropanes from non-activated olefins *via* Mn-photocatalyzed atom transfer radical addition†

**DOI:** 10.1039/d5sc02670a

**Published:** 2025-05-12

**Authors:** Jiefeng Hu, Kun Zhang, Jing Wang, Mingming Huang, Shuangru Chen, Zhuangzhi Shi, Todd B. Marder

**Affiliations:** a State Key Laboratory of Flexible Electronics (LoFE) & Institute of Advanced Materials (IAM), Nanjing University of Posts & Telecommunications Nanjing 210023 China iamjfhu@njupt.edu.cn; b School of Chemistry and Molecular Engineering, Nanjing Tech University Nanjing 211816 China; c Institut für Anorganische Chemie and Institute for Sustainable Chemistry & Catalysis with Boron, Julius-Maximilians-Universität Würzburg Am Hubland 97074 Würzburg Germany todd.marder@uni-wuerzburg.de; d State Key Laboratory of Coordination Chemistry, School of Chemistry and Chemical Engineering, Nanjing University Nanjing 210023 China

## Abstract

The application of *gem*-diboryl cyclopropanes as versatile building blocks for enhancing molecular complexity has been limited, despite the availability of a few synthetic methods. Herein, we disclose a practical and versatile manganese-catalyzed protocol that enables the synthesis of *gem*-di(boryl)cyclopropanes from non-activated alkenes in combination with (diborylmethyl)iodides. This photoinduced strategy displays good functional-group tolerance, and encompasses a wide range of applicable substrates, making it applicable to the late-stage modification of natural products. Mechanistic experiments suggest that the reaction proceeds *via* an intermolecular halogen-atom transfer radical addition, followed by deprotonative alkylation with lithium diisopropylamide, ultimately yielding cyclization products. The versatility and practicality of this approach are further highlighted by the successful implementation of several transformations, which provide an expedited route for synthesizing highly functionalized molecules.

## Abstract 2

We report the Mn_2_(CO)_10_-catalyzed halogen-atom transfer radical addition/nucleophilic cyclization of alkenes using a (diborylmethyl)iodide reagent under visible light irradiation, enabling the selective synthesis of *gem*-di(boryl)cyclopropanes.

## Graphical abstract

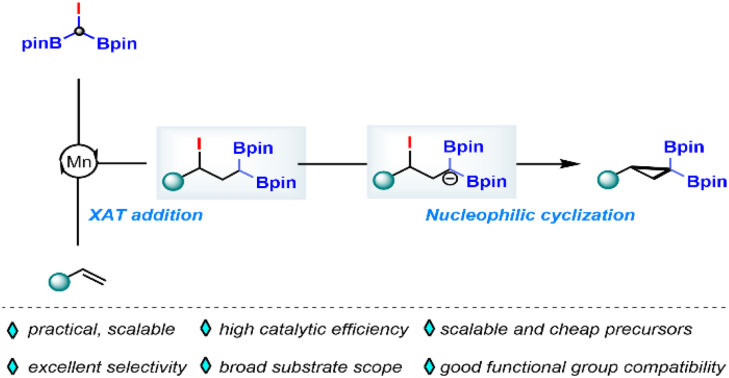

## Introduction

**Fig. 1:**
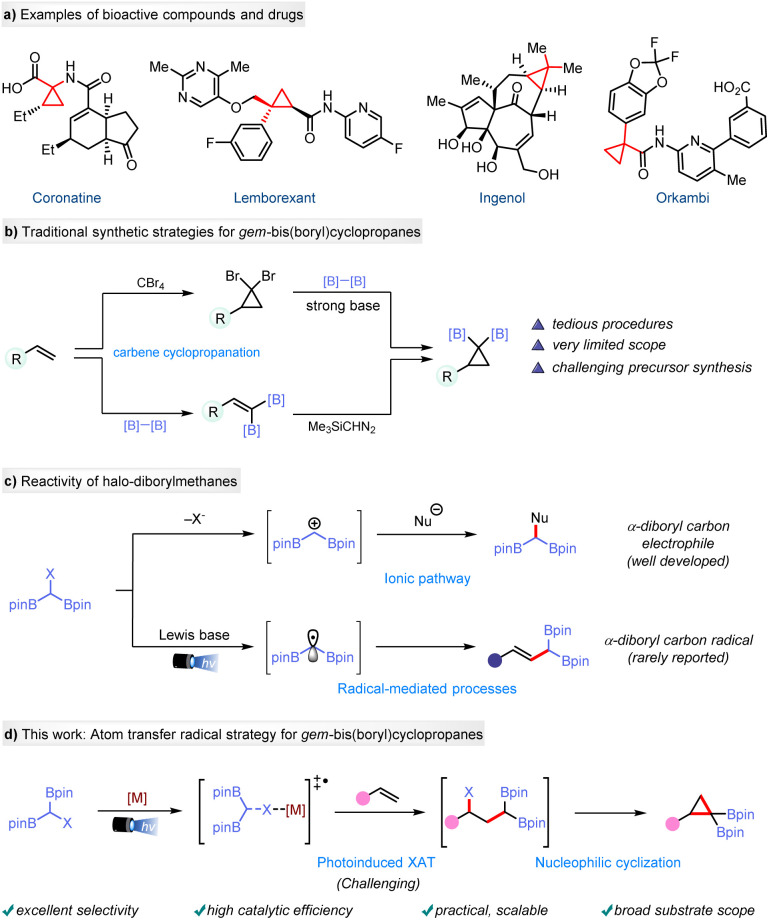
*De novo* synthesis of *gem*-bis(boryl)cyclopropanes: background, challenges, and our approach.

Cyclopropanes, particularly those with molecular skeletons containing quaternary or tetrasubstituted carbon centers, represent a valuable class of molecules characterized by high ring strain, widely found in biologically active molecules and pharmaceuticals, such as coronatine, lemborexant, ingenol, and orkambi (Fig. 1a).^1^ Over the years, numerous approaches to access these small carbocycles have been developed,^2^ as represented by [2 + 1] cyclization of olefins, including the Simmons–Smith reaction,^3^ carbenoid migration insertion,^4^ ylide-type cyclopropanation,^5^ and photoinduced radical cyclopropanation.^6–8^ While these strategies have enabled the formation of cyclopropanes, methodology for the synthesis of *gem*-di(boryl)cyclopropanes—compounds^9^ with significant potential for multi-step functionalization and cross-coupling to create highly functionalized cyclopropyl derivatives—remains scarce and is generally limited in terms of diversification. Traditionally, their synthesis has relied on borylative cross-coupling of 1,1-dibromocyclopropanes with bis(pinacolato)diboron (B_2_pin_2_)^10^ or the use of diazo compounds for palladium-catalyzed cyclopropanation of 1,1-diborylalkenes (Fig. 1b).^11^ However, these methods suffer from some drawbacks, such as harsh conditions, cumbersome procedures, challenging precursor synthesis, and limited substrate scope. A recent breakthrough by Liu and co-workers has provided a novel approach using halogenated *gem*-diborylmethane as a boron ylide precursor, enabling the cyclopropanation of electron-deficient olefins to deliver *gem*-diborylcyclopropanes.^12^ Despite this progress, there remains a clear need for the development of efficient and versatile methods for accessing *gem*-di(boryl)cyclopropanes from non-activated olefins.

1,1-Diborylalkanes are versatile building blocks extensively used in medicine, materials science, and synthetic chemistry.^13^ They operate through two primary reaction modes: generating α-borylalkylmetal species or α-boryl carbanions for monodeborylative cross-coupling,^14^ and deprotonation to form *gem*-diboryl carbanions that readily engage in cross-coupling reactions with electrophiles.^15^ Recent studies have increasingly focused on synthesizing α-halogenated diboron compounds and exploring their coupling reactions with nucleophiles to expand the reaction modes of *gem*-diborylalkanes (Fig. 1c).^16^ However, their potential for photoinduced radical reactivity has been rarely explored.^17^ In 2023, our group reported the photoinduced borylcyclopropanation of alkenes using a (diborylmethyl)iodide to synthesize cyclopropyl boronic esters^18^*via* an radical polar crossover (RPC) mechanism involving an α-iodoboryl carbon-centered radical.^19^ Recently, Molloy and co-workers utilized a Lewis base to develop a photoinduced method for activating ambiphilic reagents, leading to the generation of α-bimetalloid radicals which can engage with various SOMOphiles to give the functionalized organoboronates.^20^ Inspired by these reports, we envisioned that the proper choice of a catalyst could facilitate halogen-atom transfer (XAT)^21^ from (diborylmethyl)iodide to achieve iododiborylcarbo functionalization of alkenes, thereby opening a unique pathway for constructing geminal di(boronates) with distinctive structural features. Interestingly, Yin, Wang, and co-workers reported the addition of the C–I bond of RCHI(Bpin) across the CC triple bond of terminal alkynes; using 4CzIPN as the photocatalyst gave predominantly the *E*-isomer whereas using Mn_2_(CO)_10_ as the photocatalyst gave predominantly the *Z*-isomer.^21*e*^ Herein, we present a photoinduced method for the modular and efficient synthesis of *gem*-di(boryl)cyclopropanes through manganese-catalyzed XAT radical addition of (diboronmethyl)iodide to alkenes, followed by nucleophilic cyclization (Fig. 1d). Additionally, the intermediate adducts obtained during this process can serve as valuable precursors for various transformations, such as Heck-type cross-coupling and radical borylation.

## Results and discussion

In our initial investigation, we focused on a model reaction between 4-phenyl-1-butene (1a) and 2,2′-(iodomethylene)bis(4,4,5,5-tetramethyl-1,3,2-dioxaborolane) (2a). We first screened various potential photocatalysts in dry *n-*hexane. Commonly used photocatalysts, such as *fac*-[Ir(ppy)_3_], [Ru(bpy)_3_]Cl_2_, eosin Y, and [Mes-Acr]^+^[BF_4_]^−^, showed no catalytic activity, and the starting materials were recovered (Table 1, entries 1–4). With 4CzIPN as a photocatalyst, the reaction mixture was irradiated with a 25 W blue light-emitting diode (LED, *λ*_max_ = 440 nm) at room temperature for 3 h, followed by the treatment with lithium diisopropylamide (LDA, 2.5 M, in THF) at −20 °C, and a trace amount of 1,1-bis(boryl)cyclopropane (3a) was detected by gas chromatography-mass spectrometry (GC-MS). Upon replacing 4CzIPN with Mn_2_(CO)_10_,^22^ the GC yield of 3a significantly increased, and we successfully isolated the cyclopropane product with an 81% yield (Table 1, entry 6). However, using Mn(CO)_5_Br as the photocatalyst led to reduced reactivity (Table 1, entry 7), and MnBr_2_ failed to produce the desired *gem*-bis(boryl)cyclopropanes (Table 1, entry 8). Reducing the catalyst loading lowered the yield somewhat (Table 1, entry 9). We then explored various bases, including LiO^*t*^Bu, lithium tetramethylpiperidide (LTMP), and ^*n*^BuLi (entries 10–12), and found that LTMP was the most effective. The impact of different solvents was also assessed: acetonitrile (MeCN), *N*,*N*-dimethylformamide (DMF), and tetrahydrofuran (THF) proved ineffective for the photoinduced cyclopropanation (Table 1, entries 13–15), while dichloromethane (DCM) lowered the yield of 3a (Table 1, entry 16). Finally, control experiments validated the indispensability of the photocatalyst and visible light exposure for this transformation (Table 1, entries 17 and 18). Exploration of other reaction conditions is given in the ESI.†

**Table 1:** Effect of reaction parameters^a^

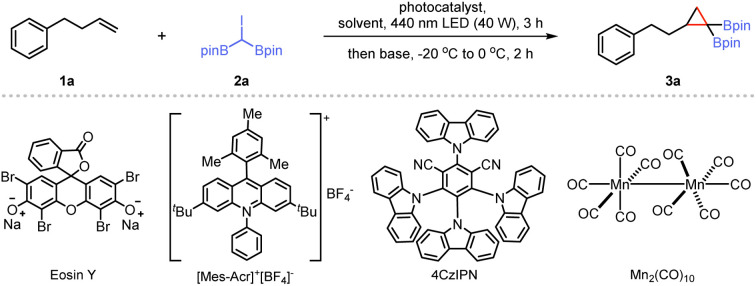				
Entry	Photocatalyst (mol%)	Base	Solvent	Yield^b^ (%)
1	*fac*-Ir(ppy)_3_ (2)	LDA	^*n*^Hexane	0
2	[Ru(bpy)_3_]Cl_2_ (2)	LDA	^*n*^Hexane	0
3	Eosin Y (10)	LDA	^*n*^Hexane	0
4	[Mes-Acr]^+^[BF_4_]^−^ (10)	LDA	^*n*^Hexane	0
5	4CzIPN (10)	LDA	^*n*^Hexane	<10
6	Mn_2_(CO)_10_ (10)	LDA	^*n*^Hexane	90 (81)^c^
7	Mn(CO)_5_Br (10)	LDA	^*n*^Hexane	27
8	MnBr_2_ (10)	LDA	^*n*^Hexane	0
9	Mn_2_(CO)_10_ (5)	LDA	^*n*^Hexane	71
10	Mn_2_(CO)_10_ (10)	LiO^*t*^Bu	^*n*^Hexane	0
11	Mn_2_(CO)_10_ (10)	LTMP	^*n*^Hexane	87
12	Mn_2_(CO)_10_ (10)	^*n*^BuLi	^*n*^Hexane	22
13	Mn_2_(CO)_10_ (10)	LDA	MeCN	0
14	Mn_2_(CO)_10_ (10)	LDA	DMF	0
15	Mn_2_(CO)_10_ (10)	LDA	THF	<10
16	Mn_2_(CO)_10_ (10)	LDA	DCM	47
17	—	LDA	^*n*^Hexane	0
18^d^	Mn_2_(CO)_10_ (10)	LDA	^*n*^Hexane	0

a Unless otherwise noted, the reaction conditions are as follows: 4-phenyl-1-butene 1a (0.3 mmol, 1 equiv.), (diborylmethyl)iodide 2a (0.36 mmol, 1.2 equiv.), photocatalyst (10 mol%), solvent (1 mL), 3 h, 440 nm blue LED (40 W), 25–40 °C, under argon. The reaction mixture was cooled to −20 °C, followed by the addition of base and stirring for 2 h at 0 °C.

b The yields of 3a were determined from the crude reaction mixtures by GC-MS analysis *vs.* a calibrated internal standard and are averages of two runs.

c Isolated yield after chromatography.

d Reaction carried out in the dark. LDA = lithium diisopropylamide; LTMP = lithium tetramethylpiperidide.

**Scheme 1:**
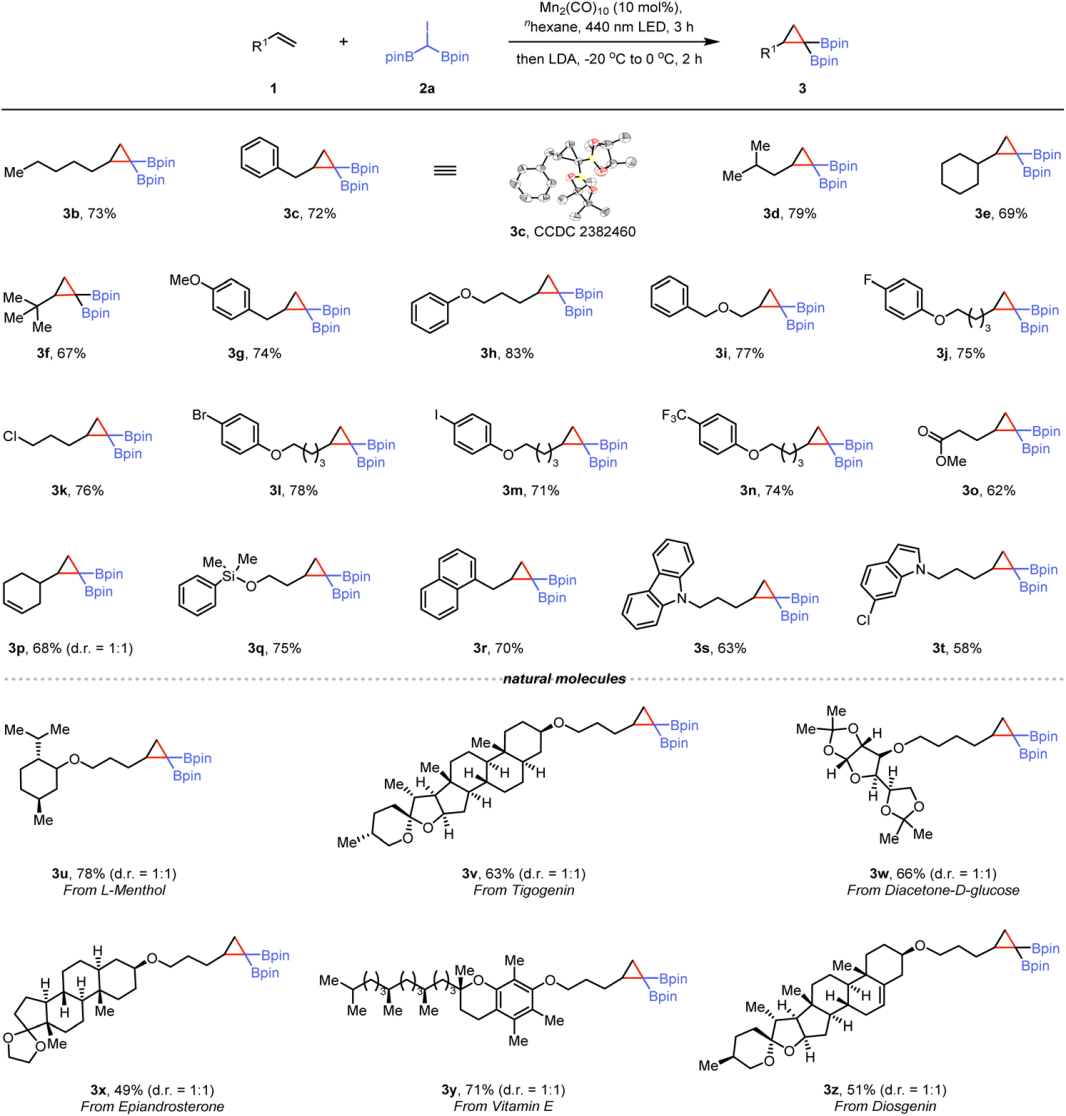
Photoinduced synthesis of *gem*-bis(boryl)cyclopropanes.

With the optimized conditions identified, we examined the scope and limitations of this one-pot synthesis method. As shown in Scheme 1, terminal alkenes, such as 1-heptene, allylbenzene, and 4-methylpent-1-ene, afforded the corresponding *gem*-disubstituted cyclopropanes efficiently (3b–3d). The structure of compound 3c, was confirmed by single-crystal X-ray diffraction.^23^ For highly sterically hindered olefins, this cyclization reaction proceeds smoothly under standard reaction conditions (3e and 3f). Moreover, the mild conditions accommodated a wide range of functional groups, including ethers (3g–3i), halide (3j–3m), trifluoromethyl (3n), and ester (3o). A substrate containing an internal olefin moiety under our conditions afforded the desired product 3p in 68% yield, resulting from reaction at the terminal olefin moiety, with the internal C=C bond remaining untouched. An alkene containing a siloxy group gave the corresponding *gem*-di(boryl)cyclopropane (3q) in good yield. 1-Allylnaphthalene was a suitable substrate for this reaction yielding 3r. Furthermore, substrates featuring heterocyclic cores, such as carbazole and indole, yielded the corresponding products (3s and 3t) in 63% and 78% yields, respectively. However, using acyclic internal alkenes or methyl acrylate as substrates under the standard conditions, no products or adducts were detected by GC-MS (see ESI† for details). After evaluating the scope of this photochemical method, we next aimed to demonstrate its application by incorporating di(boryl)cyclopropanes into natural products and biologically relevant molecules. An *L*-menthol derivative reacted with 2a using the light-induced Mn-catalyzed system, leading to *gem*-bis(boryl)cyclopropane 3u in 78% yield. Several commercially available complex molecules, such as tigogenin and diacetone-*d*-glucose, were converted into corresponding alkene derivatives and subjected to the photoinduced synthesis protocol, resulting in corresponding products (3v and 3w) in 63% and 66% yields, respectively. A compound with a ketal group, derived from epiandrosterone, exhibited good reactivity for photochemical functionalization (3x). Additionally, when a vitamin E derivative was subjected to the reaction conditions, cyclized product 3y was obtained in a 71% yield. A more complex steroidal compound also performed well under the reaction conditions to deliver the corresponding product (3z). Under standard conditions, the reaction of 2b, the brominated *gem*-diboryl analogue of 2a, with 1a resulted in the corresponding product, as confirmed by GC-MS. However, 2c, the chlorinated analogue of 2a proved ineffective in this system, failing to produce the desired product (see ESI† for details).

**Scheme 2:**
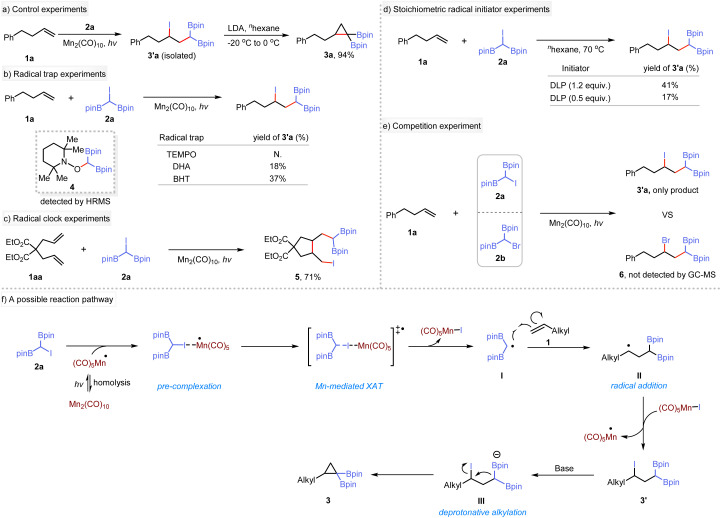
Mechanistic studies.

To elucidate the reaction mechanism, a series of experiments were conducted. The reaction of 1a with 2a was carried out using Mn_2_(CO)_10_ as a catalyst under 440 nm blue LED irradiation, yielding the *γ*-iodo-*gem*-diborylalkane 3′a. Subsequent treatment of 3′a with LDA produced the target product, indicating the Mn-catalyzed step as a crucial stage in the synthesis of cyclopropanes (Scheme 2a). Under standard conditions, the presence of radical traps such as 2,2,6,6-tetramethyl-1-piperidinyloxy (TEMPO), 9,10-dihydroanthracene (DHA), or butylated hydroxytoluene (BHT) inhibited the photoinduced difunctionalization reaction (Scheme 2b). Instead, a radical adduct 4 was identified using high-resolution mass spectrometry. These results support the formation of *gem*-diboryl carbon-centered radical species during this process. Subsequently, the difunctionalization of diene substrate 1aa was conducted under standard conditions (Scheme 2c). Interestingly, only rearrangement product 5 was detected, indicating that the iodination reaction occurs after the migrational ring-closing step. Additionally, 5 underwent intramolecular cyclization when treated with NaO^*t*^Bu, yielding a bicyclic monoboronate ester 5a (see ESI† for details). To elucidate further the reaction mechanism, we used stoichiometric initiators instead of Mn_2_(CO)_10_ for the XAT reaction between 1a and 2a. Thus, 1.2 equivalents of dilauroyl peroxide (DLP) produced 41% of the desired product compared to only 17% yield when using 0.5 equivalents (Scheme 2d). We extended these studies to include the common radical initiators AIBN and BEt_3_ (see ESI†), which showed similar behavior. In addition, a competition experiment was conducted using 1a and two different α-halogenated diboron compounds (2a and 2b). Only the iodine-containing product was observed by NMR or GC-MS (Scheme 2e). These results imply that a radical propagation pathway is unlikely to be operative in this transformation. Based on these observations, a plausible mechanism for the reaction is proposed (Scheme 2f), although other mechanism may be possible. (1) Homolysis of Mn_2_(CO)_10_ precatalyst under irradiation with a blue LED forms the (CO)_5_Mn˙ radical,^24^ which serves as the active catalyst for subsequent reactions.^21*e*,25^ (2) The (CO)_5_Mn˙ radical species acts as an iodine atom extractor from substrate 2a, forming the Mn(CO)_5_I complex and a *gem*-diboryl carbon-centered radical I. This step involves the transfer of the iodine atom from 2a to the catalytic species. (3) The *gem*-diboryl carbon-centered radical I undergoes radical addition to an alkene,^26^ generating an alkyl radical II. This step introduces the alkyl radical functionality into the reaction. (4) The alkyl radical II undergoes atom transfer using (CO)_5_Mn–I as an oxidant to form the adduct III. Simultaneously, the (CO)_5_Mn˙ catalyst is regenerated. (5) Compound III further undergoes intramolecular nucleophilic cyclization in the presence of base^27^ to give the final product.

**Scheme 3:**
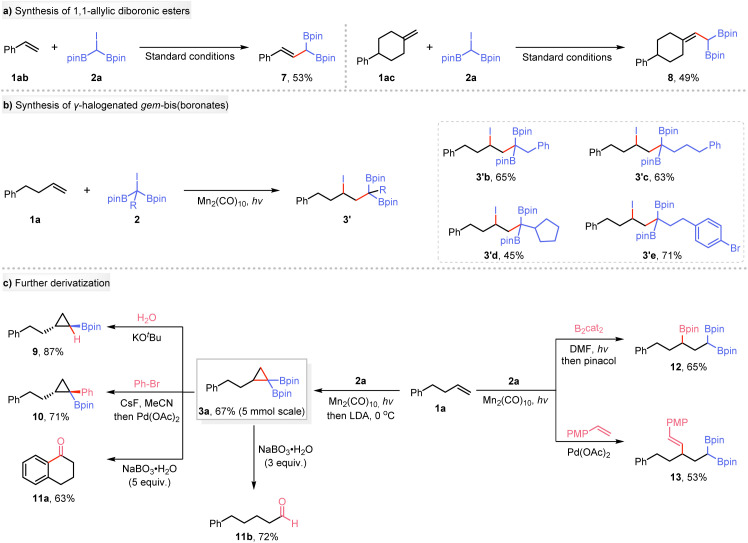
Synthetic diversification and applications. PMP = 4-methoxyphenyl.

To demonstrate the versatility and practicality of this system, we conducted a series of synthetic experiments. Thus, styrene or a 2,2-disubstituted alkene, under our conditions, did not produce cyclopropanes; instead, 1,1-allylic diboronic esters 7 and 8 were formed in yields of 53% and 49%, respectively (Scheme 3a). Subsequently, substituted iododiboryl alkanes lacking α-C(sp^3^)–H bonds were reacted photochemically with unactivated olefins, yielding products containing tetrasubstituted carbon centers (3′b–3′e) in good to moderate yields (Scheme 3b). A large-scale reaction was conducted with 1a and 2a under the standard conditions, resulting in a 67% isolated yield of product 3a, which possesses the potential for further transformations (Scheme 3c). Treatment of 3a with KO^*t*^Bu enabled it to undergo deborylative protonation, affording 1,2-substituted cyclopropylboronate 9 in 87% yield. The Suzuki–Miyaura coupling of *gem*-di(boryl)cyclopropane 3a with bromobenzene afforded coupling product 10 in 71% yield. Interestingly, compound 3a was oxidized with 5 equivalents of NaBO_3_·H_2_O to produce benzocyclohexanone (11a) with a moderate yield, whereas using 3 equivalents of NaBO_3_·H_2_O gave 5-phenylpentanal (11b). Additionally, we also explored one-pot synthetic routes, *i.e.*, a radical borylation^28^ to produce 1,1,3-alkyltriboronate (12), and a Heck-type cross-coupling^29^ to yield γ-substituted *gem*-diborylalkanes (13).

## Conclusions

In summary, we developed a general and versatile approach for the synthesis of *gem*-di(boryl)cyclopropanes *via* manganese catalysis, starting from readily available alkenes and (diborylmethyl)iodides. This transformation features operational simplicity, exceptional catalytic efficiency, excellent tolerance toward different functional groups, and applicability for late-stage modification of complex molecules. Furthermore, this method also provides an efficient route to synthesize previously inaccessible γ-iodo-*gem*-diborylalkanes. The multifunctional compounds obtained from this method serve as versatile building blocks for further transformations, which offer opportunities for synthesizing diverse molecular architectures. Mechanistic experiments support the proposed Mn-catalyzed atom transfer radical addition, followed by a base-mediated intramolecular dehydrocyclization pathway. Given the synthetic importance of *gem*-di(boryl)cyclopropanes and broad interest in XAT chemistry, we anticipate that this methodology will find extensive application in synthetic chemistry and inspire further exploration of novel multifunctional reagents, serving as a key to unlocking synthetic challenges for diverse and intricate molecular architectures.

## Author contributions

J. H. conceived and directed the project. K. Z. and M. H. discovered and developed the reaction. K. Z., J. W., and S. C. performed the experiments and collected the data. Z. S. and T. B. M. co-supervised the project. All authors discussed and analyzed the data. J. H. and T. B. M. wrote the manuscript with contribution from other authors.

## Conflicts of interest

The authors declare no competing financial interest.

## Supplementary Material

SC-016-D5SC02670A-s001

SC-016-D5SC02670A-s002

## Data Availability

ESI† is available and includes the experimental procedures, characterization data and crystallographic data for 3c. Deposition number 2382460 (for 3c) contains the supplementary crystallographic data for this paper.
