# Supplementary material for: Synthesis of gem-di(boryl)cyclopropanes from non-activated olefins via Mn-photocatalyzed atom transfer radical addition
Source: Chem Sci. 2025 May 12;16(23):10595–601. doi: 10.1039/d5sc02670a (PMC12079576; doi:10.1039/d5sc02670a)

Supplementary Information (SI) for Chemical Science.
This journal is © The Royal Society of Chemistry 2025

Supporting Information

Synthesis of *gem*-Di(boryl)cyclopropanes from Non-Activated Olefins via Mn-Photocatalyzed Atom Transfer Radical Addition

Jiefeng Hu,*[a] Kun Zhang,[b] Jing Wang,[b] Mingming Huang,[c] Shuangru Chen,[a] Zhuangzhi Shi,[d] and Todd B Marder*[c]

[a] State Key Laboratory of Flexible Electronics (LoFE) & Institute of Advanced Materials (IAM), Nanjing University of Posts & Telecommunications, 9 Wenyuan Road, Nanjing 210023, China

[b] School of Chemistry and Molecular Engineering, Nanjing Tech University, Nanjing 211816, China

[c] Institut für Anorganische Chemie and Institute for Sustainable Chemistry & Catalysis with Boron, Julius-Maximilians-Universität Würzburg, Am Hubland, 97074 Würzburg, Germany

[d] State Key Laboratory of Coordination Chemistry, School of Chemistry and Chemical Engineering, Nanjing University, Nanjing 210023, China

E-mail: iamjfhu@njupt.edu.cn; todd.marder@uni-wuerzburg.de

Table of Contents

1 Experimental Section S1

1.1 General Considerations S1

1.2 Optimization of the Reaction Conditions S3

1.3 Preparation of Starting Materials S6

1.4 Details for the Synthesis of *gem*-Bis(boryl)cyclopropanes S13

2 Synthetic Diversification and Applications S34

3 Mechanistic Investigations S30

4 Single-Crystal X-Ray Diffraction Analysis S42

5 References S45

6 NMR Spectra S47

1 Experimental Section

1.1 General Considerations

All reactions and subsequent manipulations were performed under an argon atmosphere using standard Schlenk techniques or in a glovebox (Innovative Technology Inc. and Braun Uni Lab). All reactions were carried out in oven-dried glassware. Reagent grade solvents were argon or nitrogen saturated and were dried and deoxygenated using an Innovative Technology Inc. Pure-Solv 400 Solvent Purification System, and further deoxygenated using the freeze-pump-thaw method. $CDCl_3$ was purchased from Sigma-Aldrich. The diboron reagents B_2pin_2 and B_2cat_2 were generous gifts from AllyChem Co. Ltd. $CH_2(Bpin)_2$ and $CHI(Bpin)_2$ were synthesized according to our previous report.[1,2] All other reagents were purchased from Alfa-Aesar, Sigma-Aldrich or J&K Scientific, and were checked for purity by GC-MS and/or ^1H NMR spectroscopy and used as received.

NMR spectra were recorded at 298 K using Bruker Avance 300 (^1H, 300 MHz; ^{13}C, 75 MHz, ^{11}B, 96 MHz) or Bruker DPX-400 (^1H, 400 MHz; ^{13}C, 101 MHz; ^{11}B, 128 MHz; ^{19}F, 376 MHz) spectrometers. ^1H NMR chemical shifts are reported relative to TMS and were referenced *via* residual proton resonances of the corresponding deuterated solvent ($CDCl_3$: 7.26 ppm) whereas $^{13}C\{^1H\}$ NMR spectra are reported relative to TMS using the natural-abundance carbon resonances ($CDCl_3$: 77.16 ppm). However, signals for the carbon attached to boron, C−B, are usually too broad to observe in the $^{13}C\{^1H\}$ NMR spectra. ^{11}B and ^{19}F NMR chemical shifts are reported relative to external $BF_3•OEt_3$ and $CFCl_3$, respectively. Coupling constants are given in Hertz. Automated flash chromatography was performed using a Biotage® Isolera Four system, on silica gel (Biotage SNAP cartridge KP-Sil 10 g and KP-Sil 25 g). Commercially available, precoated TLC plates (Polygram® Sil G/UV254) were purchased from J&K Scientific. The removal of solvent was performed on a rotary evaporator *in vacuo* at a maximum temperature of 40 °C. GC-MS analyses were performed using a Thermo Fisher Scientific Trace 1310 gas chromatograph (column: TG-SQC 5% phenyl methyl siloxane, 15 m, ∅ 0.25 mm, film 0.25 μm; injector: 250 °C; oven: 40 °C (2 min), 40 °C to 280 °C; carrier gas: He (1.2 mL min^{-1}) or an Agilent 7890A gas chromatograph (column: HP-5MS 5% phenyl methyl siloxane, 30 m, Ø 0.25 mm, film 0.25 μm; injector: 250 °C; oven: 40 °C (2 min), 40 °C to 280 °C (20 °C min^{-1}); carrier gas: He (1.2 mL min^{-1}) equipped with an Agilent 5975C inert MSD with triple-axis detector operating in EI mode and an Agilent 7693A series auto sampler/injector. High-resolution mass spectra were obtained using a Thermo Scientific Exactive Plus spectrometer equipped with an Orbitrap Mass Analyzer. Measurements were accomplished using an ASAP/APCI source with a corona needle, and a carrier-gas (N_2) temperature of 250 °C.

General photophysical measurements. All photoredox reactions were performed in a 10 mL thick-walled reaction tube. The setup of the photoredox reaction (Figure S1) was assembled with a Kessil^{TM} lamp (440 nm at 50% power, 20 W). The reaction tube was placed 3 cm in front of the Kessil^{TM} lamp with 1 fan for cooling.

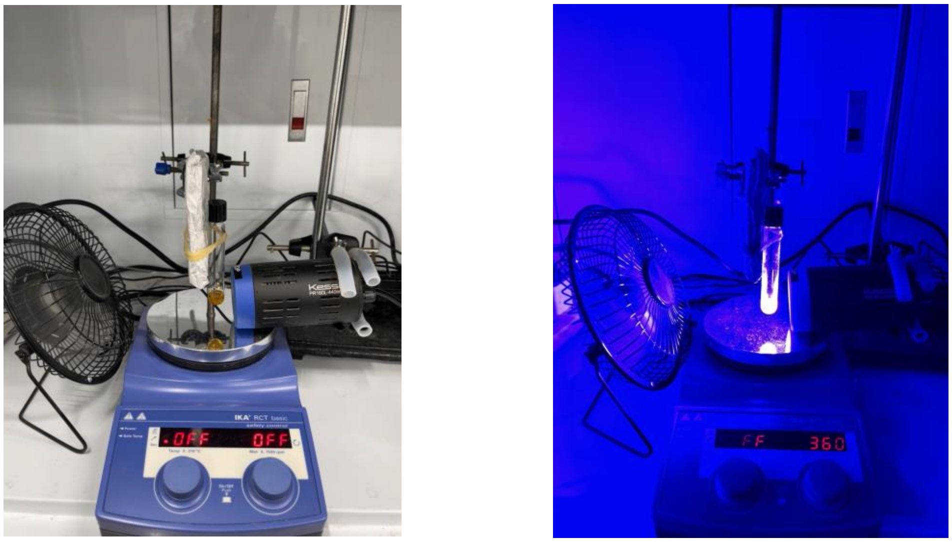

Figure S1. Set-up used for the photoredox reactions

1.2 Optimization of the Reaction Conditions

General procedure of optimization

In an argon-filled glovebox, (diboronmethyl)iodides **2a** ($CHI(Bpin)_2$, 0.36 mmol, 1.2 equiv.), catalyst (2 mol% - 15 mol%), solvent (1 mL) and alkenes **1a** (0.3 mmol, 1 equiv.) were sequentially added to 10 mL vial equipped with a magnetic stirring bar. The reaction tube was then sealed with a rubber cap and removed from the glovebox. It was placed 3 cm in front of a Kessil Lamp with 1 fan for cooling. The reaction tube was irradiated for 0.5 h - 3 h. After irradiation, the reaction mixture was cooled to -20 °C, and base (1.2 equiv.) was added, followed by stirring for 2 h at 0 ^{o}C. Upon completion, the reaction tube was charged with dodecane as an internal standard and the crude reaction mixture was analyzed by GC-MS.

Table S1: Screening of photocatalysts.

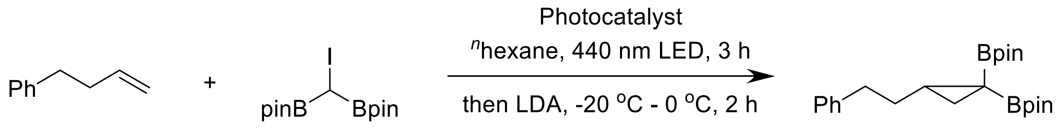

Entry	Photocatalyst	Yield of **3a** (%)
1	\	0
2	*fac*-$Ir(ppy)_3$ (2 mol%)	0
3	$[Ru(bpy)_3]Cl_2$ (2 mol%)	0
4	$Ru(dtbpy)_3(PF_6)_2$ (2 mol%)	0
5	4CzIPN (10 mol%)	<10
6	Eosin Y (10 mol%)	0
7	$[Mes\text{-}Acr]^+ [BF_4]^-$ (10 mol%)	0
8	$[Mes\text{-}Acr\text{-}Ph]^+(Cl)^-$ (10 mol%)	0
9	$Mn(CO)_5Br$ (10 mol%)	27
10	$Mn_2(CO)_{10}$ (5 mol%)	71
11	$Mn_2(CO)_{10}$ (10 mol%)	90
12	$Mn_2(CO)_{10}$ (15 mol%)	93

Reaction conditions: **1a** (0.3 mmol), **2a** (0.36 mmol), catalyst in ^{n}hexane (1 mL), 3 h, 440 nm blue LED, 25 °C - 45 °C, under argon. Then the reaction mixture was cooled to -20 °C, and LDA (2.5 M, 1.2 equiv. in THF) was added, followed by stirring for 2 h at 0 ^{o}C. The yields of **3a** were determined by GC-MS analysis using an internal standard.

Table S2: Screening of bases.

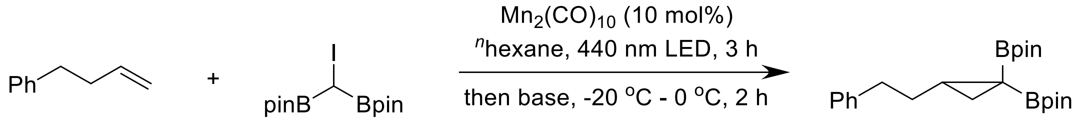

1a 2a 3a

Entry	base	Yield of **3a** (%)
1	LiO^{t}Bu	0
2	LiOMe	0
3	NaH	0
4	LDA	90
8	LTMP	87
9	^{n}BuLi	22

Reaction conditions: **1a** (0.3 mmol), **2a** (0.36 mmol), $Mn_2(CO)_{10}$ in ^{n}hexane (1 mL), 3 h, 440 nm blue LED, 25 °C – 45 °C, under argon. Then the reaction mixture was cooled to -20 °C, and base (1.2 equiv.) was added, followed by stirring for 2 hours at 0 °C. The yields of **3a** were determined by GC-MS analysis using an internal standard.

Table S3: Screening of solvents.

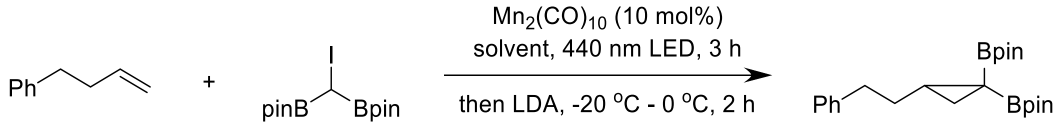

1a 2a 3a

Entry	solvent	Yield of **3a** (%)
1	DMSO	0
2	DMA	0
3	DMF	0
4	toluene	<10
5	1,4-dioxane	<10
6	THF	<10
7	MeCN	0
8	DCM	47
9	^{n}octane	73

Reaction conditions: **1a** (0.3 mmol), **2a** (0.36 mmol), $Mn_2(CO)_{10}$ in solvent (1 mL), 3 h, 440 nm blue LED, 25 °C – 45 °C, under argon. Then the reaction mixture was cooled to -20 °C, and LDA (2.5 M, 1.2 equiv. in THF) was added, followed by stirring for 2 h at 0 °C. The yields of **3a** were determined by GC-MS analysis using an internal standard.

Table S4: Screening of reaction time.

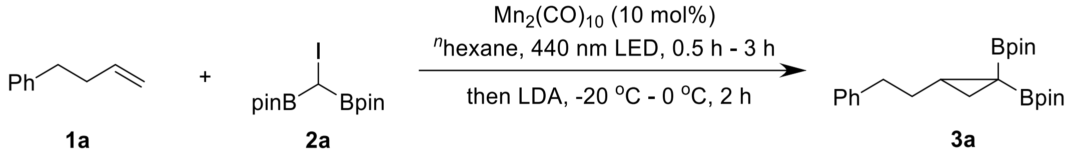

Entry	Time (min)	Yield of **3a** (%)
1	30	68
2	60	76
3	90	83
4	120	89
5	180	90

Reaction conditions: **1a** (0.3 mmol), **2a** (0.36 mmol), $Mn_2(CO)_{10}$ in ^{n}hexane (1 mL), 0.5 h - 3 h, 440 nm blue LED, 25 °C – 45 °C, under argon. Then the reaction mixture was cooled to -20 °C, and LDA (2.5 M, 1.2 equiv. in THF) was added, followed by stirring for 2 h at 0 °C. The yields of **3a** were determined by GC-MS analysis using an internal standard.

Table S5: Screening of light intensity.

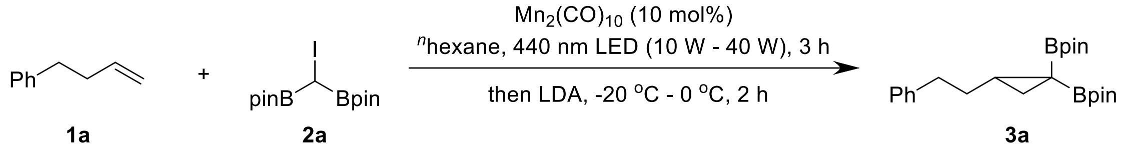

Entry	Power (W)	Yield of **3a** (%)
1	10	86
2	20	88
3	30	87
4	40	85

Reaction conditions: **1a** (0.3 mmol), **2a** (0.36 mmol), $Mn_2(CO)_{10}$ in ^{n}hexane (1 mL), 3 h, 440 nm blue LED (10 W – 40 W), 25 °C – 45 °C, under argon. Then the reaction mixture was cooled to -20 °C, and LDA (2.5 M, 1.2 equiv. in THF) was added, followed by stirring for 2 h at 0 °C. The yields of **3a** were determined by GC-MS analysis using an internal standard.

1.3 Preparation of Starting Materials

General Procedure A

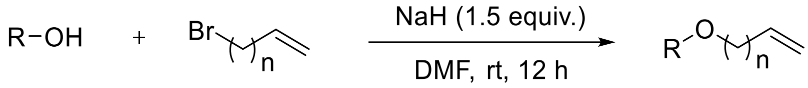

To a solution of alkyl alcohol (5 mmol, 1 equiv.) in dry DMF (15 mL) was added NaH (7.5 mmol, 60 wt% in mineral oil, 1.5 equiv.) at 0 ºC, and the reaction mixture was stirred at 0 ºC for for 30 min. The brominated olefin (7.5 mmol, 1.5 equiv.) was added dropwise. The reaction was stirred for 12 h at room temperature. The mixture was diluted with NH_4Cl and EtOAc. The organic layer was separated, and the aqueous layer was extracted twice with EtOAc. The combined organic layers were dried over Na_2SO_4, filtered, and concentrated *in vacuo*. The crude products were purified by column chromatography (PE/EtOAc) to give the corresponding alkenes.[3]

General Procedure B

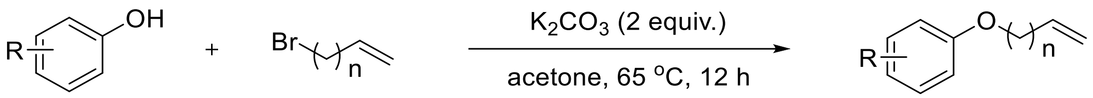

To a solution of phenol (5 mmol, 1 equiv.) and potassium carbonate (1.38 g, 10 mmol, 2 equiv.) in acetone (25 mL) was added the respective brominated olefin (7.5 mmol, 1.5 equiv.), and the reaction mixture was stirred at 65 ºC for 12 h. The mixture was then allowed to cool to room temperature, diluted with ether and quenched with water. The organic layer was separated, and the aqueous layer was extracted twice with ether. The combined organic layers were dried over Na_2SO_4, filtered, and concentrated *in vacuo*. The crude products were purified by column chromatography (PE/EtOAc) to give the corresponding alkenes.[4]

General Procedure C

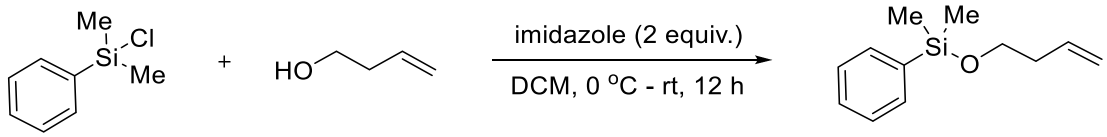

To a solution of but-3-en-1-ol (5 mmol, 1 equiv.) and imidazole (10 mmol, 2 equiv.) in DCM (15 mL) was added chlorodimethyl(phenyl)silane (5.5 mmol, 1.1 equiv.) at 0 °C, and the reaction mixture was stirred at 0 ºC for for 1 h. Then the reaction was stirred for 12 h at room temperature. The suspension was diluted with water and DCM. The organic layer was separated, and the aqueous layer was extracted twice with DCM. The combined organic layers were washed with brine, dried over Na_2SO_4, filtered, and concentrated *in vacuo*. The crude product was purified by column chromatography (PE/EtOAc) to give the corresponding alkynes.[5]

General Procedure D

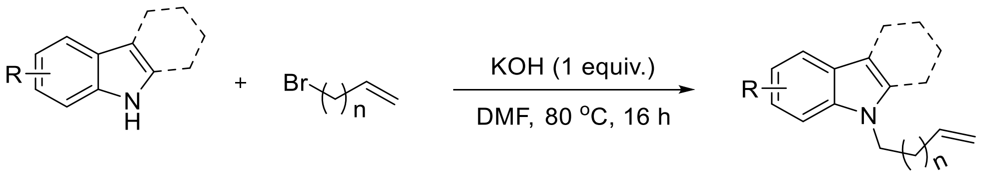

A 100-mL oven-dry Schlenk tube equipped with a magnetic stir bar was charged with brominated olefin (0.5 mL, 4 mmol), KOH (112 mg, 2 mmol), and DMF (20 mL). The *N*-heterocycle (335 mg, 2 mmol) was slowly added to the reaction mixture at 0 °C. The resulting mixture was stirred at 80 °C for 16 h while being monitored by TLC. After being quenched with water, the reaction mixture was extracted with ethyl acetate (3 × 30 mL). The organic layer was washed with brine, dried over $MgSO_4$, and evaporated under reduced pressure. The crude product was purified by flash column chromatography using a petroleum ether/ethyl acetate as the eluent.[6]

((allyloxy)methyl)benzene (1i)

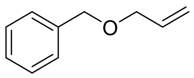

1i

According to **General procedure A,** the crude product was purified by flash chromatography on silica gel to give the product **1i** as a colorless oil (5 mmol scale, 0.56 g, 76% yield). **^{1}H NMR** (400 MHz, $CDCl_3$): δ 7.50 – 7.41 (m, 4H), 7.40 – 7.33 (m, 1H), 6.13 – 5.99 (m, 1H), 5.42 (d, *J* = 17 Hz, 1H), 5.31 (d, *J* = 10 Hz, 1H), 4.62 (s, 2H), 4.13 (d, *J* = 6 Hz, 2H). **^{13}C NMR** (101 MHz, $CDCl_3$) *δ* 138.4, 134.9, 128.5, 127.8, 127.7, 117.1, 72.2, 71.2.

The spectroscopic data for **1i** match those reported in the literature.[7]

1-fluoro-4-(hex-5-en-1-yloxy)benzene (1j)

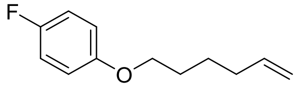

1j

According to **General procedure B,** the crude product was purified by flash chromatography on silica gel to give the product **1j** as a colorless oil (5 mmol scale, 0.8 g, 82% yield). **^{1}H NMR** (400 MHz, $CDCl_3$): δ 7.02 – 6.96 (m, 2H), 6.88 – 6.83 (m, 2H), 5.94 – 5.78 (m, 1H), 5.11 – 5.04 (m, 1H), 5.03 – 4.99 (m, 1H), 3.95 (t, *J* = 6 Hz, 2H), 2.19 – 2.13 (m, 2H), 1.86 – 1.77 (m, 2H), 1.65 – 1.55 (m, 2H). **^{13}C NMR** (101 MHz, $CDCl_3$) *δ* 157.1 (d, *J* = 238 Hz), 155.2 (d, *J* = 2 Hz), 138.5, 115.7 (d, *J* = 23 Hz), 115.4 (d, *J* = 8 Hz), 114.8, 68.4, 33.4, 28.7, 25.3. **^{19}F NMR** (376 MHz, $CDCl_3$) *δ* -124.4.

The spectroscopic data for **1j** match those reported in the literature.[8]

1-bromo-4-(hex-5-en-1-yloxy)benzene (1l)

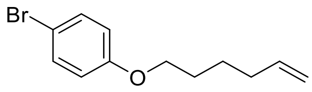

1l

According to **General procedure B,** the crude product was purified by flash chromatography on silica gel to give the product **1l** as a colorless oil (5 mmol scale, 1.03 g, 81% yield). ^{1}**H NMR** (400 MHz, $CDCl_3$): δ 7.42 – 7.35 (m, 2H), 6.82 – 6.78 (m, 2H), 5.86 (ddt, J = 17, 10, 7 Hz, 1H), 5.11 – 5.04 (m, 1H), 5.03 – 4.99 (m, 1H), 3.95 (t, J = 6 Hz, 2H), 2.16 (q, J = 8 Hz, 2H), 1.86 – 1.78 (m, 2H), 1.63 – 1.56 (m, 2H). ^{13}**C NMR** (101 MHz, $CDCl_3$) δ 158.2, 138.5, 132.2, 116.3, 114.9, 112.6, 68.0, 33.4, 28.6, 25.3.

The spectroscopic data for **1l** match those reported in the literature.[9]

1-(hex-5-en-1-yloxy)-4-iodobenzene (1m)

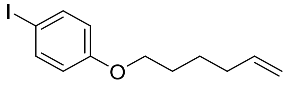

1m

According to **General procedure B,** the crude product was purified by flash chromatography on silica gel to give the product **1l** as a colorless oil (5 mmol scale, 1.18 g, 78% yield). ^{1}**H NMR** (400 MHz, $CDCl_3$): δ 7.59 – 7.55 (m, 2H), 6.72 – 6.68 (m, 2H), 5.85 (ddt, J = 17, 10, 7 Hz, 1H), 5.11 – 5.03 (m, 1H), 5.02 – 4.99 (m, 1H), 3.94 (t, J = 6 Hz, 2H), 2.18 – 2.12 (m, 2H), 1.84 – 1.76 (m, 2H), 1.62 – 1.55 (m, 2H). ^{13}**C NMR** (101 MHz, $CDCl_3$) δ 159, 138.5, 138.2, 116.9, 114.9, 82.5, 67.9, 33.4, 28.6, 25.3.

The spectroscopic data for **1m** match those reported in the literature.[10]

1-(hex-5-en-1-yloxy)-4-iodobenzene (1q)

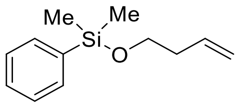

1q

According to **General procedure C,** the crude product was purified by flash chromatography on silica gel to give the product **1q** as a colorless oil (5 mmol scale, 0.74 g, 72% yield). ^{1}**H NMR** (400 MHz, $CDCl_3$): δ 7.73 – 7.63 (m, 2H), 7.53 – 7.42 (m, 3H), 5.89 (ddt, J = 17, 10, 7 Hz, 1H), 5.19 – 5.13 (m, 1H), 5.13 – 5.10 (m, 1H), 3.75 (t, J = 7 Hz, 2H), 2.39 (qt, J = 7, 1 Hz, 2H), 0.49 (s, 6H). ^{13}**C NMR** (101 MHz, $CDCl_3$) δ 138, 135.2, 133.6, 129.7, 127.9, 116.6, 62.7, 37.3, -1.7.

The spectroscopic data for **1q** match those reported in the literature.[11]

9-(pent-4-en-1-yl)-9H-carbazole (1s)

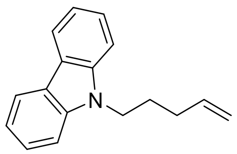

1s

According to **General procedure D,** the crude product was purified by flash chromatography on silica gel to give the product **1s** as a colorless oil (5 mmol scale, 0.76 g, 65% yield). **^{1}H NMR** (400 MHz, $CDCl_3$): δ 8.23 (d, J = 8 Hz, 2H), 7.59 (t, J = 8 Hz, 2H), 7.51 (d, J = 8 Hz, 2H), 7.36 (t, J = 7 Hz, 2H), 5.94 (ddt, J = 17, 10, 7 Hz, 1H), 5.22 – 5.11 (m, 2H), 4.39 (t, J = 7 Hz, 2H), 2.24 (q, J = 7 Hz, 2H), 2.07 (p, J = 7 Hz, 2H). **^{13}C NMR** (101 MHz, $CDCl_3$) δ 140.5, 137.6, 125.7, 123, 120.5, 118.9, 115.6, 108.8, 42.4, 31.2, 28.

The spectroscopic data for **1s** match those reported in the literature.[12]

6-chloro-1-(pent-4-en-1-yl)-1H-indole (1t)

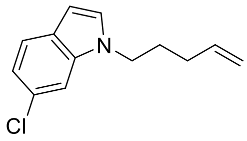

1t

According to **General procedure D,** the crude product was purified by flash chromatography on silica gel to give the product **1t** as a colorless oil (5 mmol scale, 0.67 g, 61% yield). **^{1}H NMR** (400 MHz, $CDCl_3$): δ 7.55 (d, J = 8 Hz, 1H), 7.35 (s, 1H), 7.19 – 6.99 (m, 2H), 6.49 (d, J = 4 Hz, 1H), 5.92 – 5.73 (m, 1H), 5.15 – 5.01 (m, 2H), 4.09 (t, J = 7 Hz, 2H), 2.15 – 2.02 (m, 2H), 2.01 – 1.85 (m, 2H). **^{13}C NMR** (101 MHz, $CDCl_3$) δ 137.2, 136.4, 128.6, 127.5, 127.2, 121.8, 120, 115.9, 109.5, 101.3, 45.7, 30.8, 29.1.

The spectroscopic data for **1t** match those reported in the literature.[13]

(1R,4S)-1-isopropyl-4-methyl-2-(pent-4-en-1-yloxy)cyclohexane (1u)

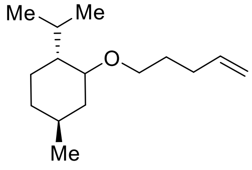

1u

According to **General procedure A,** the crude product was purified by flash chromatography on silica gel to give the product **1u** as a colorless oil (3 mmol scale, 0.35 g, 53% yield). **^{1}H NMR** (400 MHz, $CDCl_3$): δ 5.85 (ddt, J = 17, 10, 7 Hz, 1H), 5.10 – 5.02 (m, 1H), 5.02 – 4.93 (m, 1H), 3.65 (dt, J = 9, 6 Hz, 1H), 3.29 (dt, J = 9, 6 Hz, 1H), 3.02 (td, J = 11, 4 Hz, 1H), 2.31 – 2.20 (m, 1H), 2.19 – 2.07 (m, 3H), 1.77 – 1.57 (m, 5H), 1.41 – 1.29 (m, 1H), 1.28 – 1.19 (m, 1H), 1.06 – 0.96 (m, 1H), 0.94 (d, J = 4 Hz, 3H),

0.92 (d, J = 4 Hz, 3H), 0.89 – 0.82 (m, 1H), 0.79 (s, d, J = 8 Hz, 3H). ^{13}**C NMR** (101 MHz, $CDCl_3$) δ 138.5, 114.6, 79.2, 67.8, 48.3, 40.5, 34.6, 31.6, 30.5, 29.5, 25.6, 23.4, 22.4, 21, 16.2.

The spectroscopic data for **1u** match those reported in the literature.[14]

(2aR,4R,5'R,6aR,6bR,8aR,8bS,9R,10S,11aR,12aR,12bS)-5',6a,8a,9-tetramethyl-4-(pent-4-en-1-yloxy)docosahydrospiro[naphtho[2',1':4,5]indeno[2,1-b]furan-10,2'-pyran] (1v)

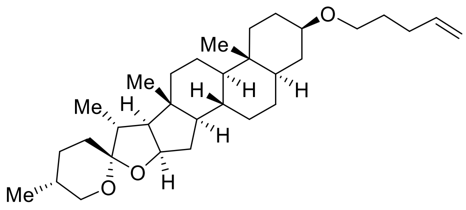

1v

According to **General procedure A,** the crude product was purified by flash chromatography on silica gel to give the product **1v** as a colorless oil (3 mmol scale, 0.65 g, 45% yield). ^{1}**H NMR** (400 MHz, $CDCl_3$): δ 5.83 (ddt, J = 17, 10, 7 Hz, 1H), 5.07 – 4.95 (m, 2H), 4.44 – 4.37 (m, 1H), 3.52 – 3.43 (m, 3H), 3.39 (t, J = 11 Hz, 1H), 3.26 – 3.14 (m, 1H), 2.13 (q, J = 8 Hz, 2H), 2.04 – 1.95 (m, 1H), 1.90 – 1.83 (m, 2H), 1.80 – 1.72 (m, 3H), 1.70 – 1.61 (m, 8H), 1.59 – 1.47 (m, 3H), 1.44 – 1.36 (m, 1H), 1.34 – 1.21 (m, 5H), 1.17 – 1.04 (m, 3H), 0.98 (d, J = 7 Hz, 3H), 0.95 – 0.85 (m, 2H), 0.83 – 0.76 (m, 9H), 0.70 – 0.60 (m, 1H). ^{13}**C NMR** (101 MHz, $CDCl_3$) δ 138.5, 114.6, 109.3, 80.9, 78.5, 67.3, 66.8, 62.2, 56.3, 54.4, 44.9, 41.6 40.6, 40.1, 37.0, 35.9, 35.1, 34.9, 32.3, 31.8, 31.4, 30.4, 30.3, 29.4, 28.80, 28.76, 28.4, 21.0, 17.1, 16.5, 14.5, 12.3. **HRMS-ESI** (m/z): Calculated (found) for $C_{32}H_{53}O_3$ $[M+H]^+$ 485.3989 (485.3982).

(3aR,5R,6R,6aR)-5-((R)-2,2-dimethyl-1,3-dioxolan-4-yl)-6-(hex-5-en-1-yloxy)-2,2-dimethyltetrahydrofuro[2,3-d][1,3]dioxole (1w)

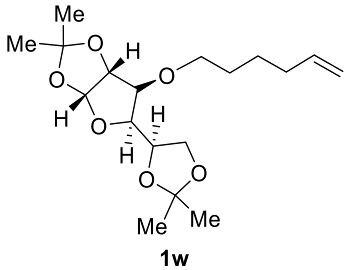

1w

According to **General procedure A,** the crude product was purified by flash chromatography on silica gel to give the product **1w** as a colorless oil (3 mmol scale, 0.61 g, 59% yield). ^{1}**H NMR** (400 MHz, $CDCl_3$): δ 5.91 – 5.85 (m, 1H), 5.84 – 5.72 (m, 1H), 5.10 – 4.80 (m, 2H), 4.53 (d, J = 4 Hz, 1H), 4.31 (q, J = 7 Hz, 1H), 4.14 – 4.05 (m, 2H), 4.01 – 3.95 (m, 1H), 3.87 – 3.82 (m, 1H), 3.65 – 3.57 (m, 1H), 3.56 – 3.48 (m, 1H), 2.10 – 2.03 (m, 2H), 1.62 – 1.54 (m, 2H), 1.50 (d, J = 1 Hz, 3H), 1.48 – 1.39 (m, 5H),

1.35 (s, 3H), 1.32 (s, 3H). **^{13}C NMR** (101 MHz, $CDCl_3$) δ 138.6, 114.6, 111.7, 108.9, 105.3, 82.5, 82.1, 81.2, 72.5, 70.4, 67.2, 33.4, 29.1, 26.8, 26.8, 26.2, 25.4, 25.3.

The spectroscopic data for **1w** match those reported in the literature.[15]

(3S,5S,8R,9S,10S,13S,14S)-10,13-dimethyl-3-(pent-4-en-1-yloxy)hexadecahydrospiro[cyclopenta[a]phenanthrene-17,2'-[1,3]dioxolane] (1x)

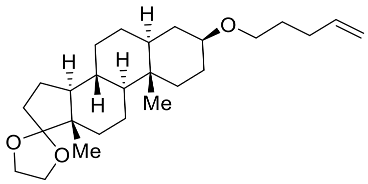

1x

According to **General procedure A,** the crude product was purified by flash chromatography on silica gel to give the product **1x** as a colorless oil (3 mmol scale, 0.49 g, 41% yield). **^{1}H NMR** (400 MHz, $CDCl_3$): δ 5.84 (ddt, J = 17, 10, 7 Hz, 1H), 5.08 – 4.94 (m, 2H), 3.97 – 3.84 (m, 4H), 3.54 – 3.41 (m, 2H), 3.27 – 3.15 (m, 1H), 2.13 (q, J = 7 Hz, 2H), 2.05 – 1.93 (m 1H), 1.89 – 1.70 (m, 4H), 1.66 (d, J = 7 Hz, 3H), 1.64 – 1.49 (m, 3H), 1.46 – 1.33 (m, 4H), 1.31 – 1.20 (m, 5H), 1.11 – 1.03 (m, 1H), 1.01 – 0.88 (m, 2H), 0.85 (s, 3H), 0.81 (s, 3H), 0.73 – 0.62 (m, 1H). **^{13}C NMR** (101 MHz, $CDCl_3$) δ 138.5, 119.5, 114.6, 78.5, 67.3, 65.2, 64.5, 54.3, 50.4, 46, 44.9, 37.1, 35.9, 35.8, 34.9, 34.2, 31.4, 30.7, 30.4, 29.3, 28.7, 28.3, 22.7, 20.6, 14.4, 12.3. **HRMS-ESI** (m/z): Calculated (found) for $C_{26}H_{43}O_3$ $[M+H]^+$ 403.3207 (403.3198).

(R)-6-(hex-5-en-1-yloxy)-2,5,7,8-tetramethyl-2-((4R,8R)-4,8,12-trimethyltridecyl)chromane (1y)

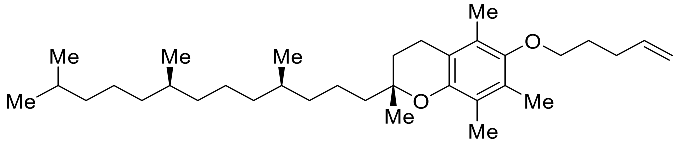

1y

According to **General procedure B,** the crude product was purified by flash chromatography on silica gel to give the product **1y** as a colorless oil (3 mmol scale, 1.12 g, 73% yield). **^{1}H NMR** (400 MHz, $CDCl_3$): δ 5.89 (ddt, J = 17, 10, 7 Hz, 1H), 5.12 – 4.97 (m, 2H), 3.65 (t, J = 7 Hz, 2H), 2.57 (t, J = 7 Hz, 2H), 2.37 – 2.24 (m, 2H), 2.16 (s, 3H), 2.12 (s, 3H), 2.08 (s, 3H), 1.95 – 1.86 (m, 2H), 1.83 – 1.69 (m, 2H), 1.58 – 1.48 (m, 4H), 1.47 – 1.24 (m, 11H), 1.23 (s, 3H), 1.18 – 1.00 (m, 7H), 0.93 – 0.80 (m, 13H). **^{13}C NMR** (101 MHz, $CDCl_3$) δ 148.3, 147.7, 138.3, 127.8, 125.8, 122.8, 117.5, 114.8, 74.7, 72.3, 40.1, 39.4, 37.5, 37.4, 37.3, 32.8, 32.7, 31.3, 30.4, 29.5, 28, 24.8, 24.4, 23.9, 22.7, 22.6, 21.0, 20.7, 19.74, 19.65, 12.7, 11.9, 11.8.

The spectroscopic data for **1y** match those reported in the literature.[16]

<u>(4R,5'S,6aS,6bR,8aR,8bS,9R,10S,11aR,12aR,12bR)-5',6a,8a,9-tetramethyl-4-(pent-4-en-1-yloxy)-1,3,3',4,4',5,5',6,6a,6b,6',7,8,8a,8b,9,11a,12,12a,12b-icosahydrospiro[naphtho[2',1':4,5]indeno[2,1-b]furan-10,2'-pyran] (1z)</u>

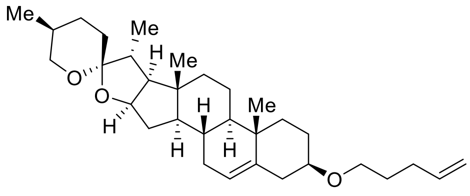

1z

According to **General procedure B,** the crude product was purified by flash chromatography on silica gel to give the product **1z** as a colorless oil (3 mmol scale, 0.72 g, 50% yield). ^{1}**H NMR** (400 MHz, $CDCl_3$): δ 5.79 (ddt, *J* = 17, 10, 7 Hz, 1H), 5.31 (s, 1H), 5.03 – 4.90 (m, 2H), 4.38 (q, *J* = 7 Hz, 1H), 3.49 – 3.39 (m, 3H), 3.34 (t, *J* = 11 Hz, 1H), 3.14 – 3.04 (m, 1H), 2.37 – 2.30 (m, 1H), 2.18 – 2.06 (m, 3H), 2.01 – 1.91 (m, 2H), 1.83 (q, *J* = 7.5 Hz, 3H), 1.75 – 1.40 (m, 15H), 1.31 – 1.05 (m, 4H), 0.99 (s, 3H), 0.94 (d, *J* = 7 Hz, 3H), 0.76 (t, *J* = 3 Hz, 6H). ^{13}**C NMR** (101 MHz, $CDCl_3$) δ 141, 138.2, 121.1, 114.6, 109.1, 80.7, 78.8, 67.2, 66.7, 62, 56.4, 50, 41.5, 40.1, 39.7, 39, 37.1, 36.9, 32, 31.7, 31.3, 31.25, 30.3, 30.2, 29.2, 28.7, 28.3, 20.7, 19.3, 17.1, 16.2, 14.5. **HRMS-ESI** (m/z): Calculated (found) for $C_{32}H_{51}O_3$ $[M+H]^+$ 483.3833 (483.3825).

1.4 Details for the Synthesis of *gem*-Bis(boryl)cyclopropanes

General procedure 1

In an argon-filled glovebox, $CHI(Bpin)_2$ **2a** (0.36 mmol, 1.2 equiv.), $Mn_2(CO)_{10}$ (10 mol%), ^{n}hexane (1 mL) and alkenes **1** (0.3 mmol, 1 equiv.) were sequentially added to 5 mL vial equipped with a magnetic stirring bar. The reaction tube was then sealed with a rubber cap and removed from the glovebox. It was placed 3 cm in front of a Kessil Lamp with 1 fan for cooling. The reaction tube was irradiated for 3 h. After irradiation, the reaction mixture was cooled to -20 °C, and LDA (2.5 M, 1.2 equiv. in THF) was added, followed by stirring for 2 h at 0 °C. The crude product was purified by flash column chromatography (hexane/EtOAc) after careful removal of the solvent *in vacuo*. All alkyl boronate products were unambiguously identified by comparison of HRMS and ^{1}H, ^{13}C{^{1}H}, ^{11}B{^{1}H} and/or ^{19}F{^{1}H} NMR spectra with literature data.

2,2'-(2-phenethylcyclopropane-1,1-diyl)bis(4,4,5,5-tetramethyl-1,3,2-dioxaborolane) (3a)

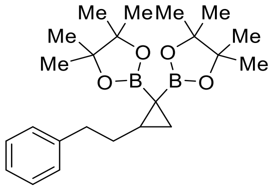

3a

According to **General procedure 1** with but-3-en-1-ylbenzene (39.7 mg, 0.3 mmol, 1 equiv.), the reaction mixture was purified by column chromatography on silica gel (hexane/EtOAc = 50/1) to yield the product **3a** as a colorless oil (96.8 mg, 81% yield).

^{1}H NMR (300 MHz, $CDCl_3$): δ 7.30 – 7.25 (m, 2H), 7.23 – 7.15 (m, 3H), 2.86 – 2.69 (m, 2H), 1.89 – 1.71 (m, 1H), 1.61 – 1.50 (m, 1H), 1.41 – 1.27 (m, 1H), 1.26 (s, 6H), 1.23 (s, 6H), 1.21 (s, 6H), 1.19 (s, 6H), 0.97 (dd, J = 7, 3 Hz, 1H), 0.68 (dd, J = 5, 3 Hz, 1H). **^{13}C NMR** (75 MHz, $CDCl_3$): δ 142.6, 128.4, 128.1, 125.4, 82.9, 82.6, 36.1, 34.8, 25.1, 24.8, 24.5, 24.3, 23.3, 15.8. **^{11}B NMR** (96 MHz, $CDCl_3$): δ 32.8. **HRMS-ESI** (m/z): Calculated (found) for $C_{23}H_{37}B_2O_4$ $[M+H]^+$ 399.2872 (399.2867). **IR** (film): 3054, 2982, 1370, 1317, 1266, 1138, 970, 850, 742, 704 cm^{-1}.

2,2'-(2-pentylcyclopropane-1,1-diyl)bis(4,4,5,5-tetramethyl-1,3,2-dioxaborolane) (3b)

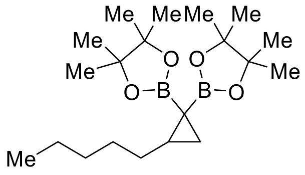

3b

According to **General procedure 1** with hept-1-ene (29.5 mg, 0.3 mmol, 1 equiv.), the reaction mixture was purified by column chromatography on silica gel (hexane/EtOAc = 50/1) to yield the product **3b** as a colorless oil (79.7 mg, 73% yield).

^{1}**H NMR** (400 MHz, $CDCl_3$): δ 1.57 – 1.51 (m, 1H), 1.46 – 1.39 (m, 2H), 1.37 – 1.30 (m, 4H), 1.26 (s, 6H), 1.24 (s, 6H), 1.20 (s, 6H), 1.19 (s, 6H), 1.16 – 1.08 (m, 1H), 0.95 – 0.87 (m, 5H), 0.65 (dd, *J* = 5, 3 Hz, 1H). ^{13}**C NMR** (101 MHz, $CDCl_3$): δ 82.9, 82.5, 32.9, 32, 25.1, 24.8, 24.6, 24.3, 23.7, 22.6, 16, 14.1. ^{11}**B NMR** (128 MHz, $CDCl_3$): δ 33.4. **HRMS-ESI** (m/z): Calculated (found) for $C_{20}H_{39}B_2O_4$ $[M+H]^+$ 365.3029 (365.3020). **IR** (film): 3054, 2982, 1372, 1316, 1266, 1139, 969, 896, 849, 742, 705 cm^{-1}.

2,2'-(2-benzylcyclopropane-1,1-diyl)bis(4,4,5,5-tetramethyl-1,3,2-dioxaborolane) (3c)

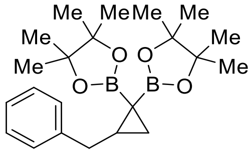

3c

According to **General procedure 1** with allylbenzene (35.5 mg, 0.3 mmol, 1 equiv.), the reaction mixture was purified by column chromatography on silica gel (hexane/EtOAc = 50/1) to yield the product **3c** as a white solid (82.9 mg, 72% yield).

^{1}**H NMR** (400 MHz, $CDCl_3$): δ 7.37 – 7.29 (m, 4H), 7.22 – 7.18 (m, 1H), 3.11 (dd, *J* = 15, 4 Hz, 1H), 2.28 (dd, *J* = 15, 10 Hz, 1H), 1.53 – 1.44 (m, 1H), 1.27 (s, 6H), 1.26 (s, 6H), 1.21 (s, 6H), 1.20 (s, 6H), 1.07 (dd, *J* = 5, 3 Hz, 1H), 0.90 (dd, *J* = 5, 3 Hz, 1H). ^{13}**C NMR** (101 MHz, $CDCl_3$): δ 142.2, 128.4, 128.2, 125.8, 83.1, 82.7, 38.8, 25.1, 24.8, 24.6, 24.4, 24.1, 16.3. ^{11}**B NMR** (128 MHz, $CDCl_3$): δ 33.1. **HRMS-ESI** (m/z): Calculated (found) for $C_{22}H_{35}B_2O_4$ $[M+H]^+$ 385.2716 (385.2709). **IR** (film): 3052, 2980, 1371, 1314, 1268, 896, 849, 742 cm^{-1}.

2,2'-(2-isobutylcyclopropane-1,1-diyl)bis(4,4,5,5-tetramethyl-1,3,2-dioxaborolane) (3d)

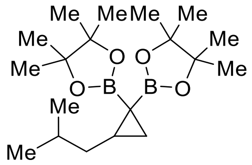

3d

According to **General procedure 1** with 4-methylpent-1-ene (25.3 mg, 0.3 mmol, 1 equiv.), the reaction mixture was purified by column chromatography on silica gel (hexane/EtOAc = 50/1) to yield the product **3d** as a colorless oil (82.9 mg, 79% yield).

^{1}H NMR (400 MHz, $CDCl_3$): δ 1.74 – 1.67 (m, 1H), 1.56 – 1.50 (m, 1H), 1.31 (d, J = 3 Hz, 1H), 1.26 (s, 6H), 1.23 (s, 6H), 1.20 (s, 6H), 1.19 (s, 6H), 0.99 – 0.95 (m, 1H), 0.94 (d, J = 7 Hz, 3H), 0.91 (d, J = 7 Hz, 3H), 0.86 (d, J = 7 Hz, 1H), 0.67 (dd, J = 5, 3 Hz, 1H). **^{13}C NMR** (101 MHz, $CDCl_3$): δ 82.9, 82.5, 42.2, 28.8, 25.1, 24.8, 24.5, 24.3, 22.9, 22.3, 22.1, 16.5. **^{11}B NMR** (128 MHz, $CDCl_3$): δ 33.5. **HRMS-ESI** (m/z): Calculated (found) for $C_{19}H_{37}B_2O_4$ $[M+H]^+$ 351.2872 (351.2865). **IR** (film): 3050, 2982, 1372, 1268, 896, 742 cm^{-1}.

2,2'-(2-cyclohexylcyclopropane-1,1-diyl)bis(4,4,5,5-tetramethyl-1,3,2-dioxaborolane) (3e)

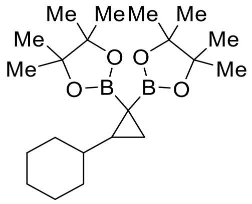

3e

According to **General procedure 1** with vinylcyclohexane (33.1 mg, 0.3 mmol, 1 equiv.), the reaction mixture was purified by column chromatography on silica gel (hexane/EtOAc = 50/1) to yield the product **3e** as a colorless oil (77.9 mg, 69% yield).

^{1}H NMR (400 MHz, $CDCl_3$): δ 1.99 – 1.89 (m, 1H), 1.83 – 1.75 (m, 1H), 1.74 – 1.67 (m, 2H), 1.64 – 1.58 (m, 1H), 1.30 – 1.27 (m, 2H), 1.26 (s, 6H), 1.24 (s, 6H), 1.20 (s, 6H), 1.18 (s, 6H), 1.13 – 1.06 (m, 3H), 1.05 – 0.97 (m, 1H), 0.90 (dd, J = 7, 3 Hz, 1H), 0.70 (dd, J = 5, 3 Hz, 2H). **^{13}C NMR** (101 MHz, $CDCl_3$): δ 82.8, 82.5, 41.8, 33.7, 33.3, 31, 26.6, 26.4, 26.1, 25.1, 24.8, 24.5, 24.3, 14.8. **^{11}B NMR** (128 MHz, $CDCl_3$): δ 34.2. **HRMS-ESI** (m/z): Calculated (found) for $C_{21}H_{39}B_2O_4$ $[M+H]^+$ 377.3029 (377.3023). **IR** (film): 2979, 2929, 2854, 1380, 1371, 1320, 1269, 1166, 1138, 970, 909, 850, 734 cm^{-1}.

2,2'-(2-(tert-butyl)cyclopropane-1,1-diyl)bis(4,4,5,5-tetramethyl-1,3,2-dioxaborolane) (3f)

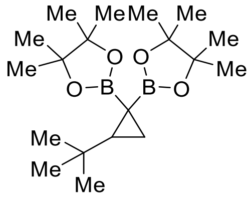

3f

According to **General procedure 1** with 3,3-dimethylbut-1-ene (25.3 mg, 0.3 mmol, 1 equiv.), the reaction mixture was purified by column chromatography on silica gel (hexane/EtOAc = 50/1) to yield the product **3f** as a colorless oil (70.4 mg, 67% yield).

^{1}H NMR (400 MHz, $CDCl_3$): δ 1.29 (s, 6H), 1.27 (s, 6H), 1.20 (s, 6H), 1.19 (s, 6H), 0.91 (s, 9H), 0.89 – 0.86 (m, 1H), 0.79 – 0.73 (m, 2H). **^{13}C NMR** (101 MHz, $CDCl_3$): δ 82.95, 82.67, 36.1, 30.9, 28.8, 24.95, 24.89, 24.6, 24.5, 10.7. **^{11}B NMR** (128 MHz, $CDCl_3$): δ 31.9. **HRMS-ESI** (m/z): Calculated (found) for $C_{19}H_{37}B_2O_4$ $[M+H]^+$ 351.2872 (351.2866). **IR** (film): 2972, 2850, 1378, 1316, 1270, 970, 850 cm^{-1}.

2,2'-(2-(4-methoxybenzyl)cyclopropane-1,1-diyl)bis(4,4,5,5-tetramethyl-1,3,2-dioxaborolane) (3g)

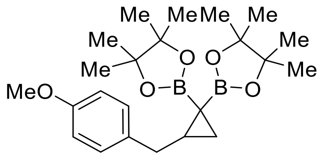

3g

According to **General procedure 1** with 1-allyl-4-methoxybenzene (44.5 mg, 0.3 mmol, 1 equiv.), the reaction mixture was purified by column chromatography on silica gel (hexane/EtOAc = 30/1) to yield the product **3g** as a white solid (91.9 mg, 74% yield).

^{1}H NMR (400 MHz, $CDCl_3$): δ 7.24 – 7.15 (m, 2H), 6.85 – 6.80 (m, 2H), 3.78 (s, 3H), 3.01 (dd, J = 15, 4 Hz, 1H), 2.19 (dd, J = 15, 10 Hz, 1H), 1.45 – 1.38 (m, 1H), 1.24 (s, 6H), 1.22 (s, 6H), 1.18 (s, 6H), 1.16 (s, 6H), 1.03 (dd, J = 7, 3 Hz, 1H), 0.84 (dd, J = 5, 3 Hz, 1H). **^{13}C NMR** (101 MHz, $CDCl_3$): δ 157.7, 134.4, 129.2, 113.6, 83, 82.6, 55.2, 37.9, 25.1, 24.7, 24.5, 24.3, 16.2. **^{11}B NMR** (128 MHz, $CDCl_3$): δ 33.4. **HRMS-ESI** (m/z): Calculated (found) for $C_{23}H_{37}B_2O_5$ $[M+H]^+$ 415.2822 (415.2814). **IR** (film): 3055, 2982, 1636, 1422, 1381, 1317, 1266, 1189, 1139, 896, 849, 738, 705, 665 cm^{-1}.

2,2'-(2-(3-phenoxypropyl)cyclopropane-1,1-diyl)bis(4,4,5,5-tetramethyl-1,3,2-dioxaborolane) (3h)

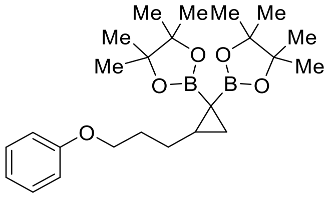

3h

According to **General procedure 1** with (pent-4-en-1-yloxy)benzene (48.7 mg, 0.3 mmol, 1 equiv.), the reaction mixture was purified by column chromatography on silica gel (hexane/EtOAc = 30/1) to yield the product **3h** as a colorless oil (106.6 mg, 83% yield).

^{1}H NMR (400 MHz, $CDCl_3$): δ 7.29 (t, J = 8 Hz, 1H), 6.97 – 6.88 (m, 3H), 4.00 (t, J = 7 Hz, 2H), 2.04 – 1.87 (m, 2H), 1.72 – 1.63 (m, 1H), 1.49 – 1.33 (m, 2H), 1.27 (s, 6H), 1.25 (s, 6H), 1.21 (s, 6H), 1.20 (s, 6H), 0.98 (dd, J = 7, 3 Hz, 1H), 0.70 (dd, J = 5, 3 Hz, 1H). **^{13}C NMR** (101 MHz, $CDCl_3$): δ 159.1, 129.3, 120.3, 114.4, 82.9, 82.6, 67.5, 29.47, 29.45, 25.1, 24.8, 24.7, 24.6, 24.3, 23.1, 15.8. **^{11}B NMR** (128 MHz, $CDCl_3$): δ 32.9. **HRMS-ESI** (m/z): Calculated (found) for $C_{24}H_{39}B_2O_5$ $[M+H]^+$ 429.2978 (429.2971). **IR** (film): 3447, 3054, 2985, 1636, 1422, 1317, 1265, 1140, 896, 741, 705 cm^{-1}.

2,2'-(2-((benzyloxy)methyl)cyclopropane-1,1-diyl)bis(4,4,5,5-tetramethyl-1,3,2-dioxaborolane) (3i)

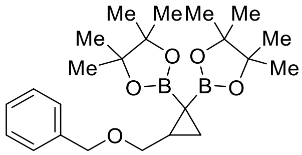

3i

According to **General procedure 1** with ((allyloxy)methyl)benzene (44.5 mg, 0.3 mmol, 1 equiv.), the reaction mixture was purified by column chromatography on silica gel (hexane/EtOAc = 30/1) to yield the product **3i** as a white solid (95.7 mg, 77% yield).

^{1}H NMR (400 MHz, $CDCl_3$): δ 7.39 – 7.31 (m, 4H), 7.30 – 7.24 (m, 1H), 4.55 (s, 2H), 3.73 (dd, J = 10, 5 Hz, 1H), 3.20 (dd, J = 10, 8 Hz, 1H), 1.63 – 1.53 (m, 1H), 1.22 (s, 6H), 1.20 (s, 6H), 1.19 (s, 6H), 1.18 (s, 6H), 1.05 (dd, J = 7, 3 Hz, 1H), 0.84 (dd, J = 5, 3 Hz, 1H). **^{13}C NMR** (101 MHz, $CDCl_3$): δ 138.6, 128.3, 127.8, 127.4, 83.01, 82.81, 72.7, 72.5, 24.9, 24.8, 24.7, 24.6, 24.4, 22.0, 14.6. **^{11}B NMR** (128 MHz, $CDCl_3$): δ 33.8. **HRMS-ESI** (m/z): Calculated (found) for $C_{23}H_{37}B_2O_5$ $[M+H]^+$ 415.2822 (415.2815). **IR** (film): 3056, 2981, 1634, 1412, 1320, 1268, 1140, 894, 721 cm^{-1}.

2,2'-(2-(4-(4-fluorophenoxy)butyl)cyclopropane-1,1-diyl)bis(4,4,5,5-tetramethyl-1,3,2-dioxaborolane) (3j)

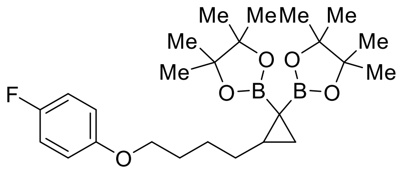

3j

According to **General procedure 1** with 1-fluoro-4-(hex-5-en-1-yloxy)benzene (58.3 mg, 0.3 mmol, 1 equiv.), the reaction mixture was purified by column chromatography on silica gel (hexane/EtOAc = 30/1) to yield the product **3j** as a colorless oil (103.5 mg, 75% yield).

^{1}H NMR (400 MHz, $CDCl_3$): δ 6.97 – 6.92 (m, 2H), 6.82 – 6.79 (m, 2H), 3.89 (t, J = 7 Hz, 2H), 1.82 – 1.75 (m, 2H), 1.60 – 1.56 (m, 2H), 1.23 (s, 6H) 1.22 – 1.20 (m, 3H), 1.20 (s, 6H), 1.17 (s, 6H), 1.16 (s, 6H), 0.94 (dd, J = 7, 3 Hz, 1H), 0.69 – 0.60 (m, 1H). **^{13}C NMR** (101 MHz, $CDCl_3$): δ 157 (d, J = 238 Hz), 155.2, 115.7 (d, J = 23 Hz), 115.4 (d, J = 8 Hz), 82.9, 82.6, 68.6, 32.8, 29.2, 26.3, 25.1, 24.8, 24.6, 24.3, 23.4, 16. **^{19}F NMR** (376 MHz, $CDCl_3$) δ -124.6. **^{11}B NMR** (128 MHz, $CDCl_3$): δ 31.8. **HRMS-ESI** (m/z): Calculated (found) for $C_{25}H_{40}B_2FO_5$ $[M+H]^+$ 461.3040 (461.3036). **IR** (film): 2980, 2932, 1507, 1471, 1445, 1380, 1372, 1316, 1266, 1249, 1213, 1167, 1139, 1097, 969, 910, 849, 829, 734, 649, 514 cm^{-1}.

<u>2,2'-(2-(3-chloropropyl)cyclopropane-1,1-diyl)bis(4,4,5,5-tetramethyl-1,3,2-dioxaborolane) (3k)</u>

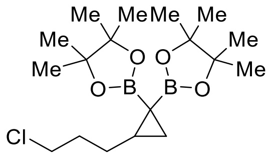

3k

According to **General procedure 1** with 5-chloropent-1-ene (31.4 mg, 0.3 mmol, 1 equiv.), the reaction mixture was purified by column chromatography on silica gel (hexane/EtOAc = 50/1) to yield the product **3k** as a colorless oil (84.5 mg, 76% yield).

^{1}H NMR (400 MHz, $CDCl_3$): δ 3.58 (t, J = 7 Hz, 2H), 2.01 – 1.86 (m, 2H), 1.69 – 1.57 (m, 1H), 1.44 – 1.27 (m, 2H), 1.26 (s, 6H), 1.24 (s, 6H), 1.20 (s, 6H), 1.19 (s, 6H), 0.96 (dd, J = 7, 3 Hz, 1H), 0.68 (dd, J = 5, 3 Hz, 1H). **^{13}C NMR** (101 MHz, $CDCl_3$): δ 83.0, 82.7, 45.0, 32.8, 30.3, 25.1, 24.8, 24.6, 24.3, 22.5, 15.8. **^{11}B NMR** (128 MHz, $CDCl_3$): δ 31.7. **HRMS-ESI** (m/z): Calculated (found) for $C_{18}H_{34}B_2ClO_4$ $[M+H]^+$ 371.2326 (371.2321). **IR** (film): 2978, 2931, 1444, 1371, 1316, 1269, 1214, 1166, 1139, 969, 849 cm^{-1}.

<u>2,2'-(2-(4-(4-bromophenoxy)butyl)cyclopropane-1,1-diyl)bis(4,4,5,5-tetramethyl-1,3,2-dioxaborolane) (3l)</u>

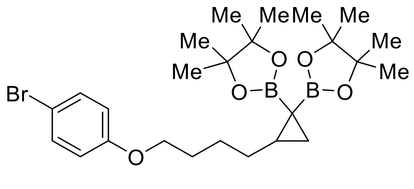

3l

According to **General procedure 1** with 1-bromo-4-(hex-5-en-1-yloxy)benzene (76.6 mg, 0.3 mmol, 1 equiv.), the reaction mixture was purified by column chromatography on silica gel (hexane/EtOAc = 30/1) to yield the product **3l** as a colorless oil (121.9 mg, 78% yield).

^{1}H NMR (400 MHz, $CDCl_3$): δ 7.36 (d, J = 9 Hz, 2H), 6.77 (d, J = 9 Hz, 2H), 3.91 (t, J = 7 Hz, 2H), 1.83 – 1.77 (m, 2H), 1.62 – 1.58 (m, 2H), 1.28 (t, J = 6 Hz, 1H), 1.27 – 1.24 (m, 2H), 1.25 (s, 6H), 1.22 (s, 6H), 1.20 (s, 6H), 1.18 (s, 6H), 0.98 – 0.94 (m, 1H), 0.67 (dd, J = 5, 3 Hz, 1H). **^{13}C NMR** (101 MHz, $CDCl_3$): δ 158.2, 132.2, 116.3, 112.5, 82.9, 82.6, 68.3, 32.8, 29, 26.2, 25.1, 24.8, 24.6, 24.3, 23.4, 16. **^{11}B NMR** (128 MHz, $CDCl_3$): δ 33.5. **HRMS-ESI** (m/z): Calculated (found) for $C_{25}H_{40}B_2BrO_5$ $[M+H]^+$ 521.2240 (521.2237). **IR** (film): 2977, 1596, 1505, 1378, 1372, 1327, 1265, 1229, 1137, 968, 845, 788, 671, 579 cm^{-1}.

2,2'-(2-(4-(4-iodophenoxy)butyl)cyclopropane-1,1-diyl)bis(4,4,5,5-tetramethyl-1,3,2-dioxaborolane) (3m)

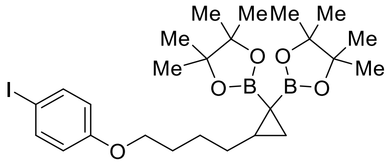

3m

According to **General procedure 1** with 1-(hex-5-en-1-yloxy)-4-iodobenzene (90.7 mg, 0.3 mmol, 1 equiv.), the reaction mixture was purified by column chromatography on silica gel (hexane/EtOAc = 30/1) to yield the product **3m** as a white solid (121 mg, 71% yield).

^{1}H NMR (400 MHz, $CDCl_3$): δ 7.55 (d, J = 9 Hz, 2H), 6.68 (d, J = 9 Hz, 2H), 3.92 (t, J = 7 Hz, 2H), 1.84 – 1.78 (m, 2H), 1.62 – 1.58 (m, 2H), 1.32 – 1.27 (m, 3H), 1.26 (s, 6H), 1.23 (s, 6H), 1.20 (s, 6H), 1.19 (s, 6H), 0.96 (dd, J = 7, 3 Hz, 1H), 0.67 (dd, J = 5, 3 Hz, 1H). **^{13}C NMR** (101 MHz, $CDCl_3$): δ 159, 138.1, 116.9, 82.9, 82.6, 68.1, 32.8, 29, 26.2, 25.1, 24.8, 24.6, 24.3, 23.4, 16. **^{11}B NMR** (128 MHz, $CDCl_3$): δ 31.5. **HRMS-ESI** (m/z): Calculated (found) for $C_{25}H_{40}B_2IO_5$ $[M+H]^+$ 569.2101 (569.2093). **IR** (film): 2981, 2252, 1587, 1487, 1471, 1380, 1372, 1315, 1243, 1175, 1139, 968, 908, 849, 822, 734, 650 cm^{-1}.

2,2'-(2-(4-(4-(trifluoromethyl)phenoxy)butyl)cyclopropane-1,1-diyl)bis(4,4,5,5-tetramethyl-1,3,2-dioxaborolane) (3n)

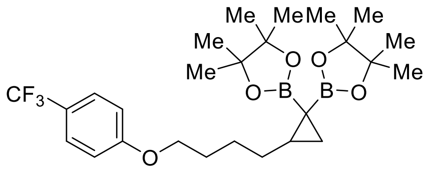

3n

According to **General procedure 1** with 1-(hex-5-en-1-yloxy)-4-(trifluoromethyl)benzene (73.3 mg, 0.3 mmol, 1 equiv.), the reaction mixture was purified by column chromatography on silica gel (hexane/EtOAc = 30/1) to yield the product **3n** as a colorless oil (113.3 mg, 74% yield).

^{1}H NMR (400 MHz, $CDCl_3$): *δ* 7.54 (d, *J* = 9 Hz, 2H), 6.95 (d, *J* = 9 Hz, 2H), 4.02 – 3.97 (m, 2H), 1.87 – 1.77 (m, 2H), 1.68 – 1.54 (m, 2H), 1.33 – 1.26 (m, 3H), 1.25 (s, 6H), 1.23 (s, 6H), 1.20 (s, 6H), 1.19 (s, 6H), 1.00 – 0.94 (m, 1H), 0.68 (dd, *J* = 5, 3 Hz, 1H). **^{13}C NMR** (101 MHz, $CDCl_3$): *δ* 161.6, 133.4, 126.8 (q, *J* = 3 Hz), 126.6 (q, *J* = 272 Hz), 114.4, 82.9, 82.6, 68.2, 32.7, 28.9, 26.2, 25.1, 24.8, 24.6, 24.3, 23.4, 16. **^{11}B NMR** (128 MHz, $CDCl_3$): *δ* 33.1. **HRMS-ESI** (m/z): Calculated (found) for $C_{26}H_{40}B_2F_3O_5$ $[M+H]^+$ 511.3008 (511.3002). **IR** (film): 2982, 1721, 1636, 1381, 1372, 1313, 1266, 1222, 1168, 1138, 1018, 969, 896, 849, 739, 705 cm^{-1}.

methyl 3-(2,2-bis(4,4,5,5-tetramethyl-1,3,2-dioxaborolan-2-yl)cyclopropyl)propanoate (3o)

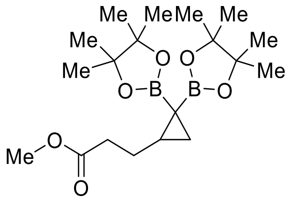

3o

According to **General procedure 1** with methyl pent-4-enoate (34.2 mg, 0.3 mmol, 1 equiv.), the reaction mixture was purified by column chromatography on silica gel (hexane/EtOAc = 20/1) to yield the product **3o** as a colorless oil (70.7 mg, 62% yield).

^{1}H NMR (400 MHz, $CDCl_3$): *δ* 3.61 (s, 3H), 1.61 – 1.47 (m, 2H), 1.45 – 1.31 (m, 2H), 1.21 (s, 24H), 1.12 – 1.03 (m, 1H), 0.85 (t, *J* = 8 Hz, 1H), 0.77 – 0.69 (m, 1H). **^{13}C NMR** (101 MHz, $CDCl_3$): *δ* 175.0, 83.1, 83.1, 51.4, 29, 25.3, 24.8, 24.6, 24.5, 20.1, 16. **^{11}B NMR** (128 MHz, $CDCl_3$): *δ* 33. **HRMS-ESI** (m/z): Calculated (found) for $C_{19}H_{35}B_2O_6$ $[M+H]^+$ 381.2614 (381.2611). **IR** (film): 2983, 2256, 1732, 1641, 1423, 1371, 1314, 1262, 1214, 1141, 906, 732, 649 cm^{-1}.

2,2'-(2-(cyclohex-3-en-1-yl)cyclopropane-1,1-diyl)bis(4,4,5,5-tetramethyl-1,3,2-dioxaborolane) (3p)

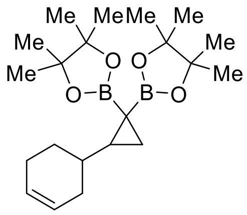

3p

According to **General procedure 1** with 4-vinylcyclohex-1-ene (32.5 mg, 0.3 mmol, 1 equiv.), the reaction mixture was purified by column chromatography on silica gel (hexane/EtOAc = 50/1) to yield the product **3p** as a colorless oil (76.3 mg, 68% yield). Diastereomeric ratio (dr) was determined through ^{13}C NMR.

^{1}H NMR (400 MHz, $CDCl_3$): δ 5.82 – 5.53 (m, 2H), 2.30 – 1.86 (m, 5H), 1.47 – 1.39 (m, 1H), 1.26 (s, 6H), 1.23 (s, 6H), 1.21 (s, 6H), 1.19 (s, 6H), 1.14 – 1.06 (m, 1H), 1.06 – 0.96 (m, 1H), 0.94 (dd, J = 7, 3 Hz, 1H), 0.78 – 0.69 (m, 1H). **^{13}C NMR** (101 MHz, $CDCl_3$): δ 126.9, 126.7, 126.6, 126.5, 82.9, 82.8, 82.6, 37.9, 37.7, 32.1, 31.9, 30.2, 30, 29.3, 28.8, 25, 24.8, 24.5, 24.4, 24.2, 14.8, 14.6. **^{11}B NMR** (128 MHz, $CDCl_3$): δ 33.5. **HRMS-ESI** (m/z): Calculated (found) for $C_{21}H_{37}B_2O_4$ $[M+H]^+$ 375.2872 (375.2865). **IR** (film): 3056, 2972, 1702, 1420, 1266, 1138, 894, 731, 705 cm^{-1}.

<u>(2-(2,2-bis(4,4,5,5-tetramethyl-1,3,2-dioxaborolan-2-yl)cyclopropyl)ethoxy)dimethyl(phenyl)silane (3q)</u>

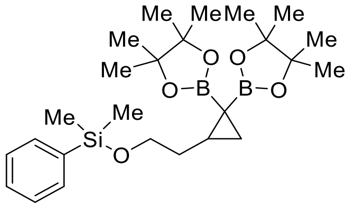

3q

According to **General procedure 1** with (but-3-en-1-yloxy)dimethyl(phenyl)silane (61.9 mg, 0.3 mmol, 1 equiv.), the reaction mixture was purified by column chromatography on silica gel (hexane/EtOAc = 40/1) to yield the product **3q** as a colorless oil (106.3 mg, 75% yield).

^{1}H NMR (400 MHz, $CDCl_3$): δ 7.65 – 7.55 (m, 2H), 7.46 – 7.31 (m, 3H), 3.85 – 3.58 (m, 2H), 1.88 – 1.78 (m, 1H), 1.45 – 1.37 (m, 1H), 1.33 – 1.27 (m, 1H), 1.24 (s, 6H), 1.22 (s, 6H), 1.20 (s, 6H), 1.18 (s, 6H), 0.92 (dd, J = 7, 3 Hz, 1H), 0.65 (dd, J = 5, 3 Hz, 1H), 0.39 (s, 6H). **^{13}C NMR** (101 MHz, $CDCl_3$): δ 138.2, 133.5, 129.4, 127.8, 82.9, 82.6, 63.3, 36, 25.1, 24.8, 24.7, 24.6, 24.4, 20, 15.5, -1.57, -1.64. **^{11}B NMR** (128 MHz, $CDCl_3$): δ 33.5. **HRMS-ESI** (m/z): Calculated (found) for $C_{25}H_{43}B_2O_5Si$ $[M+H]^+$ 473.3060 (473.3054). **IR** (film): 3056, 1703, 1264, 896, 739, 705 cm^{-1}.

2,2'-(2-(naphthalen-2-ylmethyl)cyclopropane-1,1-diyl)bis(4,4,5,5-tetramethyl-1,3,2-dioxaborolane) (3r)

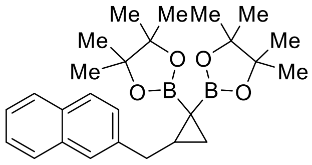

3r

According to **General procedure 1** with 2-allylnaphthalene (50.5 mg, 0.3 mmol, 1 equiv.), the reaction mixture was purified by column chromatography on silica gel (hexane/EtOAc = 50/1) to yield the product **3r** as a colorless oil (91.2 mg, 70% yield).

^{1}H NMR (400 MHz, $CDCl_3$): δ 8.09 (d, J = 8 Hz, 1H), 7.87 (d, J = 8 Hz, 1H), 7.73 (d, J = 8 Hz, 1H), 7.57 (d, J = 7 Hz, 1H), 7.53 – 7.45 (m, 3H), 3.62 (dd, J = 15, 4 Hz, 1H), 2.78 – 2.68 (m, 1H), 1.33 – 1.28 (m, 1H), 1.26 (s, 6H), 1.24 (s, 6H), 1.23 (s, 6H), 1.22 (s, 6H), 1.11 (dd, J = 7, 3 Hz, 1H), 0.96 (dd, J = 5, 3 Hz, 1H). **^{13}C NMR** (101 MHz, $CDCl_3$): δ 138.2, 133.7, 132.1, 128.6, 126.4, 125.68, 125.65, 125.4, 125.3, 123.8, 83.1, 82.8, 35.2, 25.1, 24.8, 24.6, 24.4, 23.1, 16.8. **^{11}B NMR** (128 MHz, $CDCl_3$): δ 33.4. **HRMS-ESI** (m/z): Calculated (found) for $C_{26}H_{37}B_2O_4$ $[M+H]^+$ 435.2872 (435.2865). **IR** (film): 3447, 2977, 1379, 1370, 1314, 1138, 849, 798, 790, 777, 671, 579, 433, 422 cm^{-1}.

9-(3-(2,2-bis(4,4,5,5-tetramethyl-1,3,2-dioxaborolan-2-yl)cyclopropyl)propyl)-9*H*-carbazole (3s)

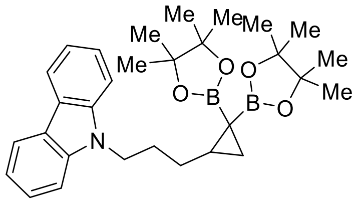

3s

According to **General procedure 1** with 9-(pent-4-en-1-yl)-9*H*-carbazole (70.6 mg, 0.3 mmol, 1 equiv.), the reaction mixture was purified by column chromatography on silica gel (hexane/EtOAc = 20/1) to yield the product **3s** as a white solid (94.7 mg, 63% yield).

^{1}H NMR (400 MHz, $CDCl_3$): δ 8.13 (t, J = 8 Hz, 2H), 7.47 (q, J = 8 Hz, 4H), 7.28 – 7.21 (m, 2H), 4.46 – 4.25 (m, 2H), 2.12 – 2.01 (m, 1H), 1.67 – 1.54 (m, 1H), 1.50 – 1.32 (m, 3H), 1.21 (s, 6H), 1.20 (s, 6H), 1.16 (s, 6H), 1.11 (s, 6H), 1.01 – 0.94 (m, 1H), 0.67 – 0.59 (m, 1H). **^{13}C NMR** (101 MHz, $CDCl_3$): δ 140.4, 125.5, 122.8, 120.3, 118.6, 108.8, 82.9, 82.7, 42.9, 30.7, 29.3, 25, 24.8, 24.6, 24.2, 23.1, 15.7. **^{11}B NMR** (128 MHz, $CDCl_3$): δ 31.9. **HRMS-ESI** (m/z): Calculated (found) for $C_{30}H_{42}B_2NO_4$ $[M+H]^+$ 502.3294 (502.3289). **IR** (film): 2978, 1725, 1703, 1607, 1518, 1469, 1371, 1309, 1242, 1200, 1139, 1083, 968, 849, 737, 703 cm^{-1}.

<u>1-(3-(2,2-bis(4,4,5,5-tetramethyl-1,3,2-dioxaborolan-2-yl)cyclopropyl)propyl)-6-chloro-1*H*-indole (3t)</u>

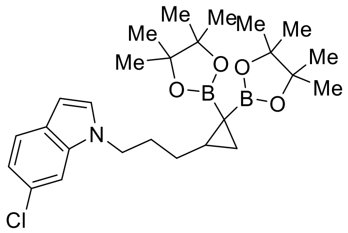

3t

According to **General procedure 1** with 6-chloro-1-(pent-4-en-1-yl)-1H-indole (65.9 mg, 0.3 mmol, 1 equiv.), the reaction mixture was purified by column chromatography on silica gel (hexane/EtOAc = 20/1) to yield the product **3t** as a colorless oil (84.5 mg, 58% yield).

^{1}**H NMR** (400 MHz, $CDCl_3$): δ 7.52 (d, J = 8 Hz, 1H), 7.35 (s, 1H), 7.12 – 7.02 (m, 2H), 6.46 (d, J = 3 Hz, 1H), 4.18 – 4.04 (m, 2H), 2.06 – 1.92 (m, 2H), 1.61 – 1.47 (m, 1H), 1.25 – 1.23 (m, 2H), 1.20 (s, 12H), 1.19 (s, 6H), 1.16 (s, 6H), 0.97 (dd, J = 7, 3 Hz, 1H), 0.64 (dd, J = 5, 3 Hz, 1H). ^{13}**C NMR** (101 MHz, $CDCl_3$): δ 136.4, 128.4, 127.3, 127, 121.7, 119.8, 109.4, 101.2, 83, 82.7, 46.3, 30.5, 30.4, 25.1, 24.8, 24.6, 24.3, 22.8, 15.7. ^{11}**B NMR** (128 MHz, $CDCl_3$): δ 33.3. **HRMS-ESI** (m/z): Calculated (found) for $C_{26}H_{39}B_2ClNO_4$ $[M+H]^+$ 486.2748 (486.2742). **IR** (film): 3056, 2982, 2302, 1421, 1368, 1312, 1261, 1142, 895, 739, 704 cm^{-1}.

Some unsuccessful examples

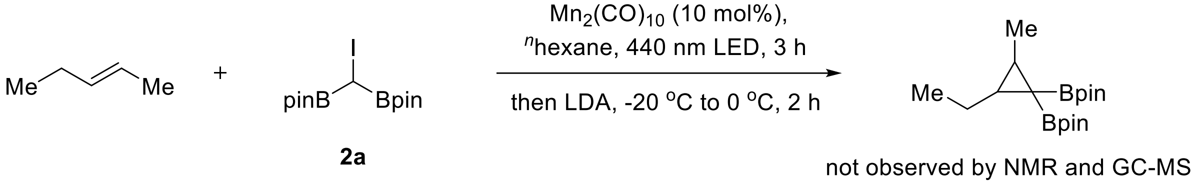

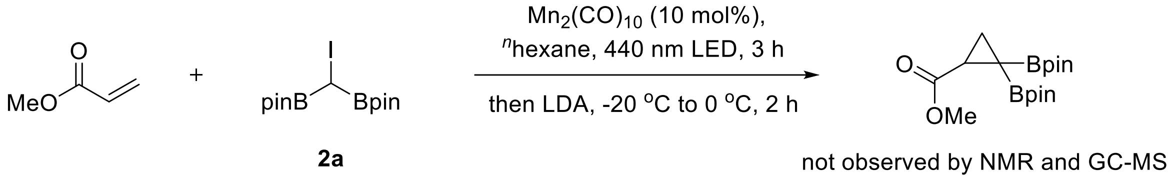

In an argon-filled glovebox, $CHI(Bpin)_2$ **2a** (0.36 mmol, 1.2 equiv.), $Mn_2(CO)_{10}$ (10 mol%), ^{n}hexane (1 mL) and acyclic internal alkenes or methyl acrylates (0.3 mmol, 1 equiv.) were sequentially added to 5 mL vial equipped with a magnetic stirring bar. The reaction tube was then sealed with a rubber cap and removed from the glovebox. It was placed 3 cm in front of a Kessil Lamp with 1 fan for cooling. The reaction tube was irradiated for 3 h. After irradiation, the reaction mixture was cooled to -20 °C, and LDA (2.5 M, 1.2 equiv. in THF) was added, followed by stirring for 2 h at 0 °C. The mixture was monitored by NMR and GC-MS analysis, and no adducts are detected.

Reactivity of chloro and bromine *gem*-diboronates

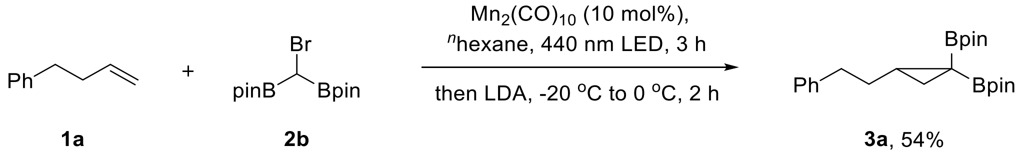

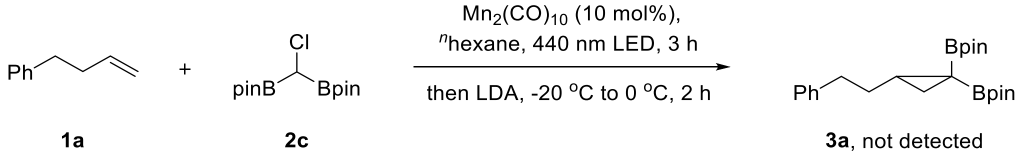

In an argon-filled glovebox, bromo *gem*-diboronate **2b** or chloro *gem*-diboronate **2c** (0.36 mmol, 1.2 equiv.), $Mn_2(CO)_{10}$ (10 mol%), ^{n}hexane (1 mL) and alkenes (0.3 mmol, 1 equiv.) were sequentially added to 5 mL vial equipped with a magnetic stirring bar. The reaction tube was then sealed with a rubber cap and removed from the glovebox. It was placed 3 cm in front of a Kessil Lamp with 1 fan for cooling. The reaction tube was irradiated for 3 h. After irradiation, the reaction mixture was cooled to -20 °C, and LDA (2.5 M, 1.2 equiv. in THF) was added, followed by stirring for 2 h at 0 °C. The reaction was monitored by TLC and GC-MS analysis. The bromine *gem*-diboronate could undergo borylcyclopropanation with

alkene **1a,** providing the target product with 54% yield. However, the chlorinated reagents **2c** proved ineffective in this system, failing to produce the desired product.

(±)-2,2'-(2-(3-(((2S,5R)-2-isopropyl-5-methylcyclohexyl)oxy)propyl)cyclopropane-1,1-diyl)bis(4,4,5,5-tetramethyl-1,3,2-dioxaborolane) (3u)

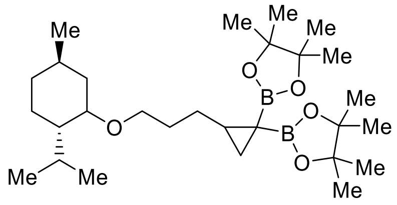

3u

According to **General procedure 1** with (1S,4R)-1-isopropyl-4-methyl-2-(pent-4-en-1-yloxy)cyclohexane (67.3 mg, 0.3 mmol, 1 equiv.), the reaction mixture was purified by column chromatography on silica gel (hexane/EtOAc = 30/1) to yield the product **3u** as a colorless oil (114.7 mg, 78% yield, 1:1 d.r.).

^{1}H NMR (400 MHz, $CDCl_3$): δ 3.66 – 3.52 (m, 1H), 3.32 – 3.19 (m, 1H), 3.03 – 2.88 (m, 1H), 2.32 – 2.14 (m, 2H), 2.12 – 1.99 (m, 1H), 1.94 – 1.82 (m, 1H), 1.75 – 1.48 (m, 6H), 1.39 – 1.29 (m, 1H), 1.22 (s, 6H), 1.20 (s, 6H), 1.16 (s, 6H), 1.15 (s, 6H), 0.97 – 0.81 (m, 10H), 0.78 – 0.71 (m, 3H), 0.67 – 0.58 (m, 1H). **^{13}C NMR** (101 MHz, $CDCl_3$): δ 82.9, 82.6, 78.9, 78.8, 68.4, 68.2, 48.3, 40.5, 34.7, 31.6, 30.6, 29.82, 29.76, 25.5, 25.1, 24.8, 24.64, 24.62, 24.6, 24.5, 24.3, 23.5, 23.3, 22.4, 21.3, 21.0, 16.23, 16.20, 16, 15.9. **^{11}B NMR** (128 MHz, $CDCl_3$): δ 33.3. **HRMS-ESI** (m/z): Calculated (found) for $C_{28}H_{53}B_2O_5$ $[M+H]^+$ 491.4074 (491.4069). **IR** (film): 2981, 1769, 1373, 1324, 1266, 1247, 1169, 1138, 968, 848, 738, 704 cm^{-1}.

(±)-2,2'-(2-(3-(((2aR,4R,5'R,6aR,6bR,8aR,8bS,9R,10S,11aR,12aR,12bS)-5',6a,8a,9-tetramethyldocosahydrospiro[naphtho[2',1':4,5]indeno[2,1-b]furan-10,2'-pyran]-4-yl)oxy)propyl)cyclopropane-1,1-diyl)bis(4,4,5,5-tetramethyl-1,3,2-dioxaborolane) (3v)

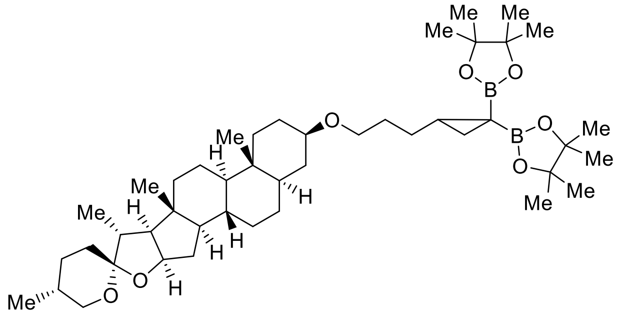

3v

According to **General procedure 1** with (2aR,4R,5'R,6aR,6bR,8aR,8bS,9R,10S,11aR,12aR,12bS)-5',6a,8a,9-tetramethyl-4-(pent-4-en-1-yloxy)docosahydrospiro[naphtho[2',1':4,5]indeno[2,1-b]furan-10,2'-pyran] (145.4 mg, 0.3 mmol, 1 equiv.), the reaction mixture was purified by column chromatography on silica gel (hexane/EtOAc = 30/1) to yield the product **3v** as a colorless oil (141.9 mg, 63% yield, 1:1 d.r.).

^{1}H NMR (400 MHz, $CDCl_3$): *δ* 4.37 (q, *J* = 7 Hz, 1H), 3.54 – 3.30 (m, 4H), 3.24 – 3.05 (m, 1H), 1.99 – 1.93 (m, 1H), 1.91 – 1.77 (m, 3H), 1.76 – 1.72 (m, 1H), 1.70 – 1.55 (m, 10H), 1.53 – 1.43 (m, 3H), 1.43 – 1.29 (m, 3H), 1.28 – 1.24 (m, 4H), 1.22 (s, 6H), 1.19 (s, 6H), 1.16 (s, 6H), 1.14 (s, 6H), 1.12 – 0.98 (m, 4H), 0.94 (d, *J* = 7 Hz, 3H), 0.92 – 0.84 (m, 3H), 0.81 – 0.72 (m, 9H), 0.66 – 0.57 (m, 2H). **^{13}C NMR** (101 MHz, $CDCl_3$): *δ* 109.2, 82.9, 82.6, 80.9, 78.3, 67.6, 66.8, 62.2, 56.3, 54.4, 44.9, 41.6, 40.6, 40.1, 37, 35.9, 35.1, 34.9, 32.3, 31.8, 31.4, 30.4, 30.3, 29.7, 28.80, 28.76, 28.3, 25.1, 24.8, 24.7, 24.6, 24.3, 23.3, 21, 17.1, 16.5, 16, 14.5, 12.3. **^{11}B NMR** (128 MHz, $CDCl_3$): *δ* 32.5. **HRMS-ESI** (m/z): Calculated (found) for $C_{45}H_{77}B_2O_5$ $[M+H]^+$ 491.4074 (491.4069). **IR** (film): 2981, 1769, 1373, 1324, 1266, 1247, 1169, 1138, 968, 848, 738, 704 cm^{-1}.

(±)-2,2'-(2-(4-(((3aR,5R,6R,6aR)-5-((R)-2,2-dimethyl-1,3-dioxolan-4-yl)-2,2-dimethyltetrahydrofuro[2,3-d][1,3]dioxol-6-yl)oxy)butyl)cyclopropane-1,1-diyl)bis(4,4,5,5-tetramethyl-1,3,2-dioxaborolane) (3w)

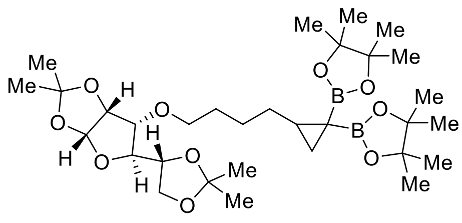

3w

According to **General procedure 1** with (3aR,5R,6R,6aR)-5-((R)-2,2-dimethyl-1,3-dioxolan-4-yl)-6-(hex-5-en-1-yloxy)-2,2-dimethyltetrahydrofuro[2,3-d][1,3]dioxole (102.7 mg, 0.3 mmol, 1 equiv.), the reaction mixture was purified by column chromatography on silica gel (hexane/EtOAc = 10/1) to yield the product **3w** as a colorless oil (120.5 mg, 66% yield, 1:1 d.r.).

^{1}H NMR (400 MHz, $CDCl_3$): *δ* 5.86 (d, *J* = 4 Hz, 1H), 4.51 (s, 1H), 4.30 (q, *J* = 7 Hz, 1H), 4.15 – 4.02 (m, 2H), 3.96 (t, *J* = 7 Hz, 1H), 3.83 (d, *J* = 3 Hz, 1H), 3.63 – 3.53 (m, 1H), 3.53 – 3.42 (m, 1H), 2.29 – 1.96 (m, 1H), 1.62 – 1.51 (m, 2H), 1.48 (s, 3H), 1.41 (s, 3H), 1.34 (s, 3H), 1.31 (s, 3H), 1.24 (s, 2H) 1.22 (s, 6H), 1.20 (s, 6H), 1.19 – 1.16 (m, 2H), 1.17 (s, 6H), 1.15 (s, 6H), 0.91 (dd, *J* = 7, 3 Hz, 1H), 0.69 – 0.54 (m, 1H). **^{13}C NMR** (101 MHz, $CDCl_3$): *δ* 111.6, 108.8, 105.3, 82.9, 82.7, 82.5, 82, 81.2, 72.5, 70.7, 67.2, 32.8, 32.7, 29.7, 29.6, 29.5, 26.8, 26.8, 26.2, 25.4, 25.1, 24.9, 24.83, 24.76, 24.7, 24.54, 24.46, 24.3, 23.4, 16. **^{11}B NMR** (128 MHz, $CDCl_3$): *δ* 32.6. **HRMS-ESI** (m/z): Calculated (found) for $C_{31}H_{55}B_2O_{10}$

[M+H]$^+$ 595.3819 (595.3814). **IR** (film): 2982, 1654, 1373, 1317, 1266, 1215, 1166, 1138, 1080, 1019, 968, 849, 735, 705 cm^{-1}.

(±)-2,2'-(2-(3-(((3S,5S,8R,9S,10S,13S,14S)-10,13-dimethylhexadecahydrospiro[cyclopenta[a]phenanthrene-17,2'-[1,3]dioxolan]-3-yl)oxy)propyl)cyclopropane-1,1-diyl)bis(4,4,5,5-tetramethyl-1,3,2-dioxaborolane) (3x)

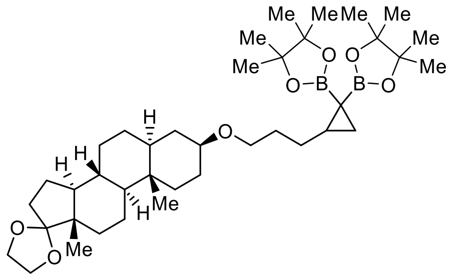

3x

According to **General procedure 1** with (3S,5S,8R,9S,10S,13S,14S)-10,13-dimethyl-3-(pent-4-en-1-yloxy)hexadecahydrospiro[cyclopenta[a]phenanthrene-17,2'-[1,3]dioxolane] (120.8 mg, 0.3 mmol, 1 equiv.), the reaction mixture was purified by column chromatography on silica gel (hexane/EtOAc = 10/1) to yield the product **3x** as a colorless oil (98.3 mg, 49% yield, 1:1 d.r.).

^{1}H NMR (400 MHz, $CDCl_3$): δ 3.94 – 3.87 (m, 2H), 3.86 – 3.82 (m, 2H), 3.44 (td, *J* = 7, 3Hz, 2H), 3.16 (tt, *J* = 11, 5 Hz, 1H), 1.99 – 1.93 (m, 1H), 1.82 – 1.48 (m, 12H), 1.42 – 1.31 (m, 5H), 1.28 – 1.24 (m, 4H), 1.22 (s, 6H), 1.20 (s, 6H), 1.23 – 1.18 (m, 1H), 1.17 (s, 12H), 1.15 (m, 1H), 0.92 (m, 2H), 0.89 – 0.85 (m, 1H), 0.82 (s, 3H), 0.78 (s, 3H), 0.71 – 0.60 (m, 2H). **^{13}C NMR** (101 MHz, $CDCl_3$): δ 119.5, 82.9, 82.5, 78.2, 67.6, 65.2, 64.5, 54.2, 50.7, 46, 44.9, 37.1, 35.8, 35.7, 34.9, 34.2, 31.4, 30.7, 30.3, 29.6, 28.7, 28.3, 25.1, 24.8, 24.7, 24.5, 24.3, 23.3, 22.6, 20.6, 16, 14.4, 12.3. **^{11}B NMR** (128 MHz, $CDCl_3$): δ 33.2. **HRMS-ESI** (m/z): Calculated (found) for $C_{39}H_{67}B_2O_7$ [M+H]$^+$ 669.5067 (669.5064). **IR** (film): 2980, 1652, 1317, 1267, 1215, 1017, 968, 849, 705 cm^{-1}.

(±)-2,2'-(2-(4-(((R)-2,5,6,8-tetramethyl-2-((4R,8R)-4,8,12-trimethyltridecyl)chroman-7-yl)oxy)butyl)cyclopropane-1,1-diyl)bis(4,4,5,5-tetramethyl-1,3,2-dioxaborolane) (3y)

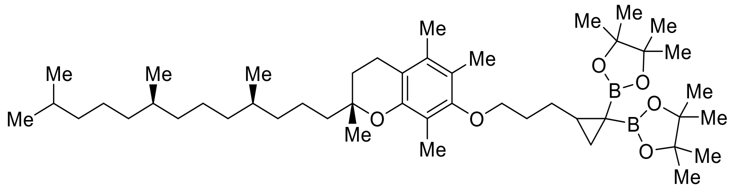

3y

According to **General procedure 1** with (R)-7-(hex-5-en-1-yloxy)-2,5,6,8-tetramethyl-2-((4R,8R)-4,8,12-trimethyltridecyl)chromane (153.9 mg, 0.3 mmol, 1 equiv.), the reaction mixture was purified by

column chromatography on silica gel (hexane/EtOAc = 10/1) to yield the product **3y** as a colorless oil (165.9 mg, 71% yield, 1:1 d.r.).

^{1}H NMR (400 MHz, $CDCl_3$): δ 3.63 (t, J = 7 Hz, 2H), 2.56 (t, J = 7 Hz, 2H), 2.14 (s, 3H), 2.10 (s, 3H), 2.07 (s, 3H), 1.99 – 1.86 (m, 2H), 1.81 – 1.69 (m, 4H), 1.59 – 1.47 (m, 4H), 1.44 – 1.26 (m, 11H), 1.24 (s, 6H), 1.22 (s, 6H), 1.19 (s, 6H), 1.17 (s, 6H), 1.16 – 1.01 (m, 8H), 0.99 (s, 1H), 0.90 – 0.80 (m, 14H), 0.68 (s, 1H). **^{13}C NMR** (101 MHz, $CDCl_3$): δ 148.4, 147.6, 127.9, 125.9, 122.7, 117.4, 82.9, 82.6, 74.7, 72.9, 40.1, 39.4, 37.5, 37.5, 37.4, 37.3, 32.8, 32.7, 31.3, 30.6, 30.0, 28.0, 25.1, 24.8, 24.6, 24.5, 24.4, 23.9, 23.5, 22.74, 22.65, 21.1, 20.7, 19.8, 19.7, 16.0, 12.7, 11.9, 11.8. **^{11}B NMR** (128 MHz, $CDCl_3$): δ 32.7. **HRMS-ESI** (m/z): Calculated (found) for $C_{48}H_{85}B_2O_6$ $[M+H]^+$ 779.6527 (779.6539). **IR** (film): 2929, 1460, 1380, 1319, 1265, 1249, 1139, 1088, 969, 849, 741 cm^{-1}.

(±)-2,2'-(2-(3-(((4R,5'S,6aS,6bR,8aR,8bS,9R,10S,11aR,12aR,12bR)-5',6a,8a,9-tetramethyl-1,3,3',4,4',5,5',6,6a,6b,6',7,8,8a,8b,9,11a,12,12a,12b-icosahydrospiro[naphtho[2',1':4,5]indeno[2,1-b]furan-10,2'-pyran]-4-yl)oxy)propyl)cyclopropane-1,1-diyl)bis(4,4,5,5-tetramethyl-1,3,2-dioxaborolane) (3z)

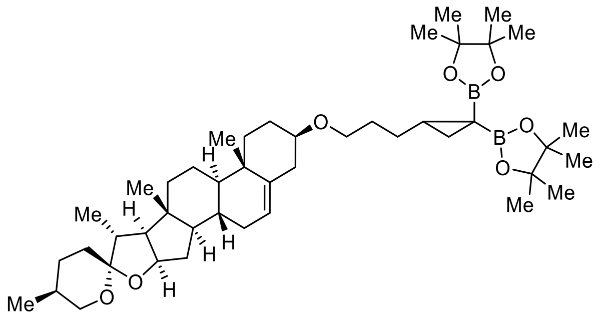

3z

According to **General procedure 1** with (4R,5'S,6aS,6bR,8aR,8bS,9R,10S,11aR,12aR,12bR)-5',6a,8a,9-tetramethyl-4-(pent-4-en-1-yloxy)-1,3,3',4,4',5,5',6,6a,6b,6',7,8,8a,8b,9,11a,12,12a,12b-icosahydrospiro[naphtho[2',1':4,5]indeno[2,1-b]furan-10,2'-pyran] (144.8 mg, 0.3 mmol, 1 equiv.), the reaction mixture was purified by column chromatography on silica gel (hexane/EtOAc = 10/1) to yield the product **3z** as a colorless oil (114.6 mg, 51% yield, 1:1 d.r.).

^{1}H NMR (400 MHz, $CDCl_3$): δ 5.35 (d, J = 6 Hz, 1H), 4.42 (q, J = 7 Hz, 1H), 4.28 – 4.16 (m, 1H), 3.55 – 3.42 (m, 3H), 3.39 (t, J = 11 Hz, 1H), 3.12 (m, 1H), 2.37 (dd, J = 13, 3 Hz, 1H), 2.22 – 2.14 (m, 1H), 2.10 – 1.94 (m, 4H), 1.92 – 1.83 (m, 5H), 1.81 – 1.76 (m, 2H), 1.70 – 1.58 (m, 7H), 1.57 – 1.37 (m, 6H), 1.29 – 1.27 (m, 2H), 1.26 (s, 6H), 1.24 (s, 6H), 1.23 (s, 6H), 1.22 (s, 6H), 1.15 – 1.10 (m, 2H), 1.03 (s, 3H), 0.99 (d, J = 7 Hz, 3H), 0.80 (t, J = 3 Hz, 6H). **^{13}C NMR** (101 MHz, $CDCl_3$): δ 141.1, 121.1, 109.3, 83.2, 83.1, 80.8, 78.9, 67.0, 66.8, 62.1, 56.5, 50.1, 43.8, 41.6, 40.2, 39.8, 39.1, 37.3, 37.2, 37, 36.7, 32.1, 31.8, 31.4, 31.3, 30.4, 30.3, 28.8, 28.4, 24.87, 24.85, 24.7, 24.5, 24.4, 20.8, 19.4, 17.1, 16.3, 14.5. **^{11}B**

NMR (128 MHz, $CDCl_3$): δ 32.4. **HRMS-ESI** (m/z): Calculated (found) for $C_{45}H_{75}B_2O_7$ $[M+H]^+$ 749.5693 (749.5655). **IR** (film): 2977, 2932, 2248, 1456, 1371, 1319, 1270, 1214, 1140, 1096, 1051, 1007, 969, 910, 864, 848, 734, 671, 647, 578 cm^{-1}.

2 Mechanistic Investigations

Control experiments

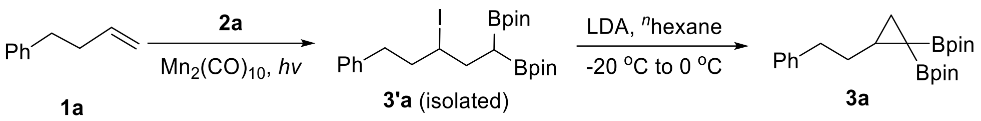

In an argon-filled glove box, a 5 mL vial equipped with a magnetic stirrer bar was charged sequentially with $CHI(Bpin)_2$ **2** (0.36 mmol, 1.2 equiv.), $Mn_2(CO)_{10}$ (10 mol%) followed by the addition of ^{n}hexane (1 mL) and alkene **1a** (0.3 mmol, 1 equiv.). The reaction mixture was stirred at 25 °C - 45 °C under 440 nm blue LED irradiation for 3 h. Then, the mixture was filtered through celite and washed with DCM. The combined organic phase was dried using Na_2SO_4 and then concentrated *in vacuo*. The crude product was purified by column chromatography on silica gel (hexane/EtOAc = 30/1) to afford the corresponding product **3′a** as a white solid (145.2 mg, 92% yield). **^{1}H NMR** (300 MHz, $CDCl_3$) δ 7.27 – 7.23 (m, 2H), 7.22 – 7.16 (m, 3H), 4.13 – 4.03 (m, 1H), 2.93 – 2.85 (m, 1H), 2.79 – 2.71 (m, 1H), 2.18 – 2.09 (m, 2H), 2.04 – 1.94 (m, 2H), 1.20 (s, 12H), 1.18 (s, 12H), 1.12 – 1.09 (m, 1H). **^{13}C NMR** (75 MHz, $CDCl_3$) δ 141.0, 128.6, 128.3, 125.9, 83.2, 83.1, 43.1, 41.9, 36.8, 35.5, 24.8, 24.7, 24.5, 24.3. **^{11}B NMR** (96 MHz, $CDCl_3$) δ 32.2. **HRMS** (ESI, m/z): calcd. for $C_{23}H_{38}B_2IO_4$ $[M+H]^+$: 527.1995, found: 527.1994. **IR** (film): 2977, 2930, 1455, 1371, 1317, 1268, 1214, 1166, 1139, 970, 850, 737, 699 cm^{-1}.

A 10-mL oven-dry Schlenk tube equipped with a magnetic stir bar was charged with **3′a** (0.2 mmol, 1 equiv.) and ^{n}hexane (20 mL) under Ar. The reaction mixture was cooled to -20 °C, and LDA (2.5 M, 1.2 equiv. in THF) was added, followed by stirring for 2 h at 0 °C. The reaction was monitored by TLC and GC-MS analysis, and **3a** was detected by GC-MS in 94% yield.

Radical trapping experiments

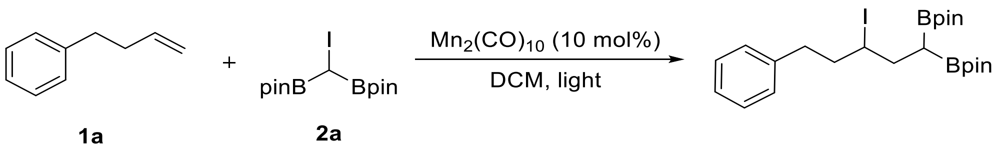

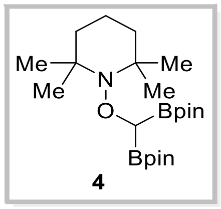

detected by HRMS

Radical trap	yield of **3a** (%)
TEMPO	N.
DHA	18%
BHT	37%

In an argon-filled glove box, $CHI(Bpin)_2$ **2a** (0.36 mmol, 1.2 equiv.) and $Mn_2(CO)_{10}$ (10 mol%) in DCM (1 mL) were added to a 10 mL thick-walled reaction tube equipped with a magnetic stirring bar. Alkene **1a** (0.3 mmol, 1 equiv.), and radical traps 2,2,6,6-tetramethylpiperidinyl-1-oxide (TEMPO, 1.5 equiv.),

9,10-dihydroanthracene (DHA, 1.5 equiv.) or butylated hydroxytoluene (BHT, 1.5 equiv.) were added. Then, the mixtures were stirred at 25 °C - 45 °C under 440 nm blue LED irradiation for 3 h, and monitored by TLC and GC-MS analysis. The reaction is effectively suppressed, and **4** was detected by HRMS. **HRMS** (ASAP, m/z): calcd. for $C_{22}H_{44}B_2NO_5$ $[M+H]^+$: 424.3400, found: 424.3397.

<u>Radical clock experiment</u>

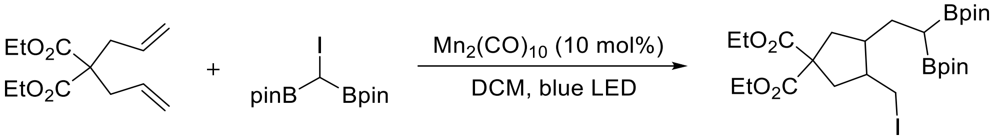

1aa **2a** **5**, 71%

In an argon-filled glove box, **2a** (0.72 mmol, 1.2 equiv.), $Mn_2(CO)_{10}$ (10 mol%), and **1aa** (0.6 mmol, 1 equiv.) in ^{n}hexane (2 mL) were added to a 10 mL thick-walled reaction tube equipped with a magnetic stirring bar. The reaction was stirred at 25 °C − 45 °C under 440 nm blue LED irradiation for 3 h. Then the mixture was monitored by TLC and NMR. The crude product was purified by column chromatography on silica gel (hexane/EtOAc = 40/1) to yield the product **5** as a white solid (0.27 g, 71% yield). **^{1}H NMR** (300 MHz, $CDCl_3$) δ 4.33 – 4.03 (m, 5H), 3.38 (dd, J = 10, 5 Hz, 1H), 3.00 (t, J = 9 Hz, 1H), 2.55 – 2.43 (m, 2H), 2.40 – 2.30 (m, 1H), 2.28 – 2.19 (m, 1H), 2.07 – 1.95 (m, 2H), 1.75 – 1.60 (m, 1H), 1.47 – 1.39 (m, 1H), 1.28 – 1.25 (m, 5H), 1.22 (s, 12H), 1.21 (s, 12H), 0.81 – 0.68 (m, 1H). **^{13}C NMR** (75 MHz, $CDCl_3$) δ 172.5, 172.4, 83.07, 83.06, 61.4, 61.3, 58.3, 45.3, 45.0, 40.3, 38.0, 24.80, 24.77, 24.52, 24.49, 14.02, 13.96, 9.2. **^{11}B NMR** (96 MHz, $CDCl_3$) δ 33.5. **HRMS** (ASAP, m/z): calcd. for $C_{26}H_{46}B_2IO_8$ $[M+H]^+$: 635.2418, found: 635.2411.

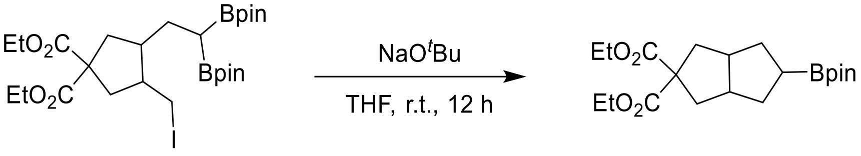

5 **5a**, 76%

In an argon-filled glove box, a 5 mL vial equipped with a magnetic stirrer bar was charged sequentially with **5** (0.3 mmol, 1 equiv.) and THF (1 mL) followed by the addition of NaO^{t}Bu (0.6 mmol, 2 equiv.). The reaction mixture was stirred at room temperature for 12 h. The reaction was monitored by TLC and GC-MS analysis. Then, the mixture was filtered through celite and washed with Et_2O. The combined organic phase was dried using Na_2SO_4 and then concentrated *in vacuo*. The crude product was purified by column chromatography on silica gel (hexane/EtOAc = 40/1) to afford bicyclic boronate ester **5a** as a colorless oil (86.7 mg, 76% yield). **^{1}H NMR** (300 MHz, $CDCl_3$) δ 4.34 – 3.97 (m, 4H), 2.57 – 2.41 (m, 3H), 2.05 – 1.93 (m, 1H), 1.87 – 1.80 (m, 1H), 1.64 – 1.51 (m, 1H), 1.45 – 1.32 (m, 2H), 1.30 – 1.22 (m, 9H), 1.22 (s, 12H). **^{13}C NMR** (75 MHz, $CDCl_3$) δ 172.3, 171.9, 83.0, 64.4, 61.14, 61.08, 44.9, 40.3, 35.8,

24.7, 14.0. **^{11}B NMR** (96 MHz, $CDCl_3$) δ 33.5. **HRMS** (ESI, m/z): calcd. for $C_{20}H_{34}BO_6$ $[M+H]^+$: 381.2443, found: 381.2450.

Stoichiometric radical initiator experiments

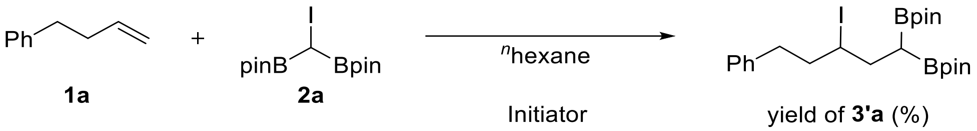

Initiator	yield of **3'a** (%)
AIBN (1.2 equiv.)	N.
AIBN (0.5 equiv.)	N.
BEt_3/O_2 (1.2 equiv.)	27%
BEt_3/O_2 (0.5 equiv.)	trace
DLP (1.2 equiv.)	41%
DLP (0.5 equiv.)	17%

Alkene **1a** (0.3 mmol, 1 equiv.) and **2a** (0.36 mmol, 1.2 equiv.) in ^{n}hexane (1 mL) were added to a 5 mL thick-walled reaction tube equipped with a magnetic stirring bar. To this mixture was added 2,2'-azobis(2-methylpropionitrile) (AIBN, 0.5 or 1.2 equiv.) at room temperature. After the addition was finished, the reaction was stirred for 3 hours at 70 ^{o}C. Then the mixtures were monitored by NMR and GC-MS analysis. No desired product was formed.

Alkene **1a** (0.3 mmol, 1 equiv.) and **2a** (0.36 mmol, 1.2 equiv.) in ^{n}hexane (1 mL) were added to a 5 mL thick-walled reaction tube equipped with a magnetic stirring bar. To this mixture was added BEt_3 (0.5 or 1.2 equiv., 1.0 M in hexanes) at 0 ^{o}C. Then O_2 was bubbled through the solution via syringe pump at 0 ^{o}C. After the addition was finished, the reaction was warmed up to room temperature and stirred for another 2 hours. Then the mixtures were monitored by NMR and GC-MS analysis. Stoichiometric BEt_3 (1.2 equiv.) provided 27% of the desired product while substoichiometric BEt_3 (0.5 equiv.) provided trace amounts of the desired product.

Alkene **1a** (0.3 mmol, 1 equiv.) and **2a** (0.36 mmol, 1.2 equiv.) in ^{n}hexane (1 mL) were added to a 5 mL thick-walled reaction tube equipped with a magnetic stirring bar. To this mixture was added dilauroyl peroxide (DLP, 0.5 or 1.2 equiv.) at room temperature. After the addition was finished, the reaction was stirred for 3 hours at 70 ^{o}C. Then the mixtures were monitored by NMR and GC-MS analysis. Stoichiometric DLP (1.2 equiv.) provided 41% of the desired product while substoichiometric DLP (0.5 equiv.) provided only 17% of the desired product.

Competition experiment

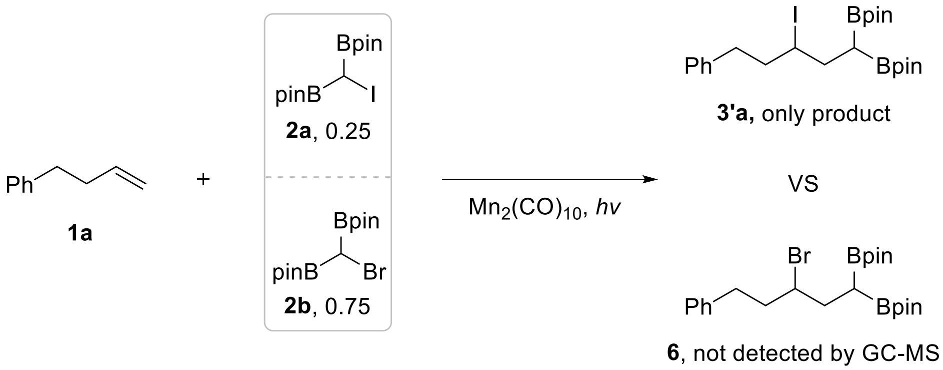

In an argon-filled glove box, **2a** (0.09 mmol), **2b** (0.27 mmol), $Mn_2(CO)_{10}$ (10 mol%), and **1a** (0.3 mmol, 1 equiv.) in ^{n}hexane (1 mL) were added to a 5 mL thick-walled reaction tube equipped with a magnetic stirring bar. After the addition was finished, the reaction was stirred at 25 °C - 45 °C under 440 nm blue LED irradiation for 3 h. Only the iodine containing product **3′a** was observed by NMR or GC-MS.

3 Synthetic Diversification and Applications

Synthesis of 1,1-allylic diboronic esters

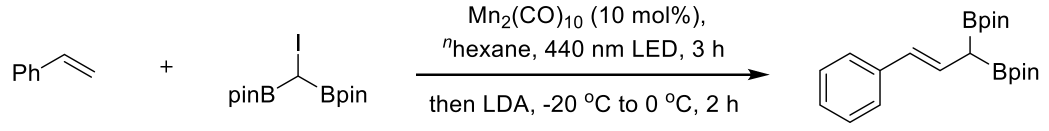

1ab **2a** **7**, 53%

In an argon-filled glove box, a 5 mL vial equipped with a magnetic stirrer bar was charged sequentially with $CHI(Bpin)_2$ **2a** (0.36 mmol, 1.2 equiv.), $Mn_2(CO)_{10}$ (10 mol%) followed by the addition of DCM (1 mL) and alkenes **1ab** (0.3 mmol, 1 equiv.). The reaction mixture was stirred at 25 °C − 45 °C under irradiation with 440 nm blue LEDs for 3 h. After irradiation, the reaction mixture was cooled to -20 °C, and LDA (2.5 M, 1.2 equiv. in THF) was added, followed by stirring for 2 h at 0 °C. The reaction was monitored by TLC and GC-MS analysis. Then, the mixture was filtered through celite and washed with DCM. The combined organic phase was dried using Na_2SO_4 and then concentrated *in vacuo*. The crude product was purified by distillation under reduced pressure (130 °C/4 mmHg) to afford the corresponding product **7** as a white solid (58.9 mg, 53% yield). **^{1}H NMR** (300 MHz, $CDCl_3$) δ 7.37 (d, J = 7 Hz, 2H), 7.28 (t, J = 8 Hz, 2H), 7.16 (t, J = 7 Hz, 1H), 6.46 (dd, J = 16, 10 Hz, 1H), 6.30 (d, J = 16 Hz, 1H), 2.02 (d, J = 10 Hz, 1H), 1.27 (s, 12H), 1.26 (s, 12H). **^{13}C NMR** (75 MHz, $CDCl_3$) δ 138.6, 128.4, 128.3, 127.7, 126.1, 125.8, 83.4, 24.7, 24.6. **^{11}B NMR** (96 MHz, $CDCl_3$) δ 32.7. **HRMS** (ESI, m/z): calcd. for $C_{21}H_{33}B_2O_4$ $[M+H]^+$: 371.2559, found: 371.2556.

The spectroscopic data for **7** match those reported in the literature.[17]

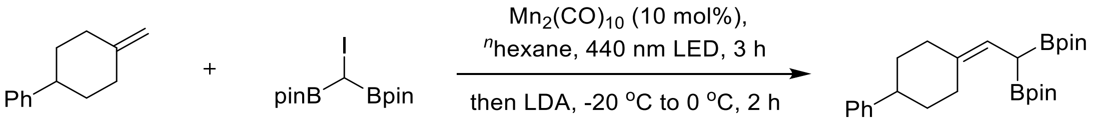

1ac **2a** **8**, 49%

In an argon-filled glove box, a 5 mL vial equipped with a magnetic stirrer bar was charged sequentially with $CHI(Bpin)_2$ **2a** (0.36 mmol, 1.2 equiv.), $Mn_2(CO)_{10}$ (10 mol%) followed by the addition of DCM (1 mL) and alkenes **1ac** (0.3 mmol, 1 equiv.). The reaction mixture was stirred at 25 °C − 45 °C under 440 nm blue LEDs for 3 h. After irradiation, the reaction mixture was cooled to -20 °C, and LDA (2.5 M, 1.2 equiv. in THF) was added, followed by stirring for 2 h at 0 °C. The reaction was monitored by TLC and GC-MS analysis. Then, the mixture was filtered through celite and washed with DCM. The combined organic phase was dried using Na_2SO_4 and then concentrated *in vacuo*. The crude product was purified by distillation under reduced pressure (141 °C/4 mmHg) to afford the corresponding product **8** as a colorless oil (64.4 mg, 49% yield). **^{1}H NMR** (300 MHz, $CDCl_3$) δ 7.35 – 7.29 (m, 2H), 7.25 – 7.17 (m, 3H), 5.52 (s, 1H), 2.78 – 2.66 (m, 1H), 2.30 – 2.03 (m, 6H), 1.98 – 1.92 (m, 1H), 1.83 – 1.69 (m, 1H),

1.26 (s, 12H), 1.25 (s, 12H), 1.04 (t, J = 8 Hz, 1H). **^{13}C NMR** (75 MHz, $CDCl_3$) δ 147.5, 139.5, 128.2, 126.9, 125.8, 119.1, 82.9, 40.3, 33.4, 32.8, 30.1, 29.2, 24.8, 24.5. **^{11}B NMR** (96 MHz, $CDCl_3$) δ 33.4. **HRMS** (ESI, m/z): calcd. for $C_{26}H_{41}B_2O_4$ $[M+H]^+$: 439.3185, found: 439.3182.

Synthesis of γ-halogenated gem-bis(boronates)

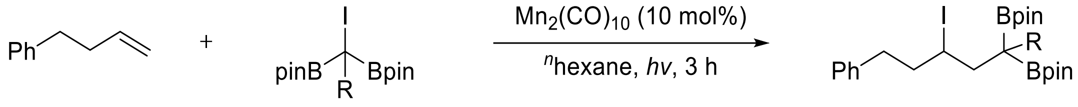

1a **2** **3'**

General procedure 2

In an argon-filled glove box, a 5 mL vial equipped with a magnetic stirrer bar was charged sequentially with substituted *gem*-diboryl iodoalkyl compound **2** (0.36 mmol, 1.2 equiv.), $Mn_2(CO)_{10}$ (10 mol%) followed by the addition of ^{n}hexane (1 mL) and alkenes **1a** (0.3 mmol, 1 equiv.). The reaction mixture was stirred at 25 °C − 45 °C under 440 nm blue LEDs for 3 h. The reaction was monitored by TLC and GC-MS analysis. Then, the mixture was filtered through celite and washed with DCM. The combined organic phase was dried using Na_2SO_4 and then concentrated *in vacuo*. The crude product was purified by flash column chromatography (hexane/EtOAc).

2,2'-(4-iodo-1,6-diphenylhexane-2,2-diyl)bis(4,4,5,5-tetramethyl-1,3,2-dioxaborolane) (3'b)

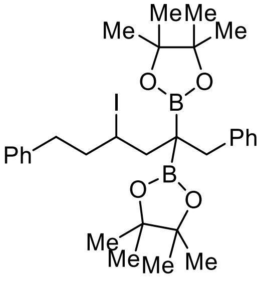

3'b

According to **General procedure 2** with 2,2'-(1-iodo-2-phenylethane-1,1-diyl)bis(4,4,5,5-tetramethyl-1,3,2-dioxaborolane) (174.2 mg, 0.36 mmol, 1.2 equiv.), the reaction mixture was purified by column chromatography on silica gel (hexane/EtOAc = 30/1) to yield the product **3'b** as a colorless oil (120.2 mg, 65% yield).

^{1}H NMR (400 MHz, $CDCl_3$): δ 7.36 – 7.29 (m, 5H), 7.25 – 7.17 (m, 5H), 4.02 – 3.91 (m, 1H), 3.01 – 2.91 (m, 1H), 2.67 – 2.62 (m, 1H), 2.58 – 2.54 (m, 2H), 2.39 (dd, J = 15, 8 Hz, 1H), 2.13 – 2.02 (m, 1H), 1.90 – 1.80 (m, 1H), 1.61 – 1.57 (m, 1H), 1.28 (s, 12H), 1.21 (s, 12H). **^{13}C NMR** (101 MHz, $CDCl_3$): δ 141, 129.3, 128.6, 128.4, 127.8, 126, 125.6, 83.5, 83.5, 42.3, 40.9, 38.5, 36.5, 34.5, 25.2, 25, 24.91, 24.86.

^{11}B NMR (128 MHz, $CDCl_3$): δ 33.4. **HRMS-ESI** (m/z): Calculated (found) for $C_{30}H_{44}B_2IO_4$ $[M+H]^+$ 617.2465 (617.2461).

<u>2,2'-(6-iodo-1,8-diphenyloctane-4,4-diyl)bis(4,4,5,5-tetramethyl-1,3,2-dioxaborolane) (3'c)</u>

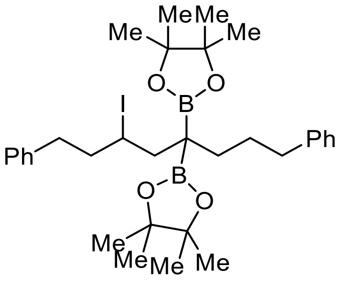

3'c

According to **General procedure 2** with 2,2'-(1-iodo-4-phenylbutane-1,1-diyl)bis(4,4,5,5-tetramethyl-1,3,2-dioxaborolane) (185.4 mg, 0.36 mmol, 1.2 equiv.), the reaction mixture was purified by column chromatography on silica gel (hexane/EtOAc = 30/1) to yield the product **3'c** as a colorless oil (121.8 mg, 63% yield).

^{1}H NMR (400 MHz, $CDCl_3$): δ 7.36 – 7.29 (m, 5H), 7.25 – 7.17 (m, 5H), 4.03 – 3.92 (m, 1H), 3.01 – 2.91 (m, 1H), 2.65 – 2.53 (m, 4H), 2.39 (dd, J = 15, 8 Hz, 1H), 2.13 – 2.02 (m, 1H), 1.89 – 1.81 (m, 1H), 1.78 – 1.72 (m, 1H), 1.65 – 1.51 (m, 2H), 1.50 – 1.41 (m, 1H), 1.28 (s, 12H), 1.21 (s, 12H). **^{13}C NMR** (101 MHz, $CDCl_3$): δ 142.9, 141.1, 128.7, 128.4, 128.3, 126, 125.6, 83.4, 83.3, 42.4, 40.6, 38.0, 36.7, 36.6, 29.4, 28.3, 25, 24.9, 24.77, 24.67. **^{11}B NMR** (128 MHz, $CDCl_3$): δ 33.8. **HRMS-ESI** (m/z): Calculated (found) for $C_{32}H_{48}B_2IO_4$ $[M+H]^+$ 645.2778 (645.2771).

<u>2,2'-(1-cyclopentyl-3-iodo-5-phenylpentane-1,1-diyl)bis(4,4,5,5-tetramethyl-1,3,2-dioxaborolane) (3'd)</u>

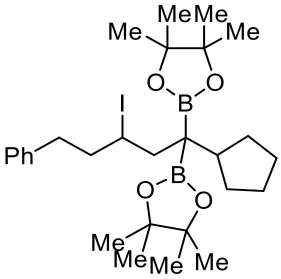

3'd

According to **General procedure 2** with 2,2'-(cyclopentyliodomethylene)bis(4,4,5,5-tetramethyl-1,3,2-dioxaborolane) (166.3 mg, 0.36 mmol, 1.2 equiv.), the reaction mixture was purified by column chromatography on silica gel (hexane/EtOAc = 30/1) to yield the product **3'd** as a colorless oil (80.2 mg, 45% yield).

^{1}H NMR (400 MHz, $CDCl_3$): δ 7.29 – 7.26 (m, 2H), 7.26 – 7.15 (m, 3H), 4.43 – 4.32 (m, 1H), 3.05 – 2.91 (m, 1H), 2.71 – 2.53 (m, 2H), 2.44 (dd, J = 15, 9 Hz, 1H), 2.11 – 1.90 (m, 3H), 1.85 – 1.72 (m, 2H), 1.53 (q, J = 7 Hz, 2H), 1.43 (dt, J = 7, 4 Hz, 2H), 1.37 – 1.31 (m, 1H), 1.27 (s, 1H), 1.19 (s, 12H), 1.18 (s, 12H). **^{13}C NMR** (101 MHz, $CDCl_3$): δ 141.4, 128.6, 128.3, 125.8, 82.9, 82.8, 43.8, 42.9, 41.1, 40.2, 37.2, 30.6, 30.3, 25.6, 25.5, 24.92, 24.88, 24.8. **^{11}B NMR** (96 MHz, $CDCl_3$): δ 33.3. **HRMS-ESI** (m/z): Calculated (found) for $C_{28}H_{46}B_2IO_4$ $[M+H]^+$ 595.2621 (595.2616).

2,2'-(1-(4-bromophenyl)-5-iodo-7-phenylheptane-3,3-diyl)bis(4,4,5,5-tetramethyl-1,3,2-dioxaborolane) (3'e)

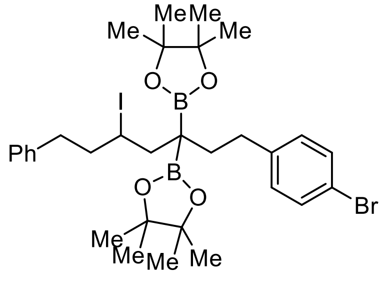

3'e

According to **General procedure 2** with 2,2'-(3-(4-bromophenyl)-1-iodopropane-1,1-diyl)bis(4,4,5,5-tetramethyl-1,3,2-dioxaborolane) (207.7 mg, 0.36 mmol, 1.2 equiv.), the reaction mixture was purified by column chromatography on silica gel (hexane/EtOAc = 30/1) to yield the product **3'e** as a white solid (151.0 mg, 71% yield).

^{1}H NMR (400 MHz, $CDCl_3$): δ 7.35 (d, J = 7 Hz, 2H), 7.32 – 7.19 (m, 5H), 6.87 (d, J = 7 Hz, 2H), 4.08 (q, J = 9 Hz, 1H), 3.06 – 2.92 (m, 1H), 2.83 – 2.58 (m, 2H), 2.52 – 2.43 (m, 2H), 2.32 – 2.07 (m, 2H), 1.92 (dt, J = 16, 8 Hz, 1H), 1.84 – 1.73 (m, 2H), 1.27 (s, 12H), 1.22 (s, 12H). **^{13}C NMR** (101 MHz, $CDCl_3$): δ 141.9, 140.7, 131.1, 130.2, 128.7, 128.4, 126, 119.1, 83.4, 83.4, 42.2, 40.6, 37.3, 36.3, 33.2, 31.1, 25, 24.9, 24.8, 24.6. **^{11}B NMR** (96 MHz, $CDCl_3$): δ 33.5. **HRMS-ESI** (m/z): Calculated (found) for $C_{31}H_{45}B_2BrIO_4$ $[M+H]^+$ 709.1727 (709.1722).

5 mmol scale synthesis of 3a

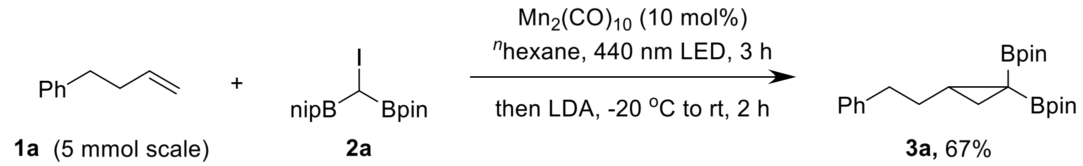

In an argon-filled glove box, a 50 mL vial equipped with a magnetic stirrer bar was charged sequentially with (diboronmethyl)iodides **2a** (6 mmol, 1.2 equiv.), $Mn_2(CO)_{10}$ (10 mol%) followed by the addition of ^{n}hexane (15 mL) and but-3-en-1-ylbenzene **1a** (5 mmol, 1 equiv.). The reaction mixture was stirred under

irradiation with 440 nm blue LEDs for 5 h. After irradiation, the reaction mixture was cooled to -20 °C, and LDA (2.5 M, 1.2 equiv. in THF) was added, followed by stirring for 2 h at 0 °C. Then, the mixture was filtered through celite and washed with DCM. The combined organic phase was dried using Na_2SO_4 and then concentrated *in vacuo*. The crude product was purified by column chromatography on silica gel (hexane/EtOAc = 60/1) to afford the corresponding product **3a** as a colorless oil (1.3 g, 67% yield).

Deborylative protonation

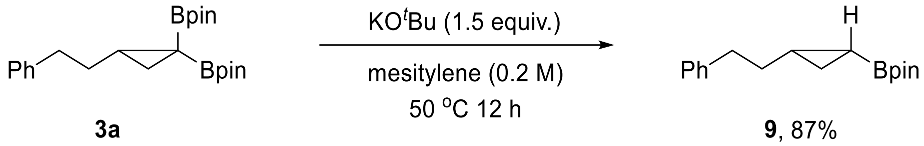

3a **9**, 87%

In an argon-filled glove box, a 10 mL vial equipped with a magnetic stirrer bar was charged sequentially with **3a** (0.3 mmol, 1 equiv.) and mesitylene (1 mL) followed by the addition of KO^{t}Bu (0.45 mmol, 1.5 equiv.). The reaction mixture was stirred at 50 °C for 12 h. The reaction was monitored by TLC and GC-MS analysis. Then, the mixture was filtered through celite and washed with DCM. The combined organic phase was dried using Na_2SO_4 and then concentrated *in vacuo*. The crude product was purified by column chromatography on silica gel (hexane/EtOAc = 50/1) to afford the corresponding product **9** as a colorless oil (71 mg, 87% yield).

^{1}H NMR (300 MHz, $CDCl_3$) δ 7.31 – 7.26 (m, 2H), 7.25 – 7.15 (m, 3H), 2.79 – 2.68 (m, 2H), 1.63 – 1.56 (m, 2H), 1.26 (s, 6H), 1.25 (s, 6H), 1.05 – 0.91 (m, 1H), 0.65 – 0.77 (m, 1H), 0.48 – 0.41 (m, 1H), -0.42 – 0.31 (m, 1H). **^{13}C NMR** (75 MHz, $CDCl_3$) δ 142.5, 128.5, 128.2, 125.6, 82.8, 37.3, 36.0, 24.7, 24.7, 18.0, 11.5. **^{11}B NMR** (96 MHz, $CDCl_3$) δ 32.9.

The spectroscopic data for **9** match those reported in the literature.[2]

Suzuki-Miyaura coupling of gem-bis(boryl)cyclopropane

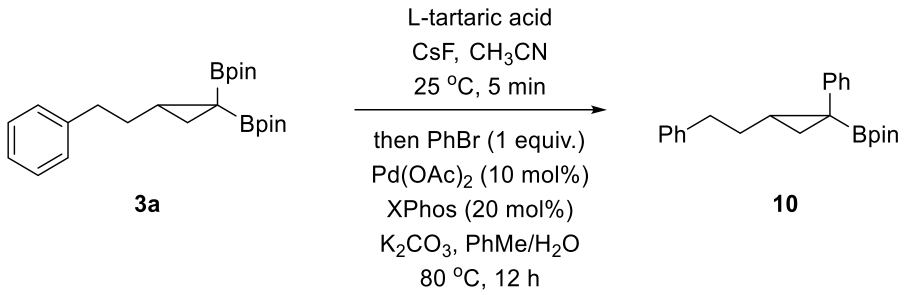

3a **10**

In an open flask, **3a** (0.5 mmol, 1 equiv) was dissolved in acetonitrile (2 mL) and methanol (2 mL). Then, a solution of cesium fluoride (2 mmol, 304 mg) in H_2O (0.3 mL) was added dropwise, and the mixture was stirred at room temperature for 5 min. Then, L-(+)-tartaric acid (1.02 mmol, 154 mg) in THF (1 mL) was added dropwise to the rapidly stirring clear solution, during which a white precipitate formed. The

reaction was complete, as detected by TCL. The reaction mixture was filtered to remove the white precipitate and washed thoroughly with excess acetonitrile (10 mL), Then, the filtrate was concentrated to obtain the corresponding cesium organotrifluoroborate as an amorphous solid, which was further dried under high vacuum.[18]

In an argon-filled glove box, a 10 mL vial equipped with a magnetic stirrer bar was charged sequentially with $Pd(OAc)_2$ (0.02 mmol, 10 mol%), and XPhos (0.04 mmol, 20 mol%). To this solid, K_2CO_3 (0.6 mmol, 3 equiv.), cesium organotrifluoroborate (0.24 mmol, 1.2 equiv.), and bromobenzene (0.2 mmol, 1 equiv.) were added followed by toluene (1.5 mL) and H_2O (0.15 mL), and the tube was sealed and removed from the glove box. The mixture was then heated at 80 °C in a preheated oil bath for 12 h. The reaction mixture was then cooled and the solvent was evaporated *in vacuo*. The crude product was purified by column chromatography on silica gel (hexane/EtOAc = 60/1) to afford the corresponding product **10** as a colorless oil (49.4 mg, 71% yield). **^{1}H NMR** (400 MHz, CD_2Cl_2) δ 7.34 – 7.26 (m, 4H), 7.25 – 7.18 (m, 5H), 7.24 – 7.12 (m, 1H), 2.85 (m, 2H), 2.02 – 1.92 (m, 2H), 1.31 (d, J = 5 Hz, 1H), 1.28-1.25 (s, 6H), 1.25-1.23 (s, 6H), 1.21 (d, J = 8 Hz, 1H), 1.13 (dd, J = 8, 4 Hz, 1H), 1.06 (dd, J = 6, 4 Hz, 1H). **^{13}C NMR** (101 MHz, CD_2Cl_2) δ 146.4, 142.6, 129.4, 128.5, 128.2, 127.8, 125.6, 125.1, 83.4, 36.2, 32.2, 27.9, 24.7, 24.4, 19.3.

The spectroscopic data for **10** match those reported in the literature.[19]

<u>Oxidation of gem-bis(boryl)cyclopropane 3a</u>

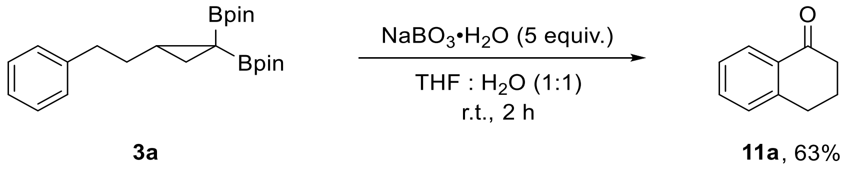

In an open flask, **3a** (0.2 mmol, 1 equiv.) was dissolved in THF (1 mL). Then, sodium perborate (100 mg, 1 mmol, 5 equiv.) was added, followed by the addition of H_2O (1 mL), and the reaction was stirred for 5 h at room temperature. Then, the reaction was quenched with H_2O (2 mL), and the mixture was extracted with EtOAc (3 × 5 mL). The organic layers were dried over Na_2SO_4 and then concentrated *in vacuo*. The crude product was purified by column chromatography on silica gel (hexane/EtOAc = 30/1) to afford the corresponding product **11a** as a colorless oil (18 mg, 63% yield). **^{1}H NMR** (400 MHz, $CDCl_3$) δ 8.06 (d, J = 8 Hz, 1H), 7.50 (m, 1H), 7.38 – 7.28 (m, 2H), 3.00 (t, J = 6 Hz, 2H), 2.71 – 2.67 (m, 2H), 2.20 – 2.15 (m, 2H). **^{13}C NMR** (101 MHz, $CDCl_3$) δ 198.4, 144.5, 133.4, 132.6, 128.8, 127.2, 126.7, 39.2, 29.7, 23.3.

The spectroscopic data for **11a** match those reported in the literature.[20]

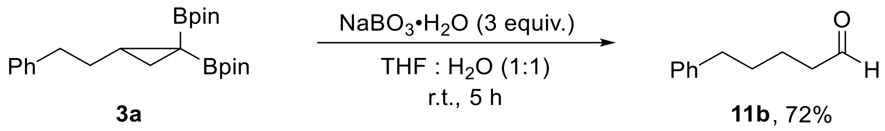

In an open flask, **3a** (0.2 mmol, 1 equiv) was dissolved in THF (1 mL). Then, sodium perborate (60 mg, 0.6 mmol, 3 equiv) was added, followed by the addition of H_2O (1 mL), and the reaction was stirred for 5 h at room temperature. Then, the reaction was quenched with H_2O (2 mL), and the mixture was extracted with EtOAc (3 × 5 mL). The organic layers were dried over Na_2SO_4 and then concentrated *in vacuo*. The crude product was purified by column chromatography on silica gel (hexane/EtOAc = 40/1) to afford the corresponding product **11b** as a colorless oil (23 mg, 72% yield). **^{1}H NMR** (400 MHz, $CDCl_3$) δ 9.78 (s, 1H), 7.34 – 7.26 (m, 2H), 7.24 – 7.18 (m, 3H), 2.70 – 2.62 (m, 2H), 2.52 – 2.44 (m, 2H), 1.74 – 1.66 (m, 4H). **^{13}C NMR** (101 MHz, $CDCl_3$) δ 202.6, 141.9, 128.38, 128.37 125.9, 43.8, 35.6, 30.9, 21.7.

The spectroscopic data for **11b** match those reported in the literature.[21]

<u>Radical borylation for 1,1,2-alkyltriboronate</u>

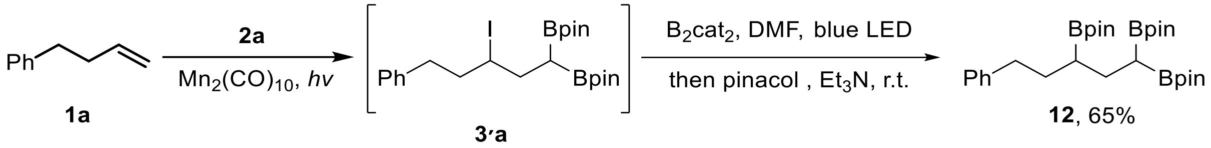

In an argon-filled glove box, a 10 mL vial equipped with a magnetic stirrer bar was charged sequentially with $CHI(Bpin)_2$ **2a** (0.36 mmol, 1.2 equiv.), $Mn_2(CO)_{10}$ (10 mol%) followed by the addition of ^{n}Hexane (1 mL) and alkenes **1a** (0.3 mmol, 1 equiv.). The reaction mixture was stirred at 25 °C − 45 °C under 440 nm blue LED irradiation for 3 h. The crude product (**3′a**) is concentrated *in vacuo*, and directly used in the next reaction step. To a Schlenk tube were added **3′a** and bis(catecholato)diboron (B_2cat_2, 1.2 mmol, 4 equiv.). The reaction vessel was evacuated and back filled with Ar three times. Dimethylformamide (0.6 mL) was added. The reaction mixture was stirred under blue LED irradiation at room temperature for 24 h. Then, a solution of pinacol (1.2 mmol) in triethylamine (1 mL) was added to the mixture. After 1 h, water (15 mL) was added, and the aqueous layer was extracted with ethyl acetate (3 ×15 mL). The combined organic layers were dried over Na_2SO_4, filtered and concentrated *in vacuo*. The crude product was purified by flash column chromatography on silica gel with petroleum ether/ethyl acetate as eluent to give the corresponding product **12** as a colorless oil (102.6 mg, 65% yield). **^{1}H NMR** (300 MHz, $CDCl_3$) δ 7.30 – 7.24 (m, 2H), 7.23 – 7.11 (m, 3H), 2.62 (td, J = 7, 3 Hz, 2H), 1.77 – 1.65 (m, 4H), 1.27 (s, 12H), 1.23 (s, 12H), 1.22 (s, 12H), 1.14 (d, J = 8 Hz, 1H), 0.90 (dd, J = 10, 6 Hz, 1H). **^{13}C NMR** (75 MHz, $CDCl_3$) δ 143.3, 128.5, 128.1, 125.4, 82.9, 82.8, 35.5, 33.6, 26.7, 25.0, 24.9, 24.6. **^{11}B NMR** (96 MHz, $CDCl_3$) δ 33.0. **HRMS** (ESI, m/z): calcd. for $C_{23}H_{38}B_2IO_4$ $[M+H]^+$: 527.3881, found: 527.3872.

The spectroscopic data for **12** match those reported in the literature.[3]

Heck-type cross-coupling for γ-substituted gem-diborylalkane

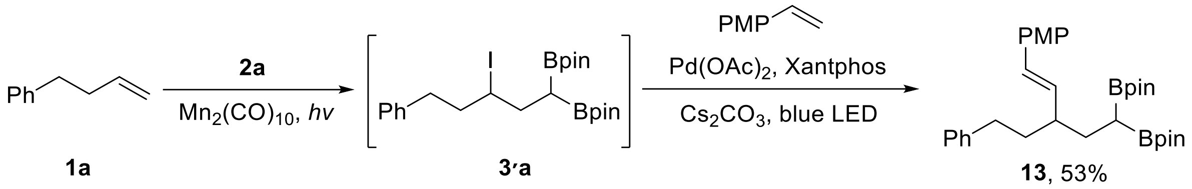

In an argon-filled glove box, a 10 mL vial equipped with a magnetic stirrer bar was charged sequentially with $CHI(Bpin)_2$ **2a** (0.36 mmol, 1.2 equiv.) and $Mn_2(CO)_{10}$ (10 mol%) followed by the addition of ^{n}hexane (1 mL) and alkenes **1a** (0.3 mmol, 1 equiv.). The reaction mixture was stirred at 25 °C − 45 °C under 440 nm blue LED irradiation for 3 h. The crude product (**3′a**) was concentrated *in vacuo*, and directly used in the next reaction step. To a Schlenk tube were added **3′a**, $Pd(OAc)_2$ (10 mol%), Xantphos (20 mol%), Cs_2CO_3 (3 equiv.), benzene (1 mL), and 1-methoxy-4-vinylbenzene (0.45 mmol, 1.5 equiv.). The reaction mixture was stirred at 25 °C − 45 °C under blue LED irradiation for 24 h.[22] The reaction was monitored by TLC and GC-MS analysis. Then, the mixture was filtered through celite and washed with Et_2O. The combined organic phase was dried using Na_2SO_4 and then concentrated *in vacuo*. The crude product was purified by column chromatography on silica gel (hexane/EtOAc = 20/1) to afford the corresponding product **13** as a colorless oil (84.8 mg, 53% yield). **^{1}H NMR** (300 MHz, $CDCl_3$) δ 7.31 – 7.23 (m, 3H), 7.19 – 7.08 (m, 4H), 6.83 (d, J = 9 Hz, 2H), 6.25 (d, J = 16 Hz, 1H), 5.76 (dd, J = 16, 9 Hz, 1H), 3.80 (s, 3H), 2.71 – 2.54 (m, 2H), 2.14 – 1.99 (m, 1H), 1.87 – 1.74 (m, 2H), 1.61 – 1.52 (m, 2H), 1.20 (s, 6H), 1.18 (s, 6H), 1.15 (s, 12H), 0.89 – 0.83 (m, 1H). **^{13}C NMR** (75 MHz, $CDCl_3$) δ 158.6, 142.8, 132.8, 130.8, 130.2, 128.5, 128.1, 127.2, 125.4, 113.7, 82.9, 55.3, 45.2, 37.1, 33.7, 31.3, 24.9, 24.8, 24.6. **^{11}B NMR** (96 MHz, $CDCl_3$) δ 32.9. **HRMS** (ESI, m/z): calcd. for $C_{32}H_{47}B_2O_5$ $[M+H]^+$: 533.3604, found: 533.3598.

4 Single-Crystal X-Ray Diffraction Analysis

A crystal suitable for single-crystal X-ray diffraction was selected, coated in perfluoropolyether oil, and mounted on a microloop. Diffraction data for **3c** were collected at 296 K on a Bruker D8 Quest X-ray diffractometer equipped with graphite-monochromatized MoK_{α} radiation. Data reduction was done with the Bruker Saint program. The structures were solved by direct methods and refined with the full-matrix least squares technique using the SHELXTL package.[23] Hydrogen atoms were placed in calculated positions with isotropic displacement parameters set to 1.2× Ueq of the attached atom. Diamond[24] software was used for graphical representation. Other structural information was extracted using Mercury[25] and OLEX2[26] software. Crystal data and experimental details are listed in Table S5; full structural information has been deposited with Cambridge Crystallographic Data Centre (CCDC: 2382460). This data can be obtained free of charge from The Cambridge Crystallographic Data Centre via www.ccdc.cam.ac.uk/data_request/cif.

Table S6: Single-crystal X-ray diffraction data and refinement details of **3c**.

Data	**3c**
CCDC number	2382460
Empirical formula	$C_{22}H_{34}B_2O_4$
Formula weight / g·mol^{-1}	384.11
T / K	296.15
Radiation, λ / Å	MoK$_\alpha$ 0.71073
Crystal size / mm³	0.12 × 0.1 × 0.08
Crystal color, habit	Colorless block
μ / mm^{-1}	0.074
Crystal system	monoclinic
Space group	$P2_1/c$
a / Å	13.1753(17)
b / Å	15.5076(19)
c / Å	12.1845(15)
α / °	90
β / °	115.425(3)
γ / °	90
Volume / Å^3	2248.4(5)
Z	4
ρ_{calc} / g·cm^{-3}	1.135
F(000)	832.0
2θ range / °	4.538 – 50.56
Reflections collected	27543
Unique reflections	4056
Parameters / restraints	302 / 132
GooF on F^2	1.019
R_1 [I>2σ (I)]	0.0988
wR^2 (all data)	0.2878
Max. / min. residual electron density / e·Å^{-3}	0.40 / –0.50

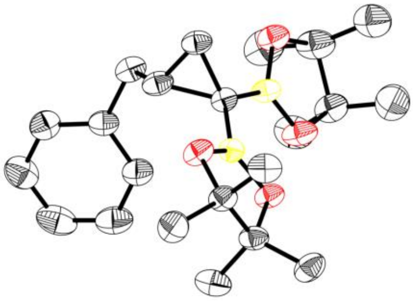

Figure S2. The solid-state molecular structure of **3c** determined by single-crystal X-ray diffraction at 296 K. All ellipsoids are drawn at the 50% probability level. H atoms are omitted for clarity. Both Bpin moieties are disordered and only the major part (51%) is shown here.

4 References

1. S. K. Bose, S. Brand, H. O. Omoregie, M. Haehnel, J. Maier, G. Bringmann and T. B. Marder, *ACS Catal.*, 2016, **6**, 8332–8335.
2. J. Hu, M. Tang, J. Wang, Z. Wu, A. Friedrich and T. B. Marder, *Angew. Chem. Int. Ed.*, 2023, **62**, e202305175.
3. Y. Zhao and S. Ge, *Angew. Chem. Int. Ed.*, 2022, **61**, e202116133.
4. F. Chang, C. Wang, Q. Chen, Y. Zhang and G. Liu, *Angew. Chem. Int. Ed.*, 2022, **61**, e202114809.
5. A. J. Cresswell, S. T.-C. Eey and S. E. Denmark, *Nat. Chem.*, 2015, **7**, 146–152.
6. J. J. Melder, M. L. Heldner, R. Kugler, L. A. Ziegenhagen, F. Rominger, M. Rudolph and S. K. Hashmi, *J. Am. Chem. Soc.*, 2024, **146**, 14521–14527.
7. W. H. Watanabe, L. E. Conlon and J. C. H. Hwa, *J. Org. Chem.*, 1958, **23**, 1666–1668.
8. F. Wang, P. Xu, F. Cong and P. Tang, *Chem. Sci.*, 2018, **9**, 8836–8841.
9. Y. Wang, J. Wang, G.-X. Li, G. He and G. Chen, *Org. Lett.*, 2017, **19**, 1442–1445.
10. K. Zhang, J.-B. Liu and F.-L. Qing, *Chem. Commun.*, 2014, **50**, 14157–14160.
11. D. Gao and C. Cui, *Chem. Eur. J.*, 2013, **19**, 11143–11147.
12. H. Oh, A. Park, K. Jeong, S.-B. Han and H. Lee, *Adv. Syn. Catal.*, 2019, **361**, 2136–2140.
13. E. L. Glaisyer, M. S. Watt and K. I. Booker-Milburn, *Org. Lett.*, 2018, **20**, 5877–5880.
14. T. Qin, J. Cornella, C. Li, L. R. Malins, J. T. Edwards, S. Kawamura, B. D. Maxwell, M. D. Eastgate and P. S. Baran, *Science*, 2016, **352**, 801–805.
15. Y.-G. Li, L. Li, M.-Y. Yang, G. He and E. A. B. Kantchev, *J. Org. Chem.*, 2017, **82**, 4907–4917.
16. S. Yang, H. Hu and M. Chen, *Org. Lett.*, 2023, **25**, 7968–7973.
17. M. Shin, M. Kim, C. Hwang, H. Lee, H. Kwon, J. Park, E. Lee and S. H. Cho, *Org. Lett.*, 2020, **22**, 2476–2480.
18. N. Kumar, R. R. Reddy and A. Masarwa, *Chem. Eur. J.*, 2019, **25**, 8008–8012.
19. D. T. Ngo, J. J. A. Garwood and D. A. Nagib, *J. Am. Chem. Soc.*, 2024, **146**, 24009–2401.
20. J. Liu, K.-F. Hu, J.-P. Qu and Y.-B. Kang, *Org. Lett.*, 2017, **19**, 5593–5596.
21. N. Xiong, Y. Li and R. Zeng, *ACS Catal.*, 2023, **13**, 1678–1685.
22. C. M. McMahon and E. J. Alexanian, *Angew. Chem. Int. Ed.*, 2014, **53**, 5974–5977.
23. G. M. Sheldrick, *Acta Crystallogr. A Found Adv.*, 2015, **71**, 3−8.
24. K. Brandenburg, Diamond (version 4.4.0), Crystal and Molecular Structure Visualization, Crystal Impact H. Putz & K. Brandenburg GbR, Bonn (Germany), 2017.
25. C. F. Macrae, I. J. Bruno, J. A. Chisholm, P. R. Edgington, P. McCabe, E. Pidcock, L. Rodriguez-Monge, R. Taylor, J. van de Streek and P. A. Wood, *J. Appl. Crystallogr.*, 2008, **41**, 466−470.

26. O. V. Dolomanov, L. J. Bourhis, R. J. Gildea, J. A. K. Howard and H. Puschmann, *J. Appl. Crystallogr.*, 2009, **42**, 339−341.

6 NMR Spectra

^{1}H NMR spectrum of compound **1i** in $CDCl_3$ (400 MHz).

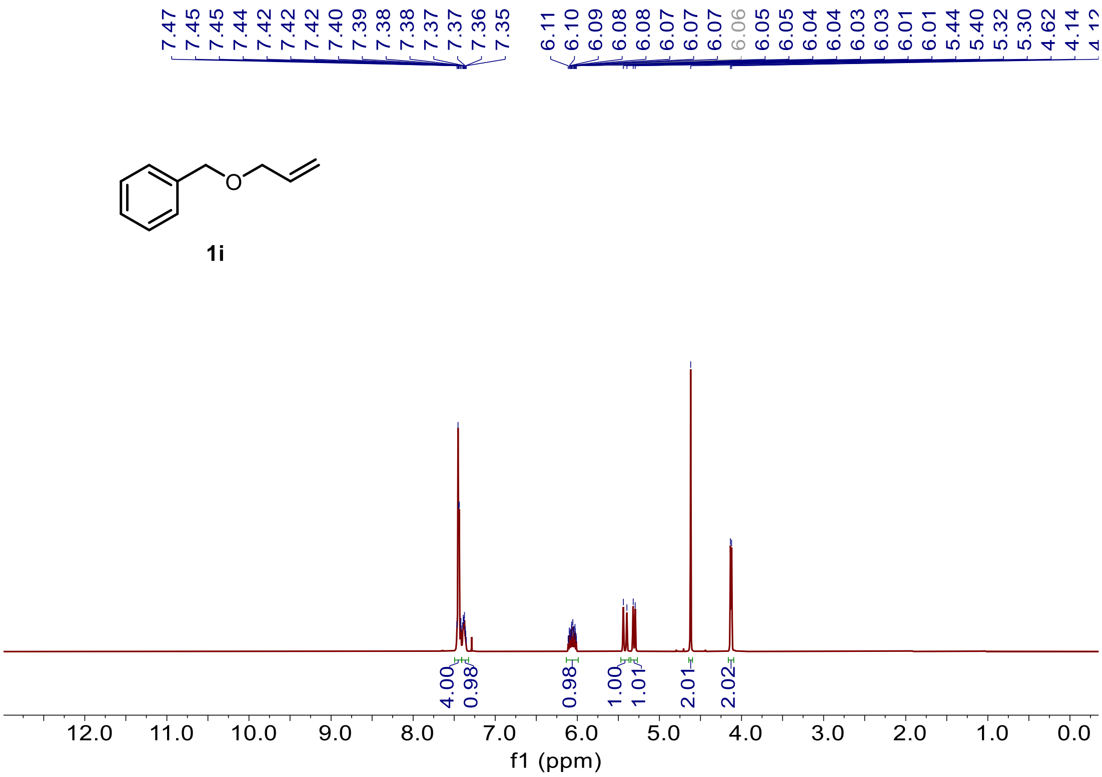

^{13}C NMR spectrum of compound **1i** in $CDCl_3$ (101 MHz).

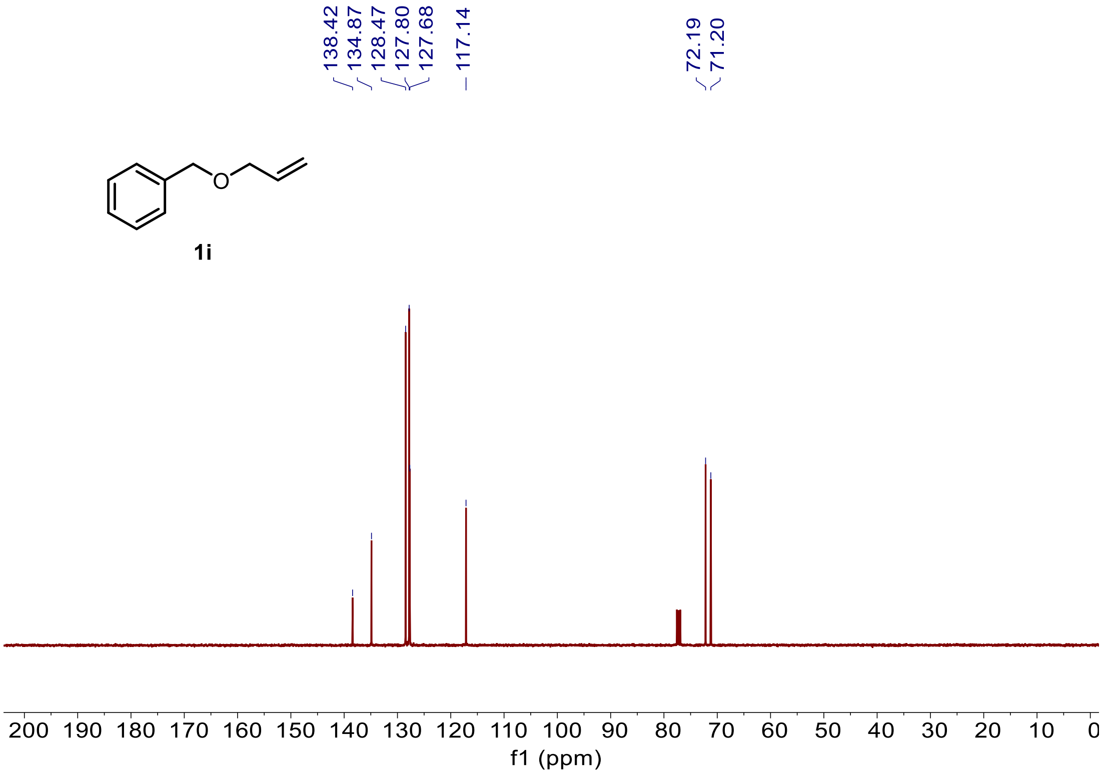

^{1}H NMR spectrum of compound **1j** in $CDCl_3$ (400 MHz).

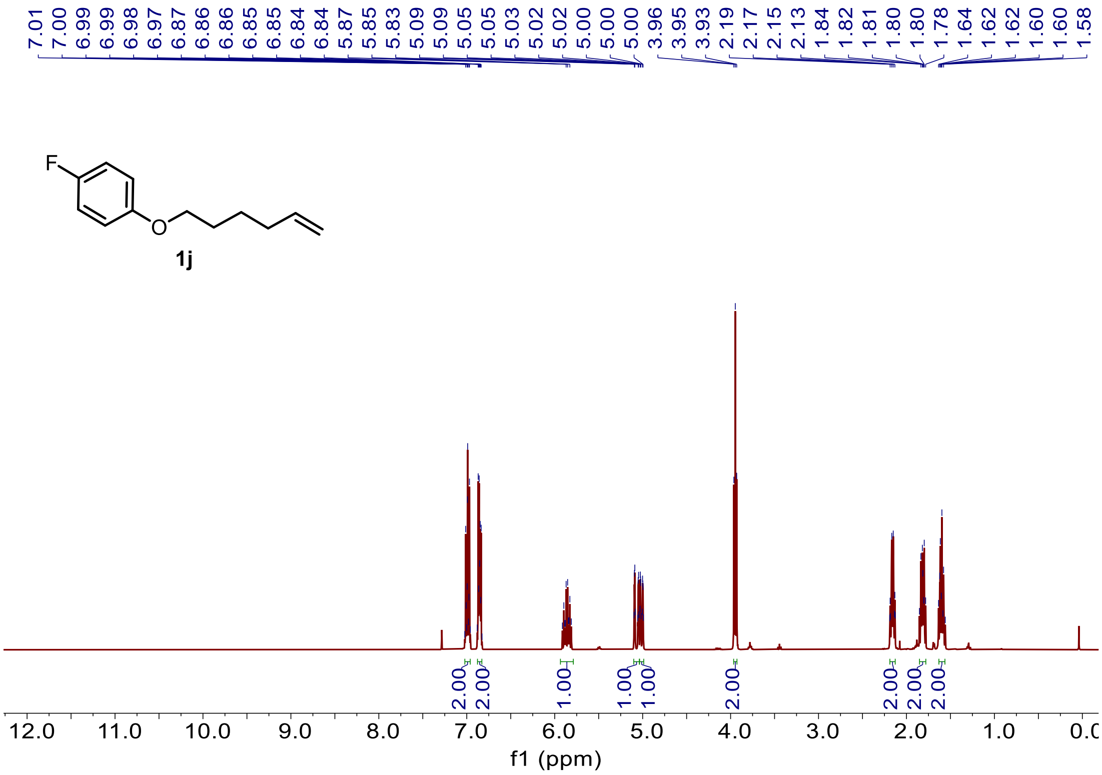

^{13}C NMR spectrum of compound **1j** in $CDCl_3$ (101 MHz).

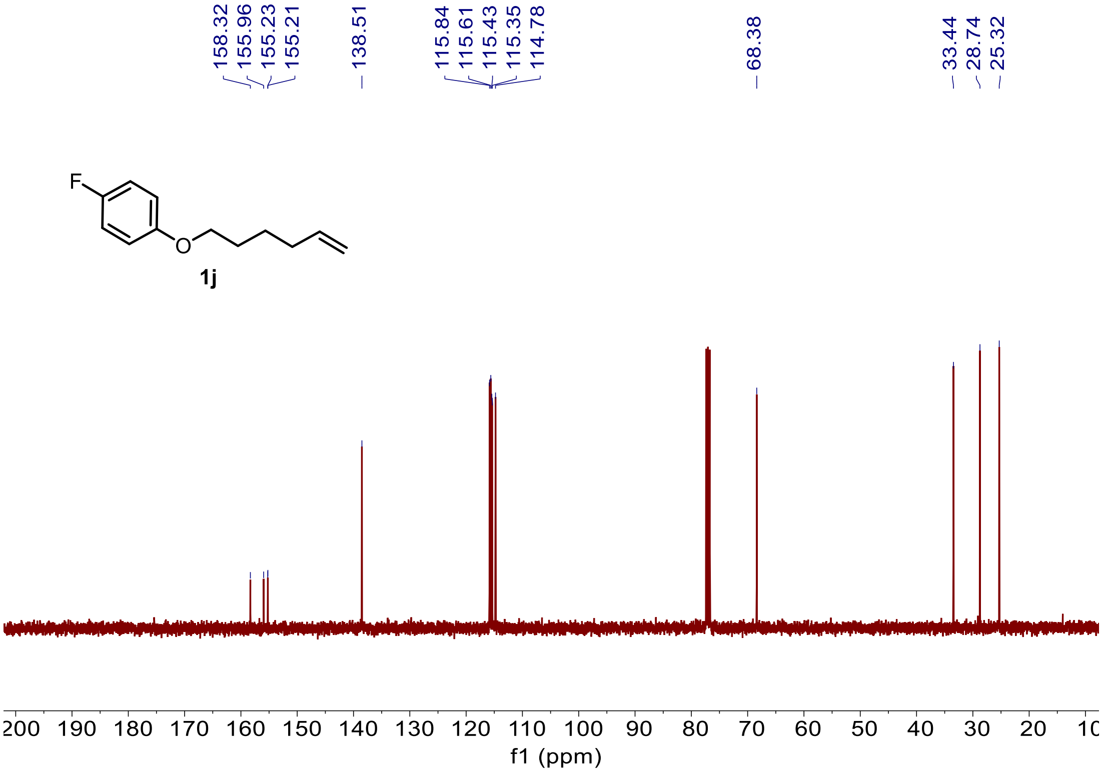

[13]F NMR spectrum of compound **1j** in $CDCl_3$ (376 MHz).

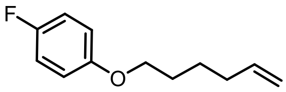

1j

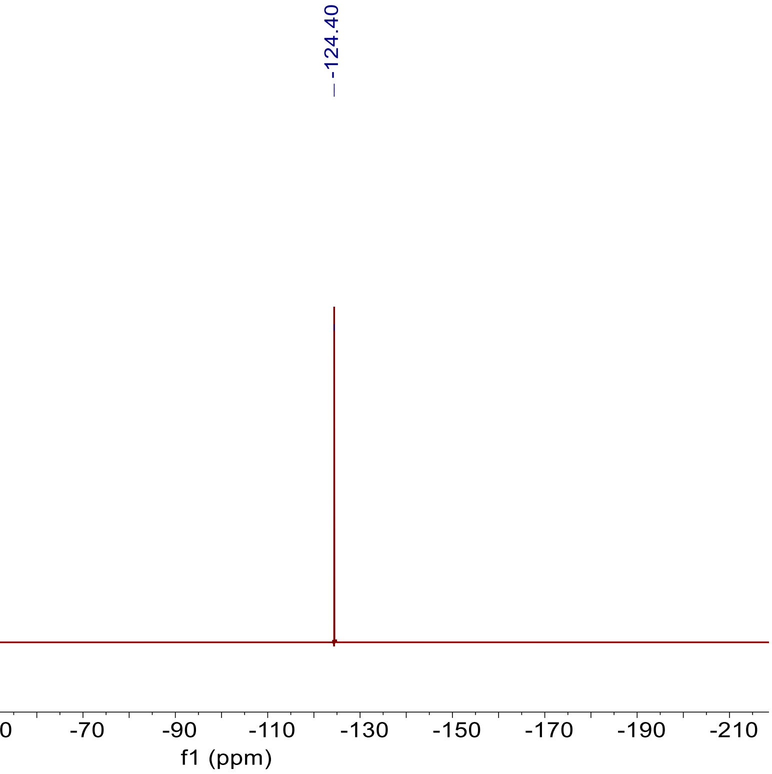

^{1}H NMR spectrum of compound **1l** in $CDCl_3$ (400 MHz).

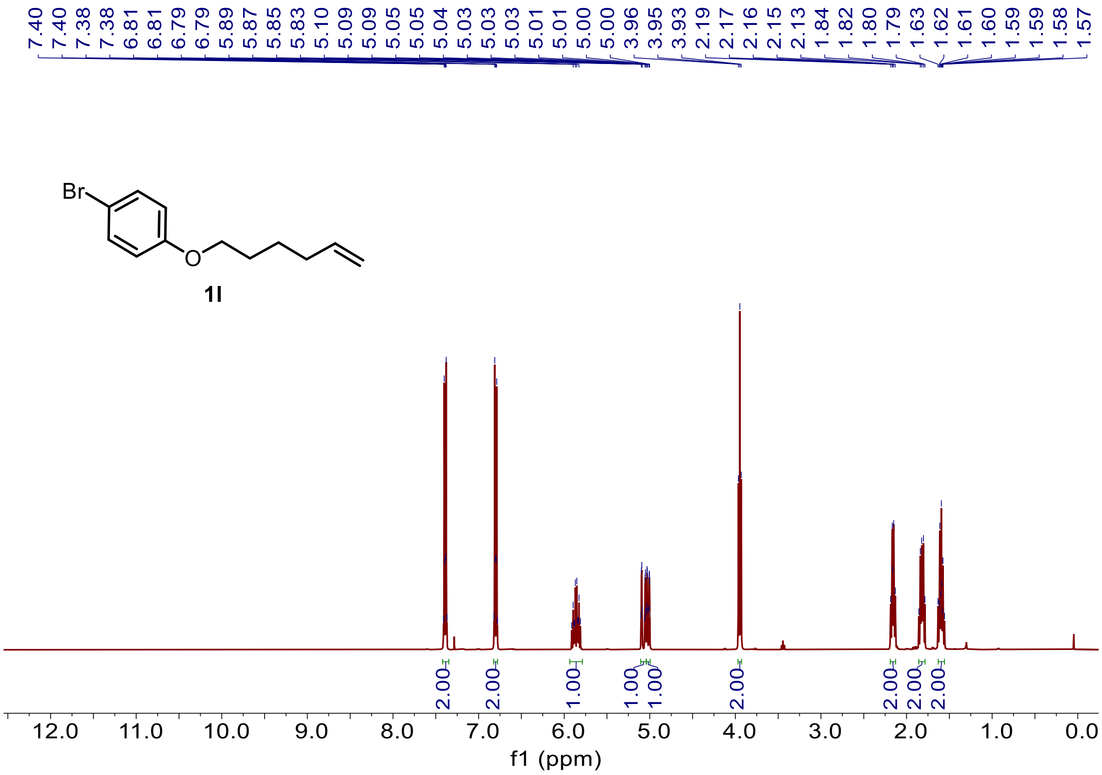

^{13}C NMR spectrum of compound **1l** in $CDCl_3$ (101 MHz).

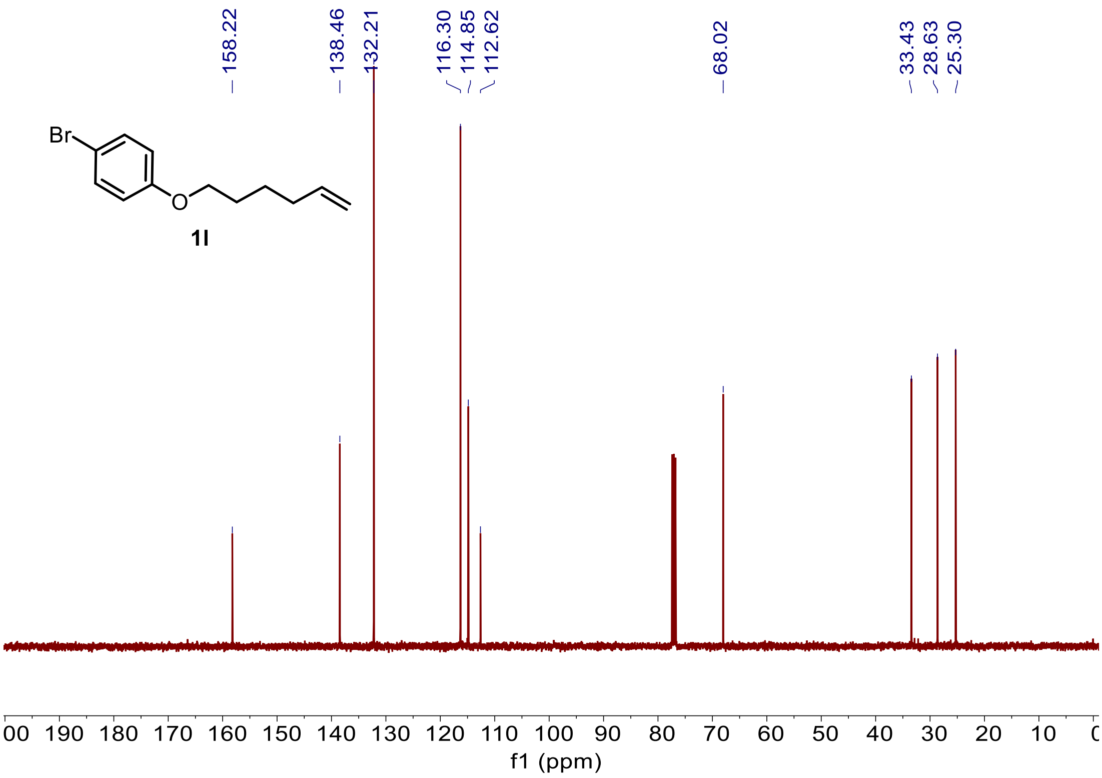

^{1}H NMR spectrum of compound **1m** in $CDCl_3$ (400 MHz).

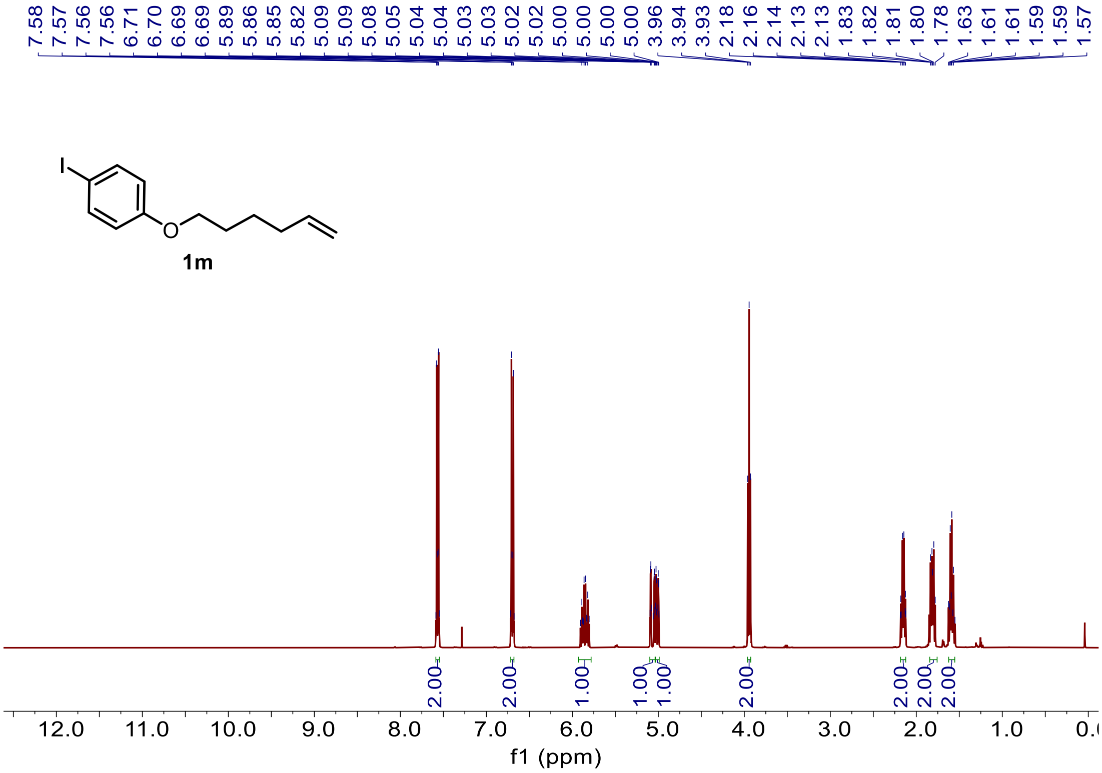

^{13}C NMR spectrum of compound **1m** in $CDCl_3$ (101 MHz).

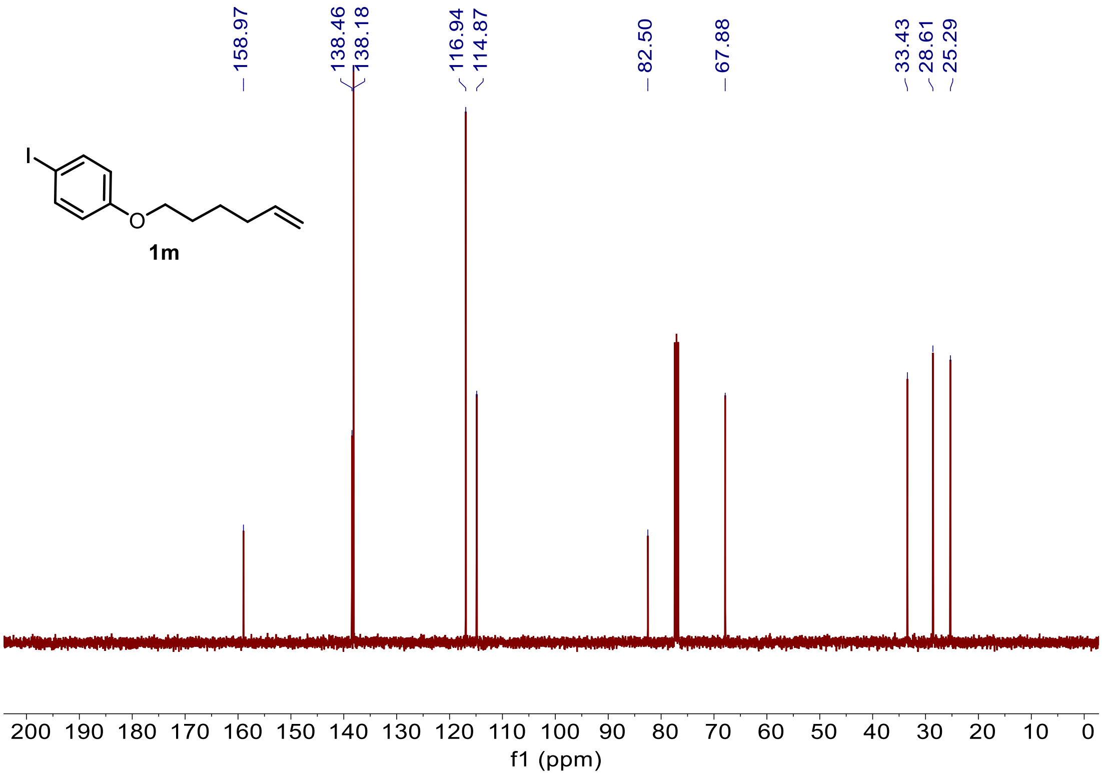

^{1}H NMR spectrum of compound **1q** in $CDCl_3$ (400 MHz).

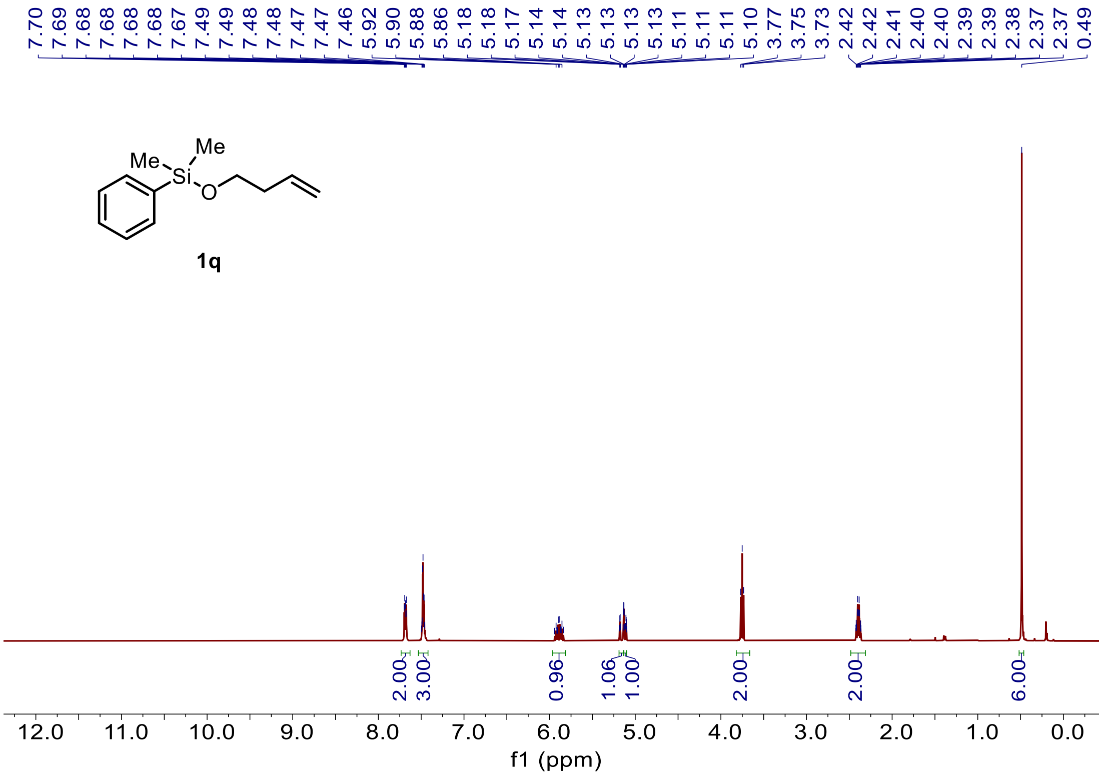

^{13}C NMR spectrum of compound **1q** in $CDCl_3$ (101 MHz).

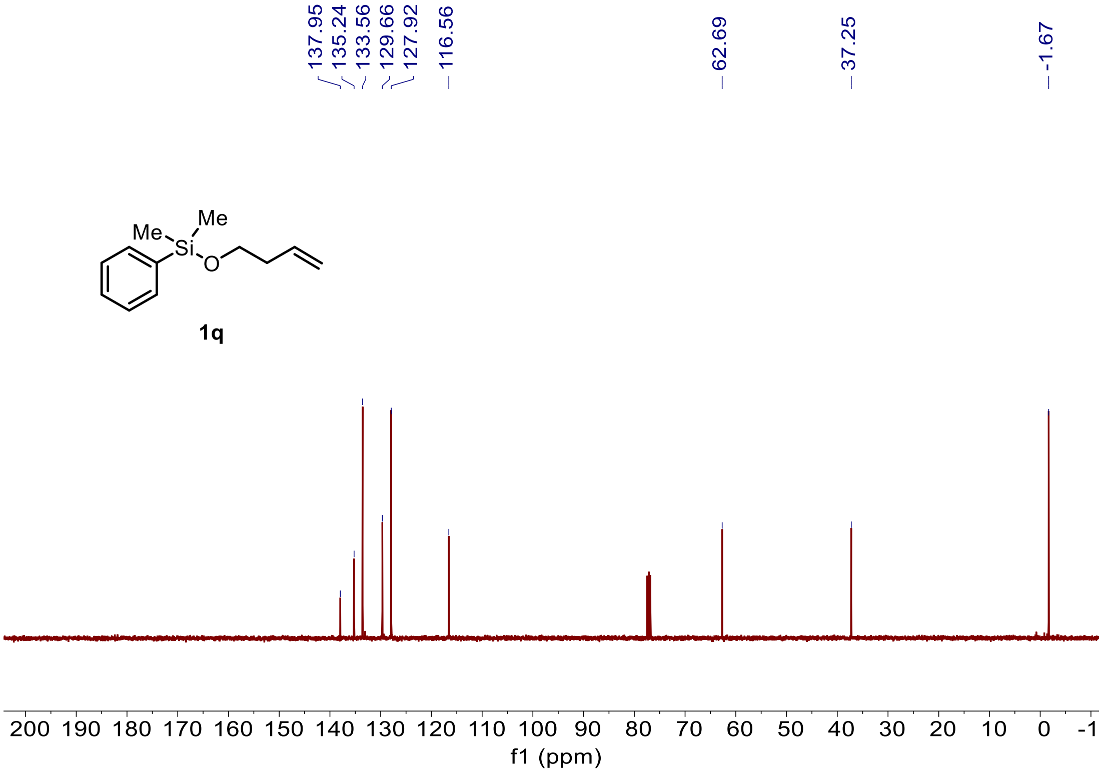

^{1}H NMR spectrum of compound **1s** in $CDCl_3$ (400 MHz).

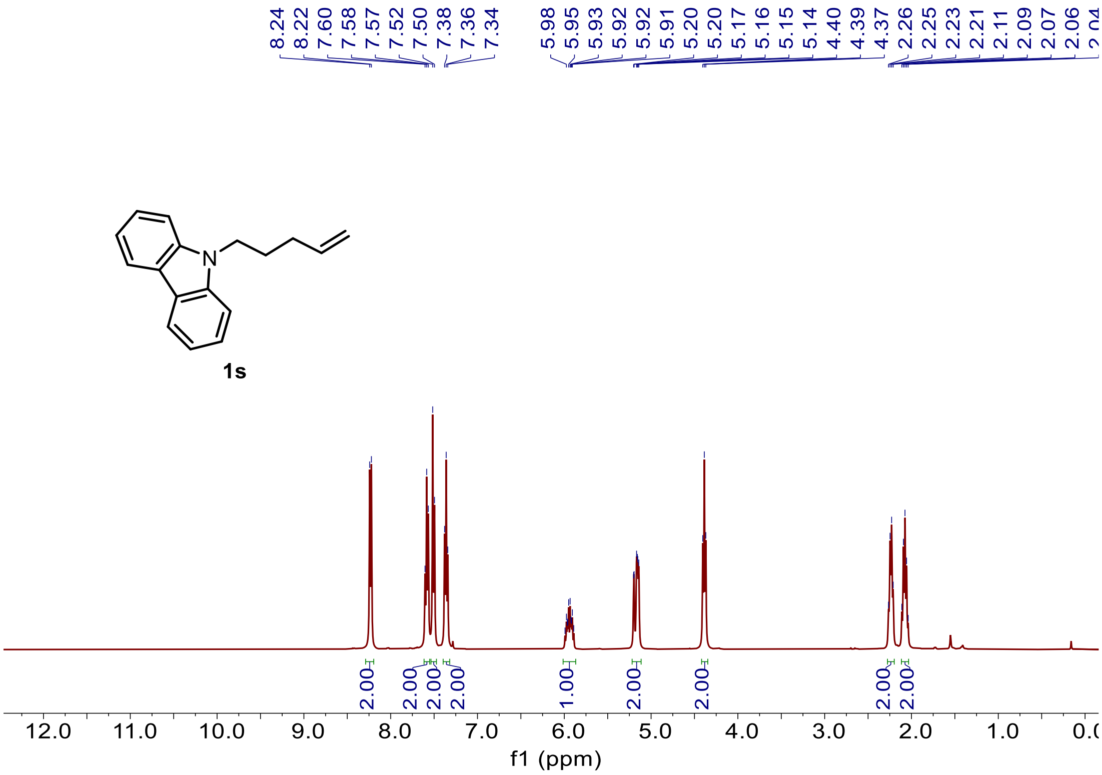

^{13}C NMR spectrum of compound **1s** in $CDCl_3$ (101 MHz).

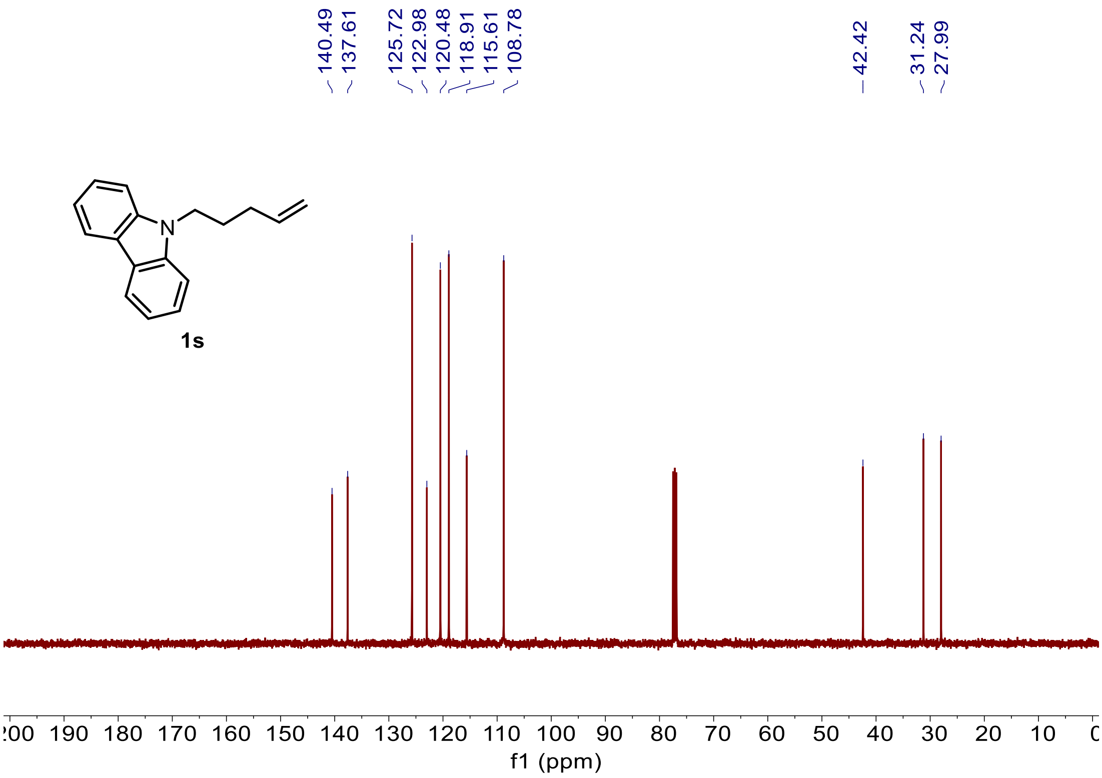

^{1}H NMR spectrum of compound **1t** in $CDCl_3$ (400 MHz).

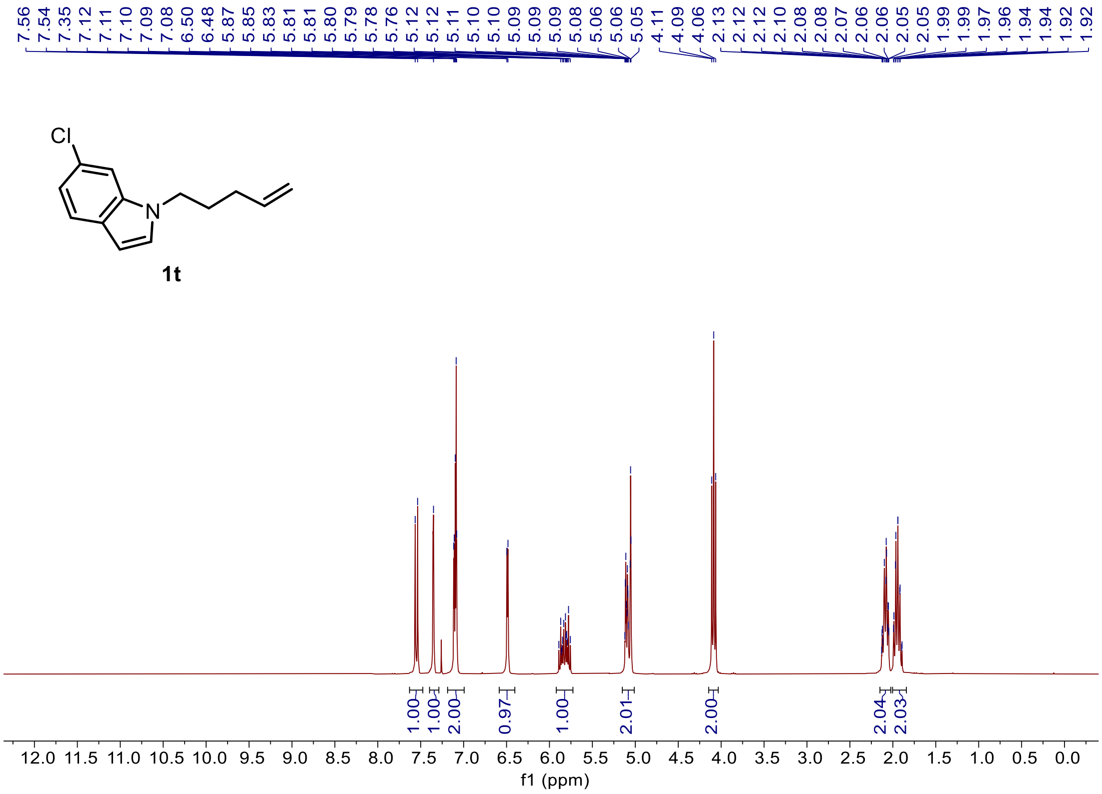

^{13}C NMR spectrum of compound **1t** in $CDCl_3$ (101 MHz).

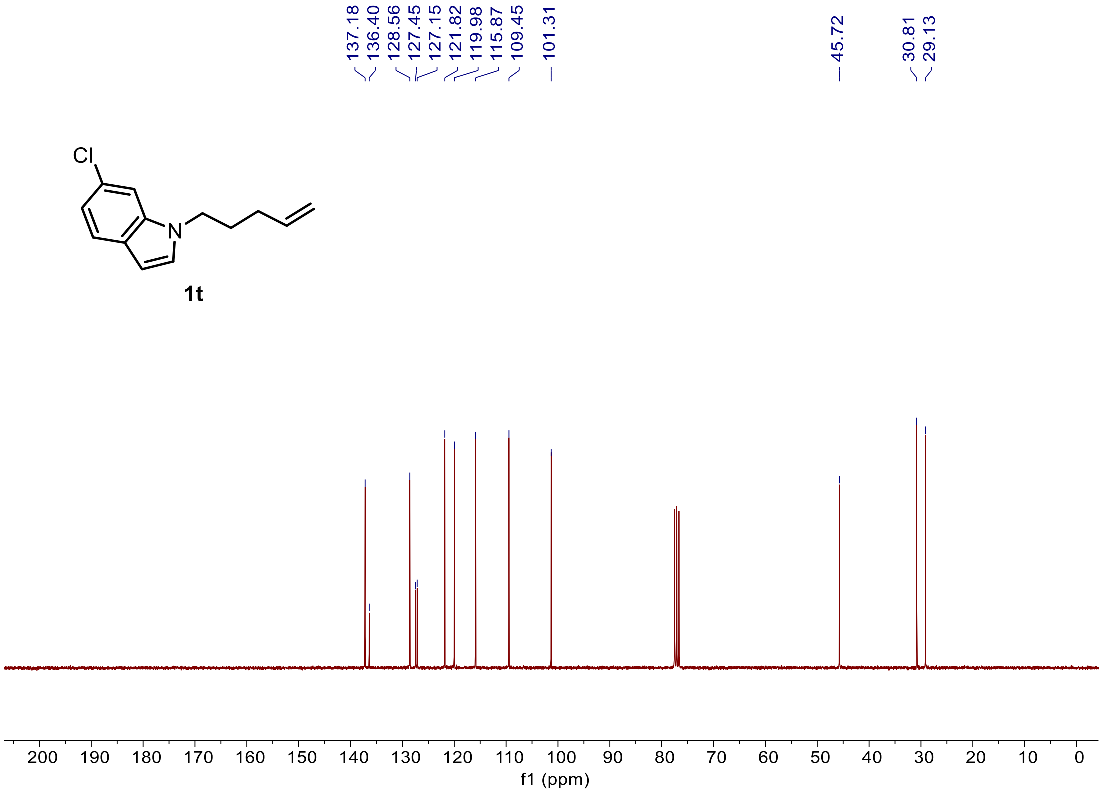

^{1}H NMR spectrum of compound **1u** in $CDCl_3$ (400 MHz).

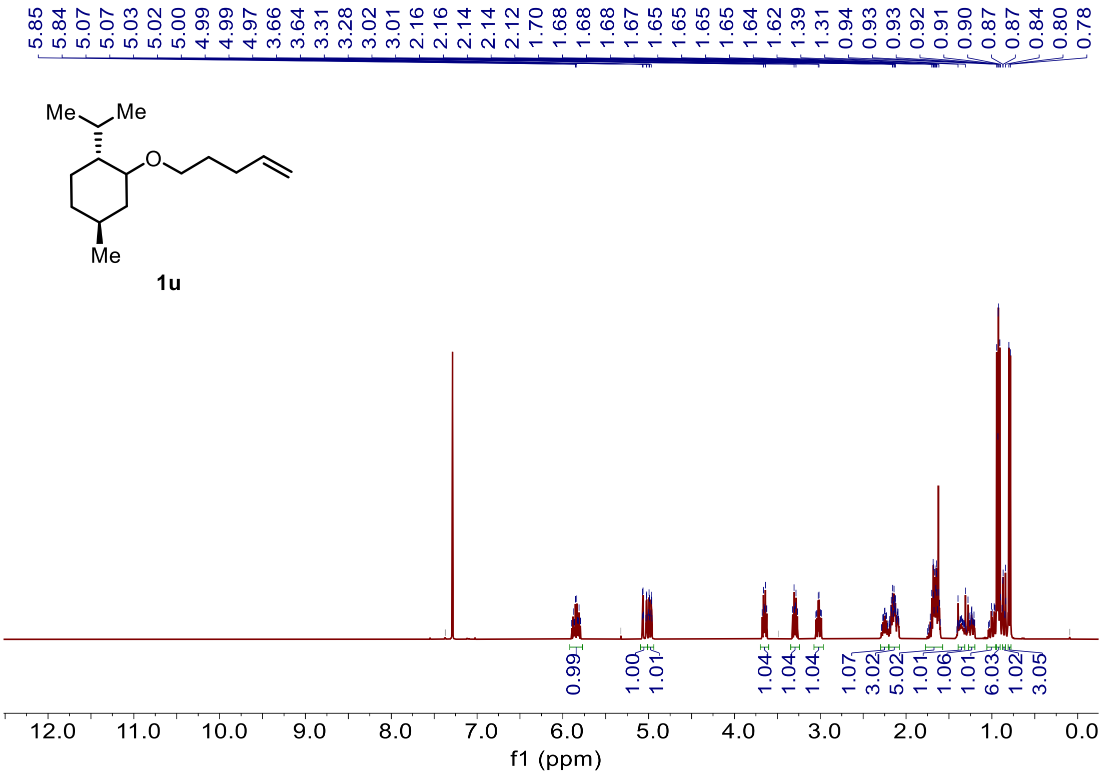

^{13}C NMR spectrum of compound **1u** in $CDCl_3$ (101 MHz).

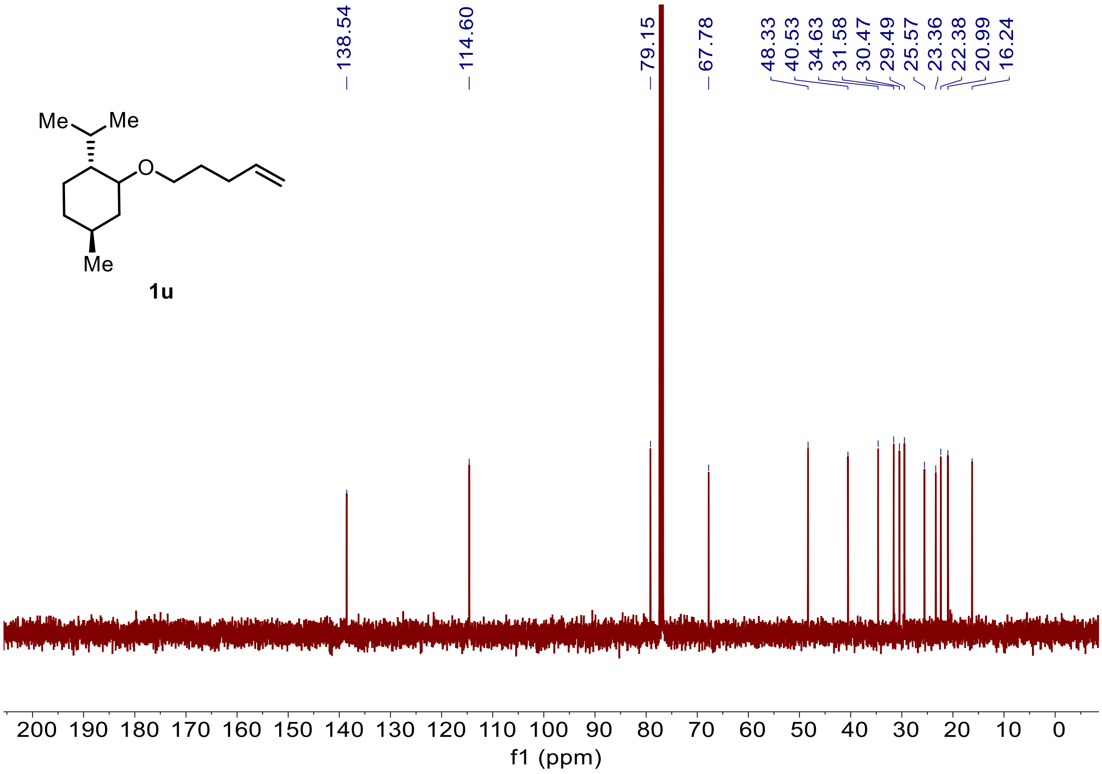

^{1}H NMR spectrum of compound **1v** in $CDCl_3$ (400 MHz).

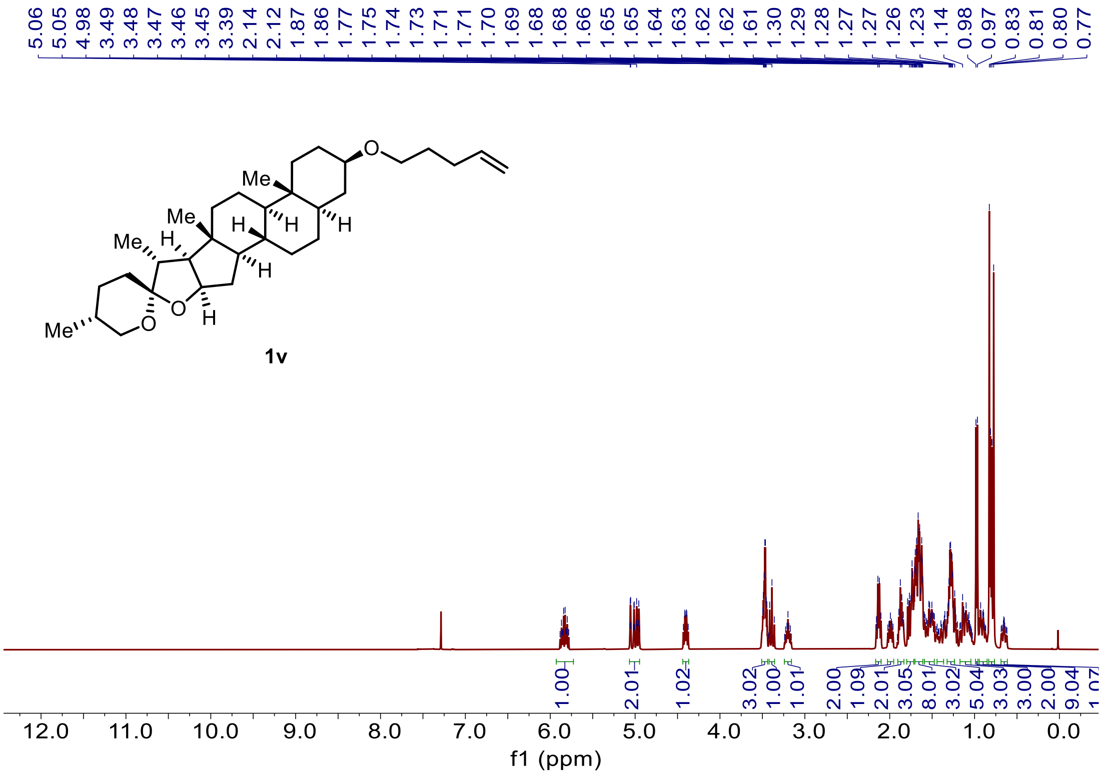

^{13}C NMR spectrum of compound **1v** in $CDCl_3$ (101 MHz).

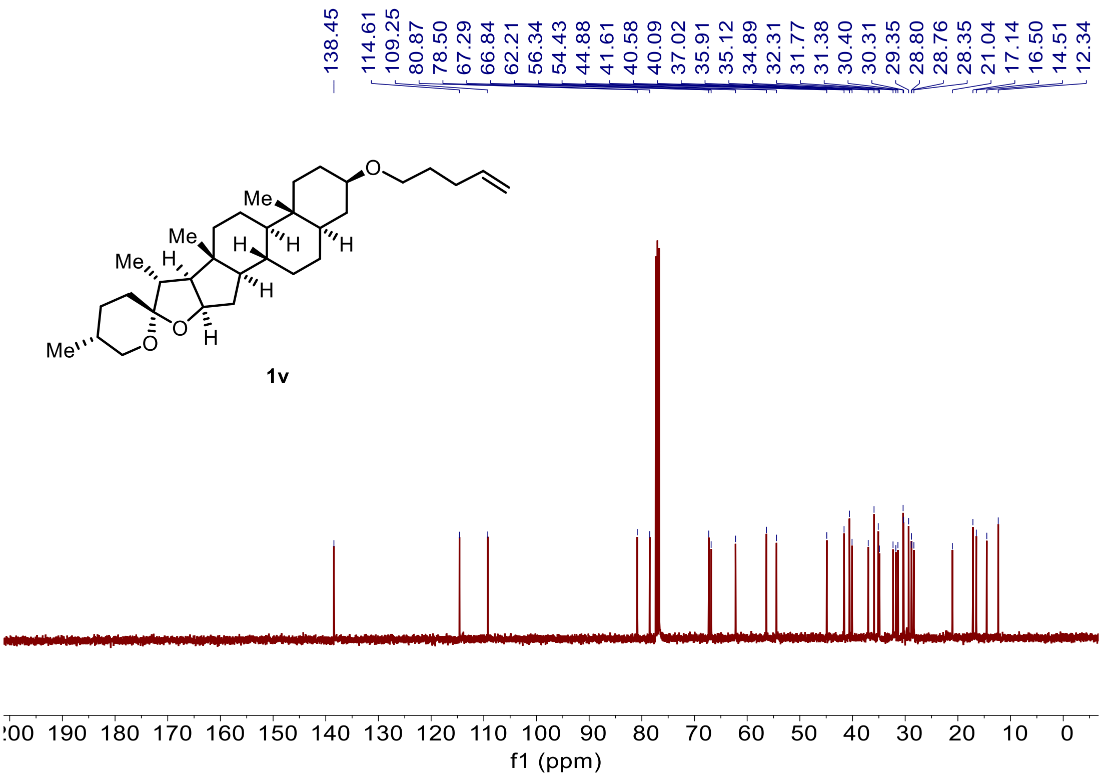

^{1}H NMR spectrum of compound **1w** in $CDCl_3$ (400 MHz).

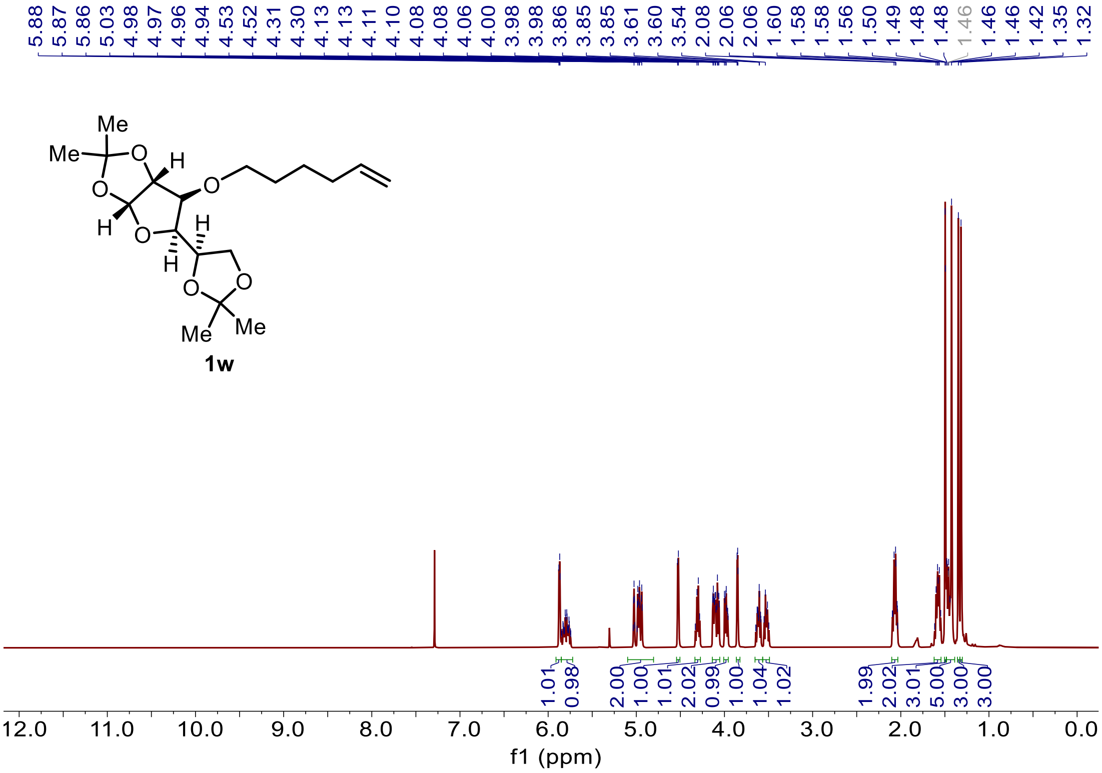

^{13}C NMR spectrum of compound **1w** in $CDCl_3$ (101 MHz).

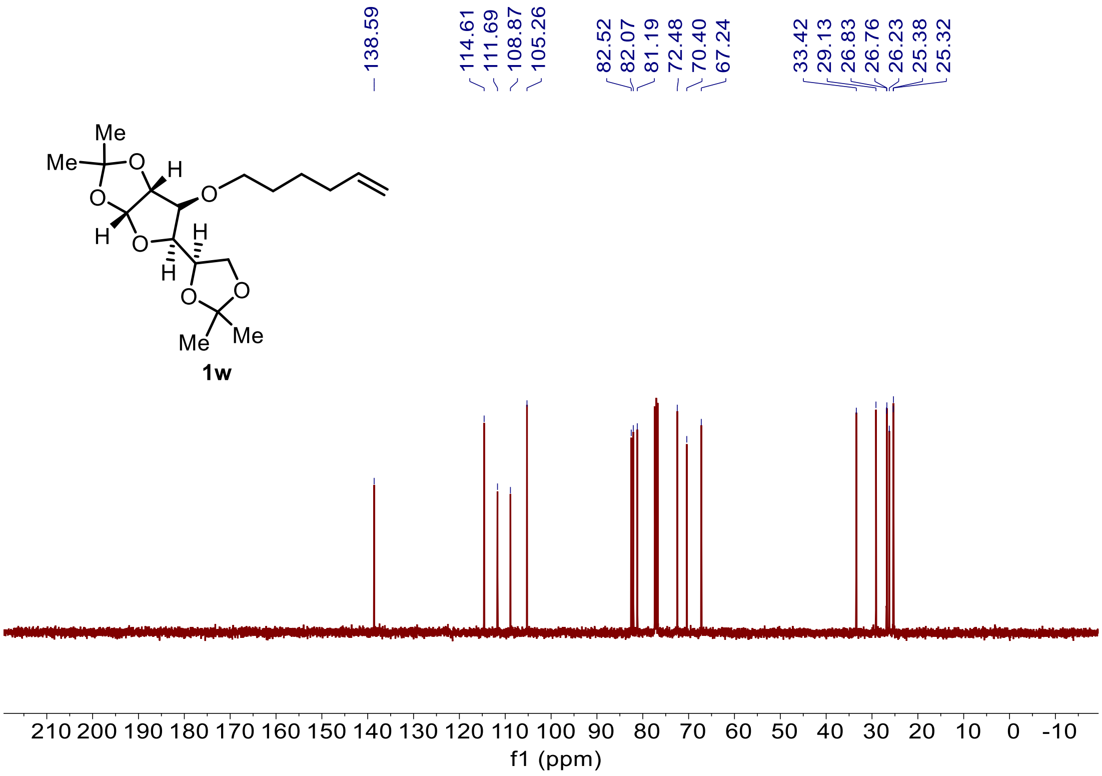

^{1}H NMR spectrum of compound **1x** in $CDCl_3$ (400 MHz).

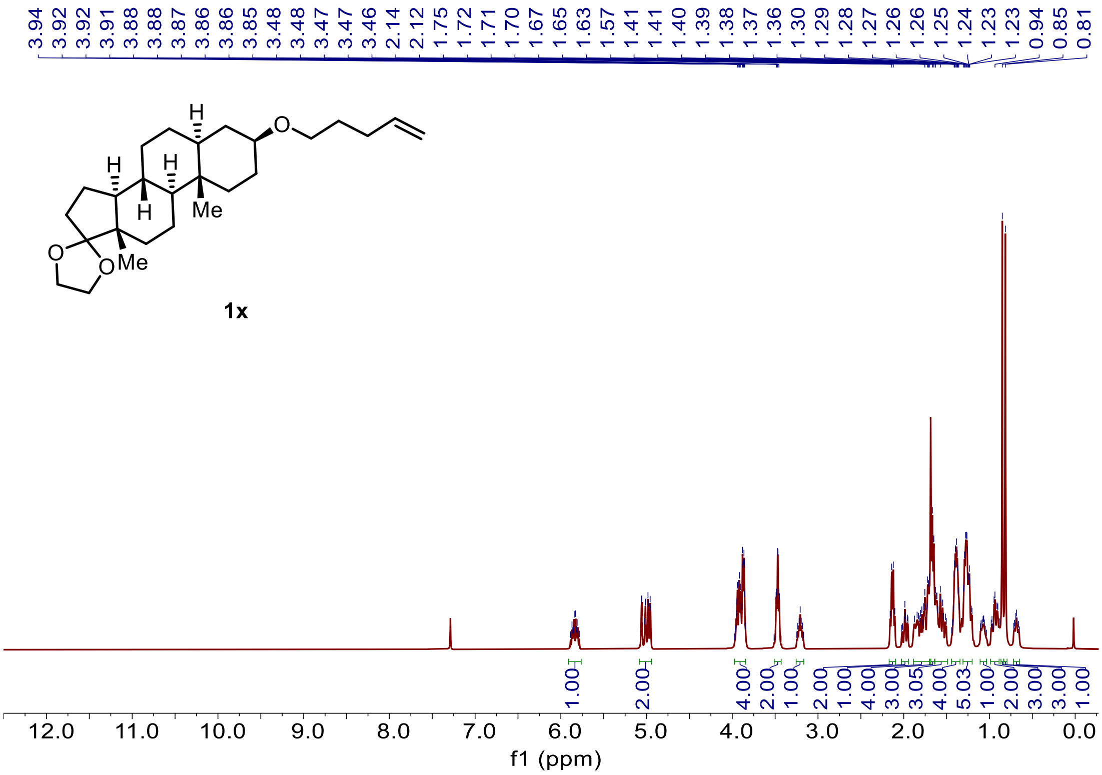

^{13}C NMR spectrum of compound **1x** in $CDCl_3$ (101 MHz).

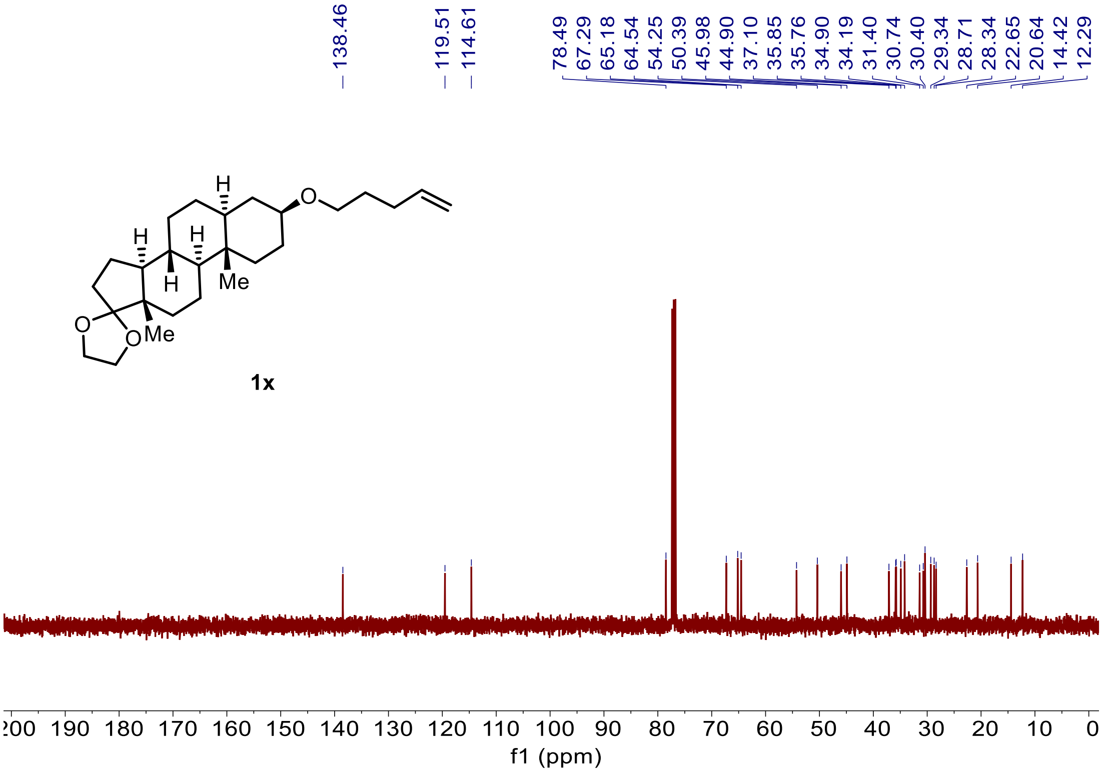

^{1}H NMR spectrum of compound **1y** in $CDCl_3$ (400 MHz).

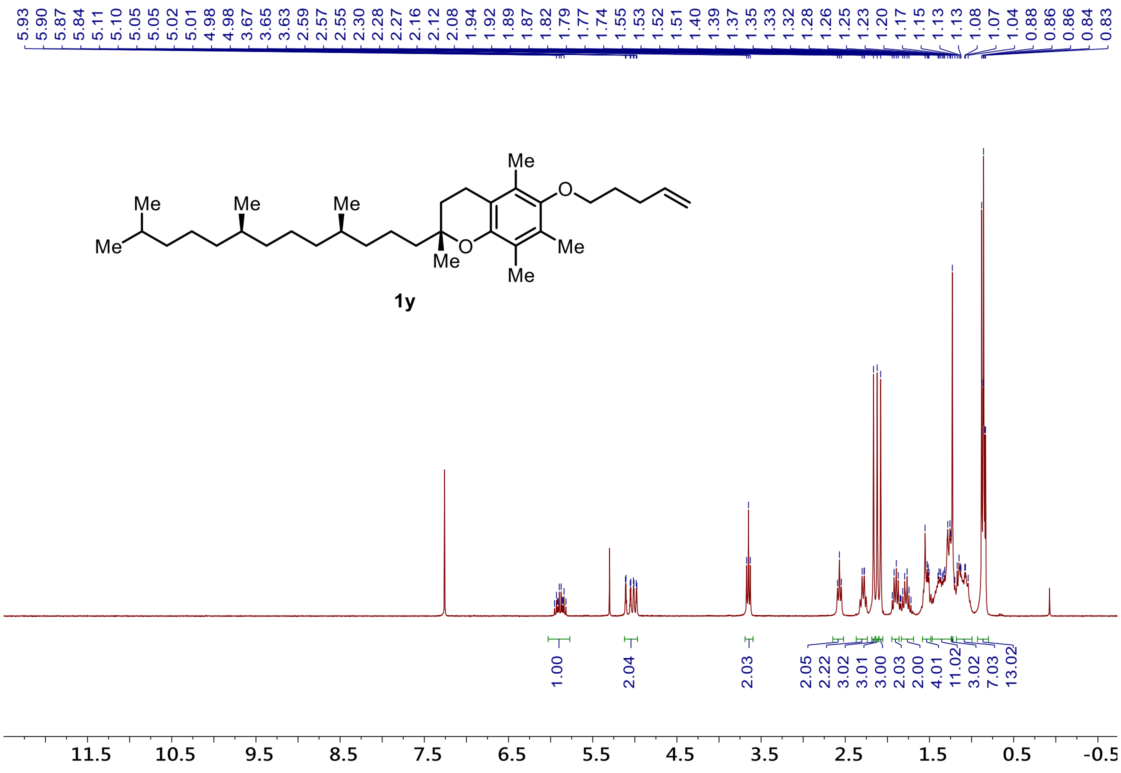

^{13}C NMR spectrum of compound **1y** in $CDCl_3$ (101 MHz).

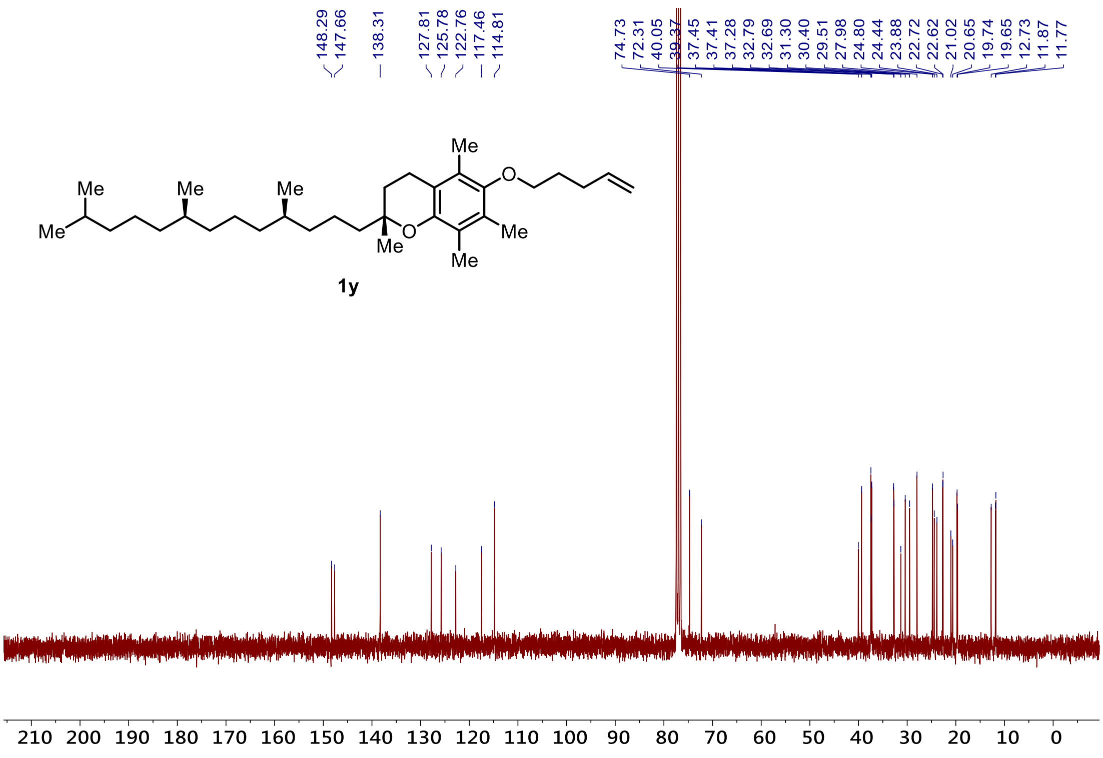

^{1}H NMR spectrum of compound **1z** in $CDCl_3$ (400 MHz).

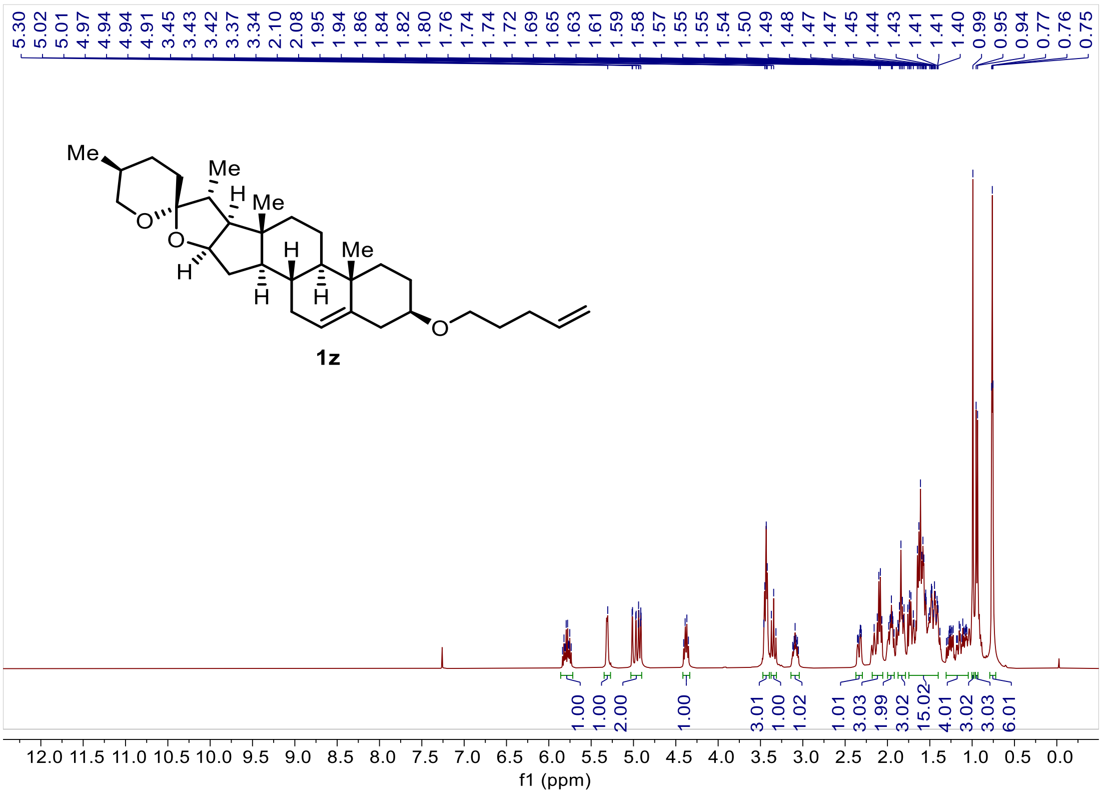

^{13}C NMR spectrum of compound **1z** in $CDCl_3$ (101 MHz).

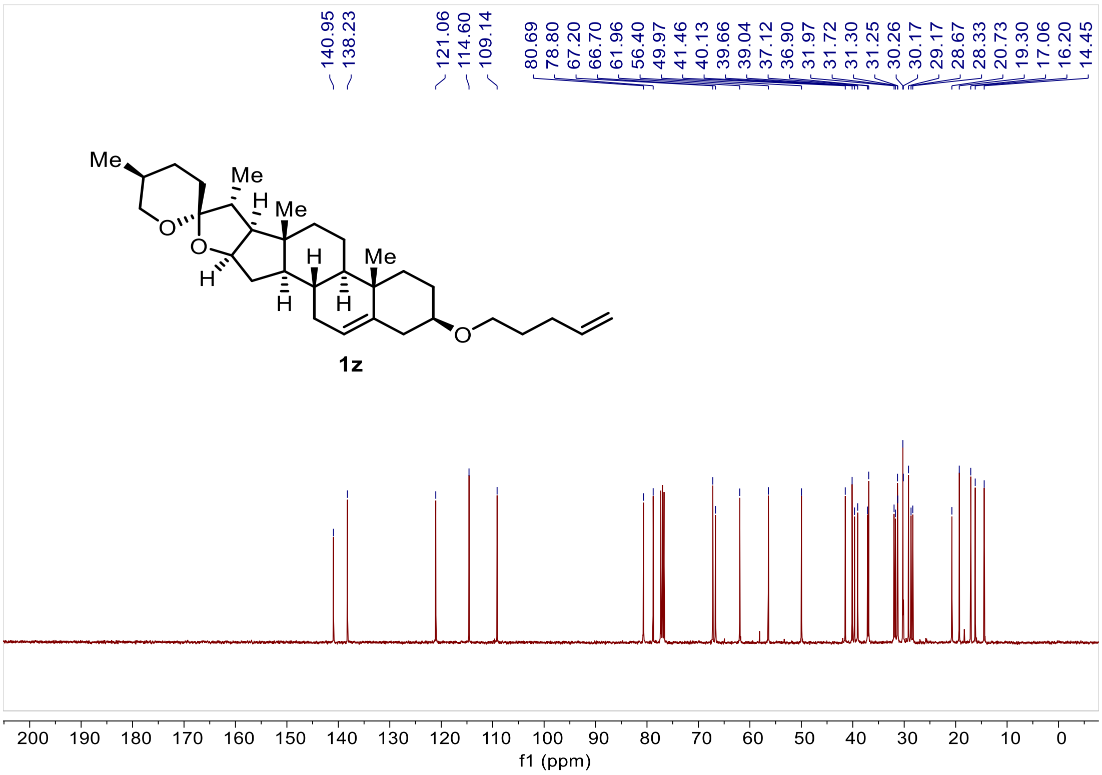

^{1}H NMR spectrum of compound **3a** in $CDCl_3$ (300 MHz).

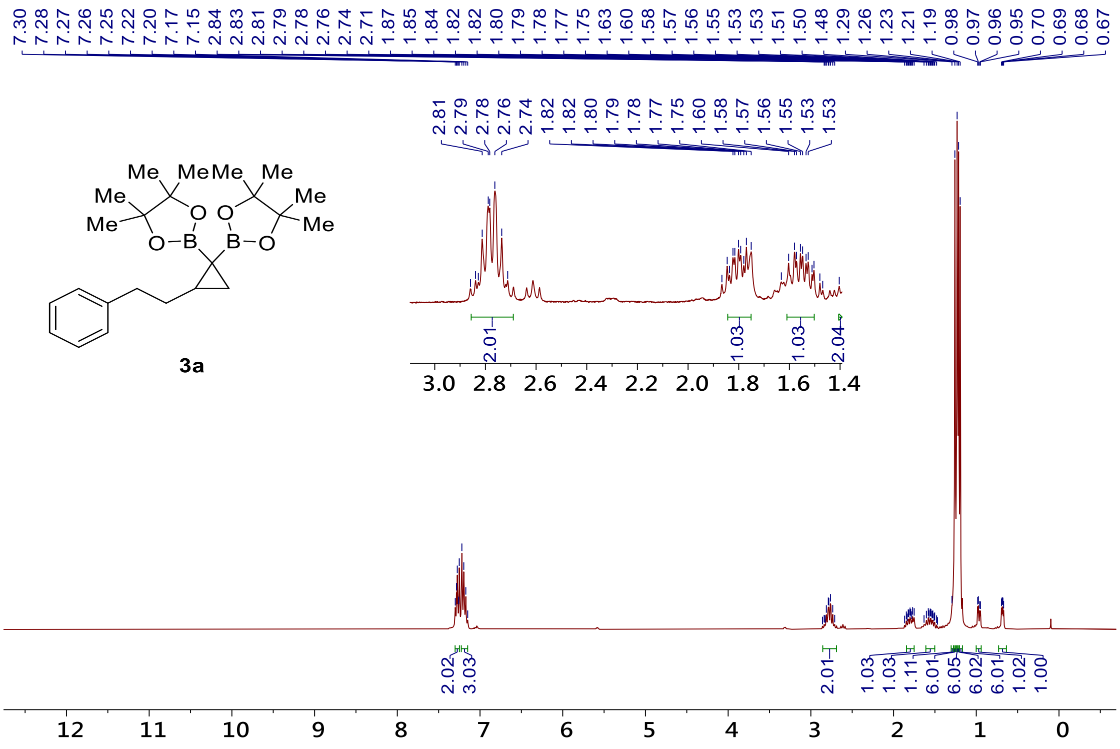

^{13}C NMR spectrum of compound **1z** in $CDCl_3$ (75 MHz).

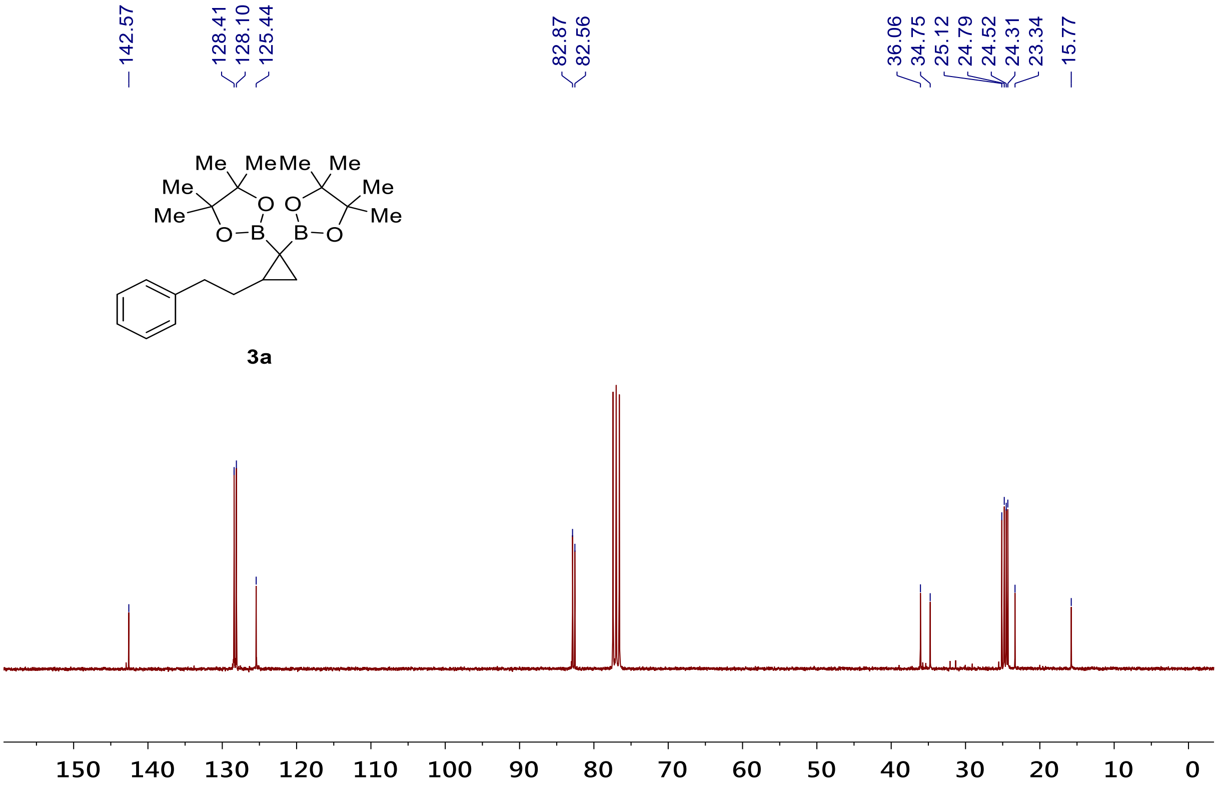

^{11}B NMR spectrum of compound **3a** in $CDCl_3$ (96 MHz).

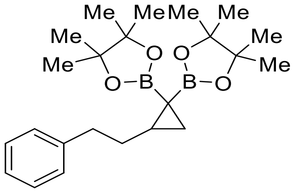

3a

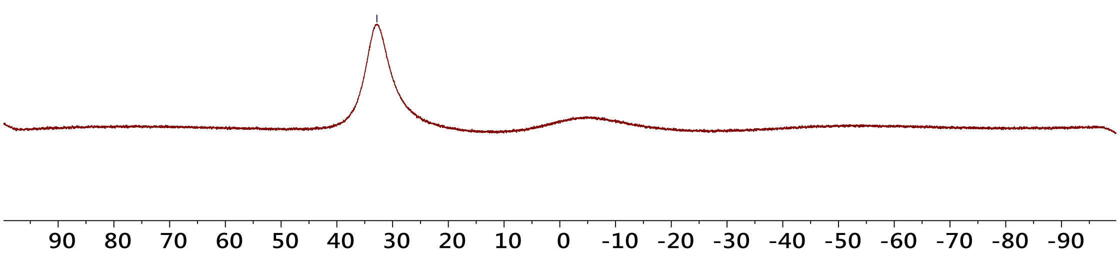

^{1}H NMR spectrum of compound **3b** in $CDCl_3$ (400 MHz).

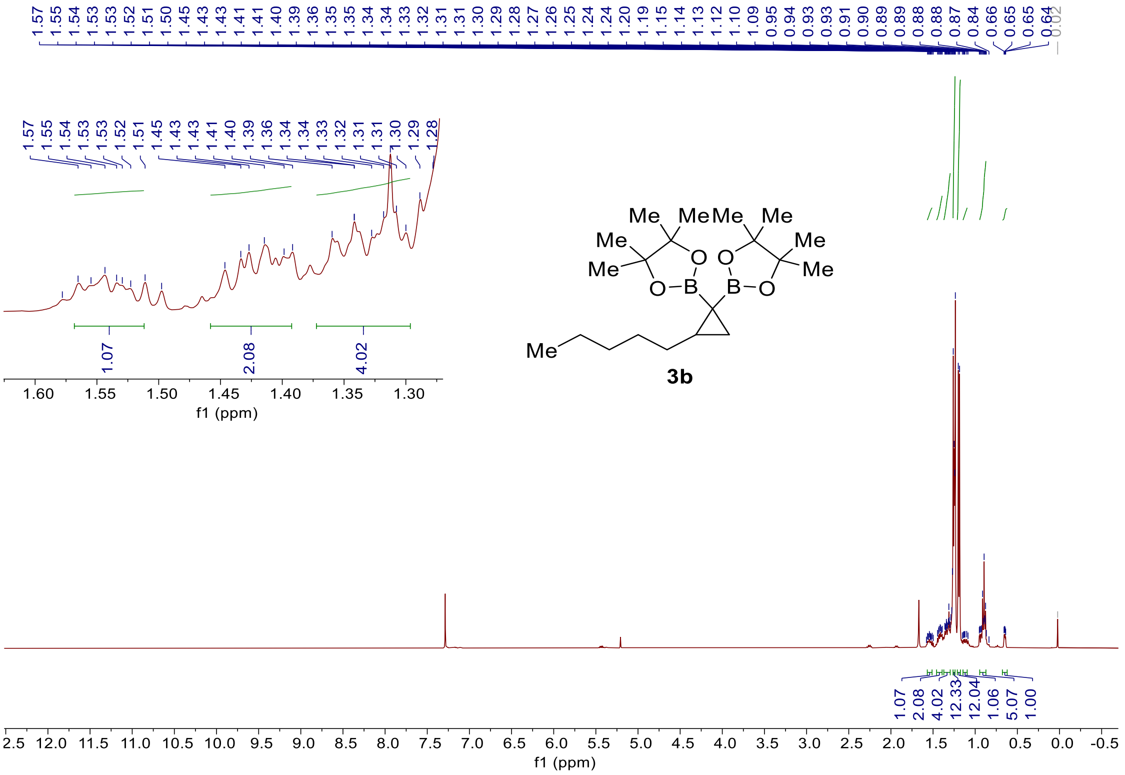

^{13}C NMR spectrum of compound **1z** in $CDCl_3$ (101 MHz).

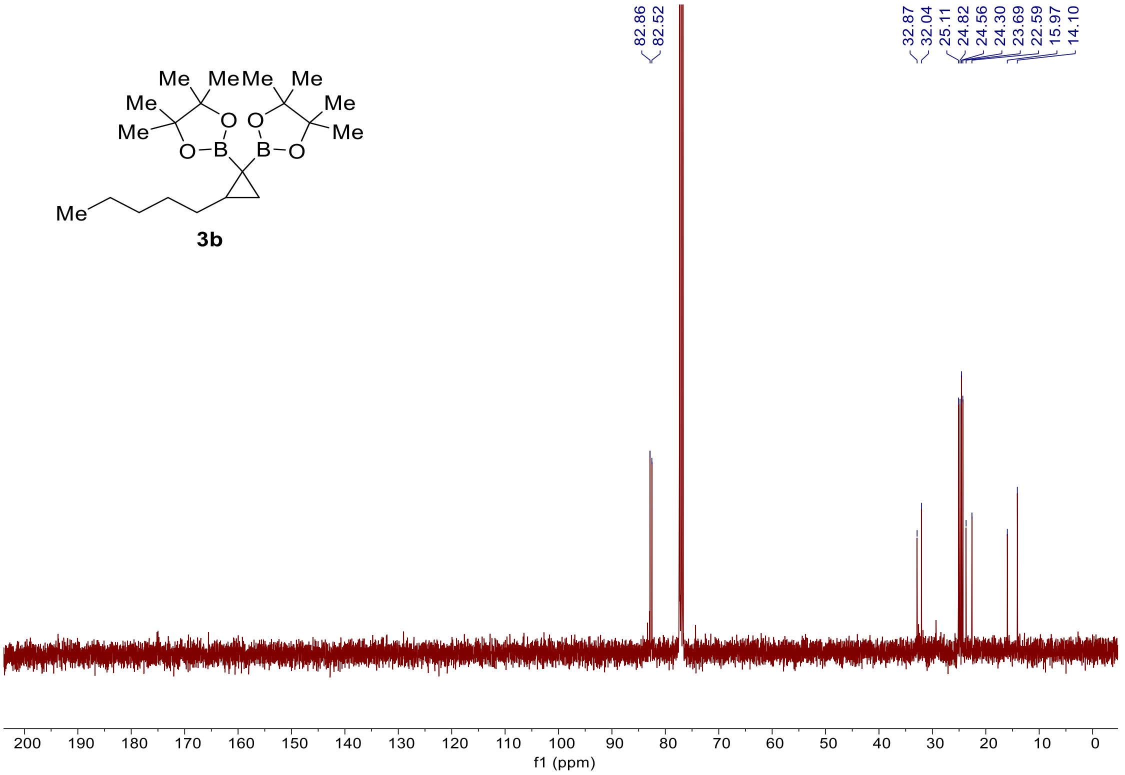

^{11}B NMR spectrum of compound **3b** in $CDCl_3$ (128 MHz).

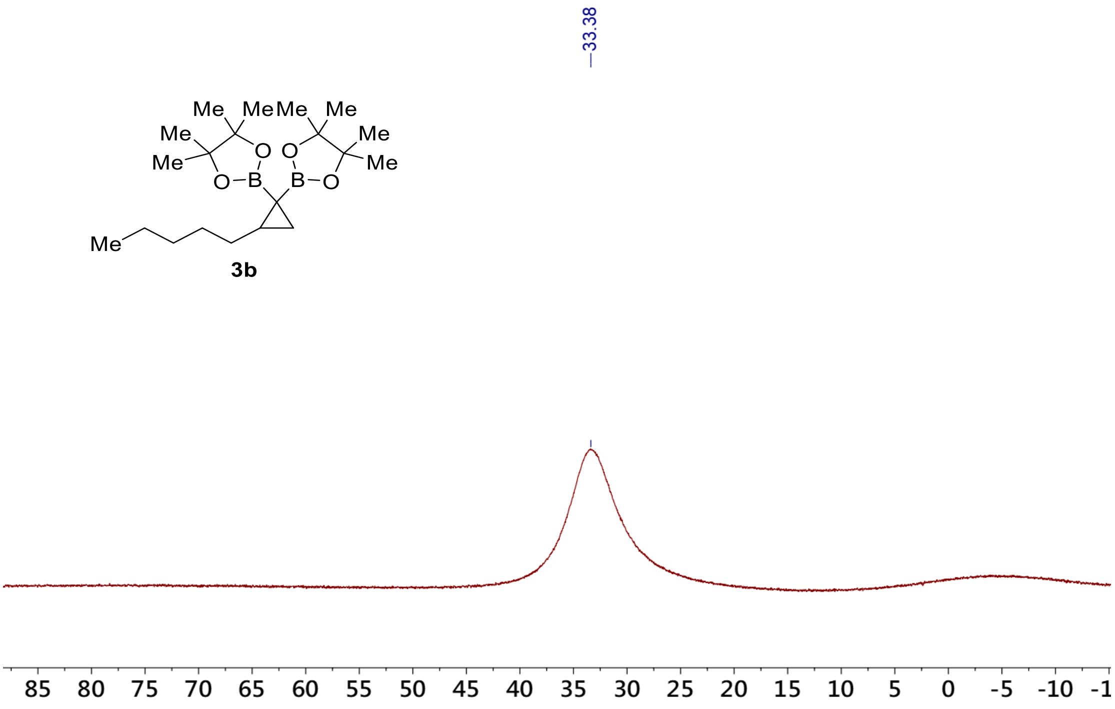

^{1}H NMR spectrum of compound **3c** in $CDCl_3$ (400 MHz).

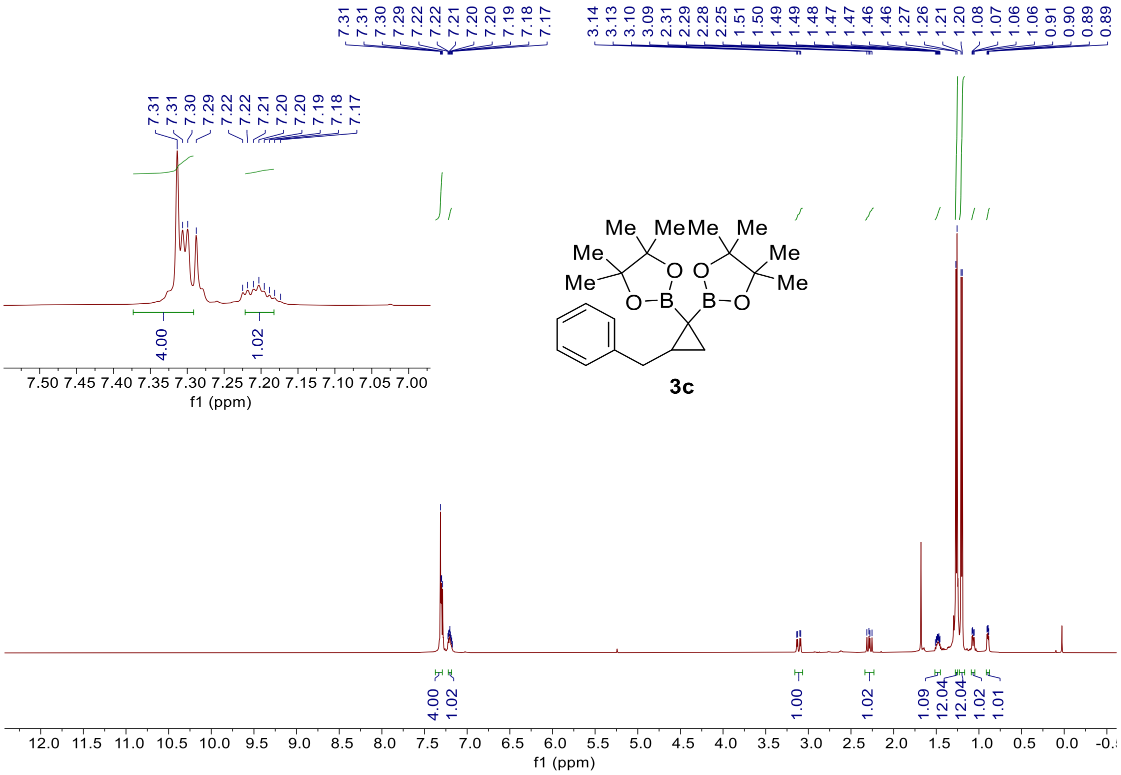

^{13}C NMR spectrum of compound **3c** in $CDCl_3$ (101 MHz).

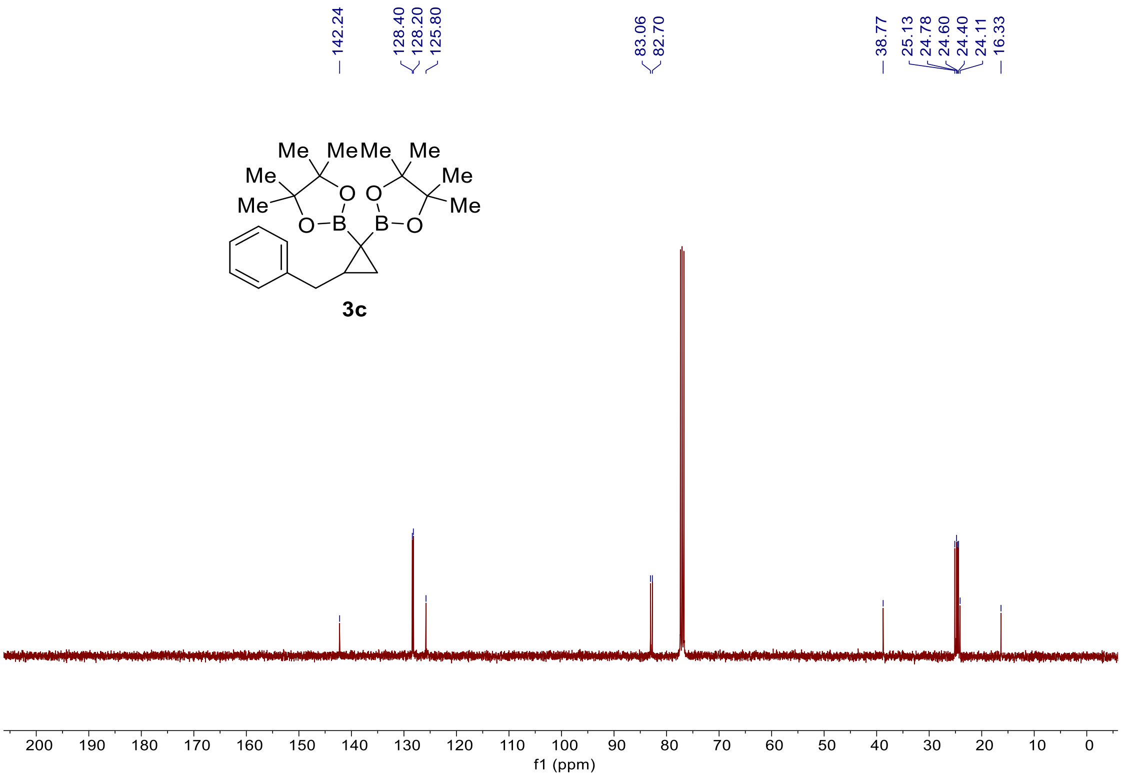

^{11}B NMR spectrum of compound **3c** in $CDCl_3$ (128 MHz).

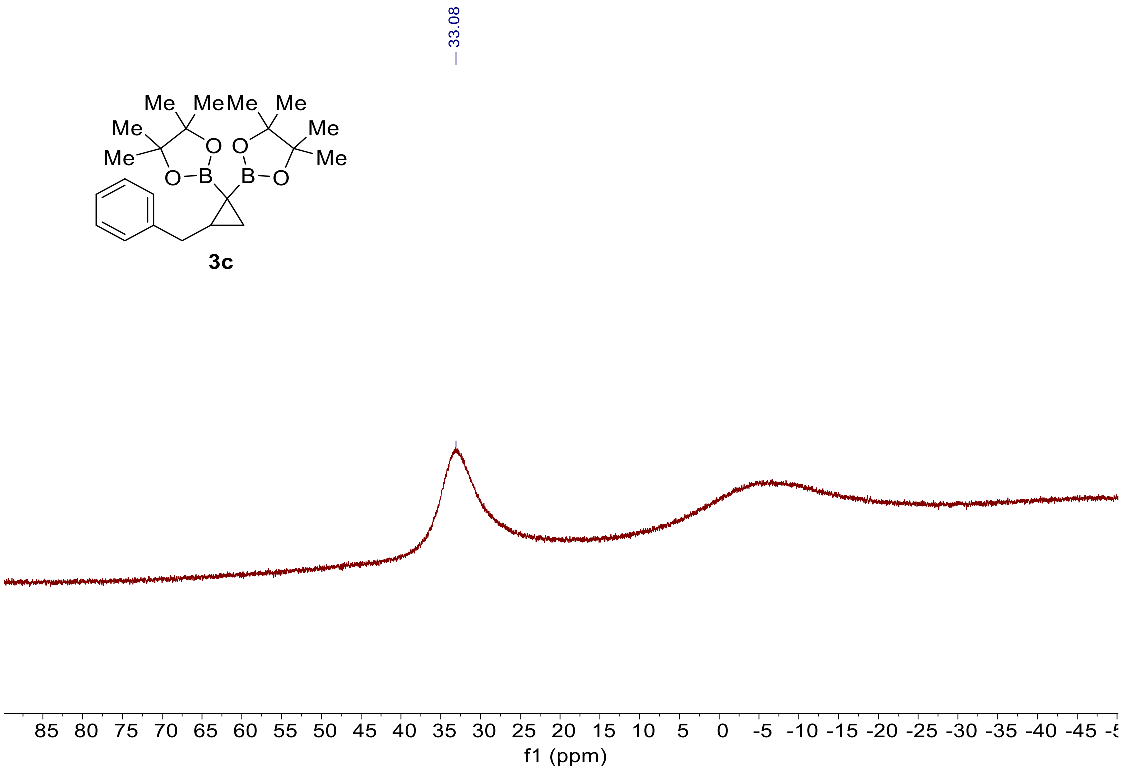

^{1}H NMR spectrum of compound **3d** in $CDCl_3$ (400 MHz).

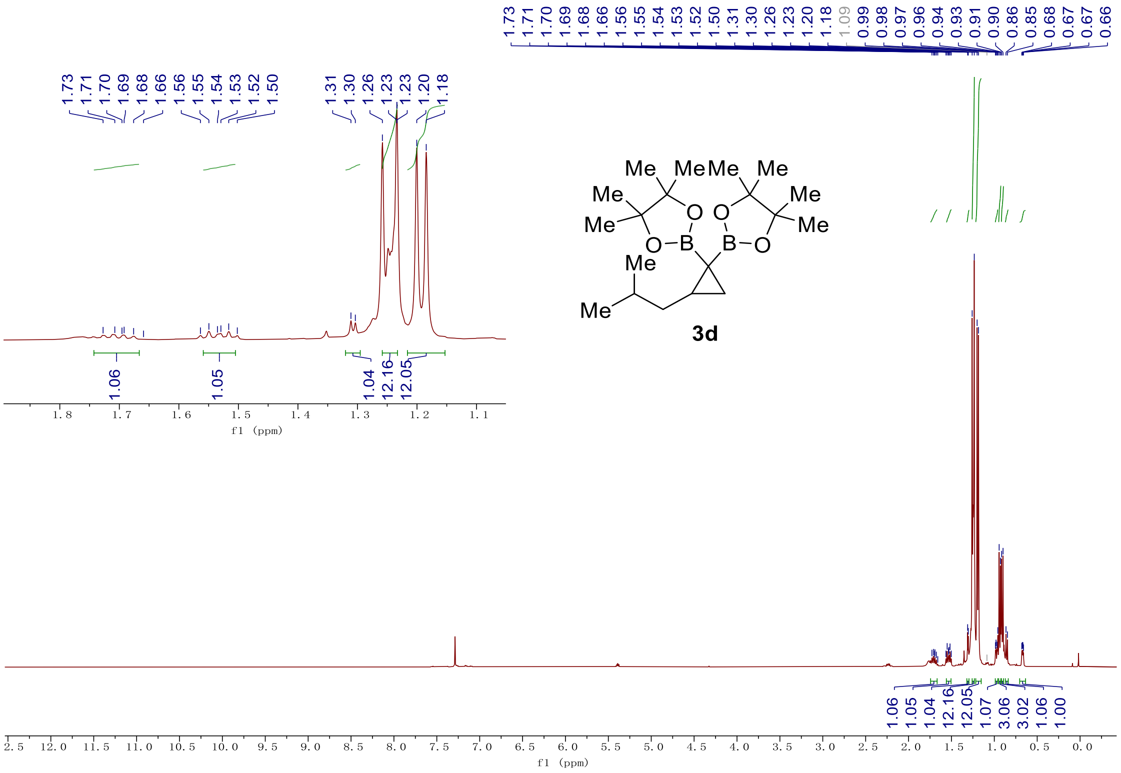

^{13}C NMR spectrum of compound **3d** in $CDCl_3$ (101 MHz).

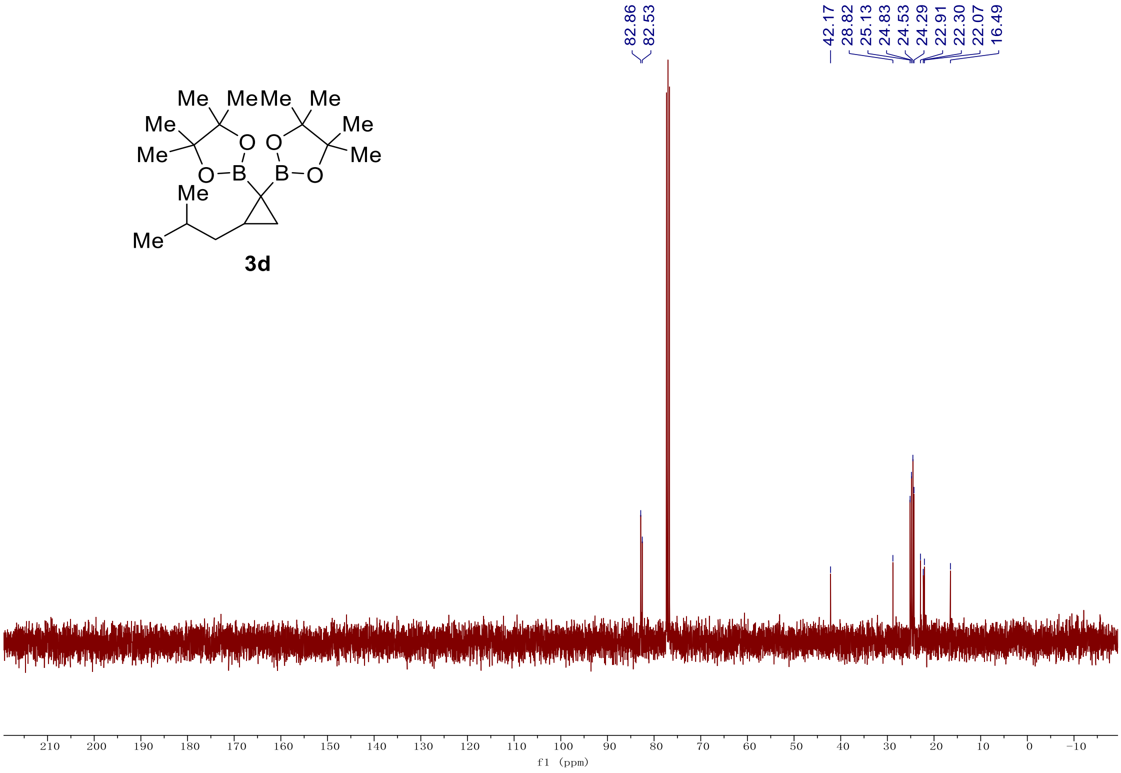

^{11}B NMR spectrum of compound **3d** in $CDCl_3$ (128 MHz).

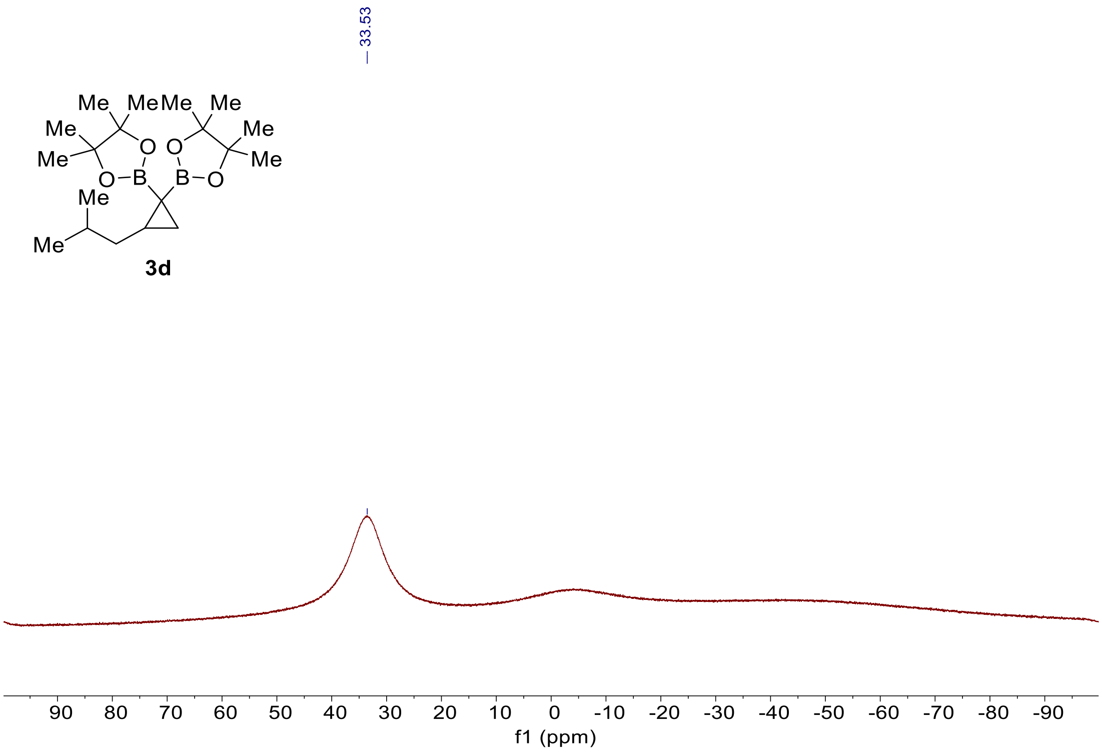

^{1}H NMR spectrum of compound **3e** in $CDCl_3$ (400 MHz).

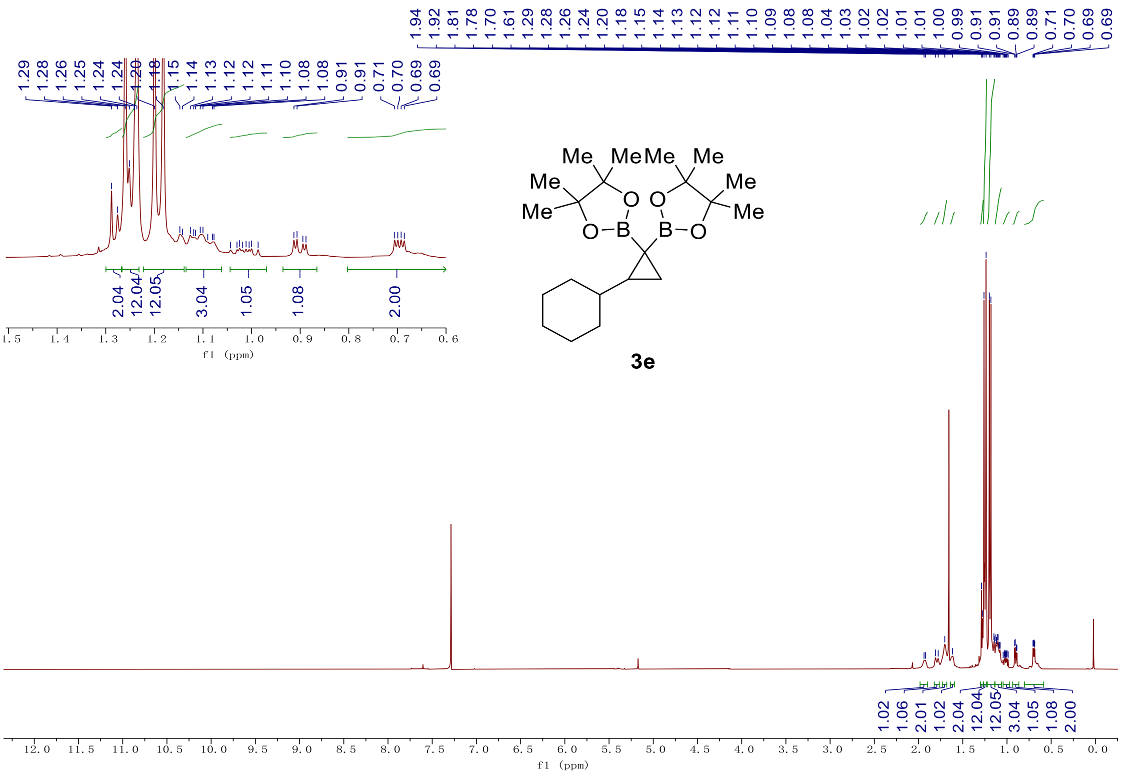

^{13}C NMR spectrum of compound **3e** in $CDCl_3$ (101 MHz).

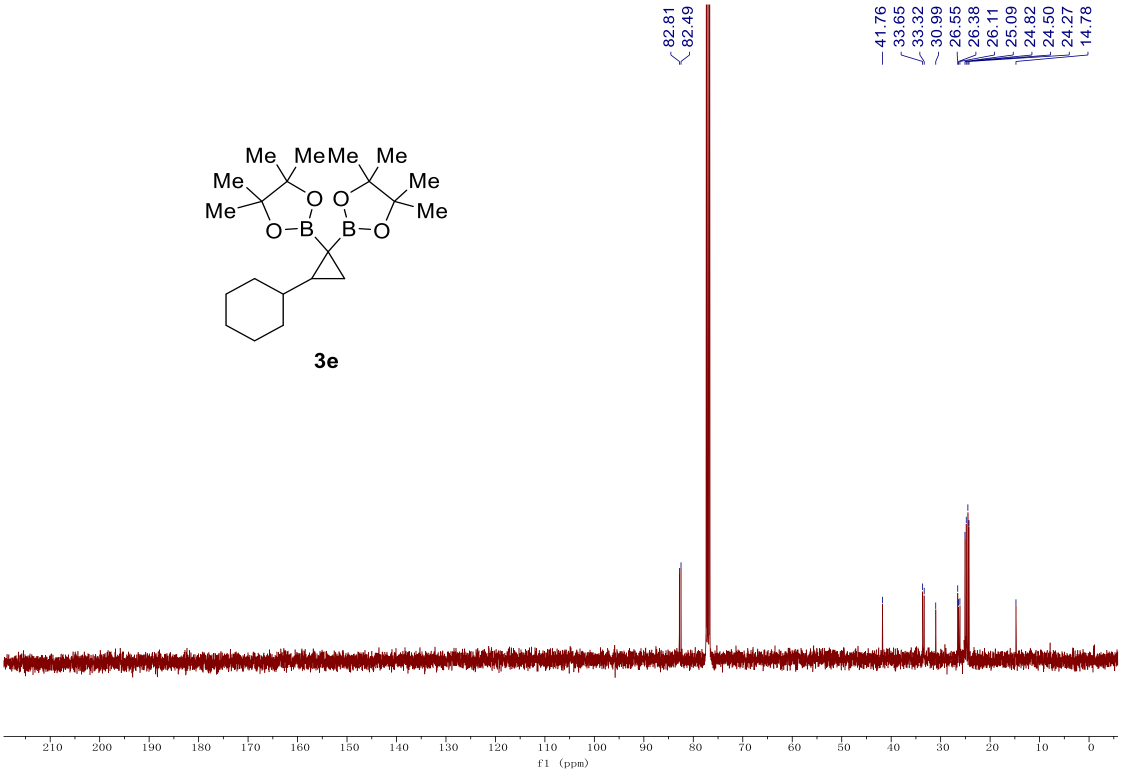

^{11}B NMR spectrum of compound **3e** in $CDCl_3$ (128 MHz).

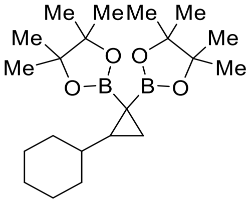

3e

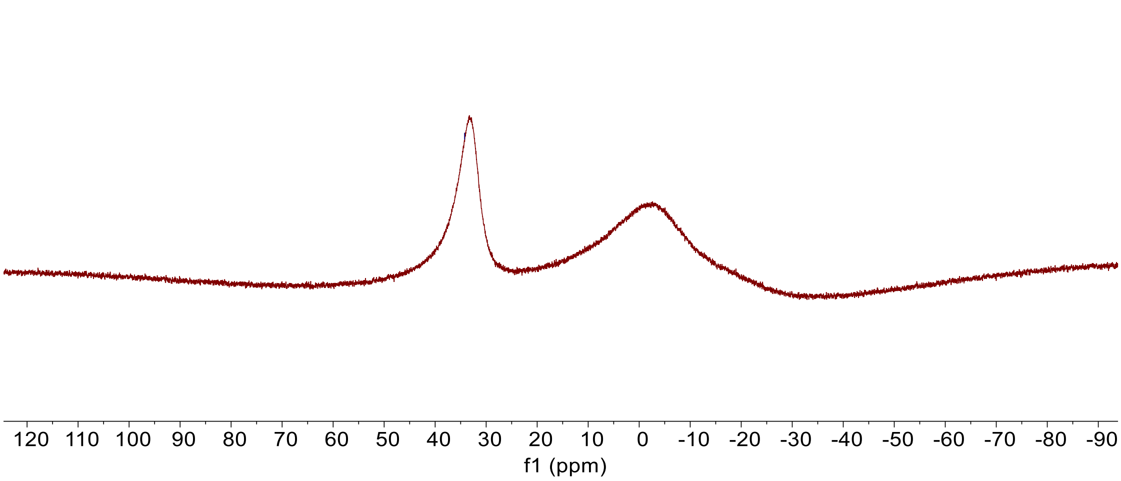

^{1}H NMR spectrum of compound **3f** in $CDCl_3$ (400 MHz).

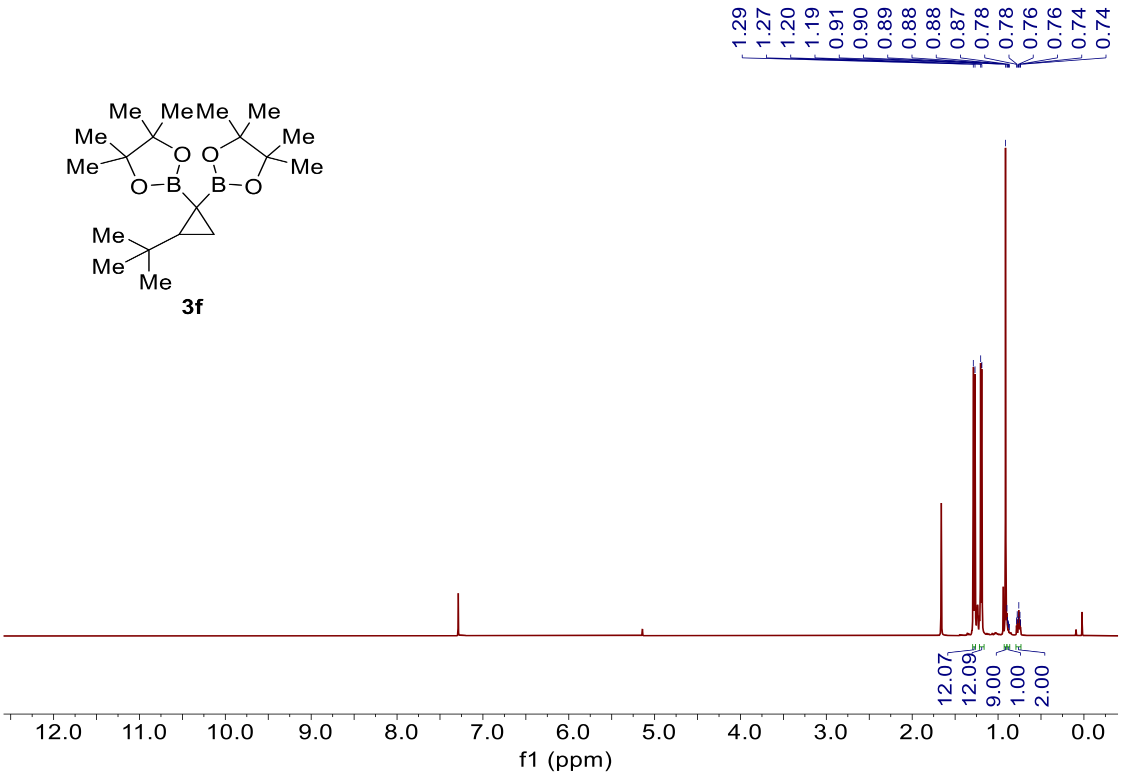

^{13}C NMR spectrum of compound **3f** in $CDCl_3$ (101 MHz).

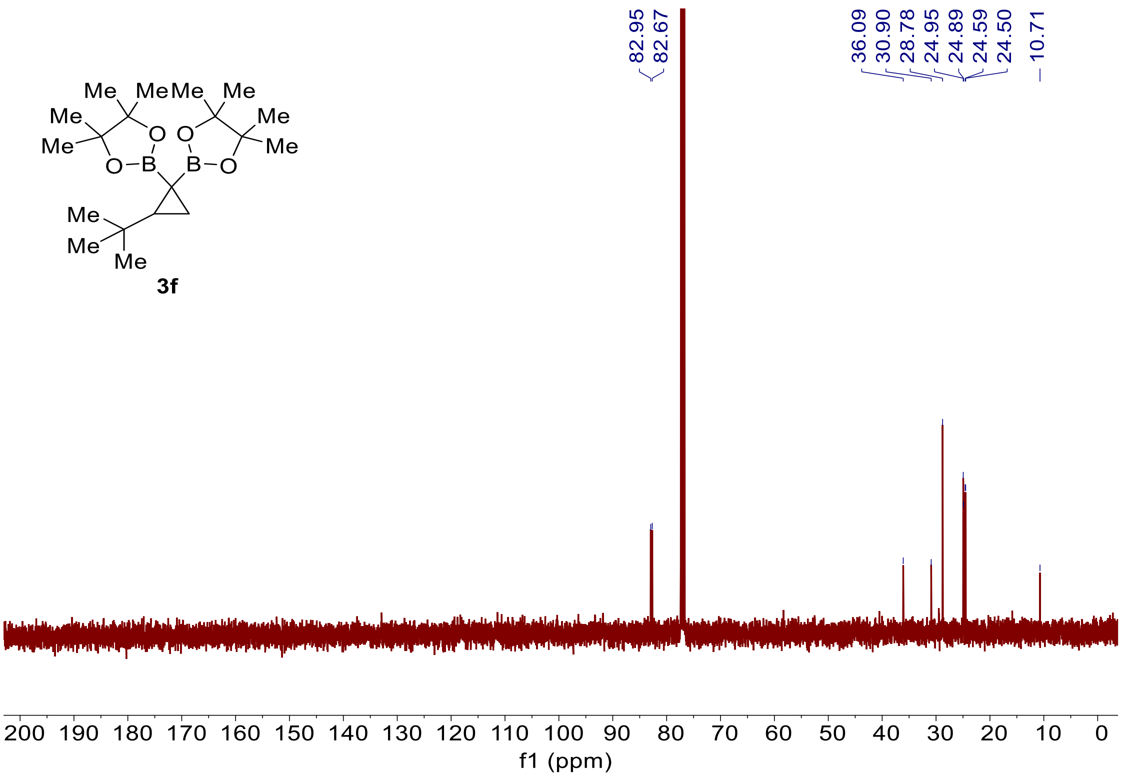

^{11}B NMR spectrum of compound **3f** in $CDCl_3$ (128 MHz).

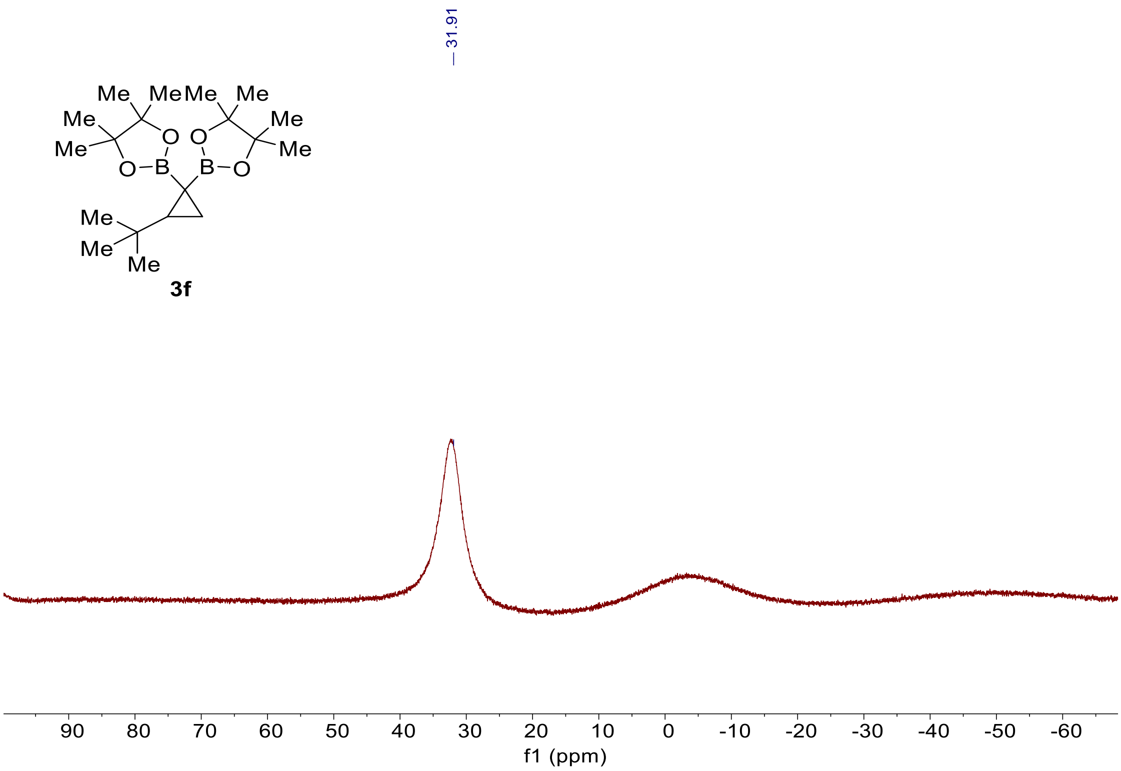

^{1}H NMR spectrum of compound **3g** in $CDCl_3$ (400 MHz).

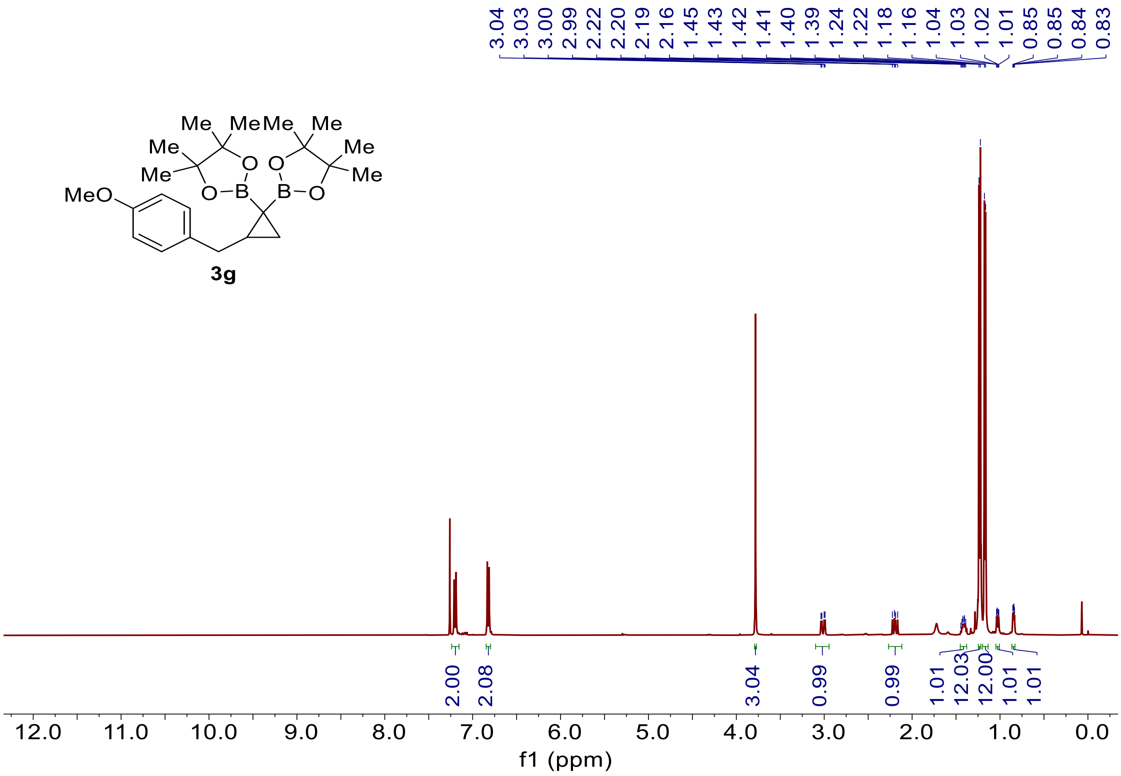

^{13}C NMR spectrum of compound **3g** in $CDCl_3$ (101 MHz).

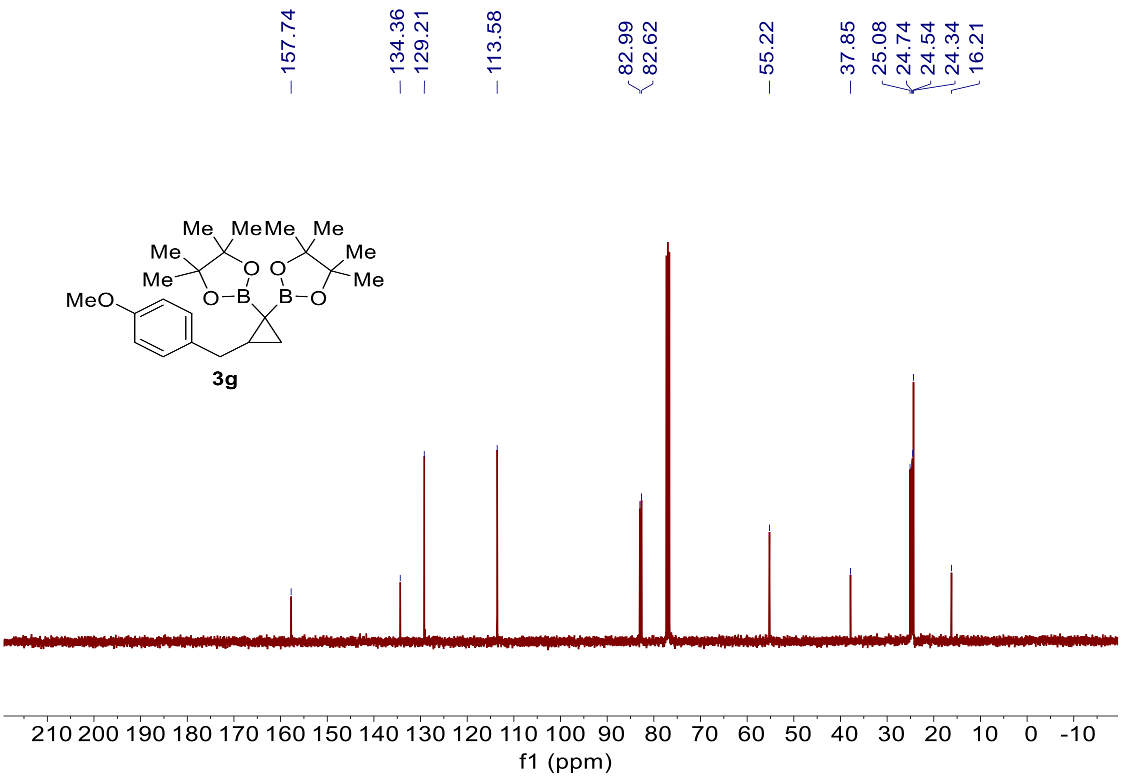

^{11}B NMR spectrum of compound **3g** in $CDCl_3$ (128 MHz).

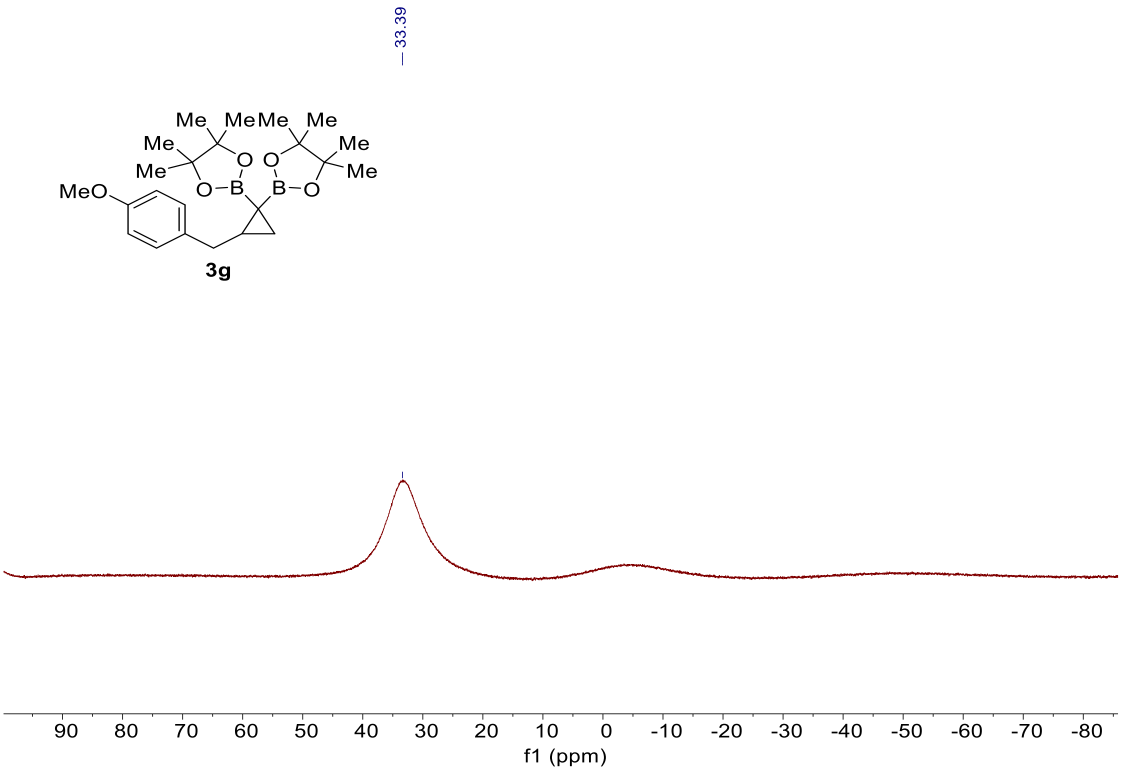

^{1}H NMR spectrum of compound **3h** in $CDCl_3$ (400 MHz).

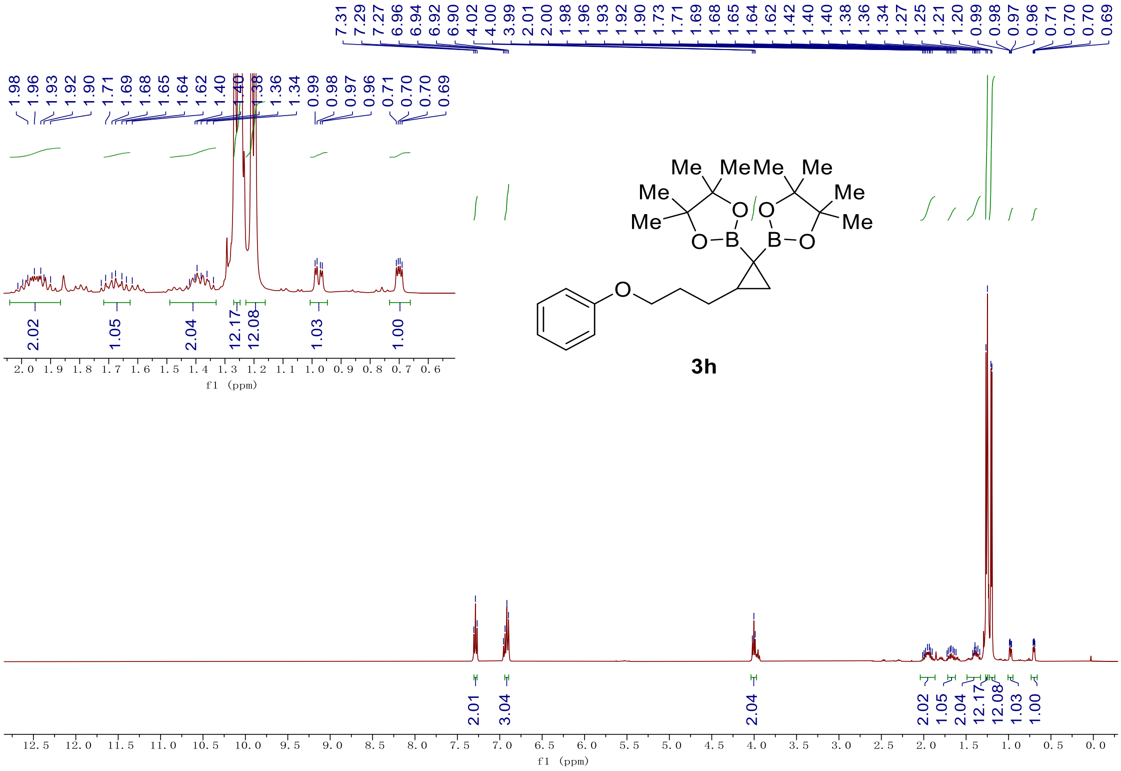

^{13}C NMR spectrum of compound **3h** in $CDCl_3$ (101 MHz).

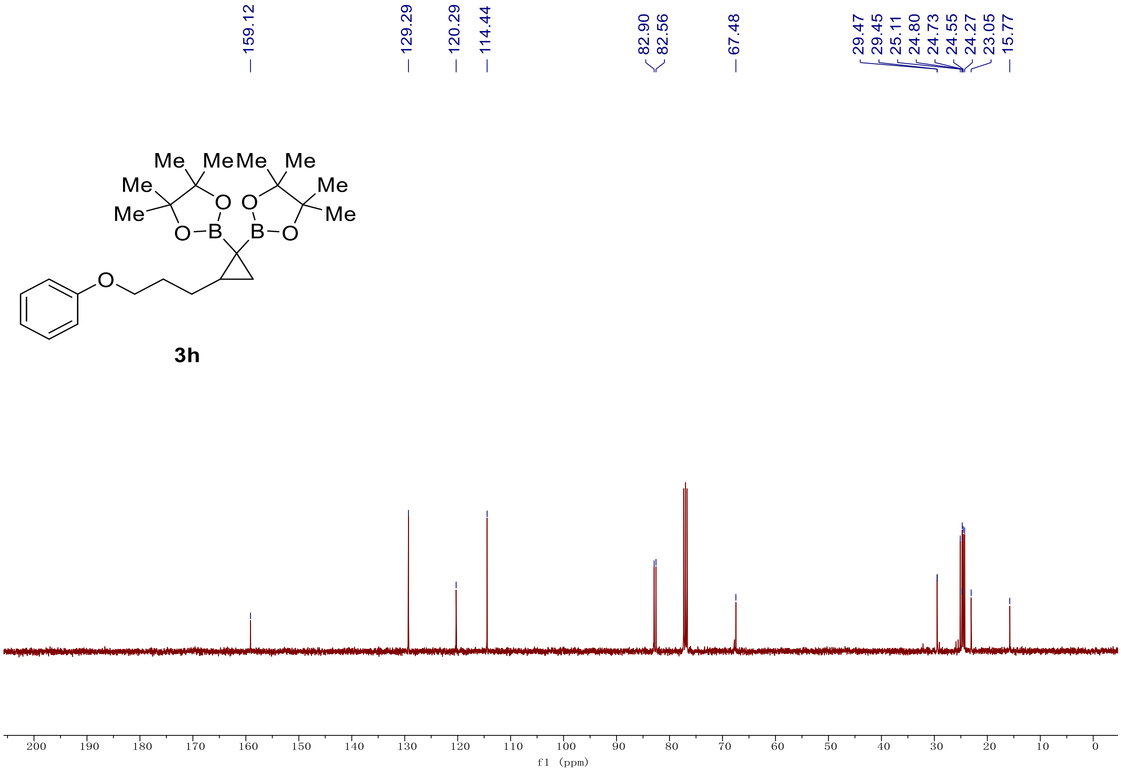

^{11}B NMR spectrum of compound **3h** in $CDCl_3$ (128 MHz).

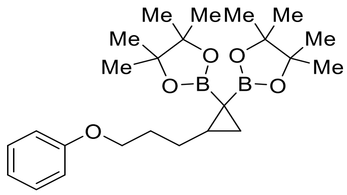

3h

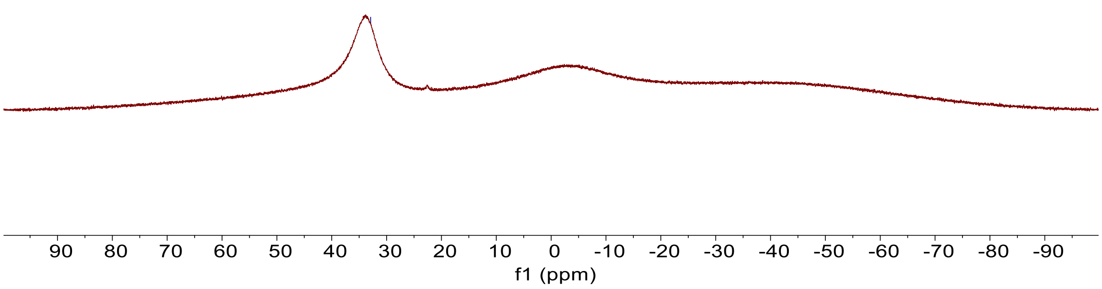

^{1}H NMR spectrum of compound **3i** in $CDCl_3$ (400 MHz).

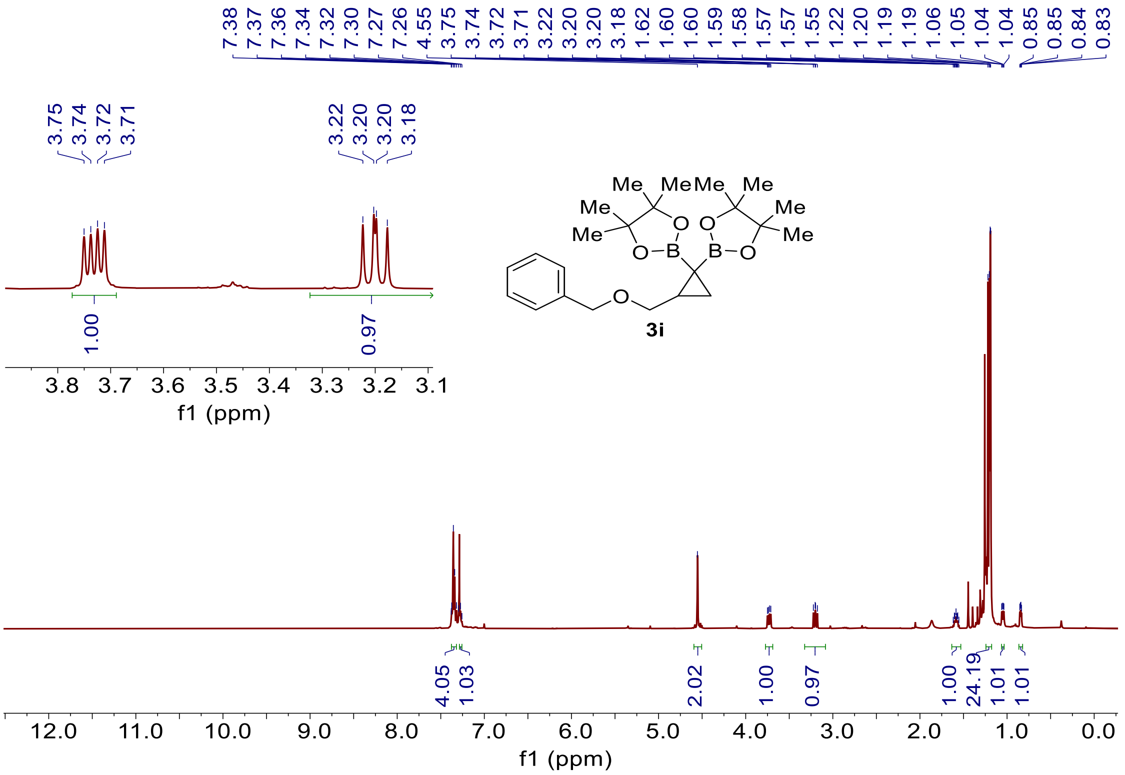

^{13}C NMR spectrum of compound **3i** in $CDCl_3$ (101 MHz).

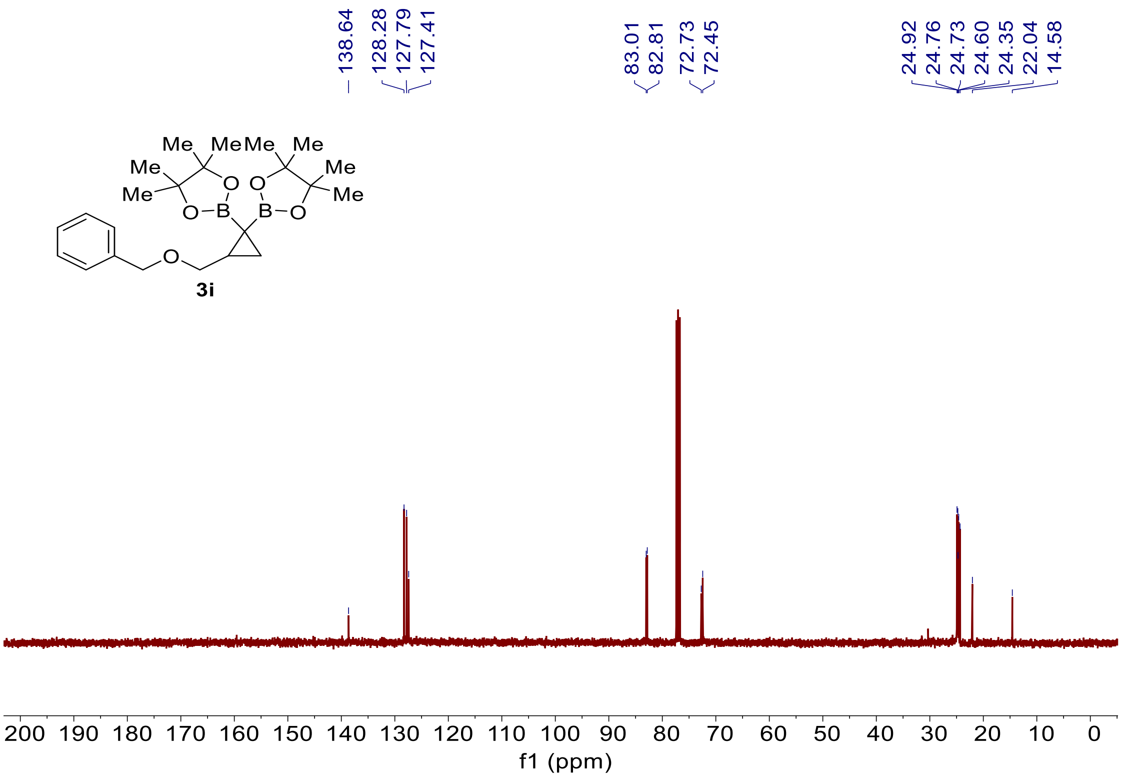

^{11}B NMR spectrum of compound **3i** in $CDCl_3$ (128 MHz).

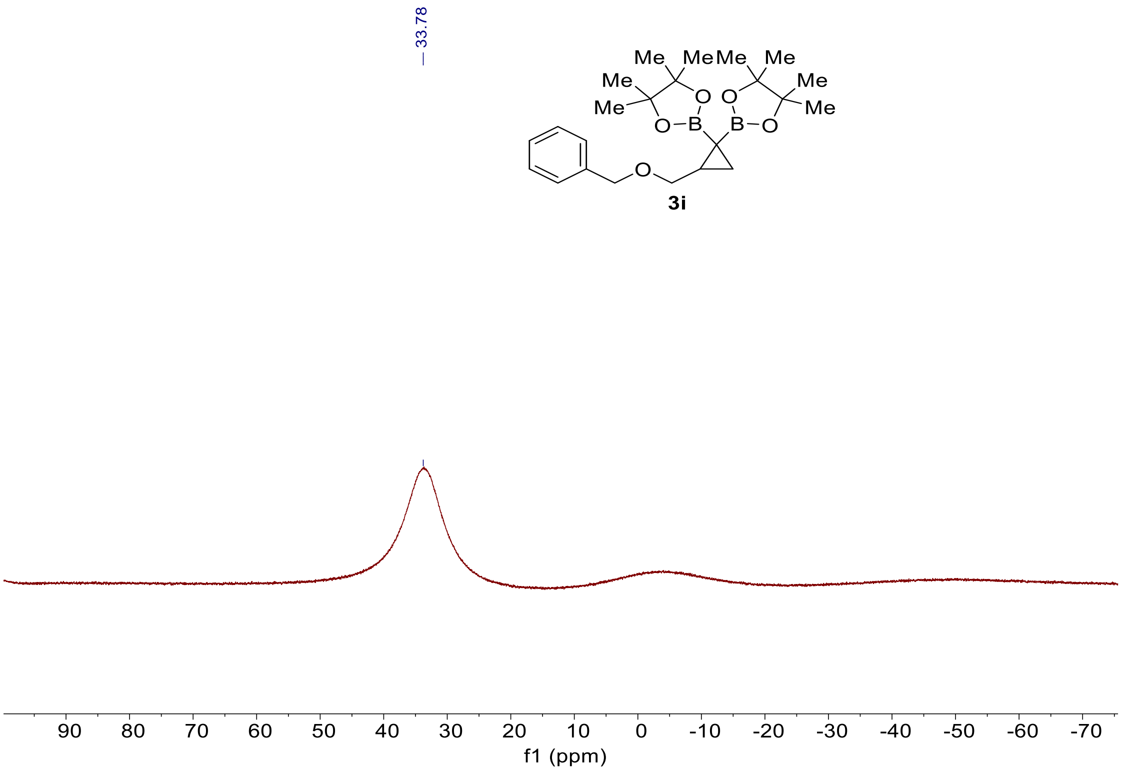

^{1}H NMR spectrum of compound **3j** in $CDCl_3$ (400 MHz).

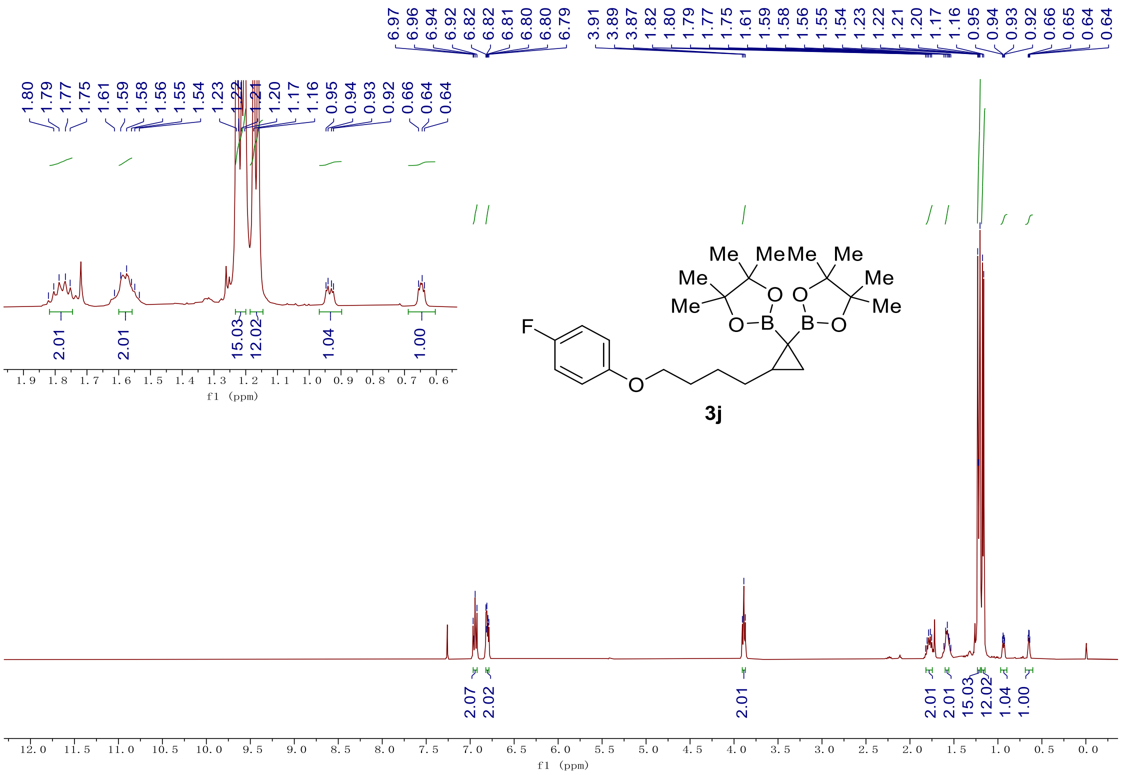

^{13}C NMR spectrum of compound **3j** in $CDCl_3$ (101 MHz).

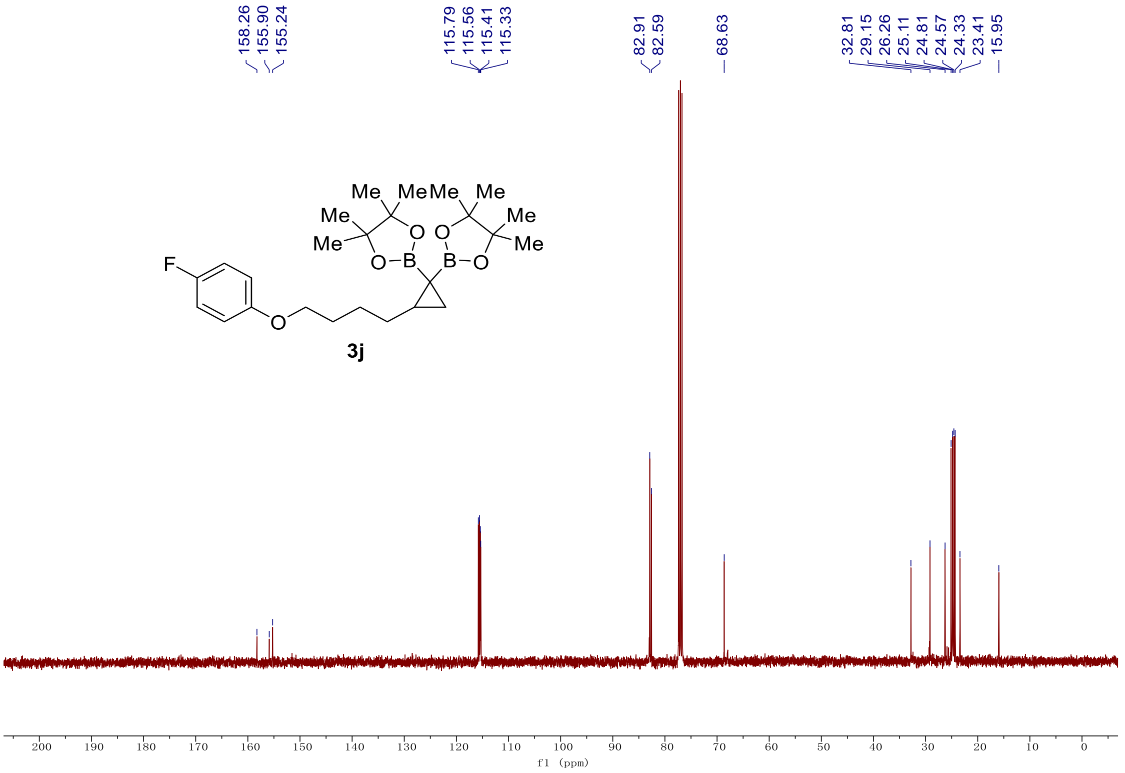

^{19}F NMR spectrum of compound **3j** in $CDCl_3$ (376 MHz).

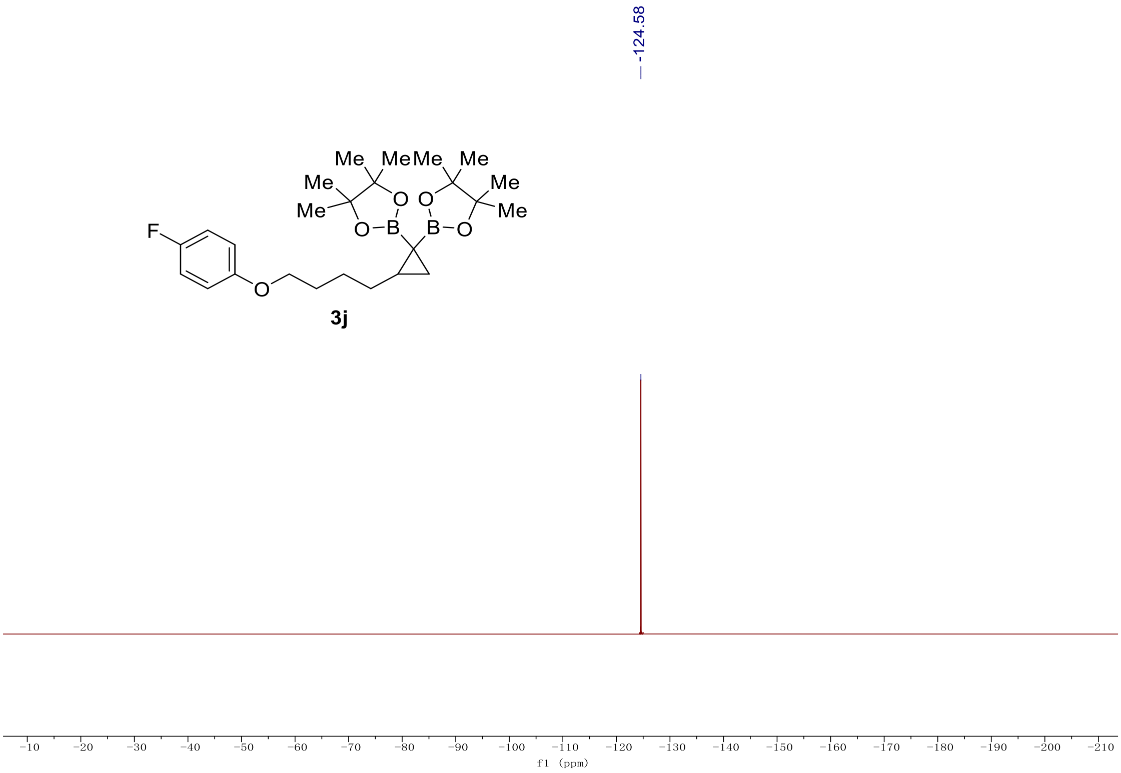

^{11}B NMR spectrum of compound **3j** in $CDCl_3$ (128 MHz).

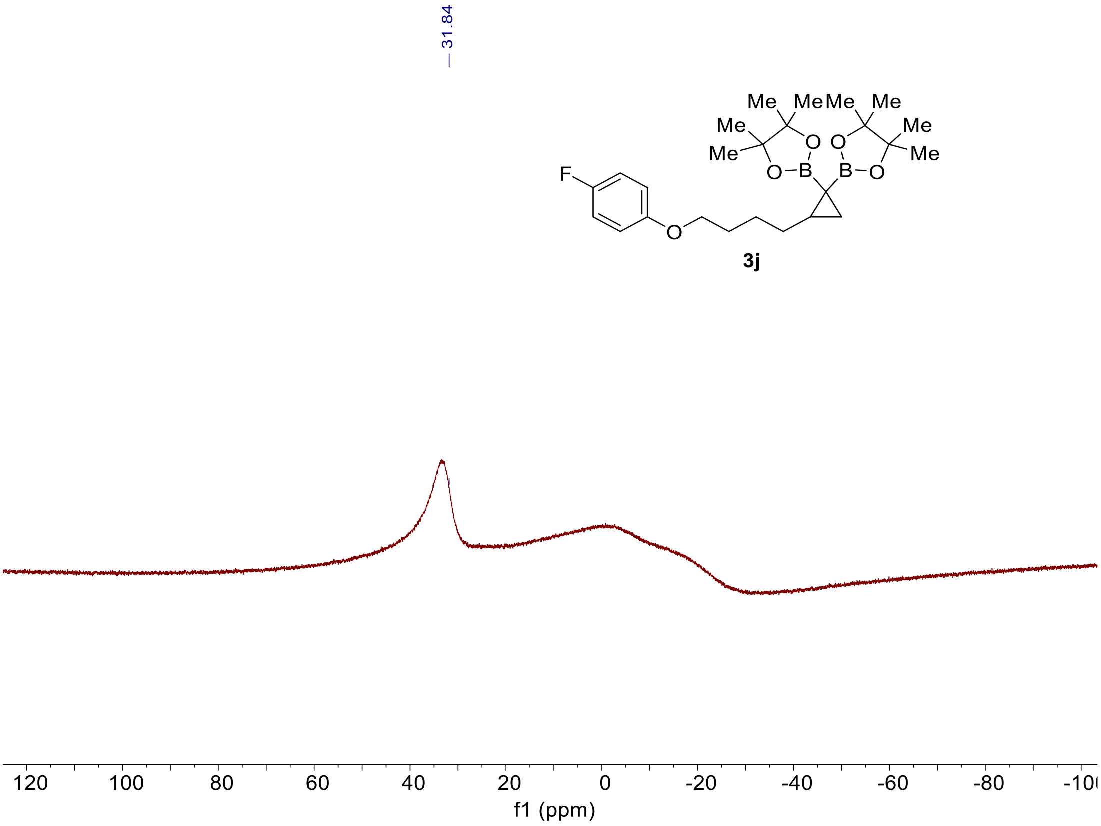

^{1}H NMR spectrum of compound **3k** in $CDCl_3$ (400 MHz).

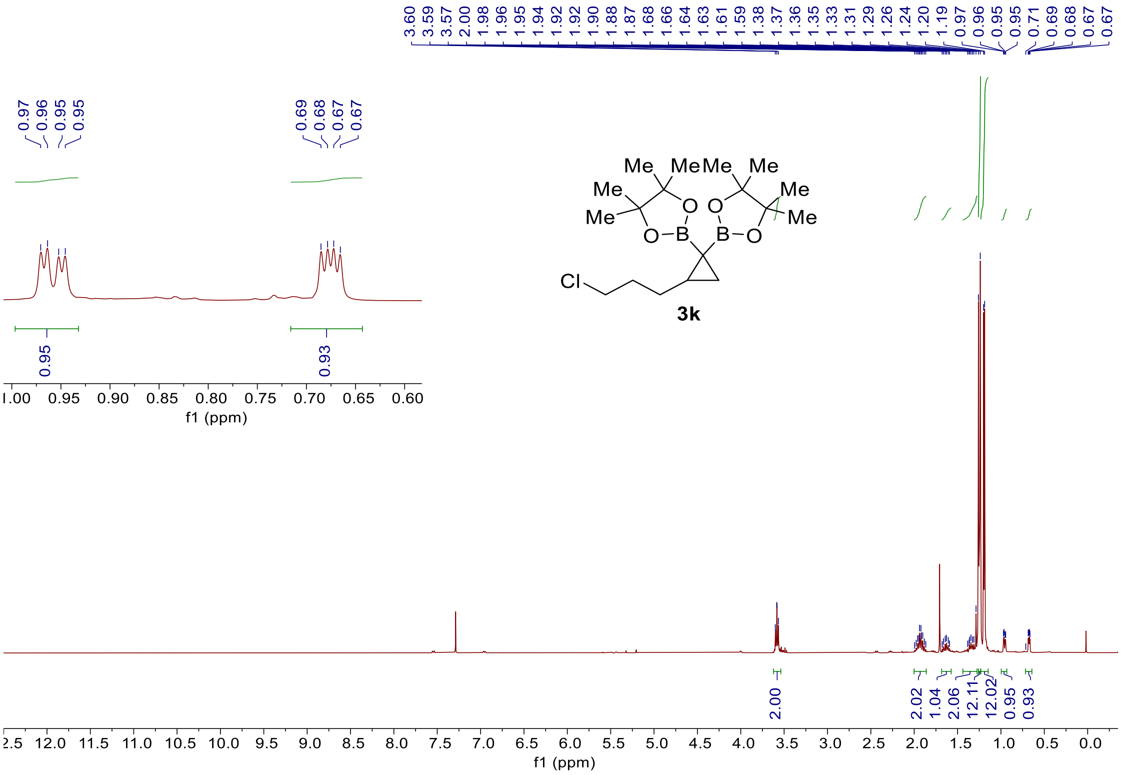

^{13}C NMR spectrum of compound **3k** in $CDCl_3$ (101 MHz).

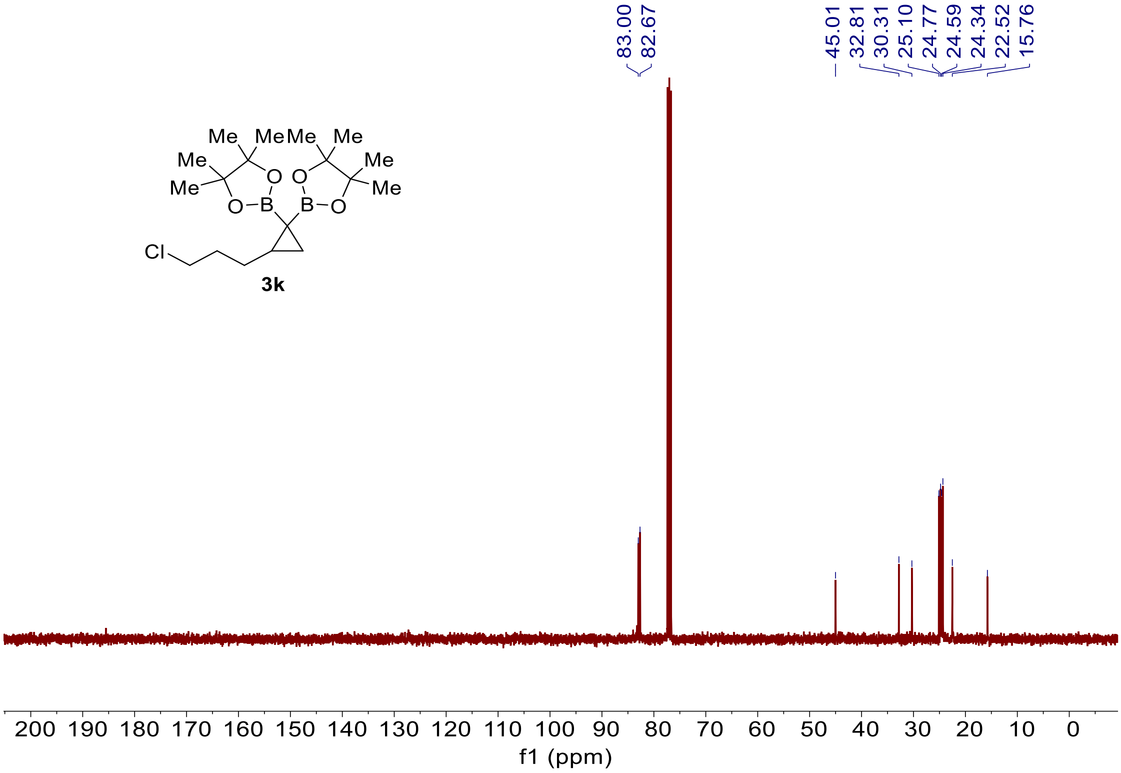

^{11}B NMR spectrum of compound **3k** in $CDCl_3$ (128 MHz).

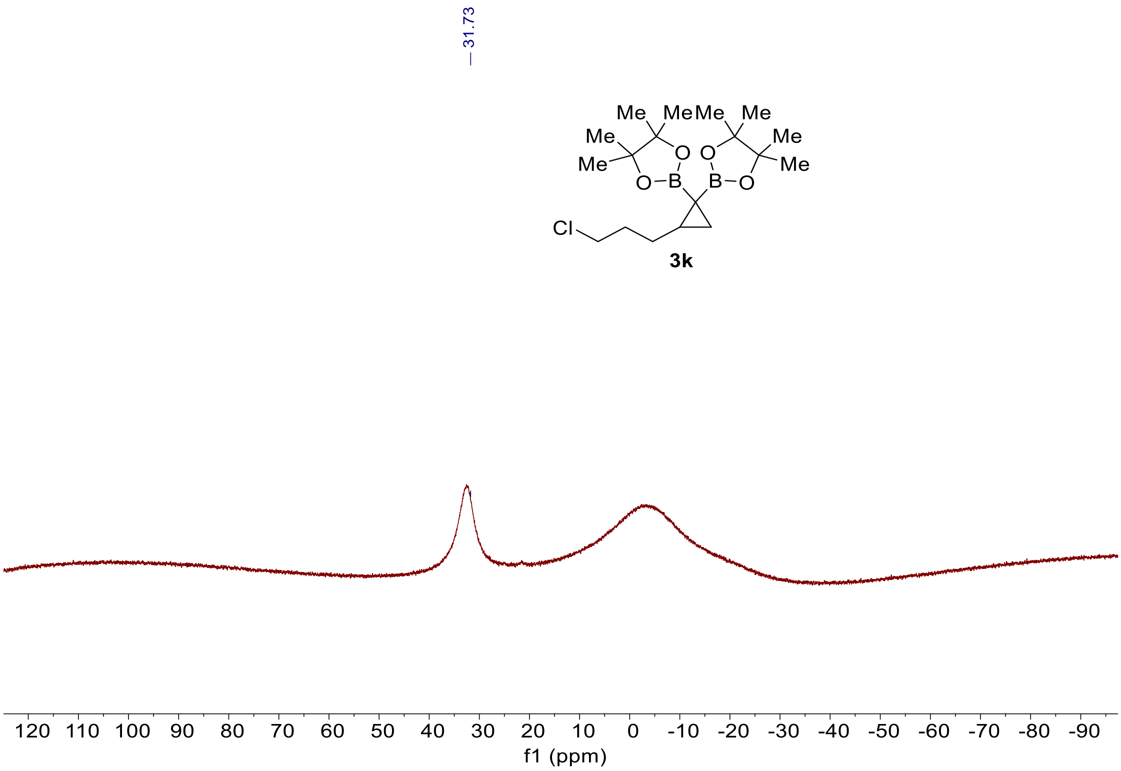

^{1}H NMR spectrum of compound **3l** in $CDCl_3$ (400 MHz).

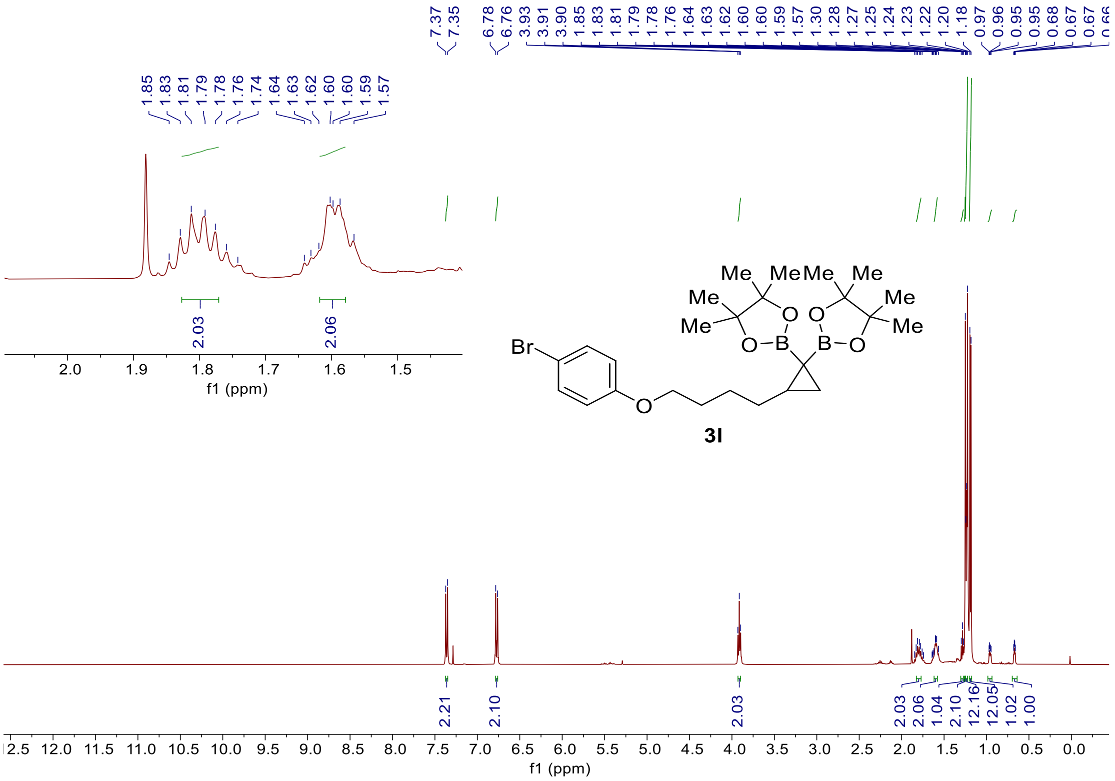

^{13}C NMR spectrum of compound **3l** in $CDCl_3$ (101 MHz).

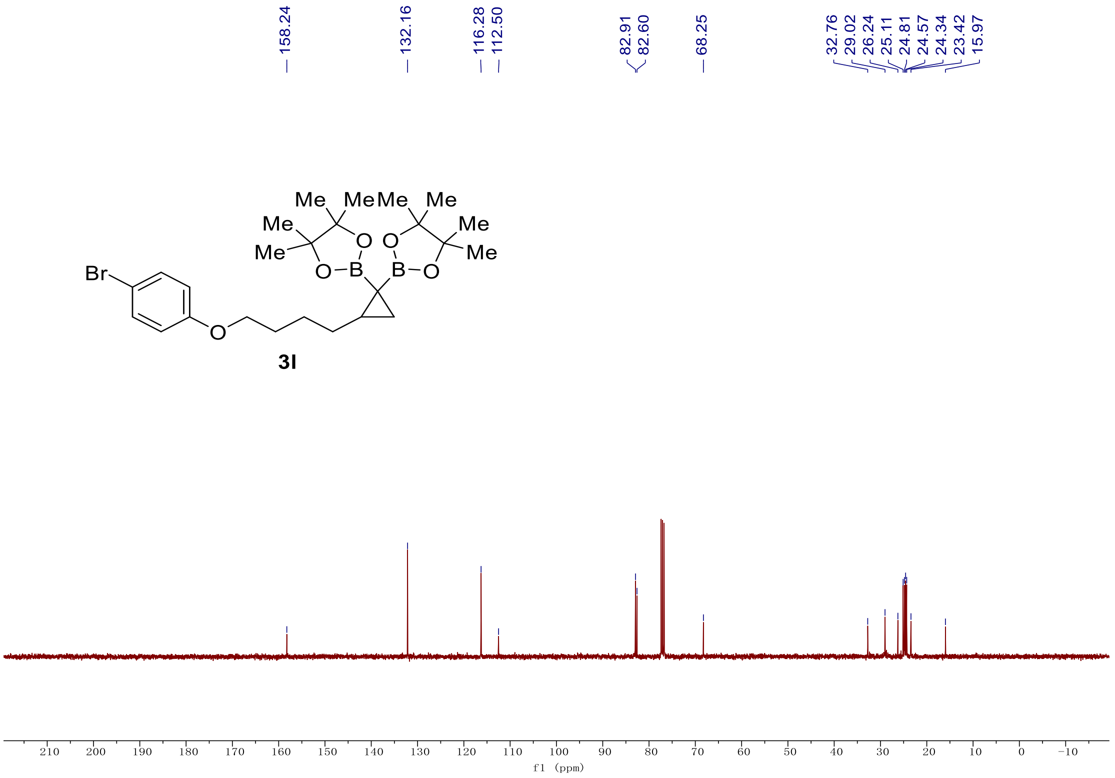

^{11}B NMR spectrum of compound **3l** in $CDCl_3$ (128 MHz).

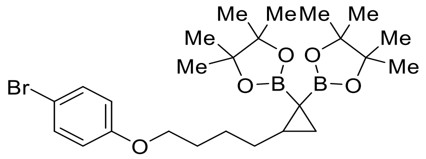

3l

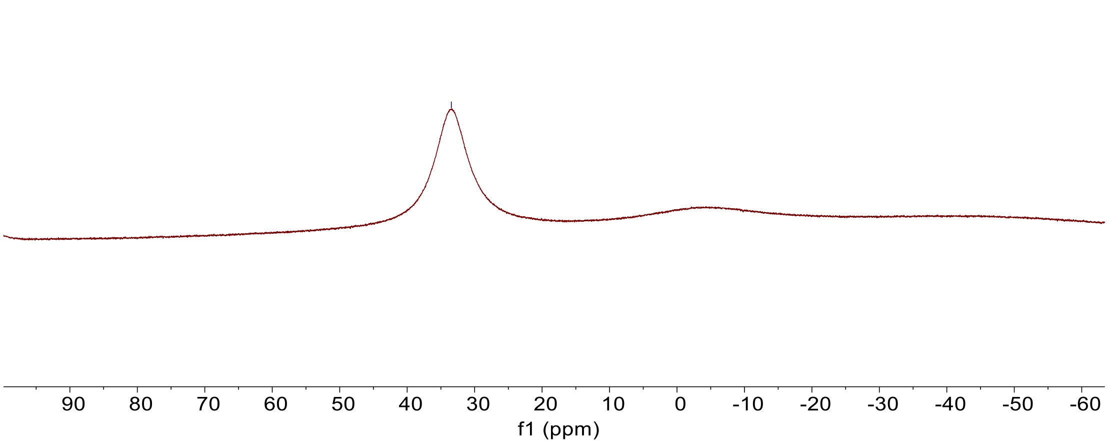

^{1}H NMR spectrum of compound **3m** in $CDCl_3$ (400 MHz).

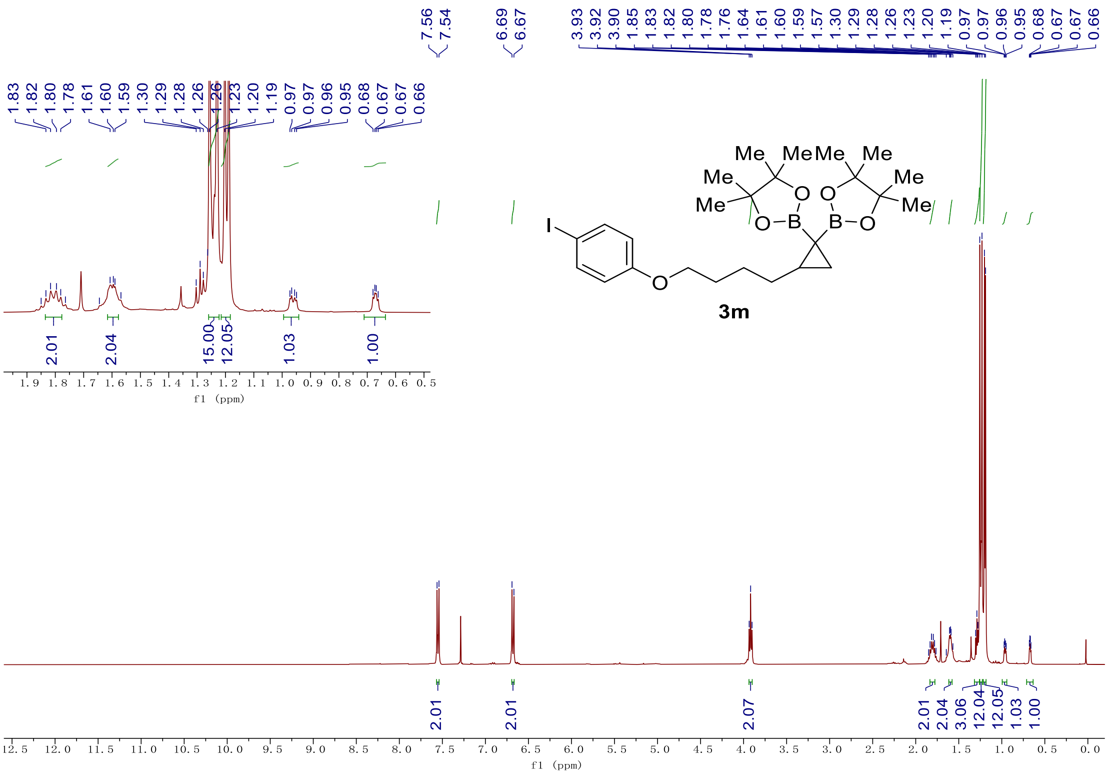

^{13}C NMR spectrum of compound **3m** in $CDCl_3$ (101 MHz).

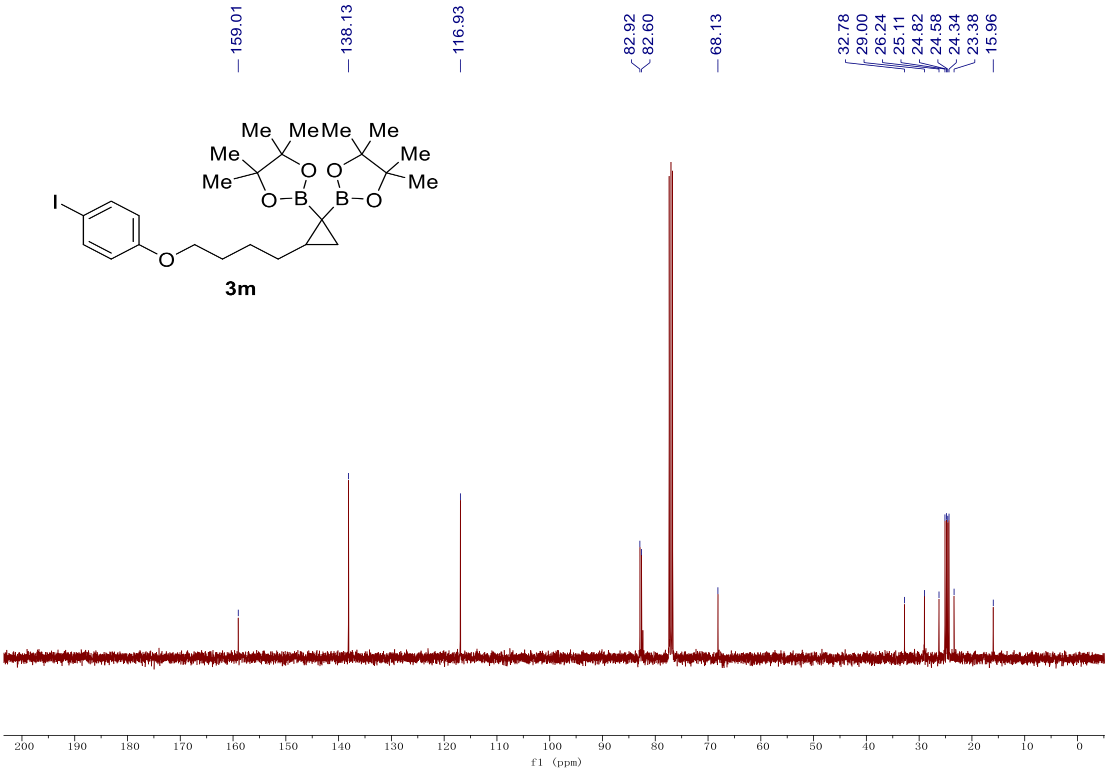

^{11}B NMR spectrum of compound **3m** in $CDCl_3$ (128 MHz).

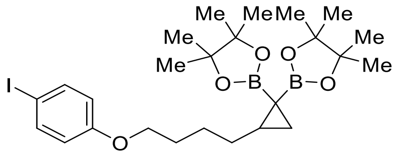

3m

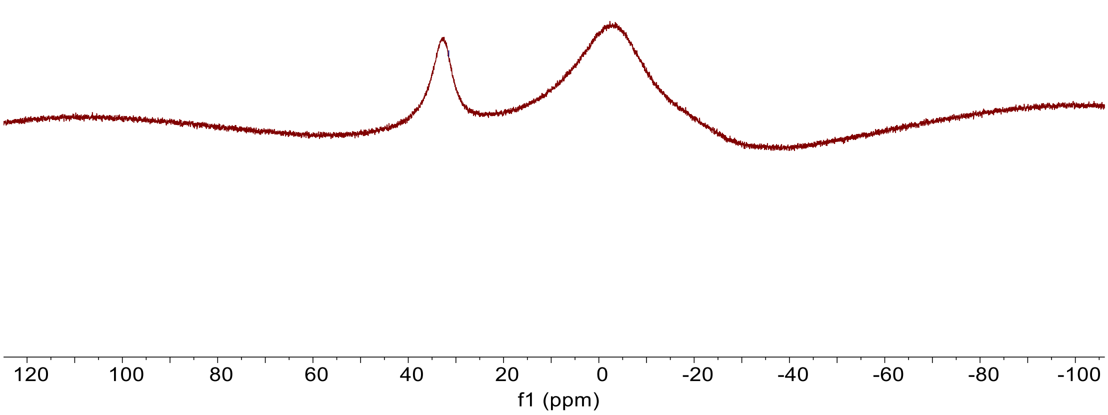

^{1}H NMR spectrum of compound **3n** in $CDCl_3$ (400 MHz).

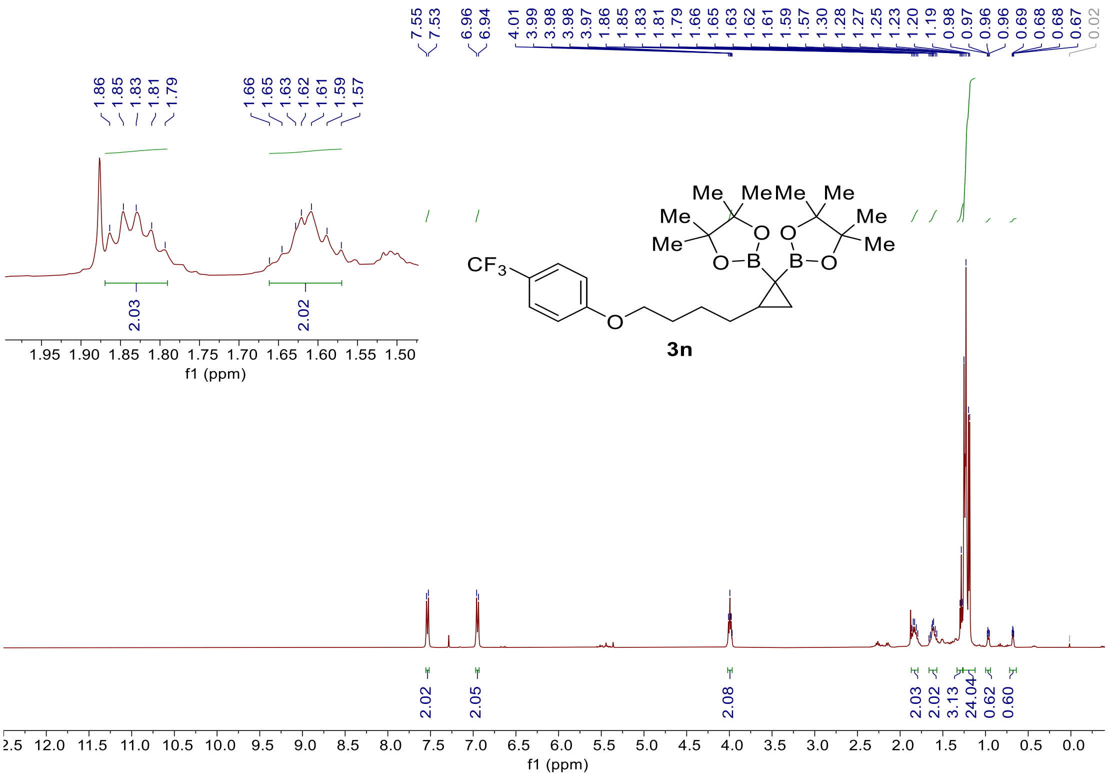

^{13}C NMR spectrum of compound **3n** in $CDCl_3$ (101 MHz).

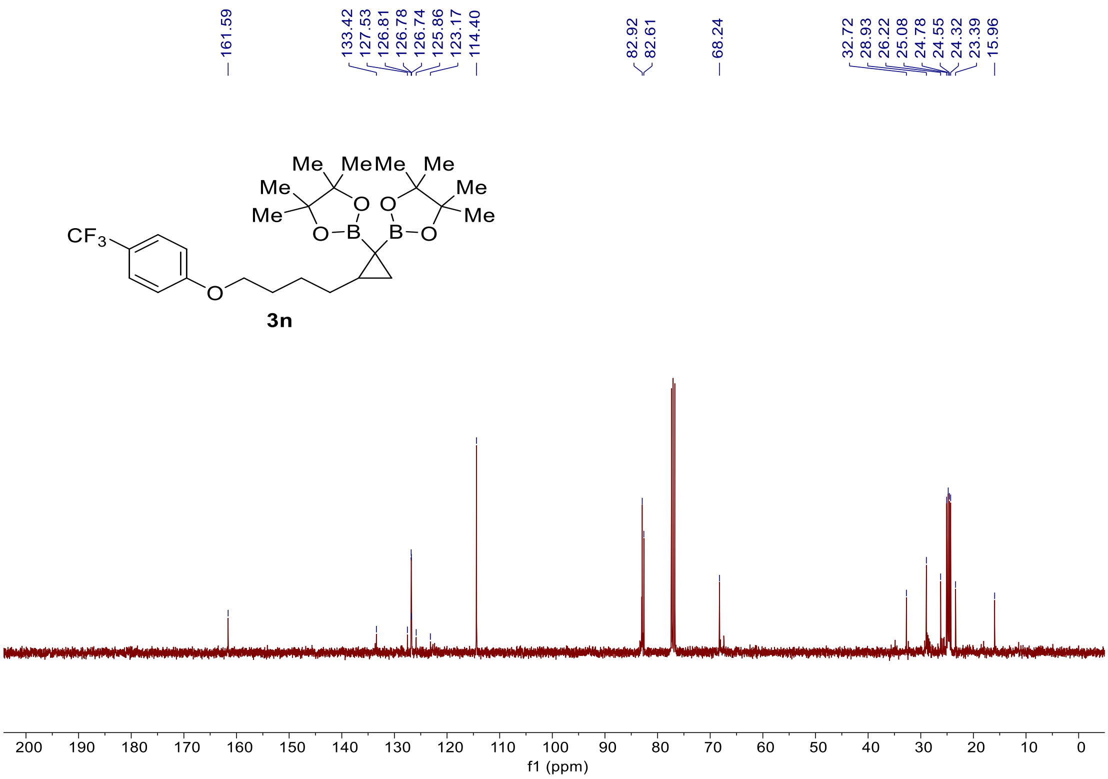

^{11}B NMR spectrum of compound **3n** in $CDCl_3$ (128 MHz).

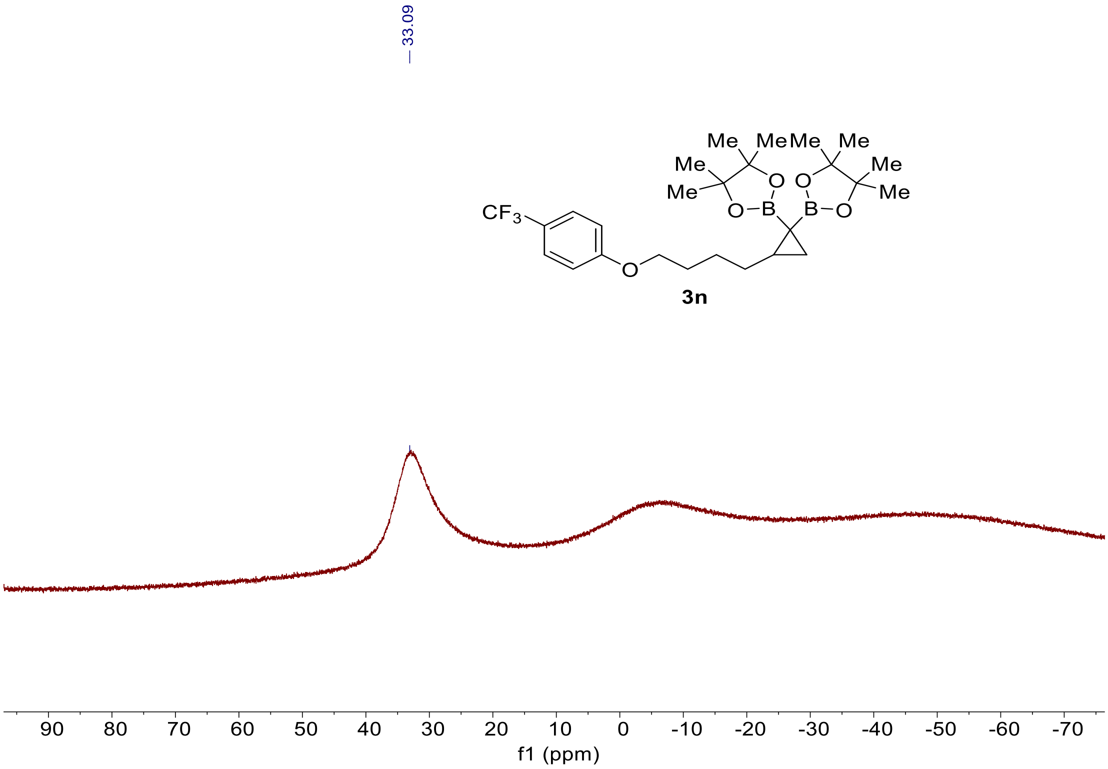

^{1}H NMR spectrum of compound **3o** in $CDCl_3$ (400 MHz).

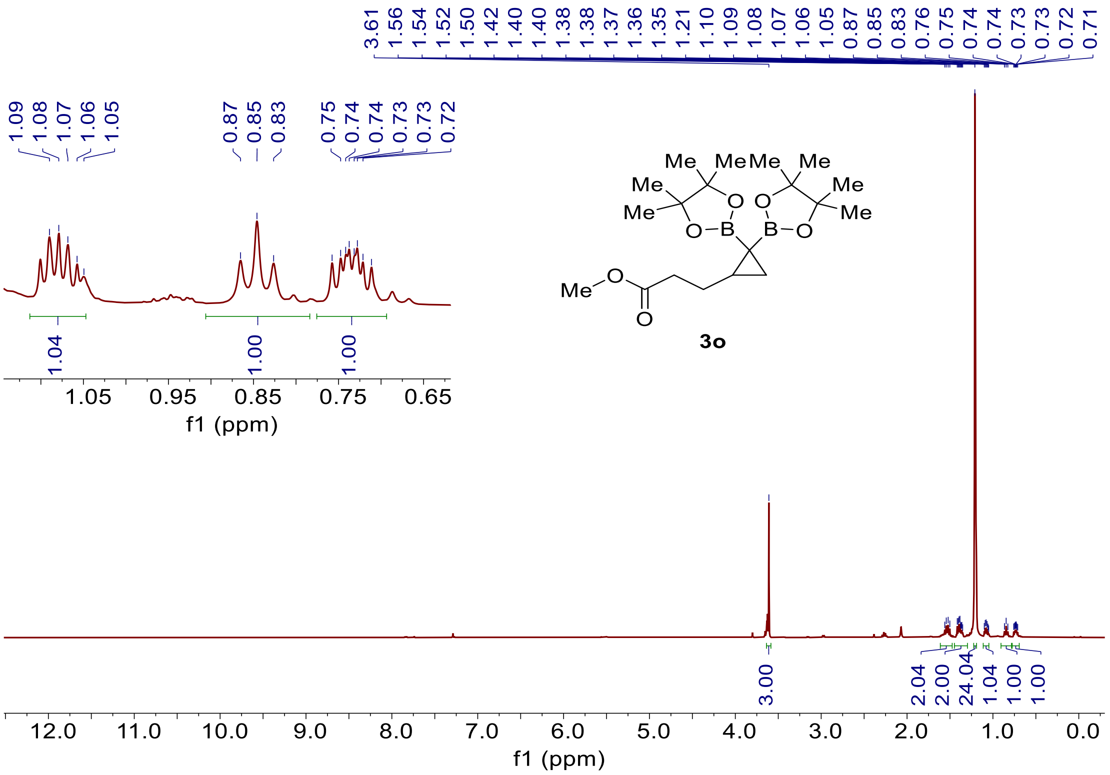

^{13}C NMR spectrum of compound **3o** in $CDCl_3$ (101 MHz).

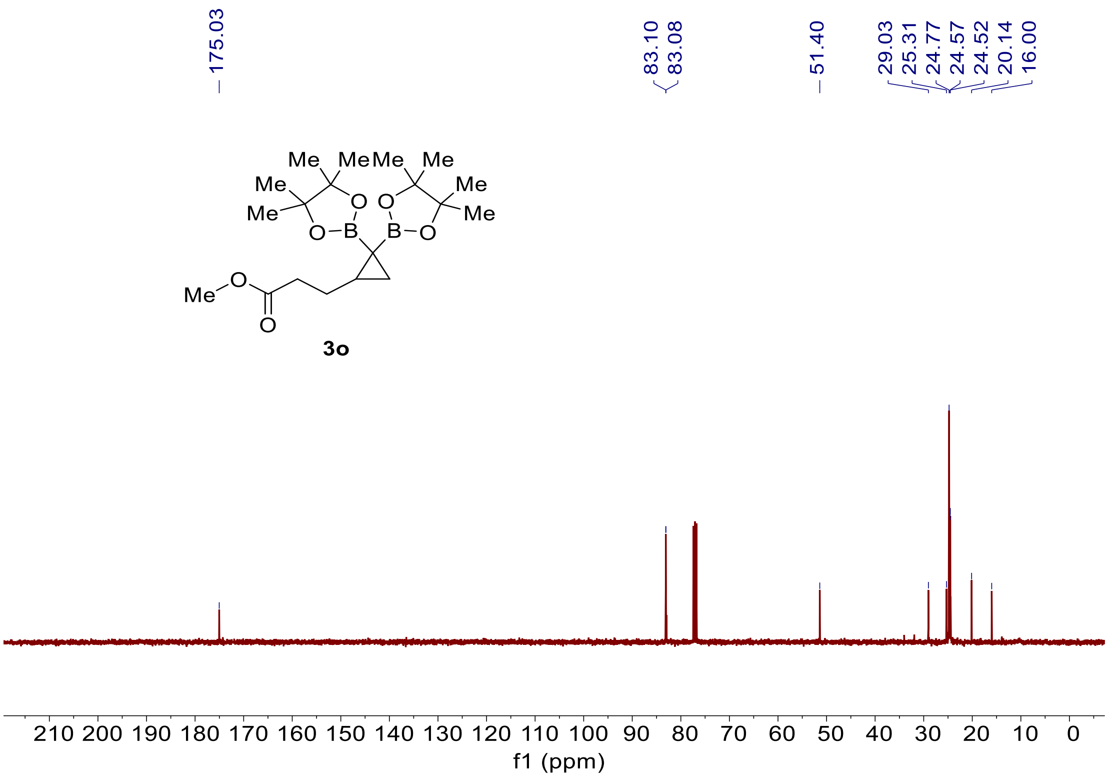

^{11}B NMR spectrum of compound **3o** in $CDCl_3$ (128 MHz).

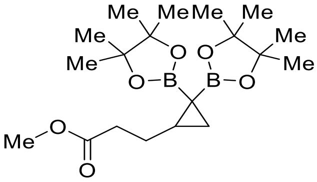

3o

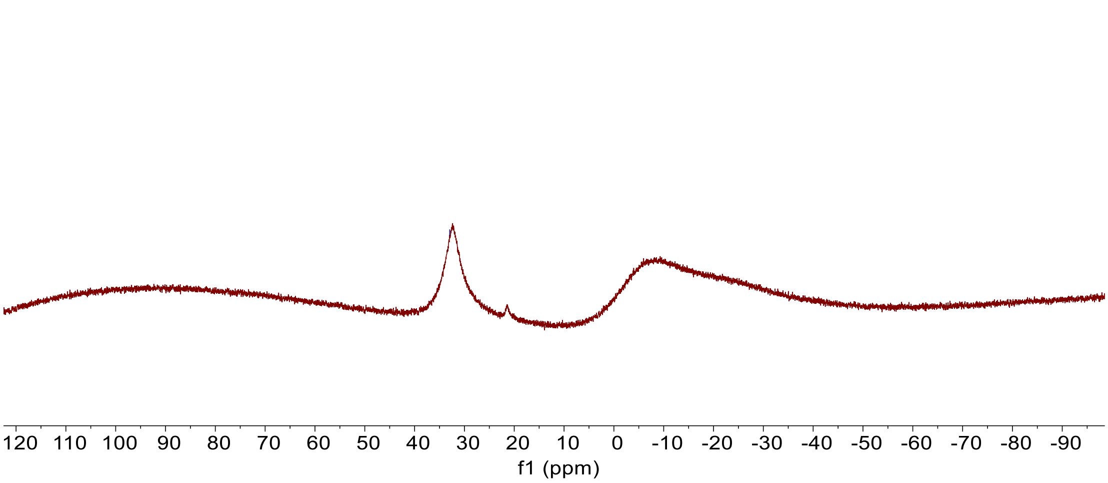

^{1}H NMR spectrum of compound **3p** in CDCl3 (400 MHz).

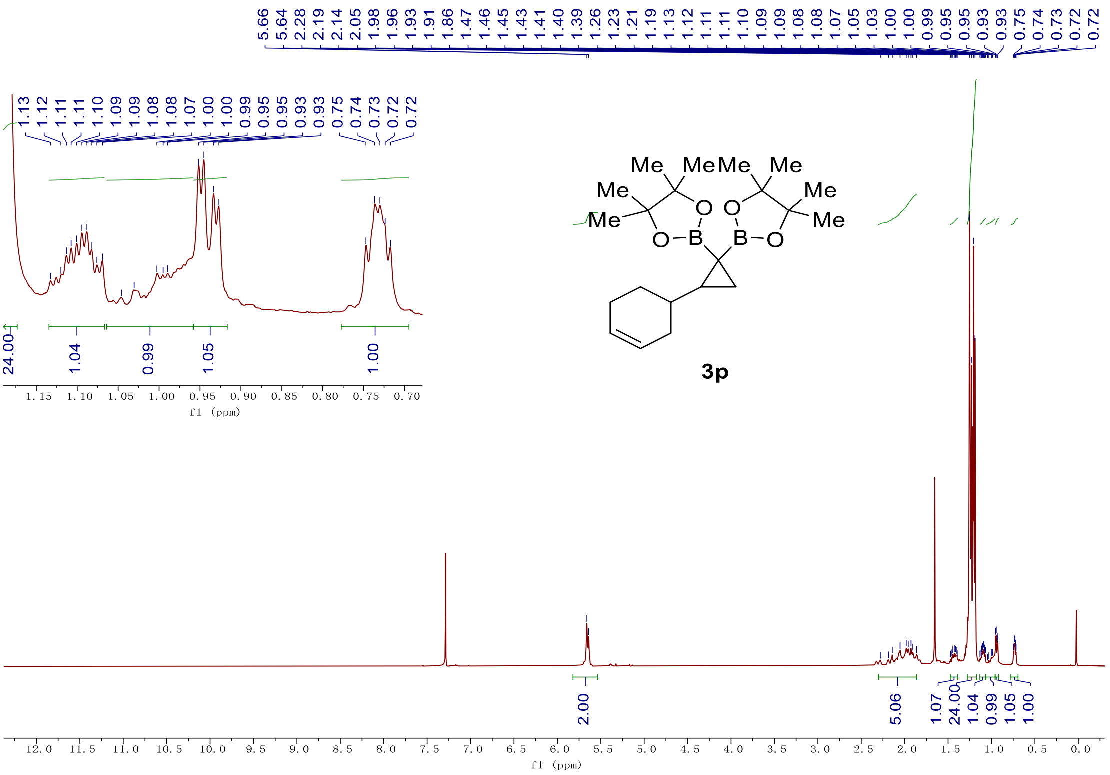

^{13}C NMR spectrum of compound **3p** in CDCl3 (101 MHz).

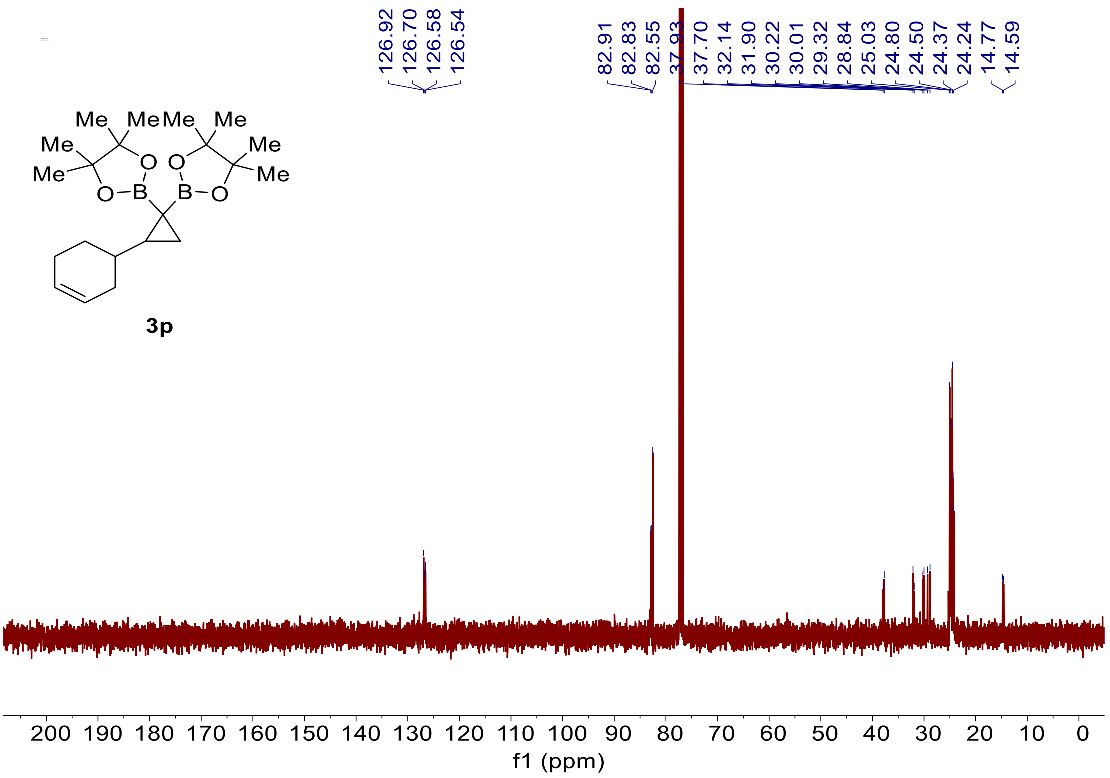

^{11}B NMR spectrum of compound **3p** in $CDCl_3$ (128 MHz).

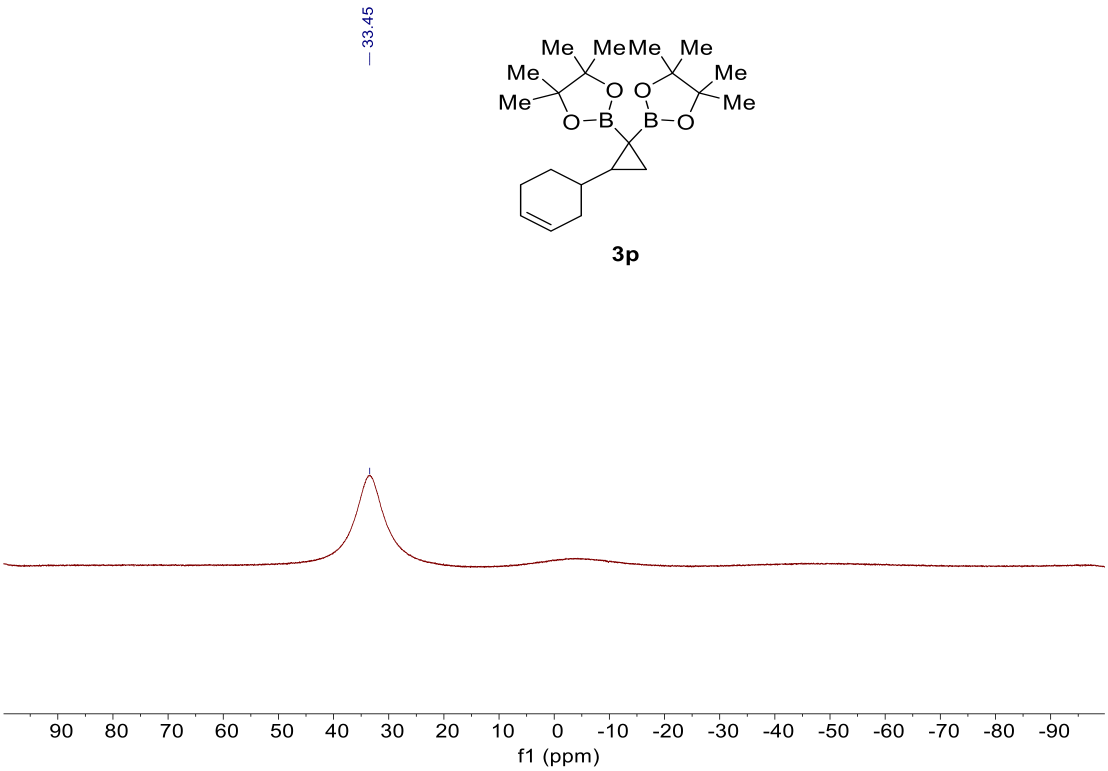

^{1}H NMR spectrum of compound **3q** in $CDCl_3$ (400 MHz).

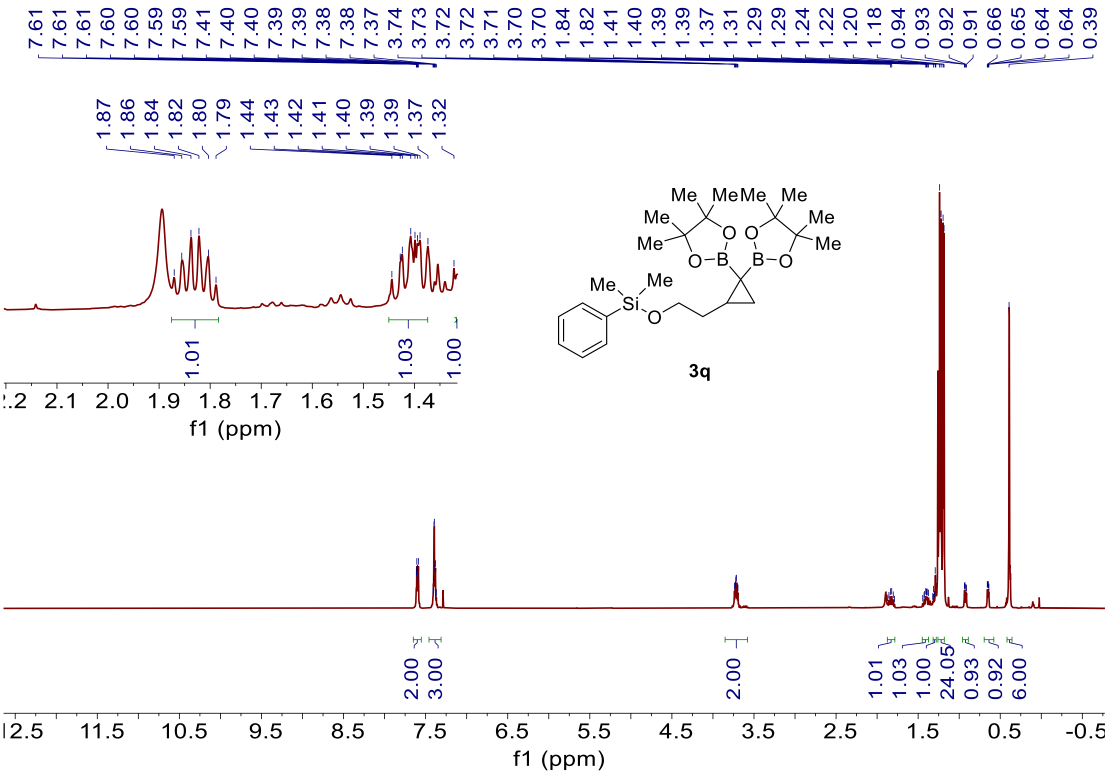

^{13}C NMR spectrum of compound **3q** in $CDCl_3$ (101 MHz).

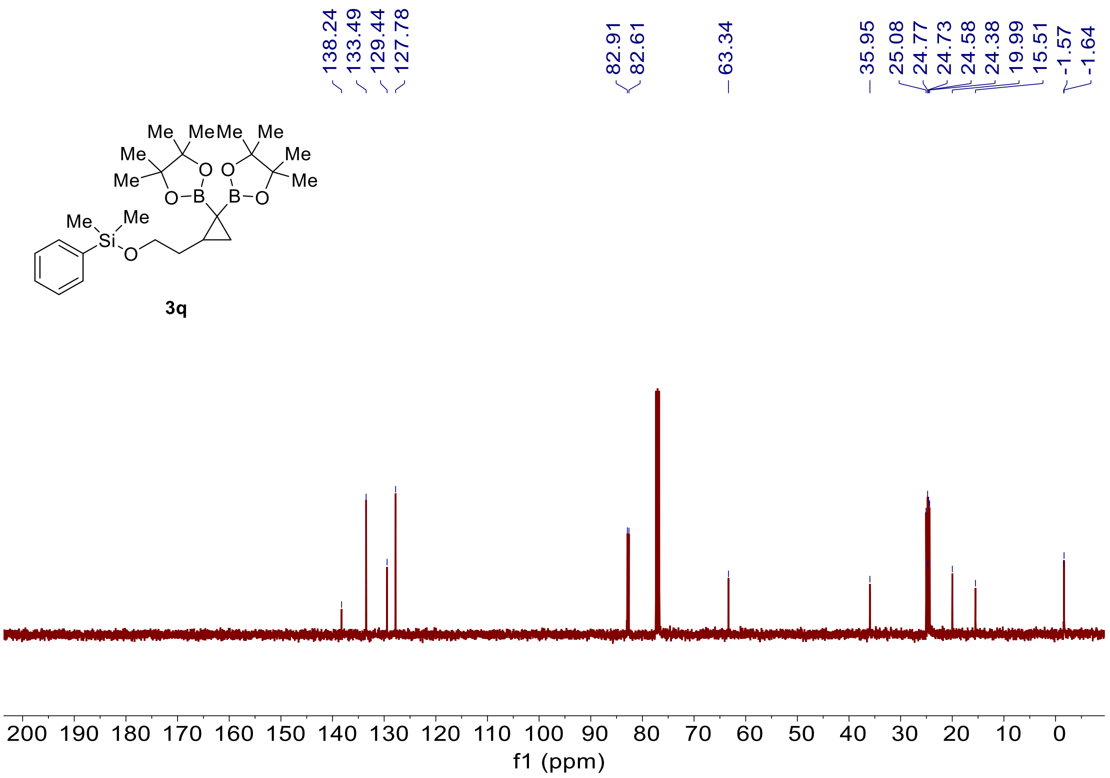

^{11}B NMR spectrum of compound **3q** in $CDCl_3$ (128 MHz).

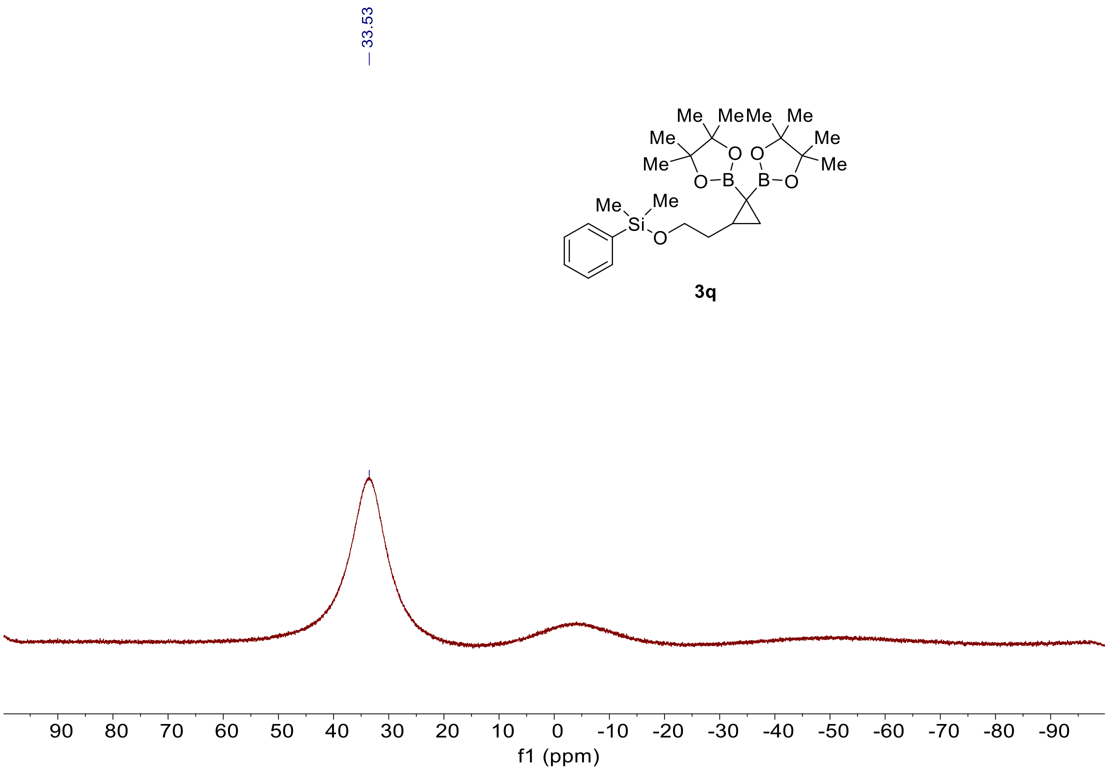

^{1}H NMR spectrum of compound **3r** in $CDCl_3$ (400 MHz).

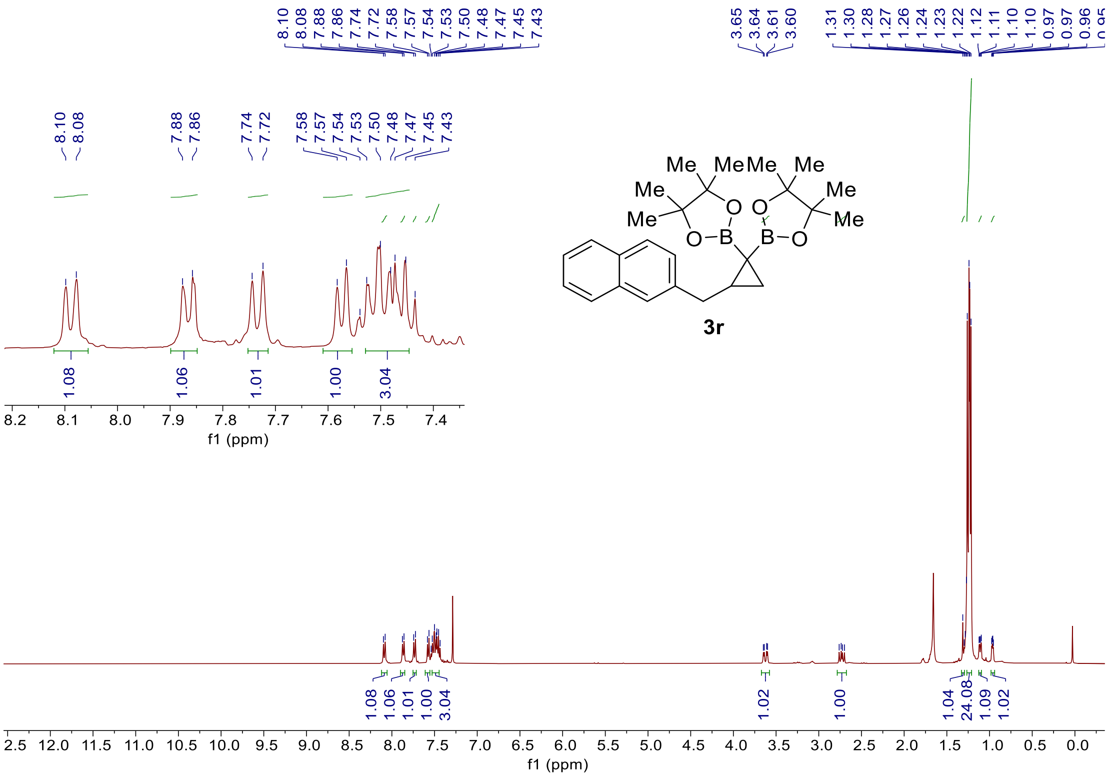

^{13}C NMR spectrum of compound **3r** in $CDCl_3$ (101 MHz).

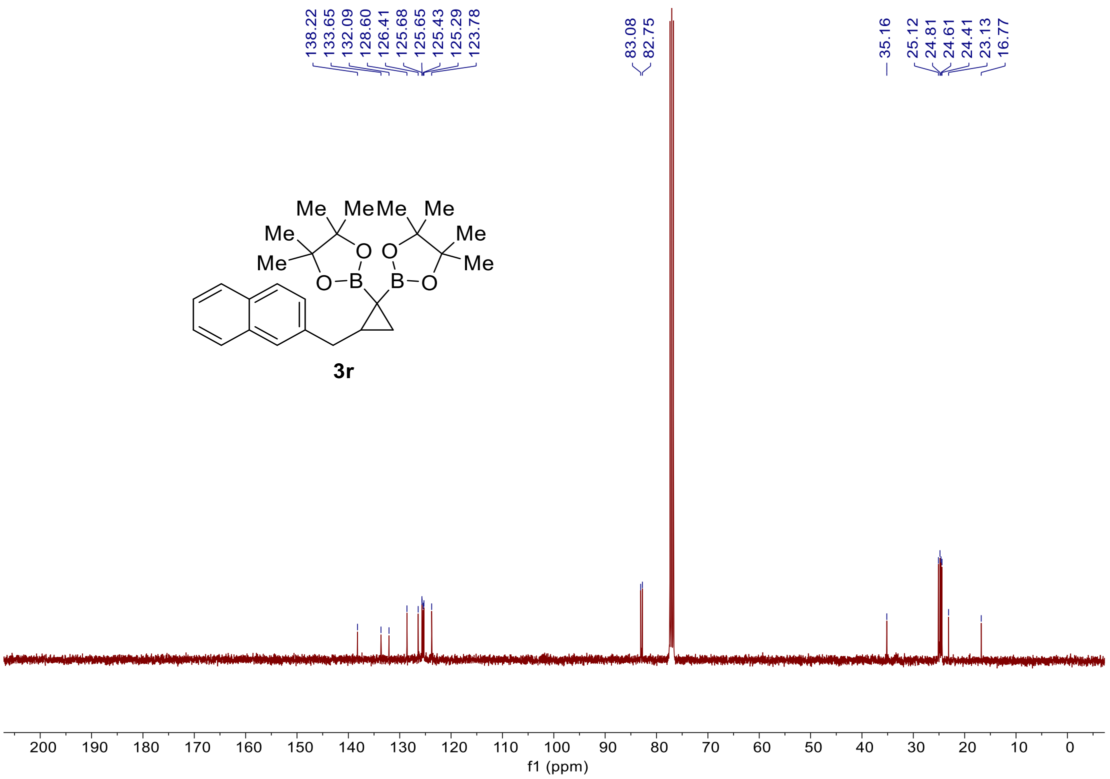

^{11}B NMR spectrum of compound **3r** in $CDCl_3$ (128 MHz).

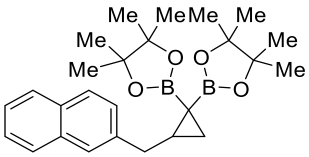

3r

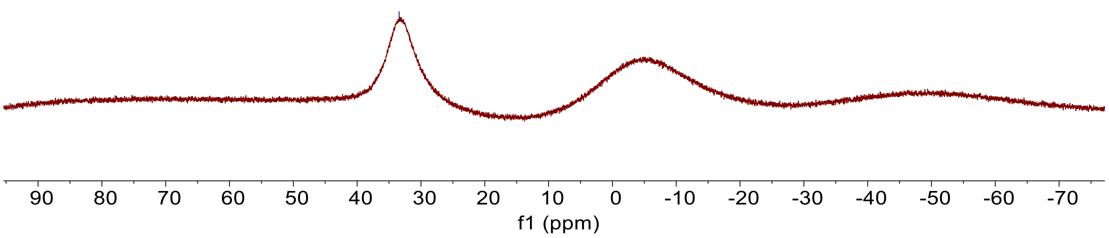

^{1}H NMR spectrum of compound **3s** in $CDCl_3$ (400 MHz).

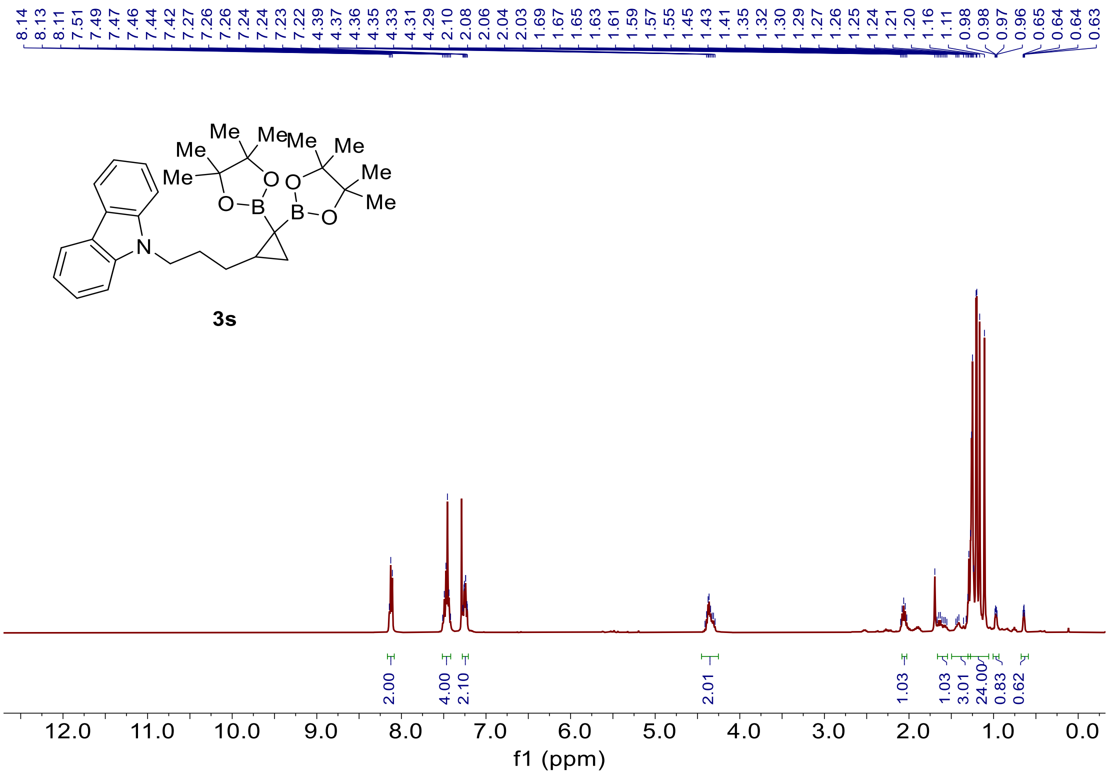

^{13}C NMR spectrum of compound **3s** in $CDCl_3$ (101 MHz).

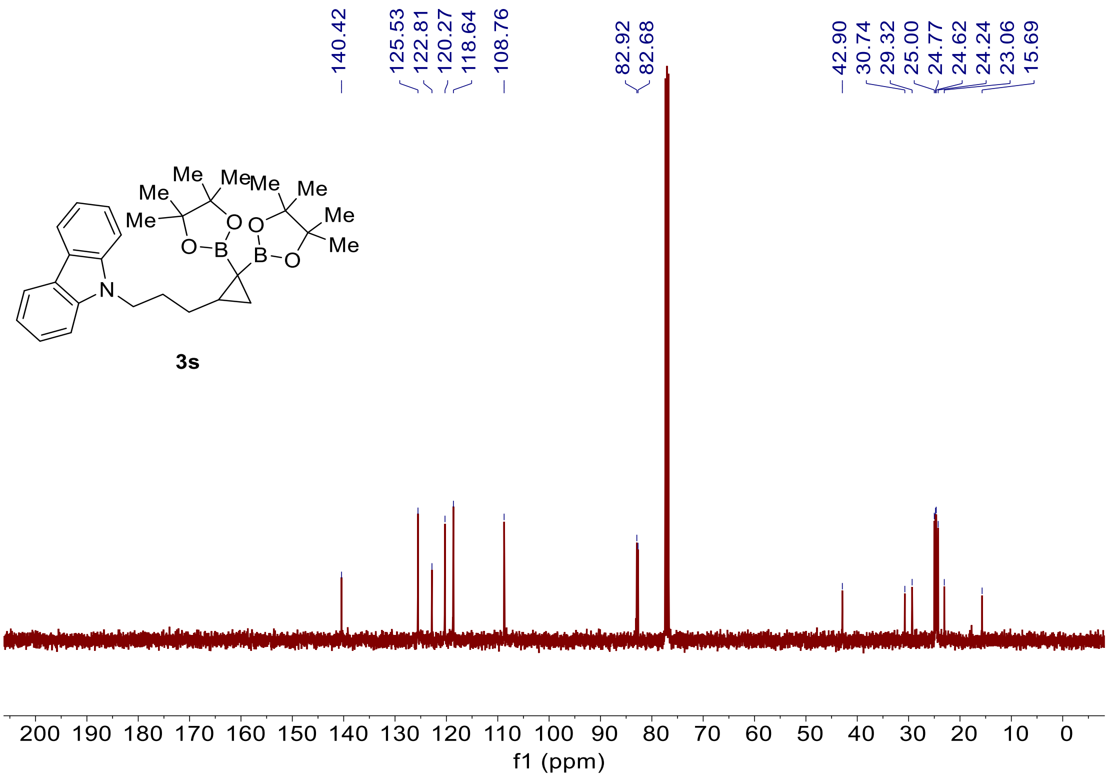

^{11}B NMR spectrum of compound **3s** in $CDCl_3$ (128 MHz).

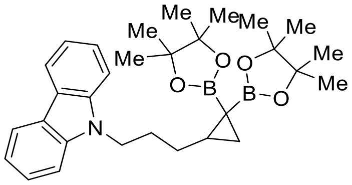

3s

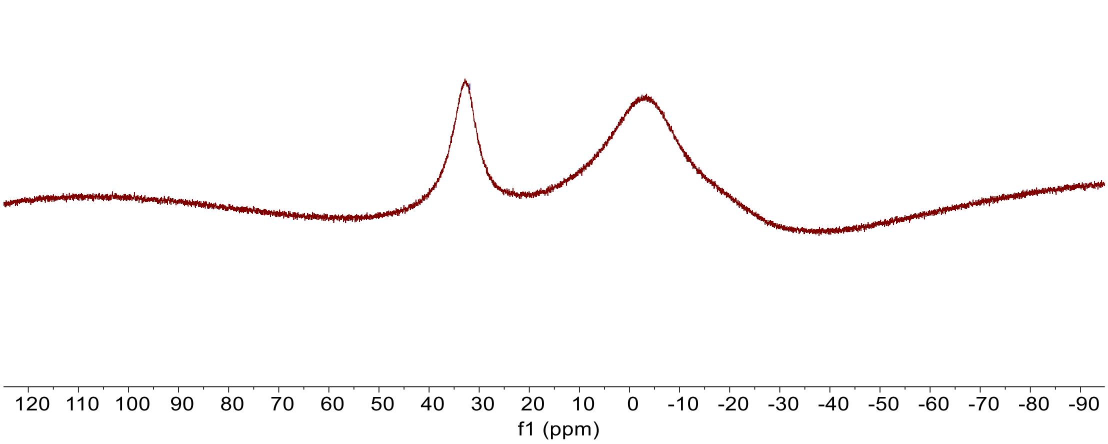

^{1}H NMR spectrum of compound **3t** in $CDCl_3$ (400 MHz).

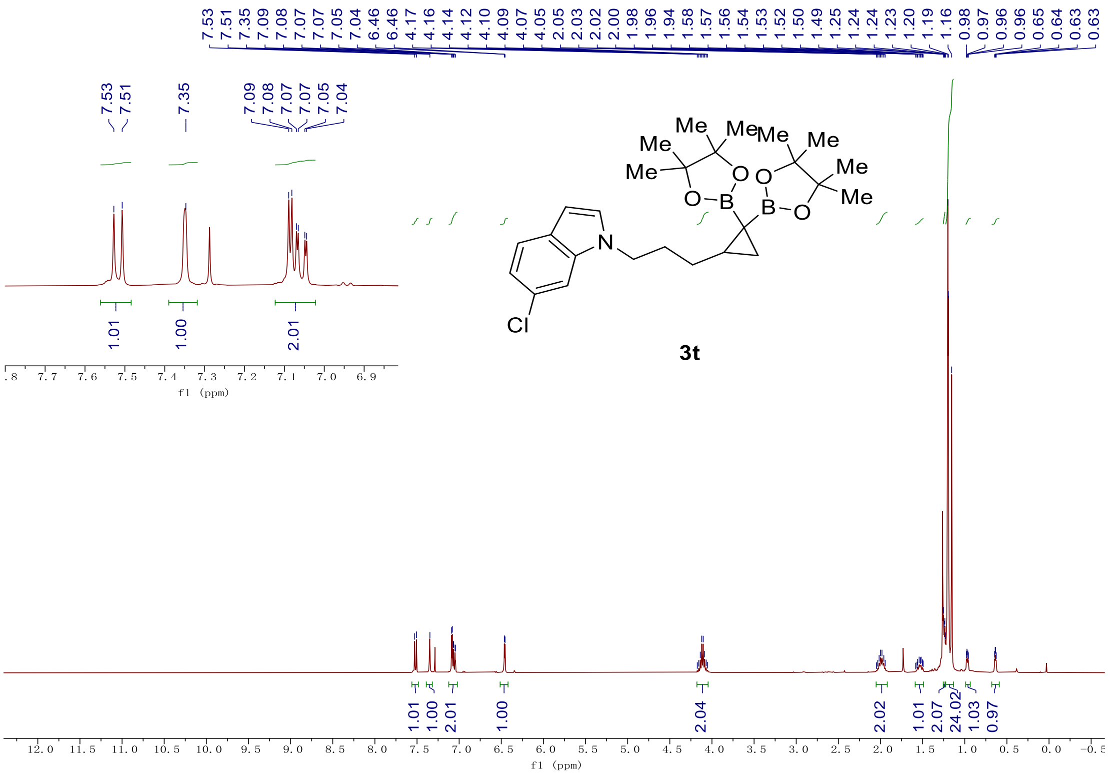

^{13}C NMR spectrum of compound **3t** in $CDCl_3$ (101 MHz).

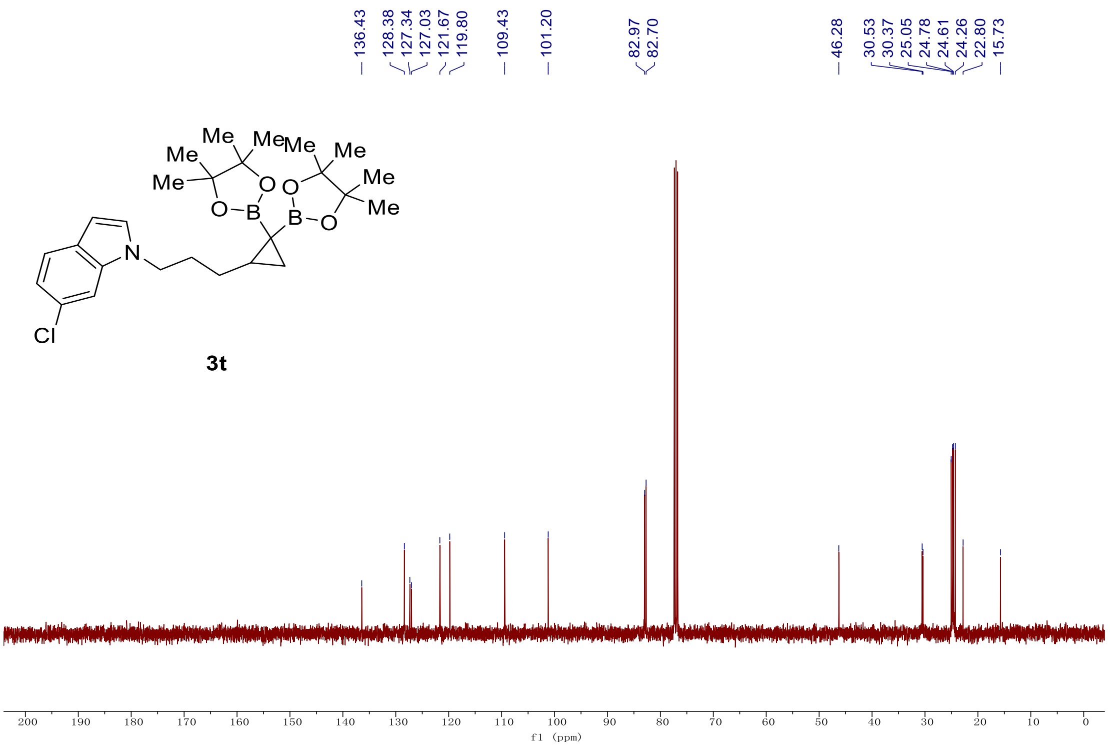

^{11}B NMR spectrum of compound **3t** in $CDCl_3$ (128 MHz).

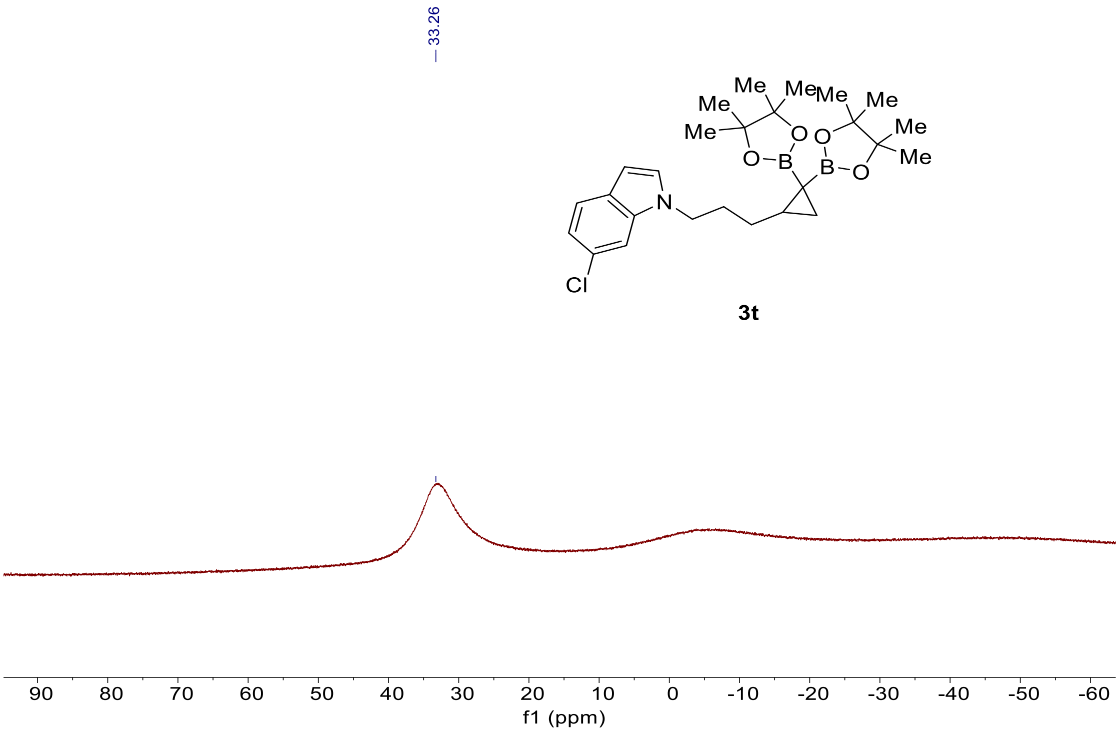

^{1}H NMR spectrum of compound **3u** in $CDCl_3$ (400 MHz).

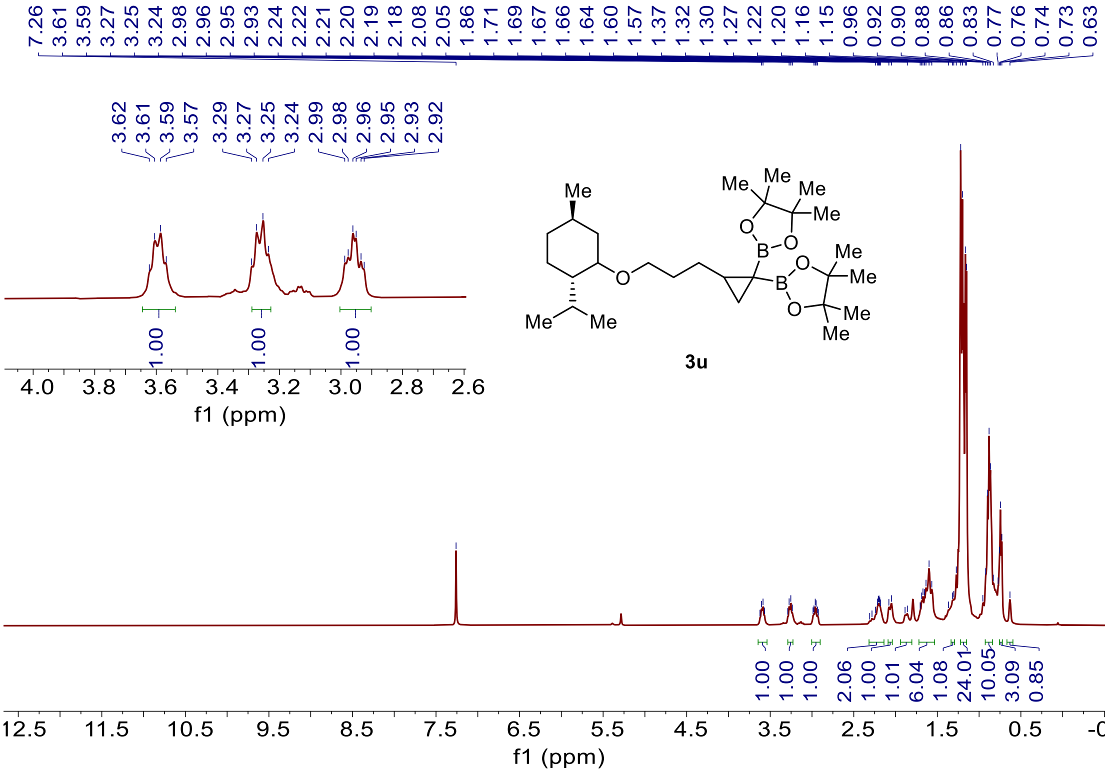

^{13}C NMR spectrum of compound **3u** in $CDCl_3$ (101 MHz).

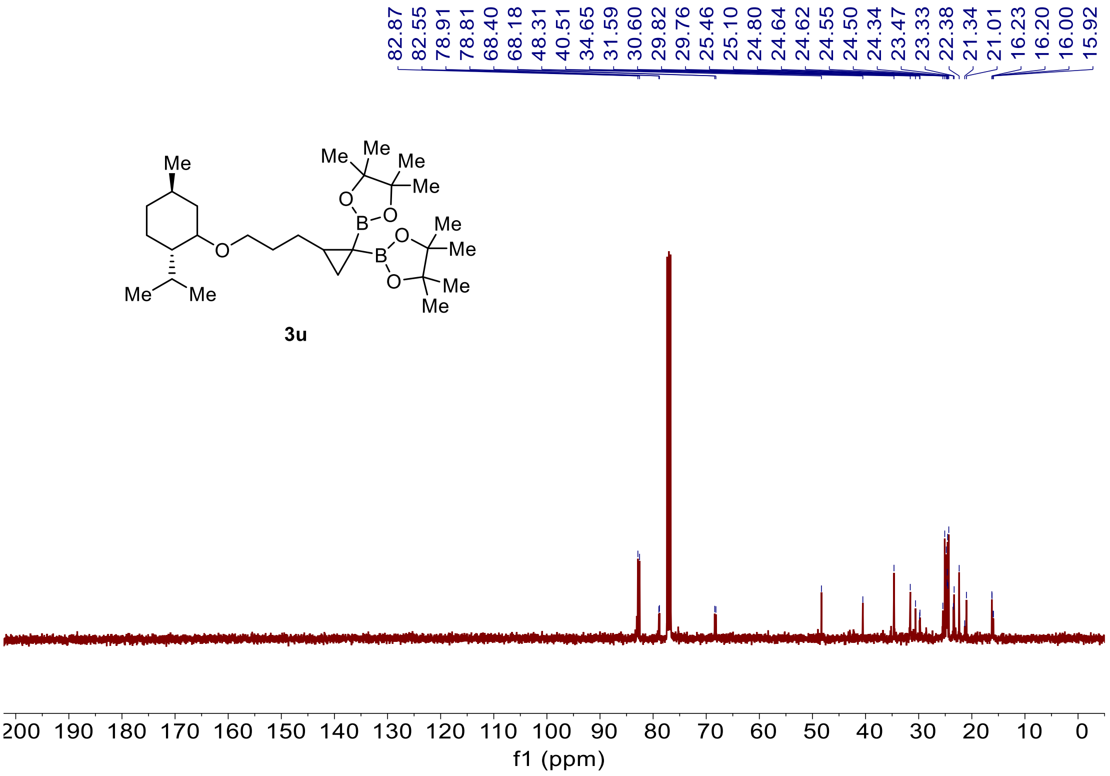

^{11}B NMR spectrum of compound **3u** in $CDCl_3$ (128 MHz).

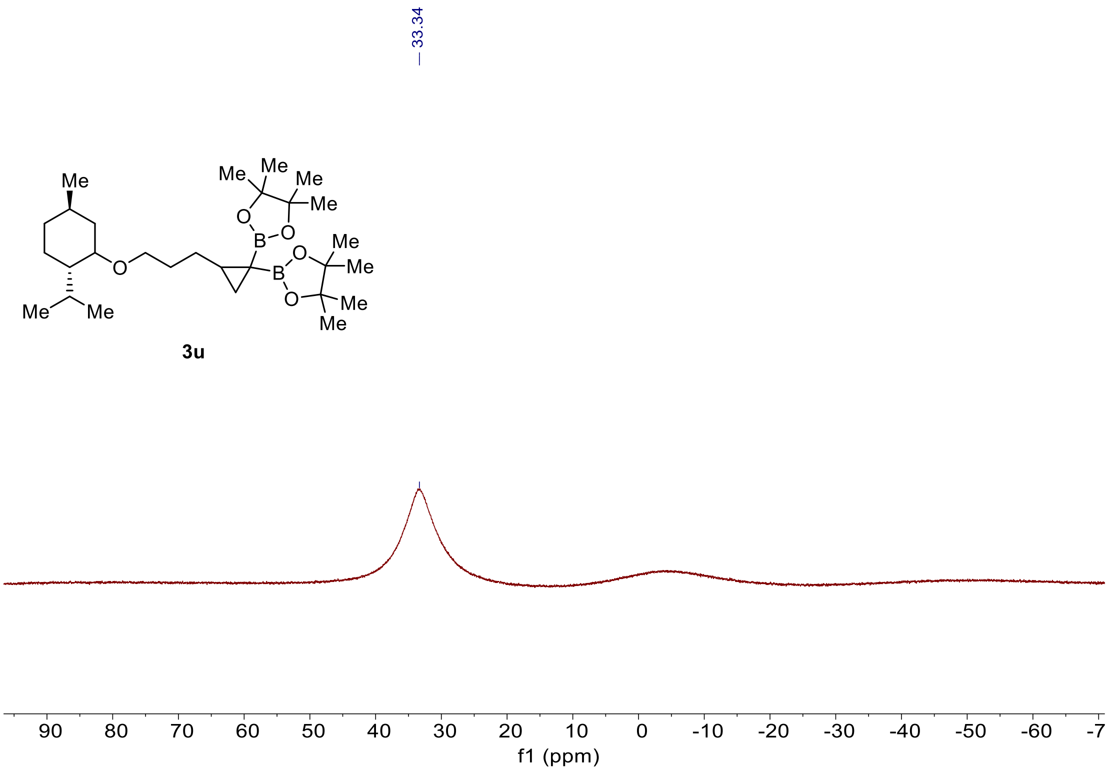

^{1}H NMR spectrum of compound **3v** in $CDCl_3$ (400 MHz).

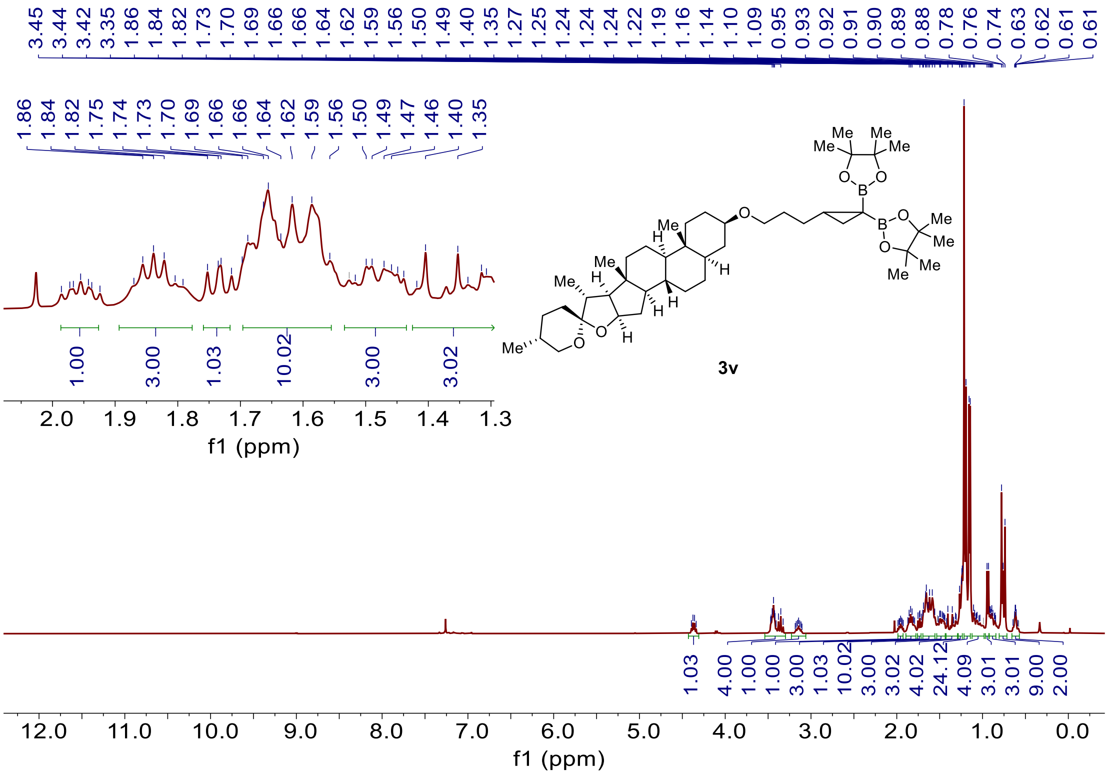

^{13}C NMR spectrum of compound **3v** in $CDCl_3$ (101 MHz).

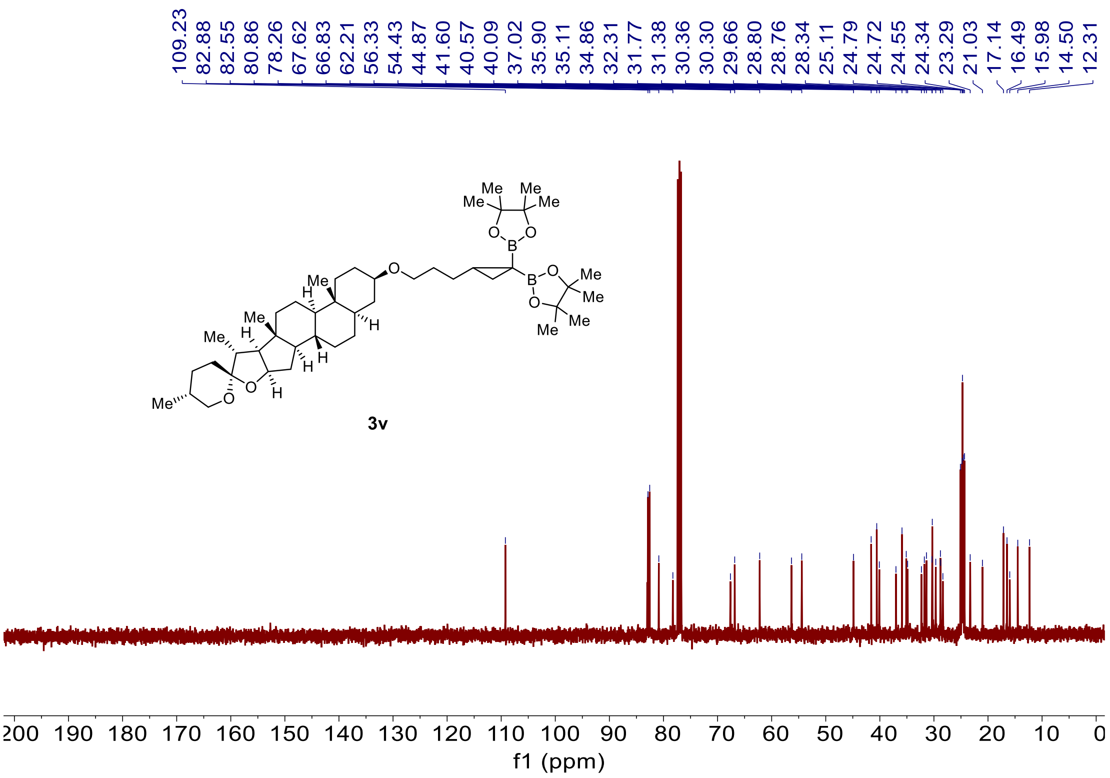

^{11}B NMR spectrum of compound **3v** in $CDCl_3$ (128 MHz).

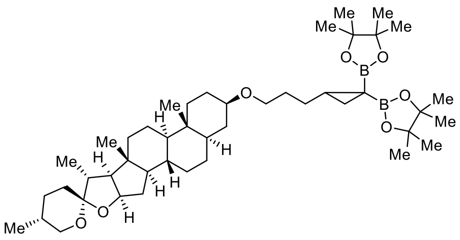

3v

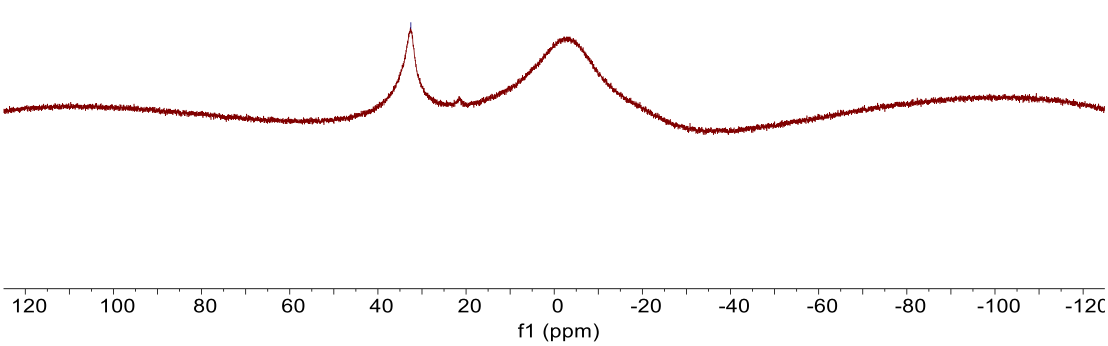

^{1}H NMR spectrum of compound **3w** in $CDCl_3$ (400 MHz).

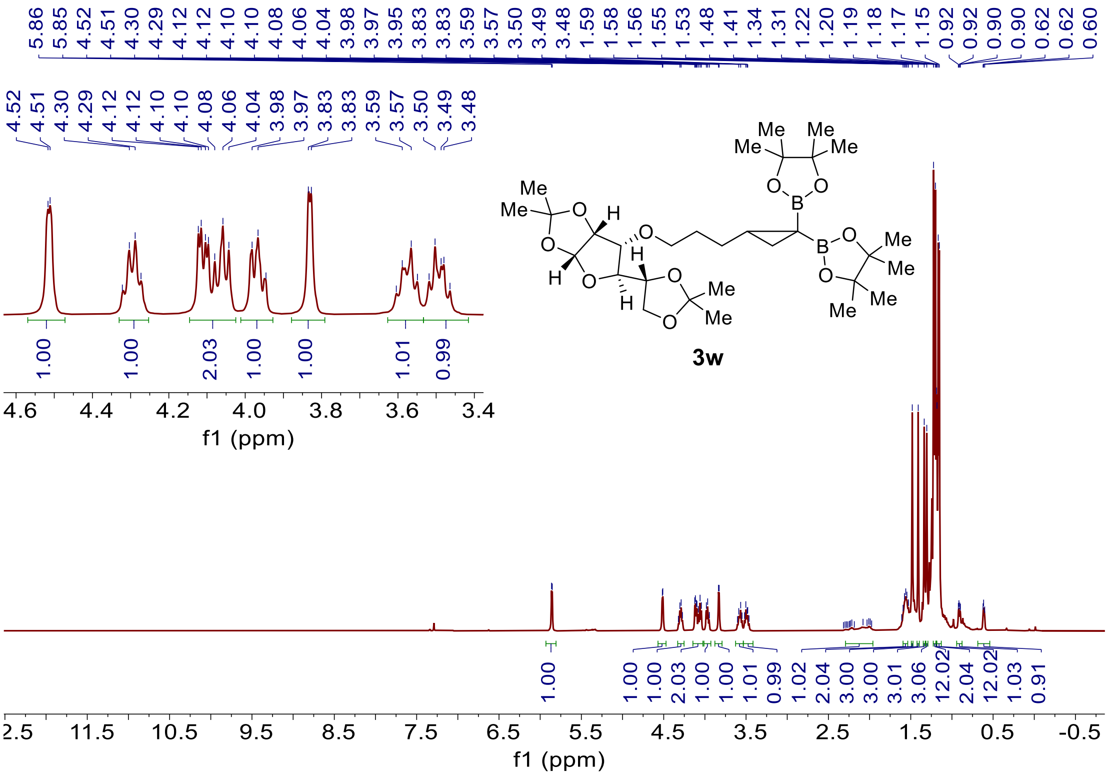

^{13}C NMR spectrum of compound **3w** in $CDCl_3$ (101 MHz).

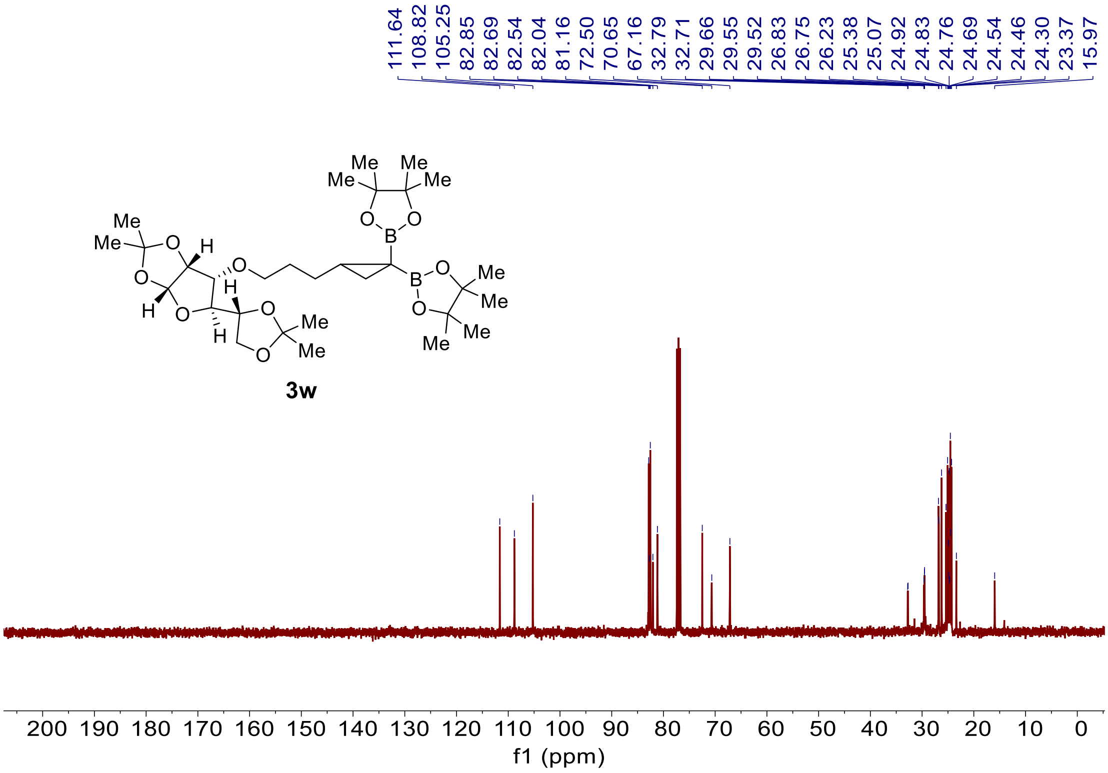

^{11}B NMR spectrum of compound **3w** in $CDCl_3$ (128 MHz).

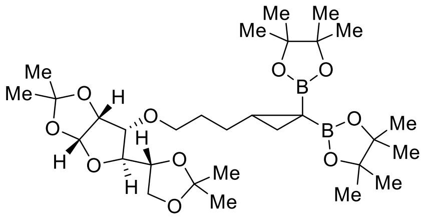

3w

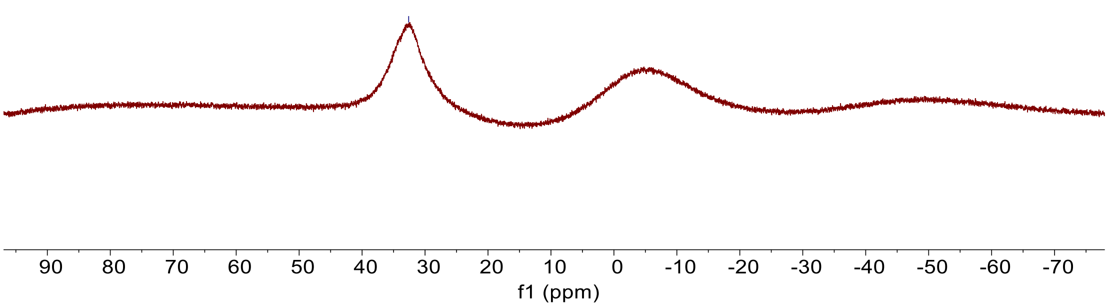

^{1}H NMR spectrum of compound **3x** in $CDCl_3$ (400 MHz).

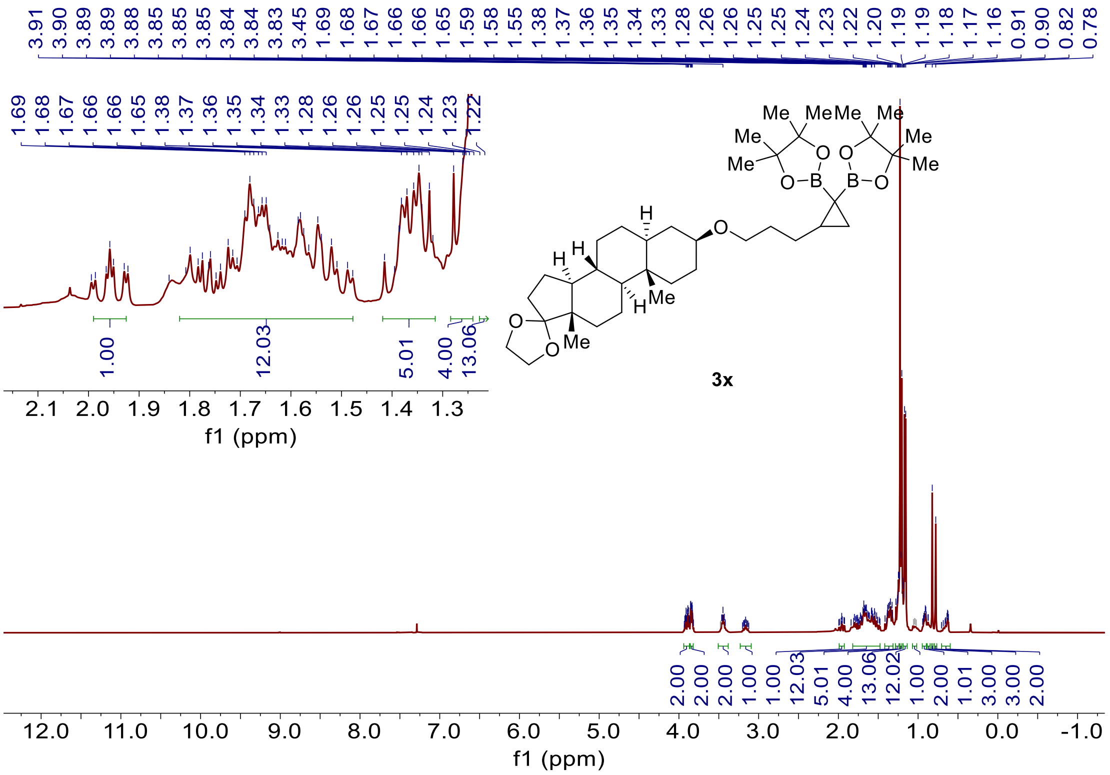

^{13}C NMR spectrum of compound **3x** in $CDCl_3$ (101 MHz).

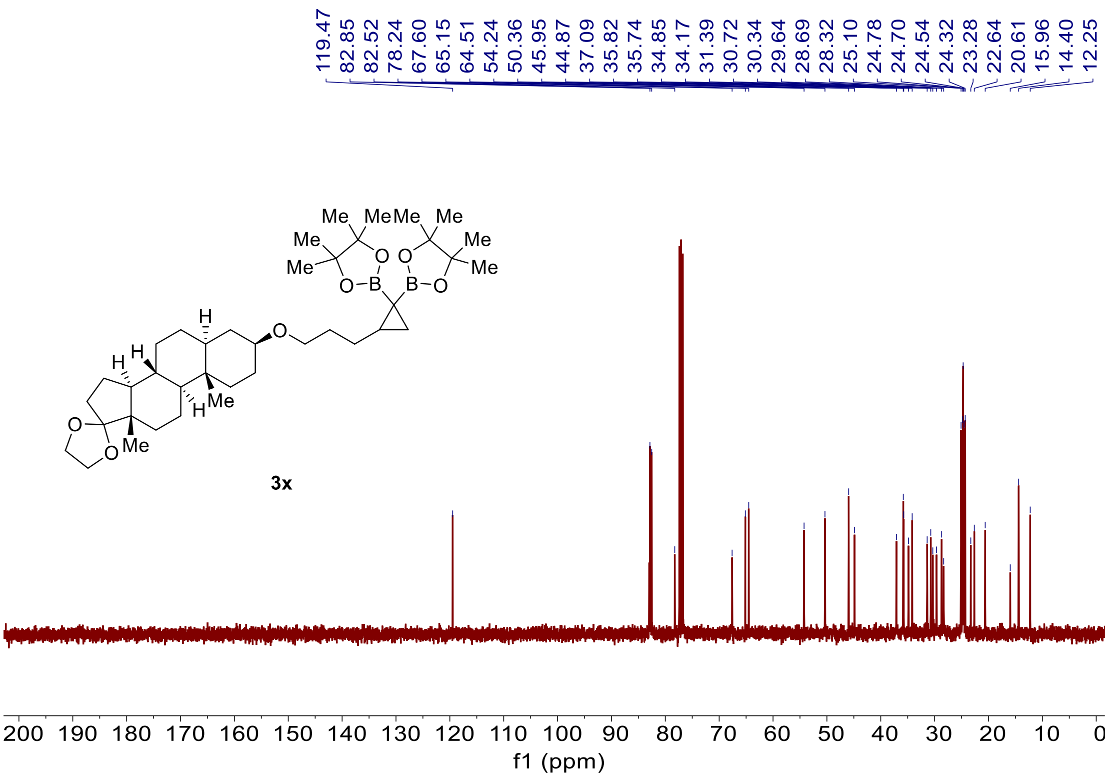

^{11}B NMR spectrum of compound **3x** in $CDCl_3$ (128 MHz).

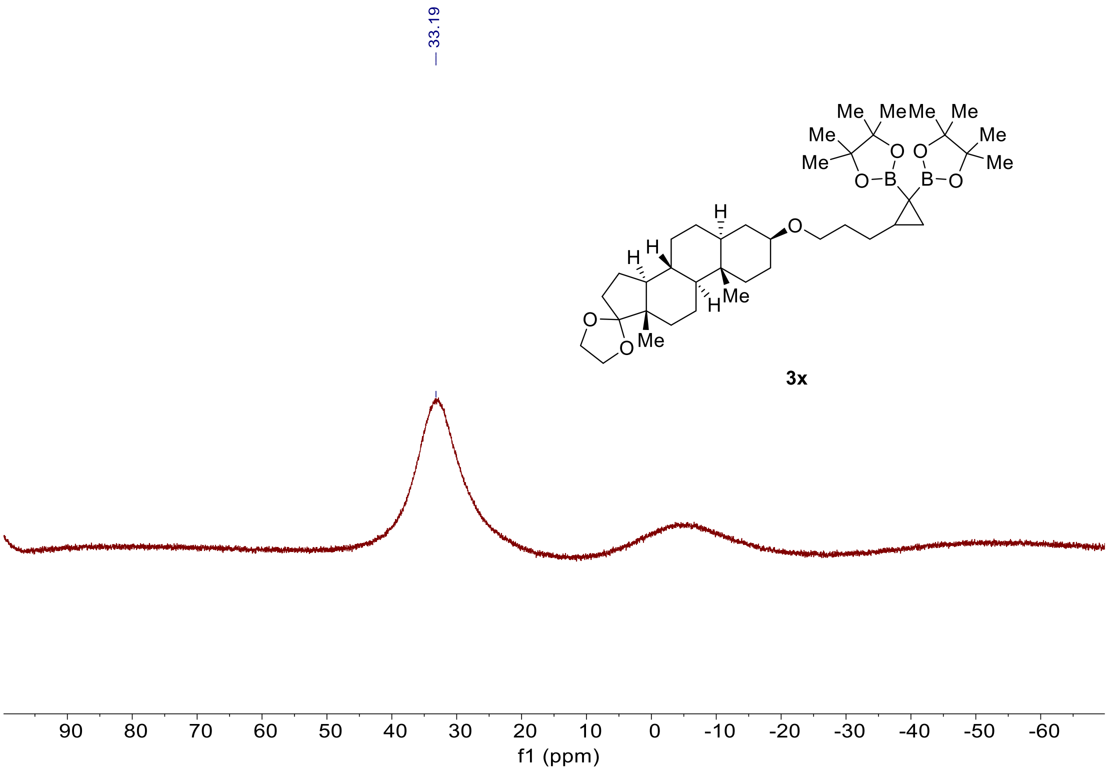

^{1}H NMR spectrum of compound **3y** in $CDCl_3$ (400 MHz).

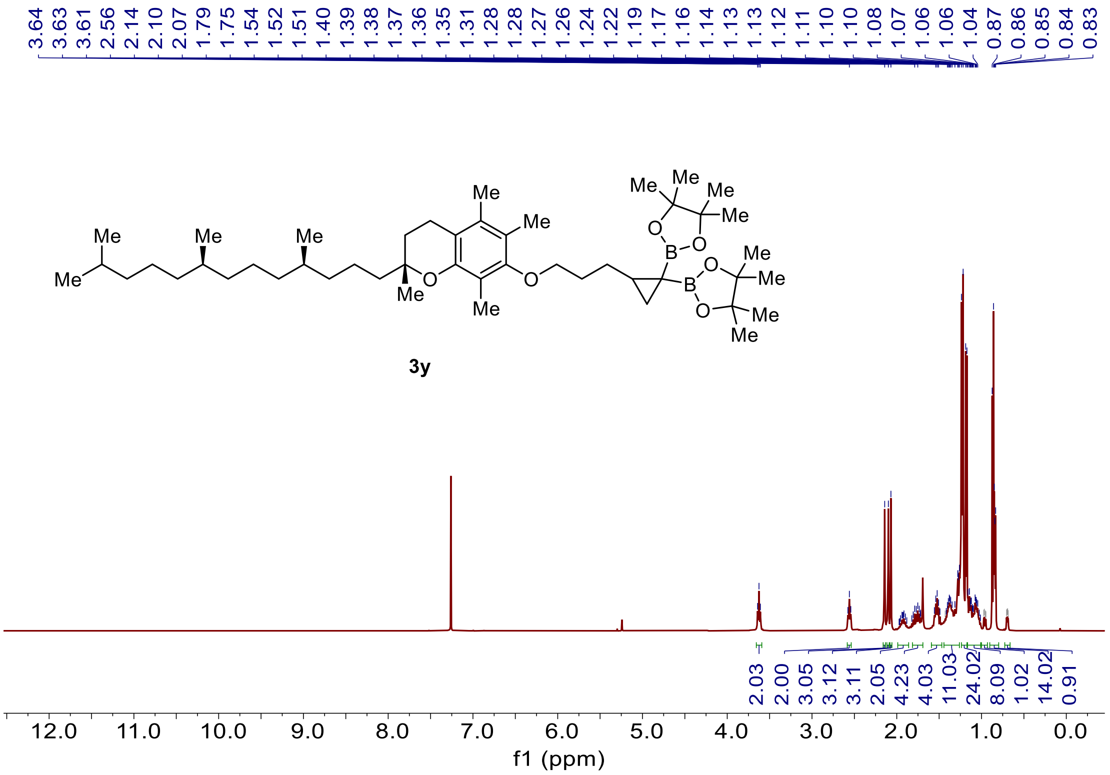

^{13}C NMR spectrum of compound **3y** in $CDCl_3$ (101 MHz).

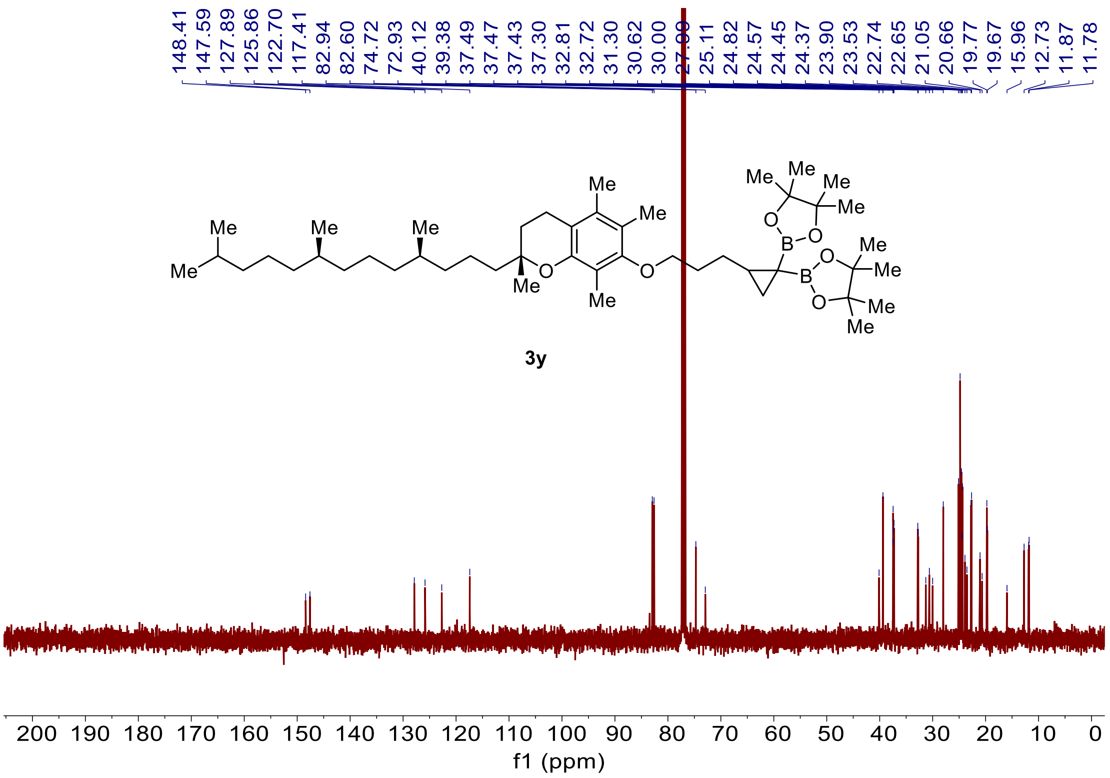

^{11}B NMR spectrum of compound **3y** in $CDCl_3$ (128 MHz).

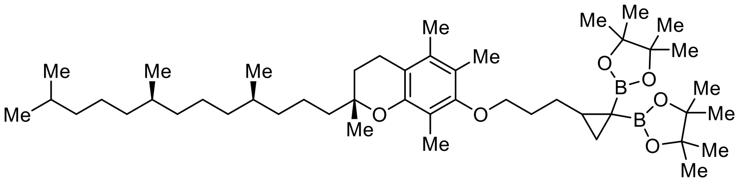

3y

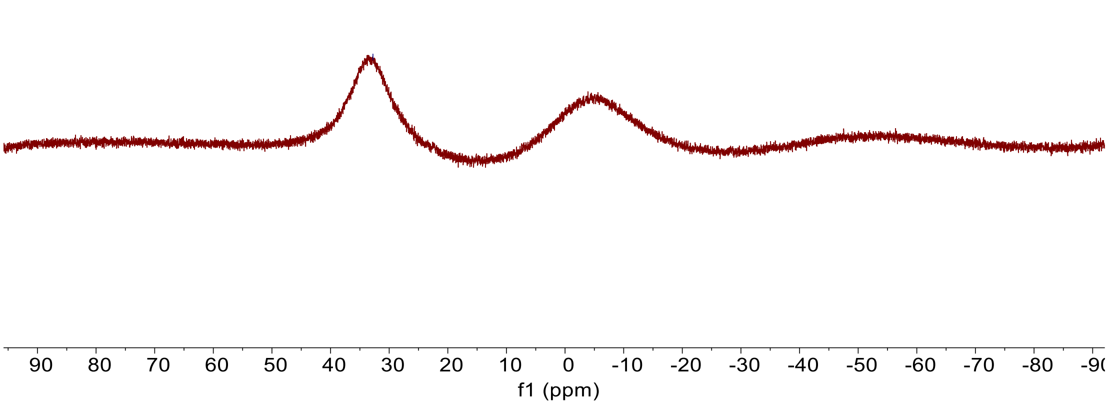

^{1}H NMR spectrum of compound **3z** in $CDCl_3$ (400 MHz).

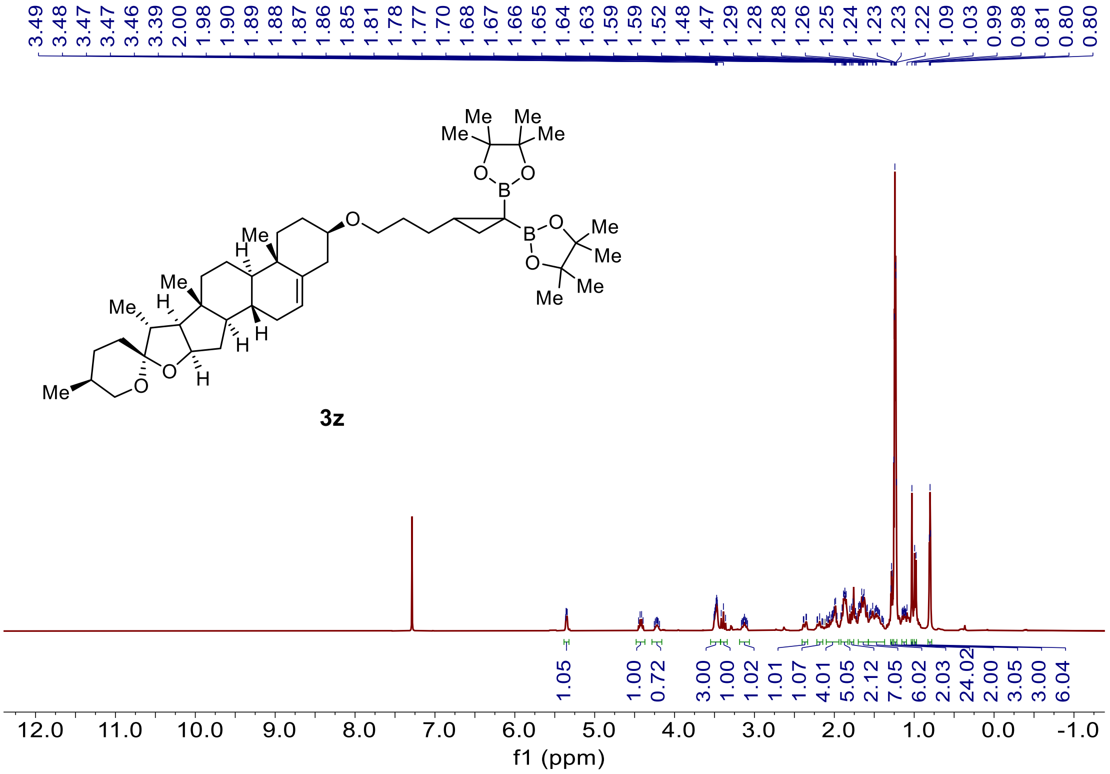

^{13}C NMR spectrum of compound **3z** in $CDCl_3$ (101 MHz).

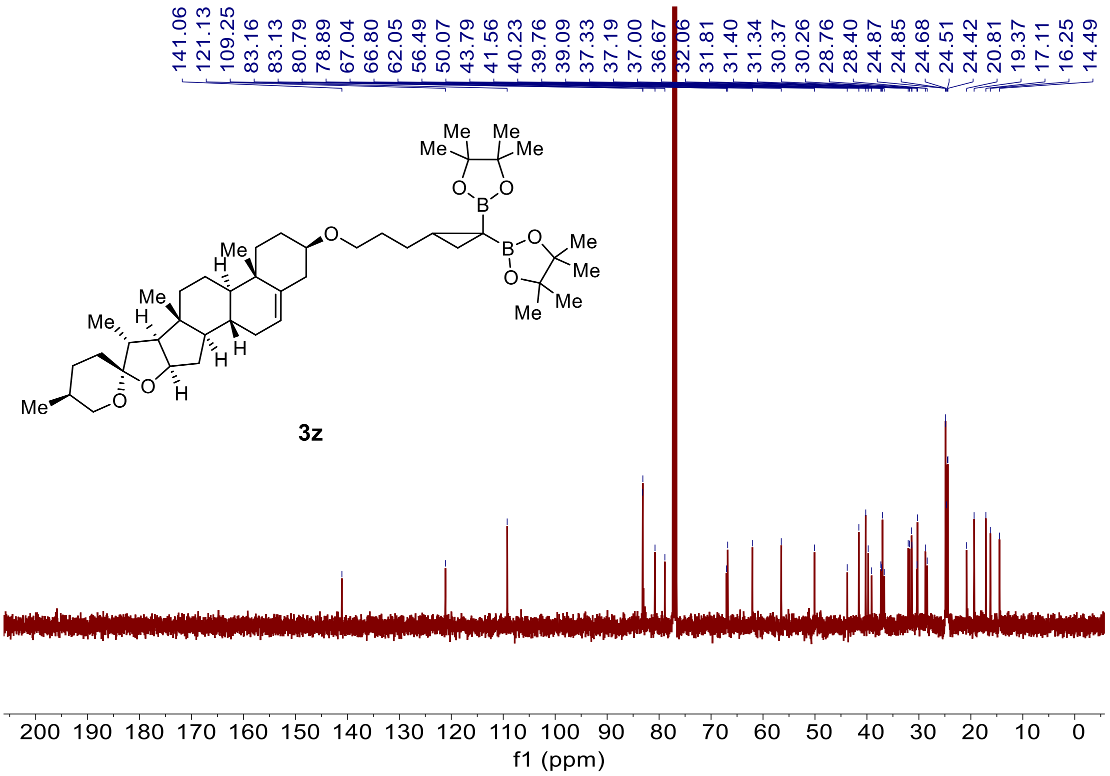

^{11}B NMR spectrum of compound **3z** in $CDCl_3$ (128 MHz).

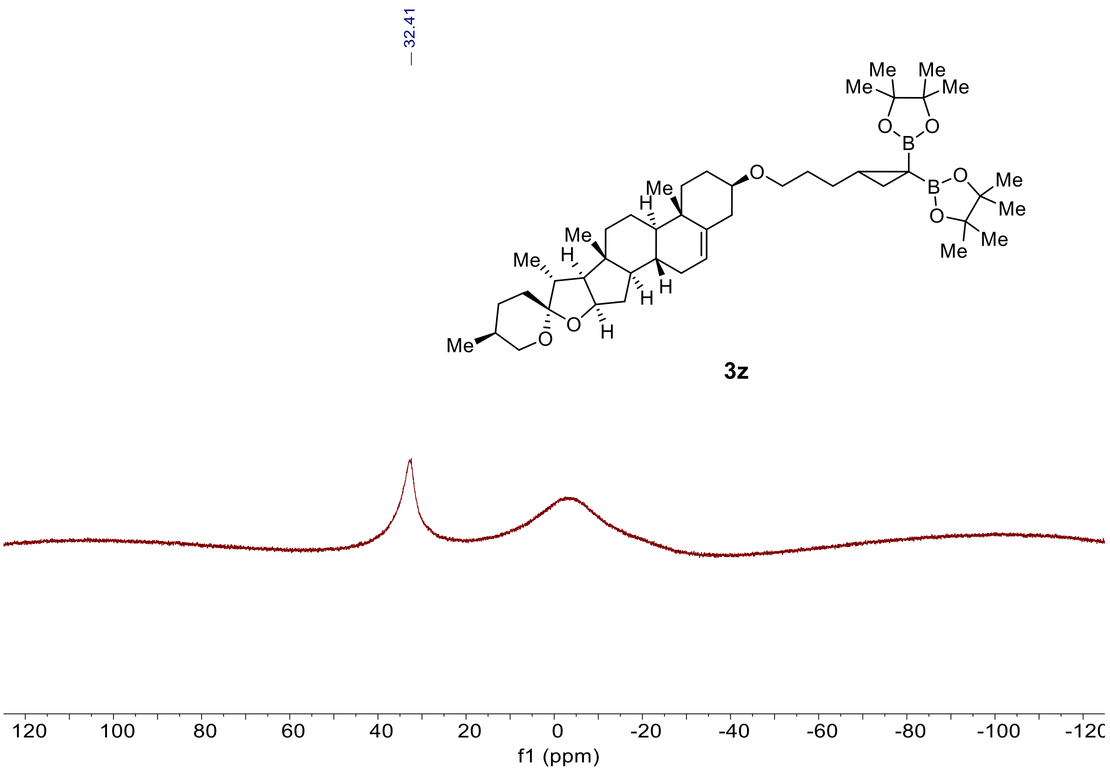

3z

^{1}H NMR spectrum of compound **3'a** in $CDCl_3$ (400 MHz).

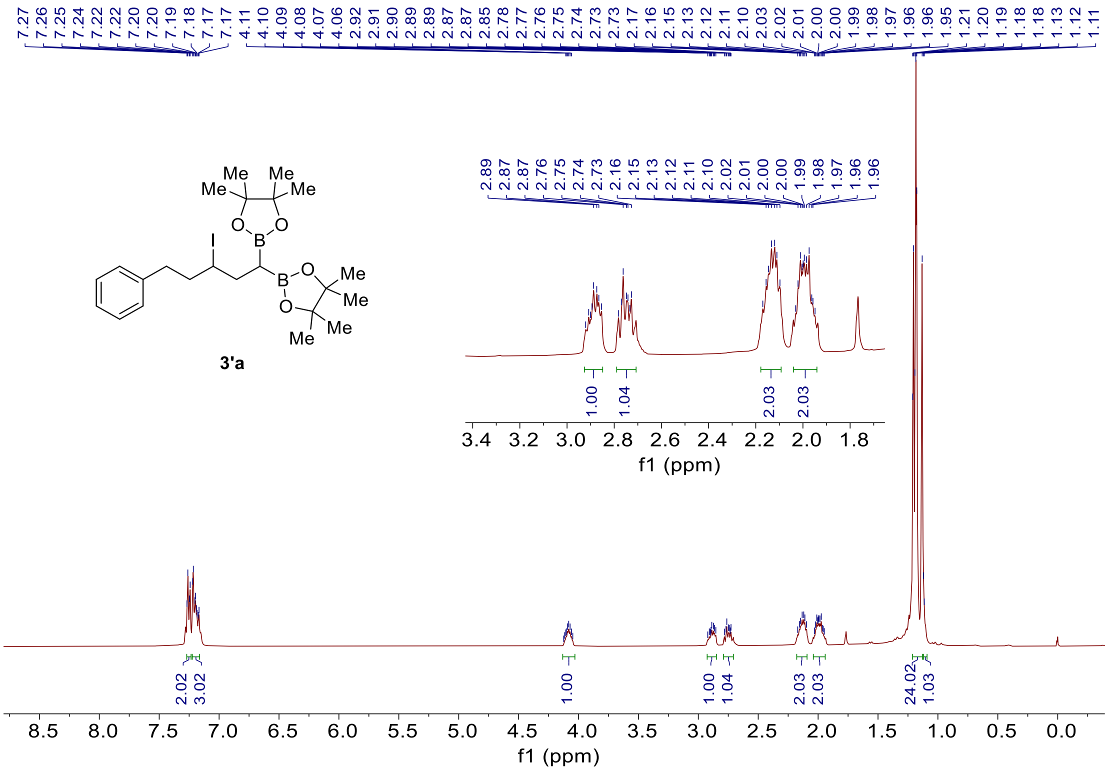

^{13}C NMR spectrum of compound **3'a** in $CDCl_3$ (101 MHz).

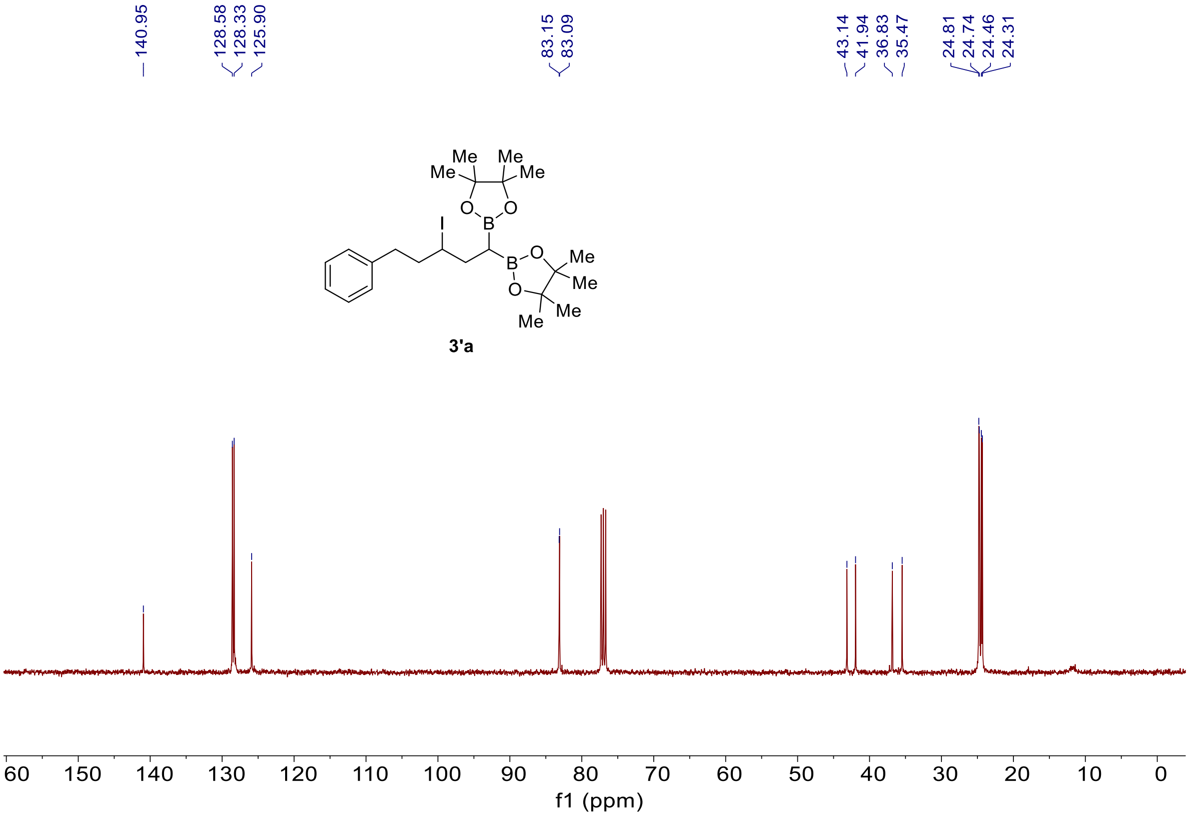

^{11}B NMR spectrum of compound **3'a** in $CDCl_3$ (128 MHz).

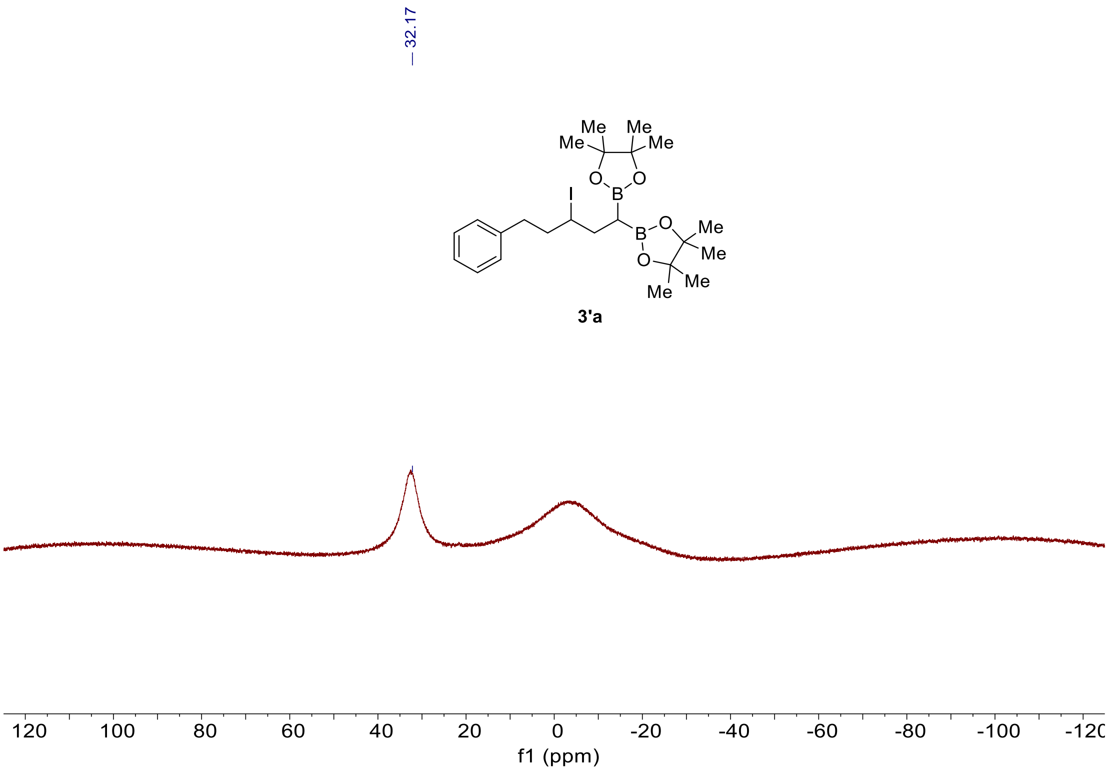

^{1}H NMR spectrum of compound **5** in $CDCl_3$ (300 MHz).

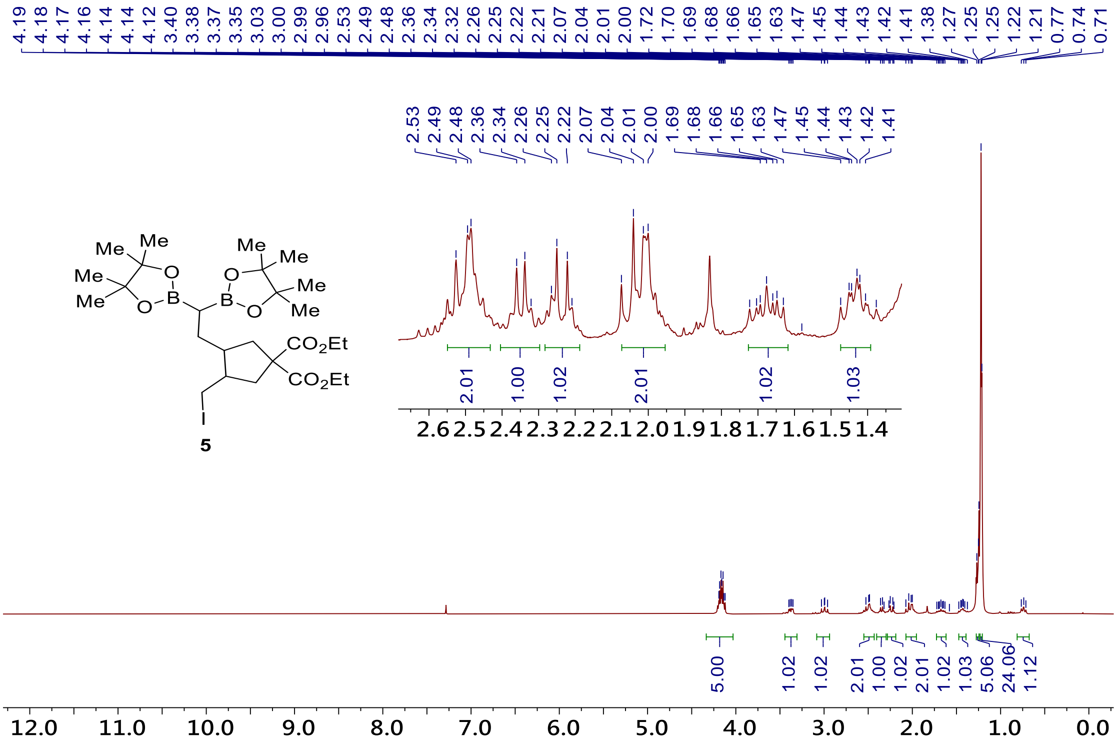

^{13}C NMR spectrum of compound **5** in $CDCl_3$ (75 MHz).

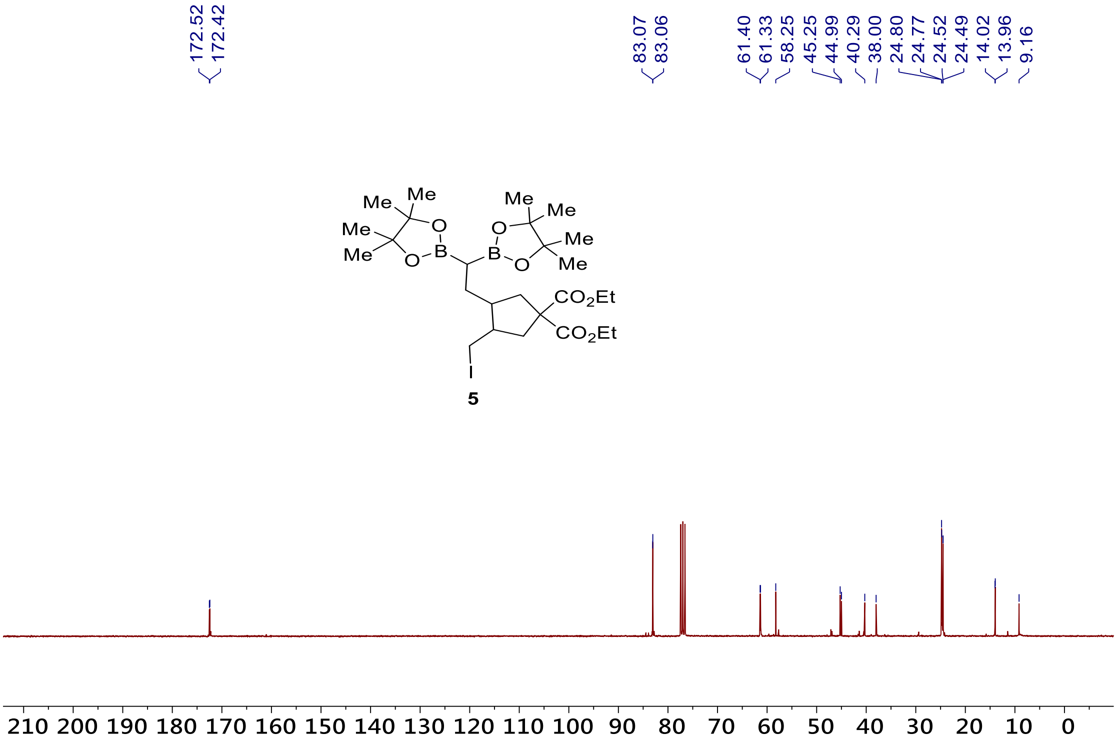

^{11}B NMR spectrum of compound **5** in $CDCl_3$ (96 MHz).

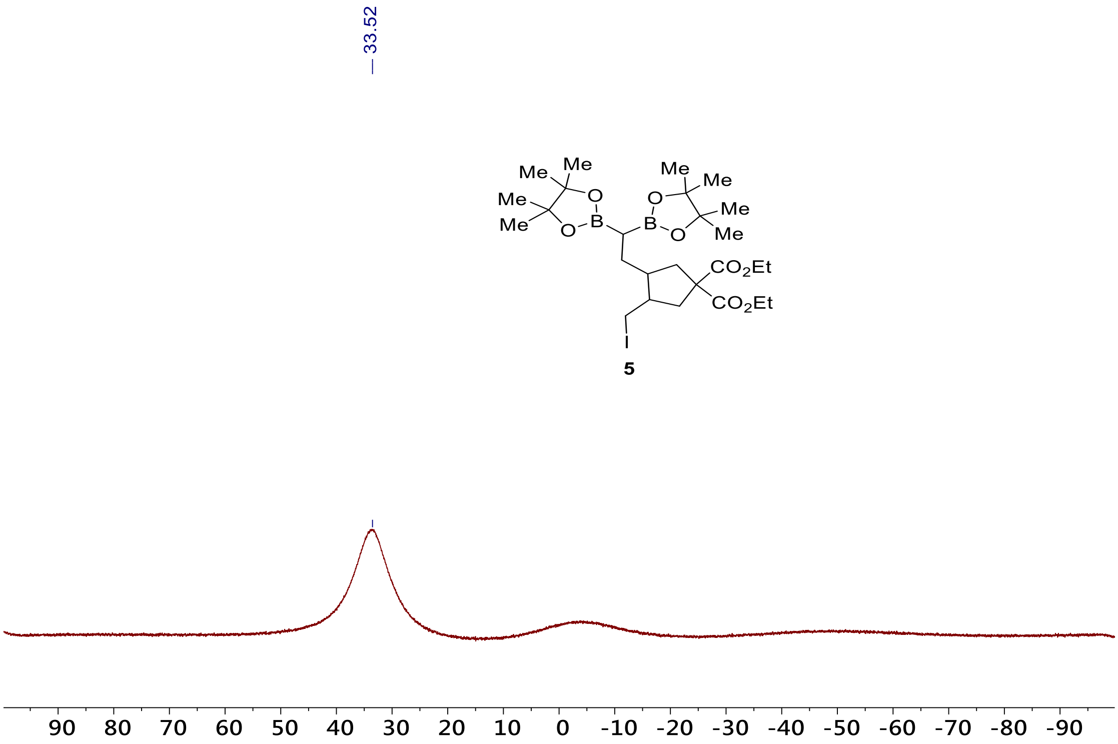

^{1}H NMR spectrum of compound **5a** in $CDCl_3$ (300 MHz).

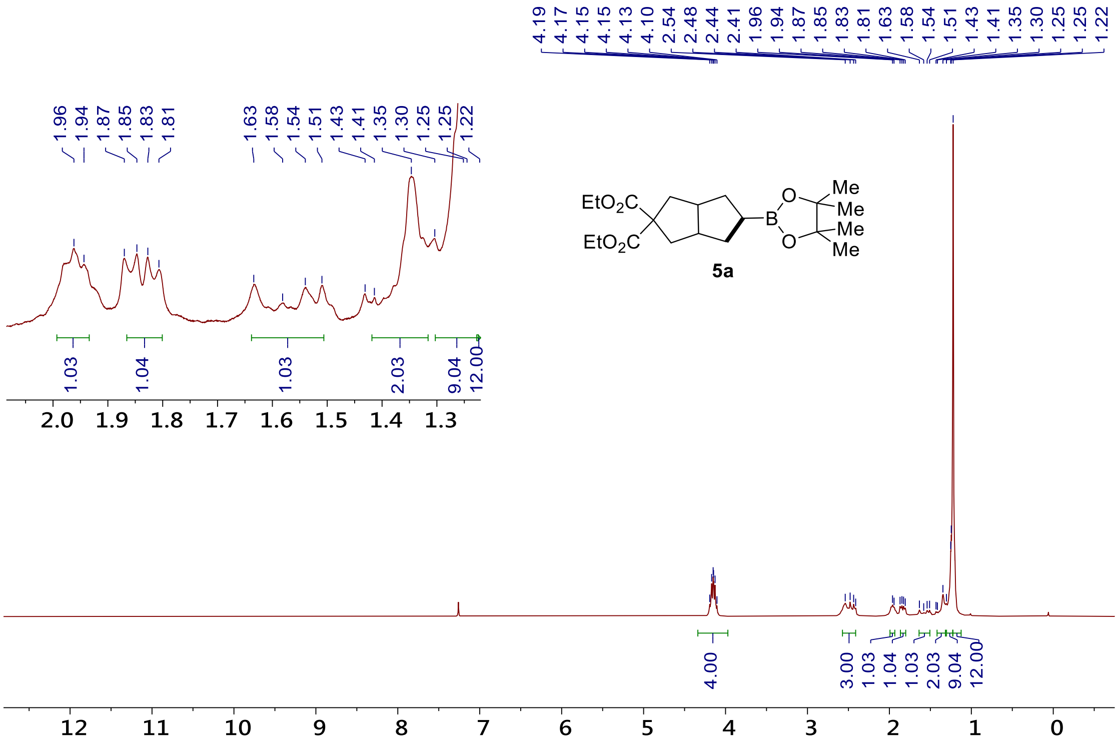

^{13}C NMR spectrum of compound **5a** in $CDCl_3$ (75 MHz).

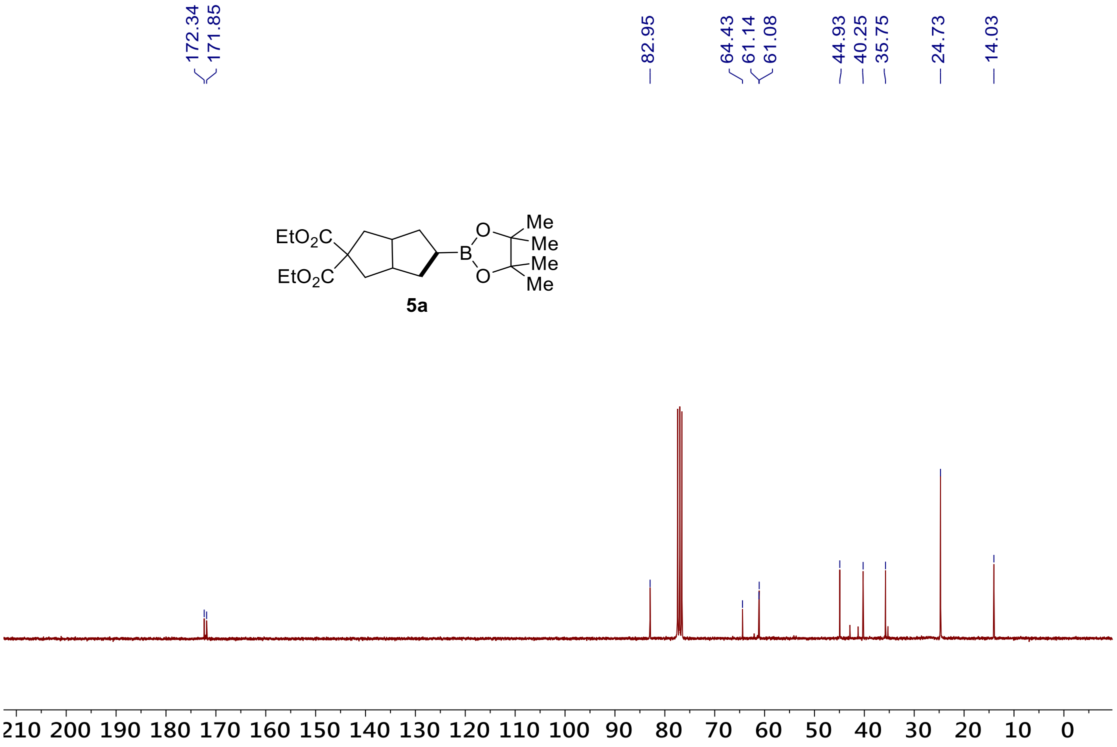

^{11}B NMR spectrum of compound **5a** in $CDCl_3$ (96 MHz).

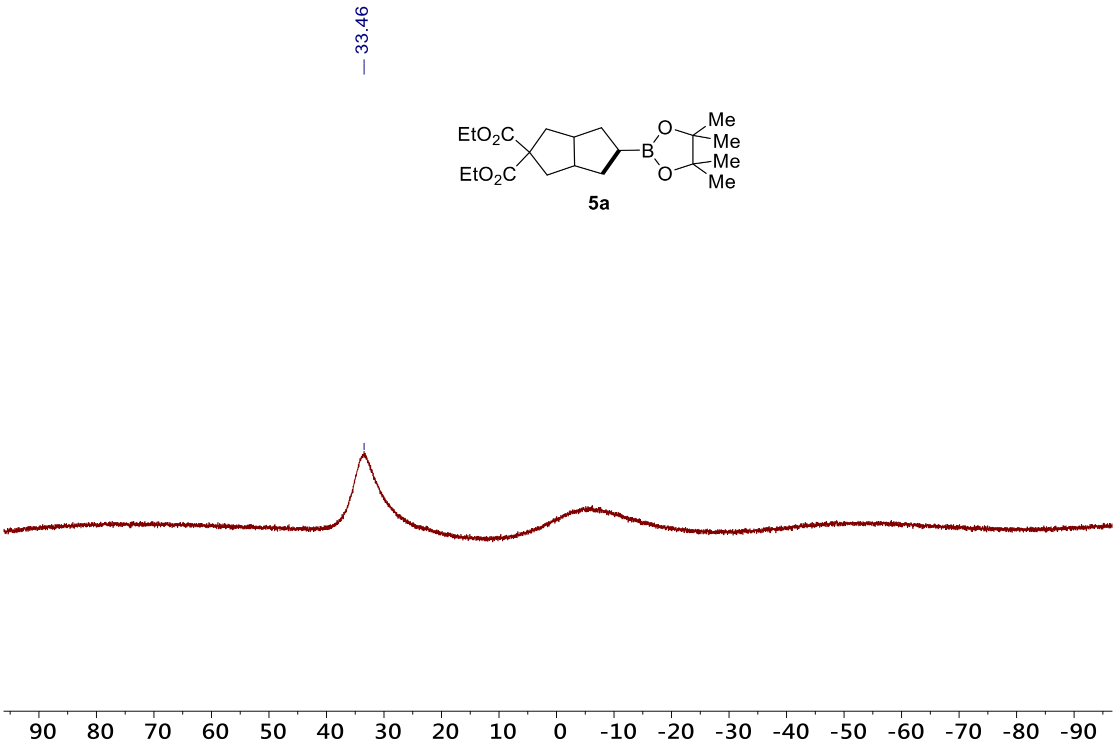

^{1}H NMR spectrum of compound **7** in $CDCl_3$ (300 MHz).

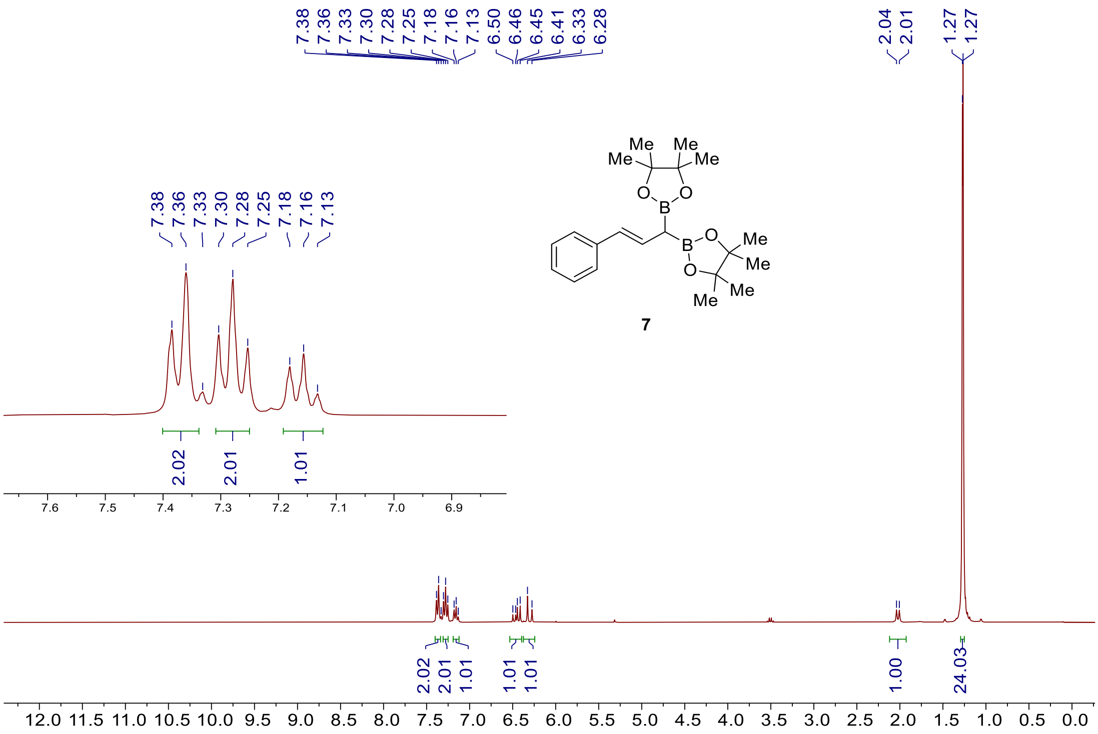

^{13}C NMR spectrum of compound **7** in $CDCl_3$ (75 MHz).

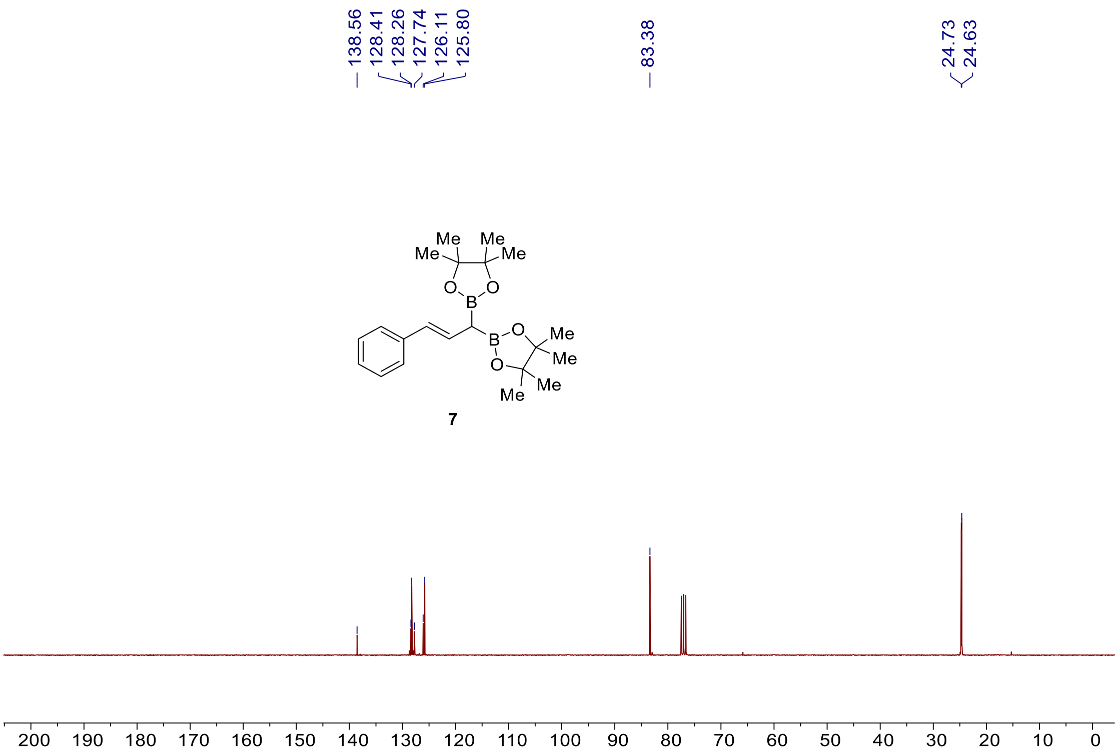

^{11}B NMR spectrum of compound **7** in $CDCl_3$ (96 MHz).

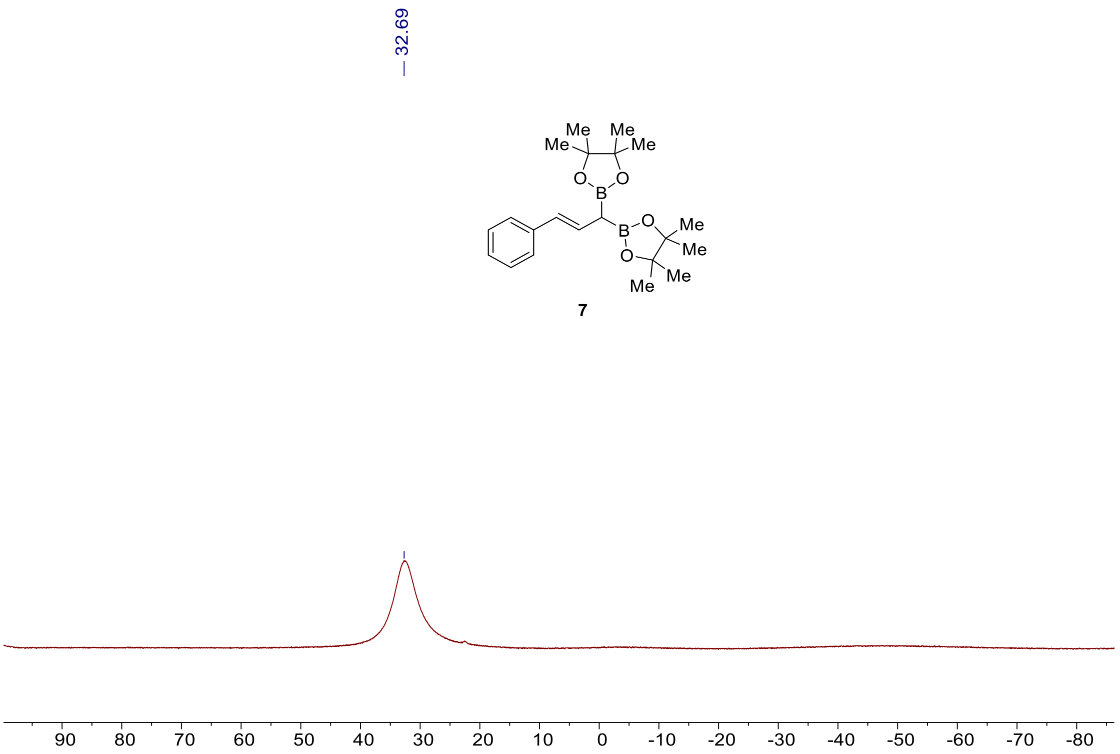

^{1}H NMR spectrum of compound **8** in $CDCl_3$ (300 MHz).

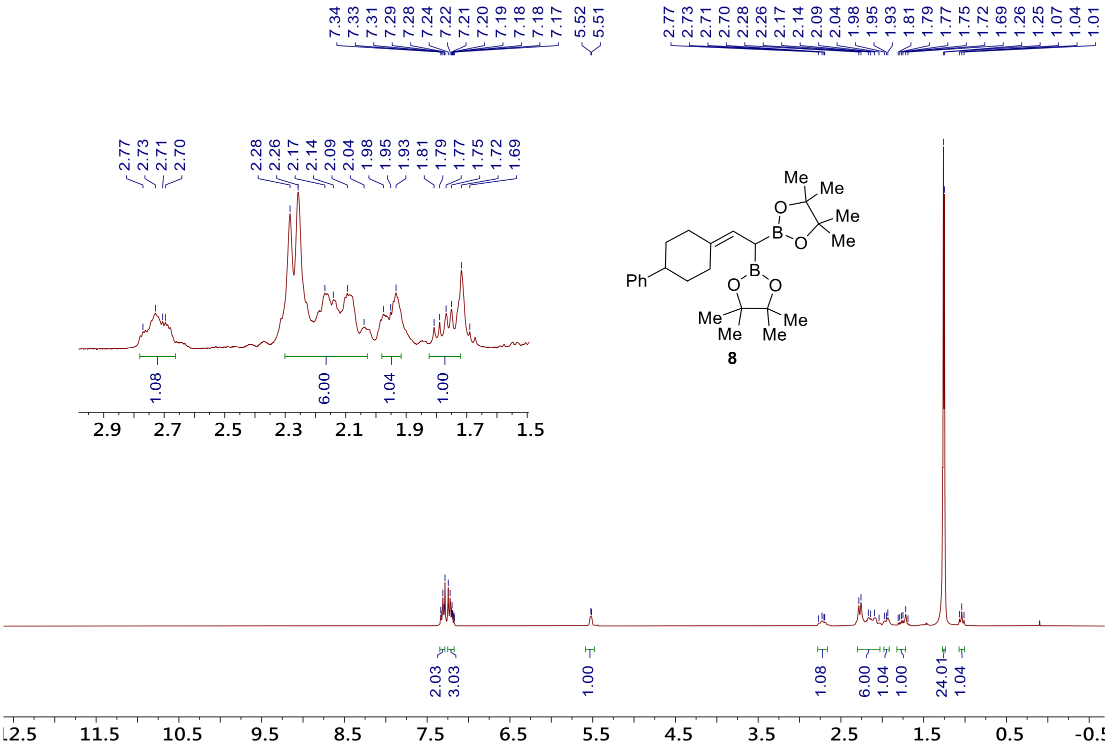

^{13}C NMR spectrum of compound **8** in $CDCl_3$ (75 MHz).

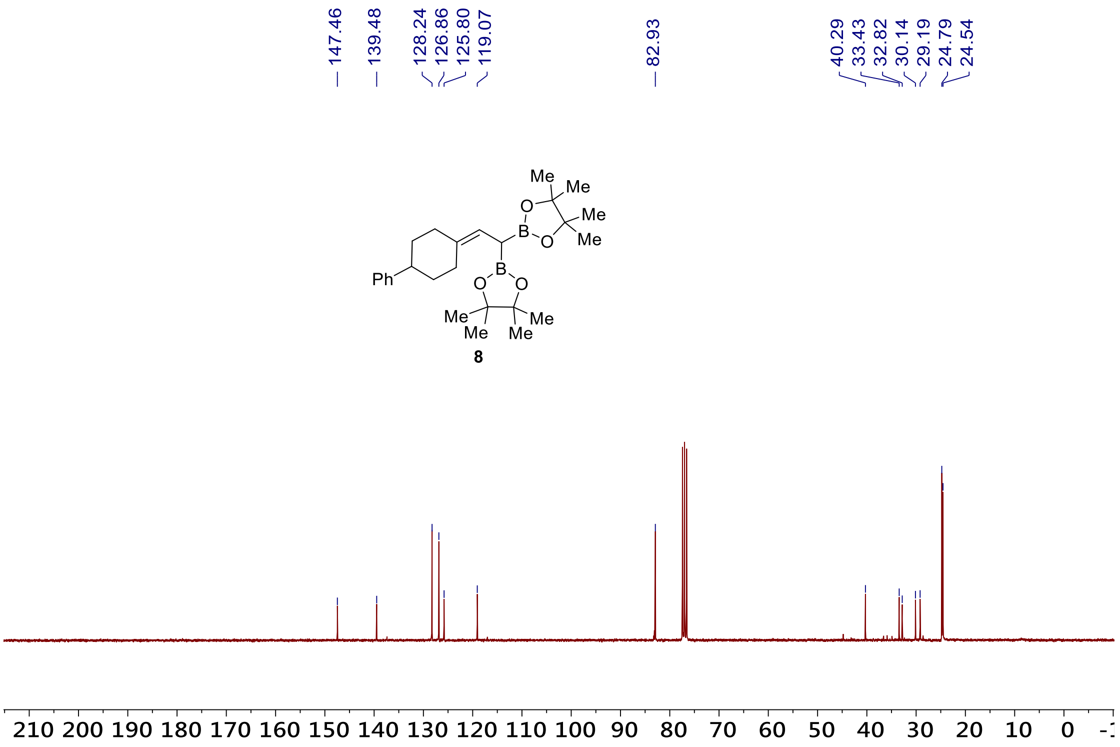

^{11}B NMR spectrum of compound **8** in $CDCl_3$ (96 MHz).

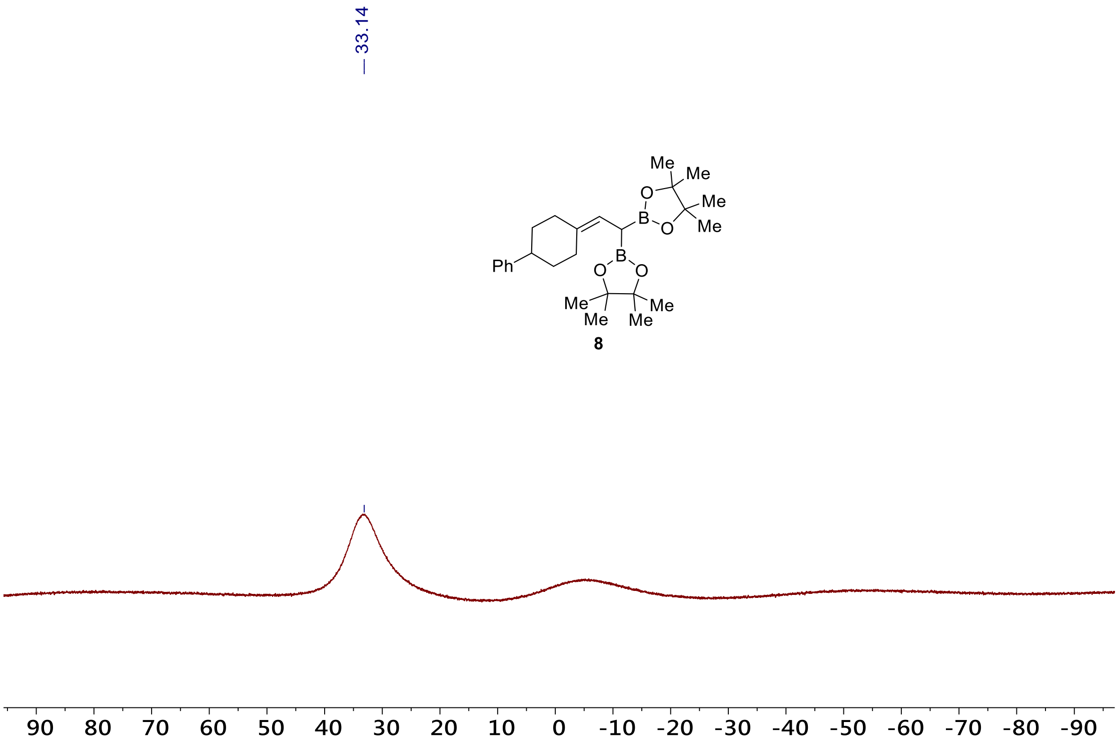

^{1}H NMR spectrum of compound **3'b** in $CDCl_3$ (400 MHz).

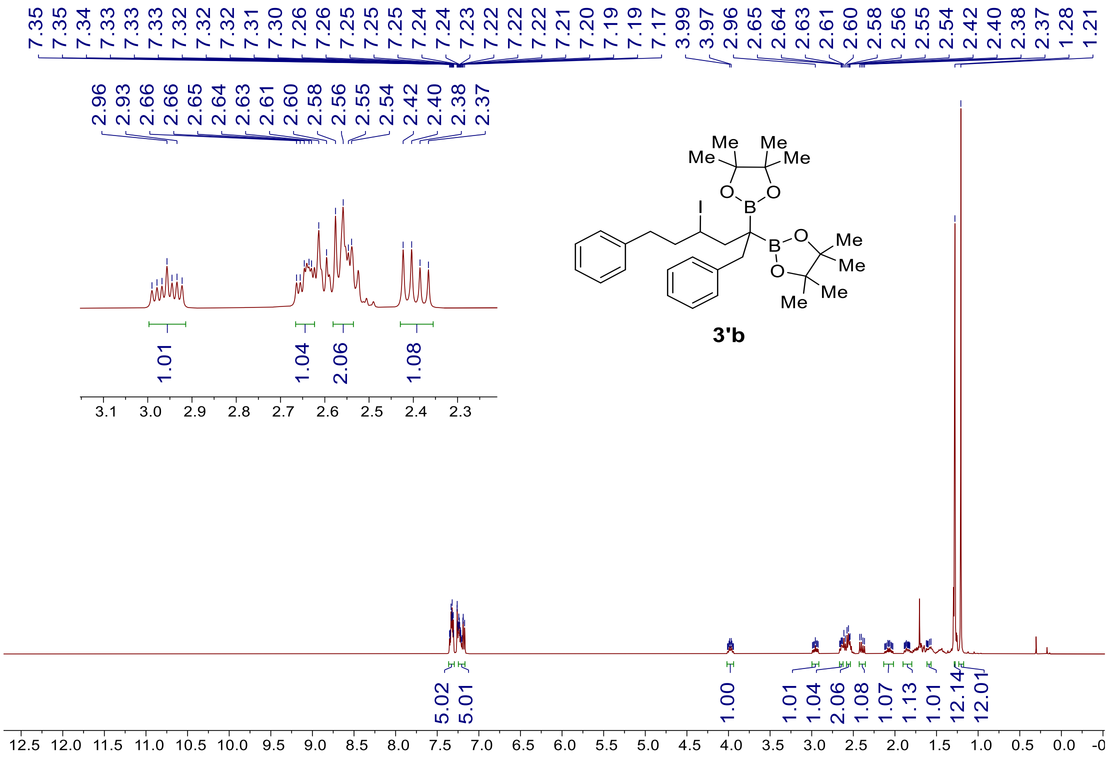

^{13}C NMR spectrum of compound **3'b** in $CDCl_3$ (101 MHz).

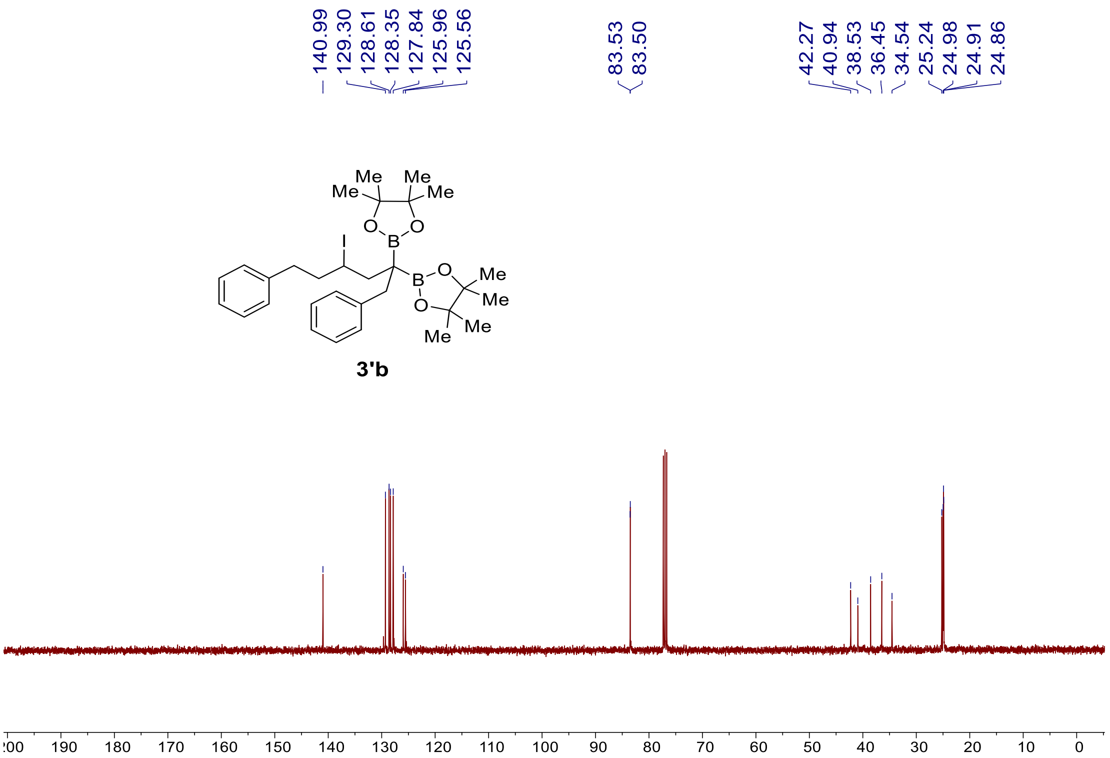

^{11}B NMR spectrum of compound **3'b** in $CDCl_3$ (128 MHz).

— 33.39

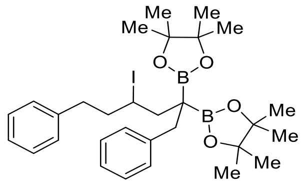

3'b

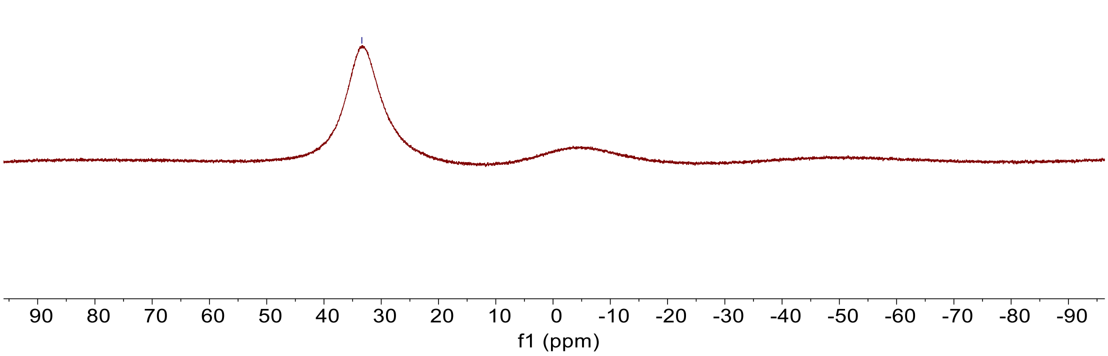

^{1}H NMR spectrum of compound **3'c** in $CDCl_3$ (400 MHz).

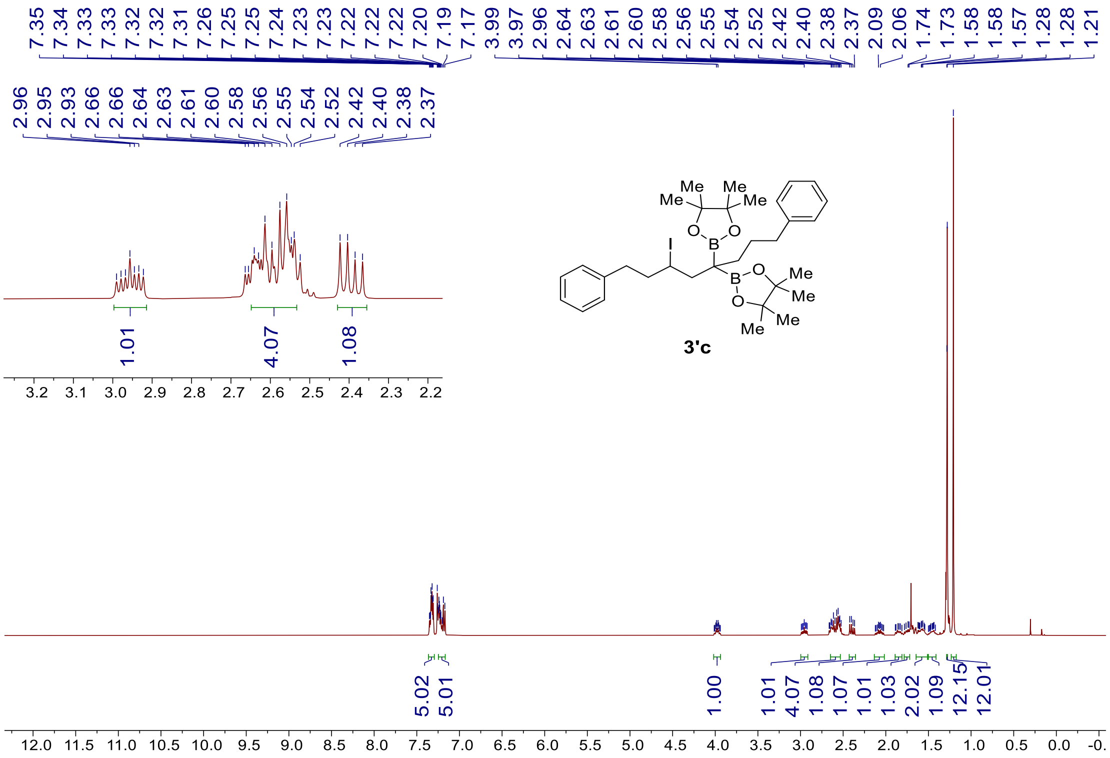

^{13}C NMR spectrum of compound **3'c** in $CDCl_3$ (101 MHz).

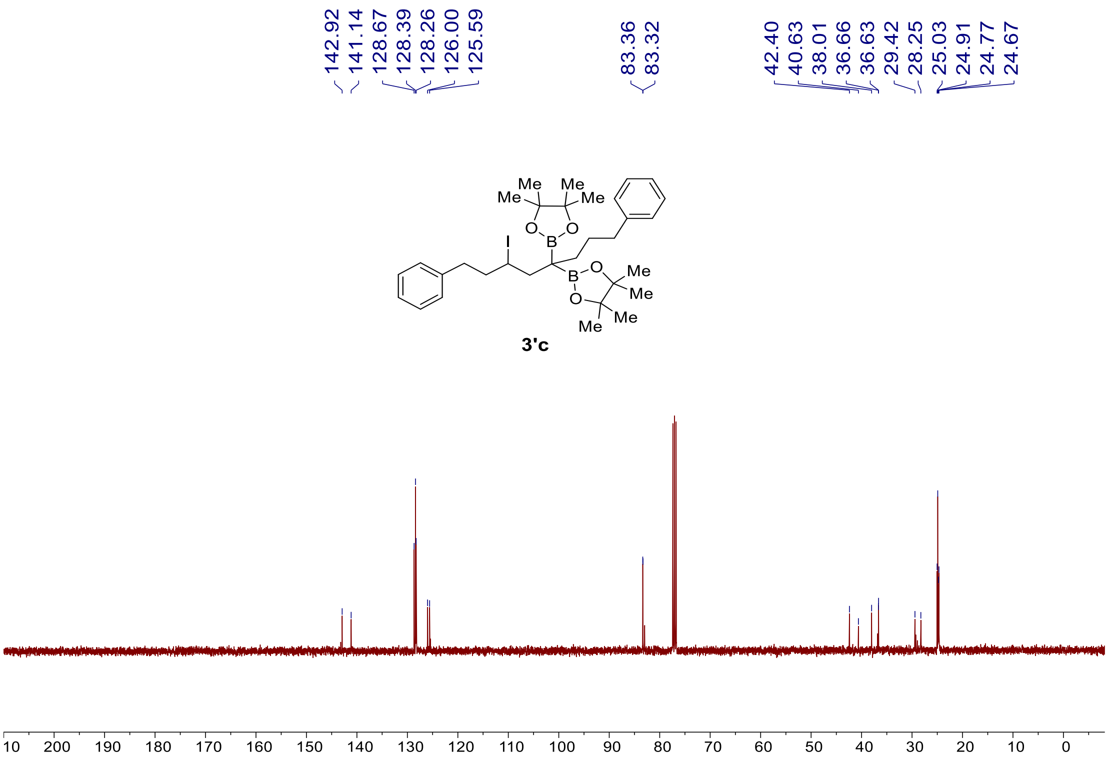

^{11}B NMR spectrum of compound **3'c** in $CDCl_3$ (128 MHz).

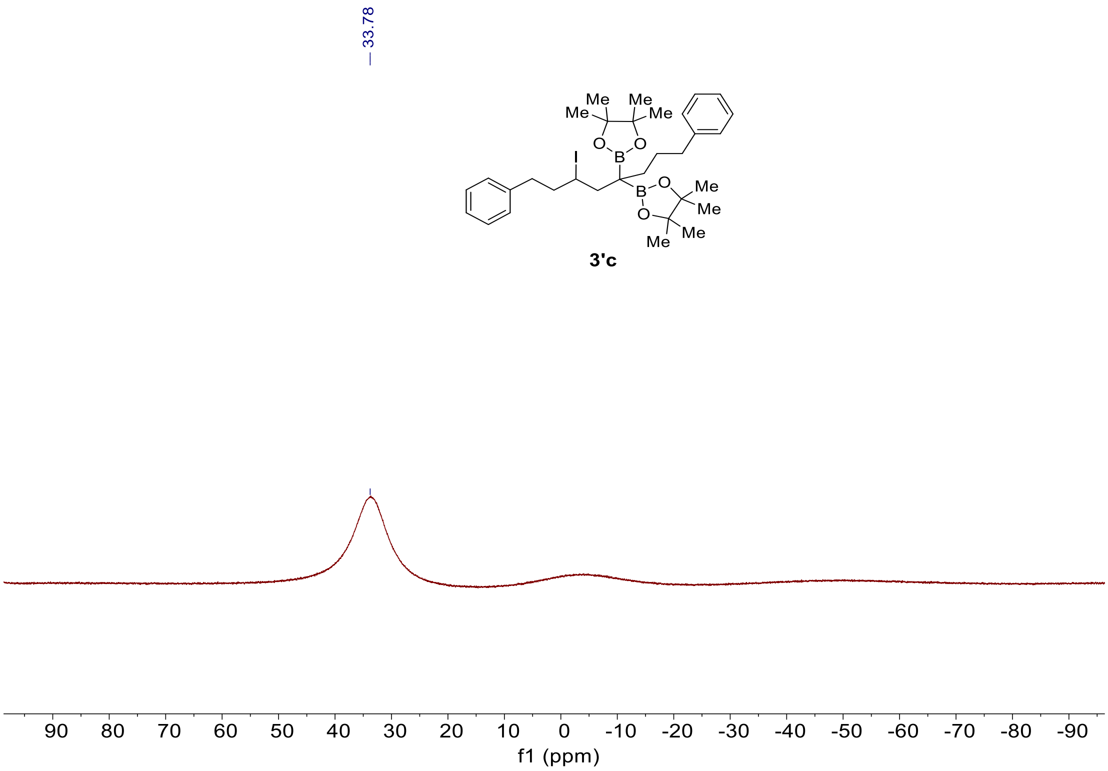

^{1}H NMR spectrum of compound **3'd** in $CDCl_3$ (400 MHz).

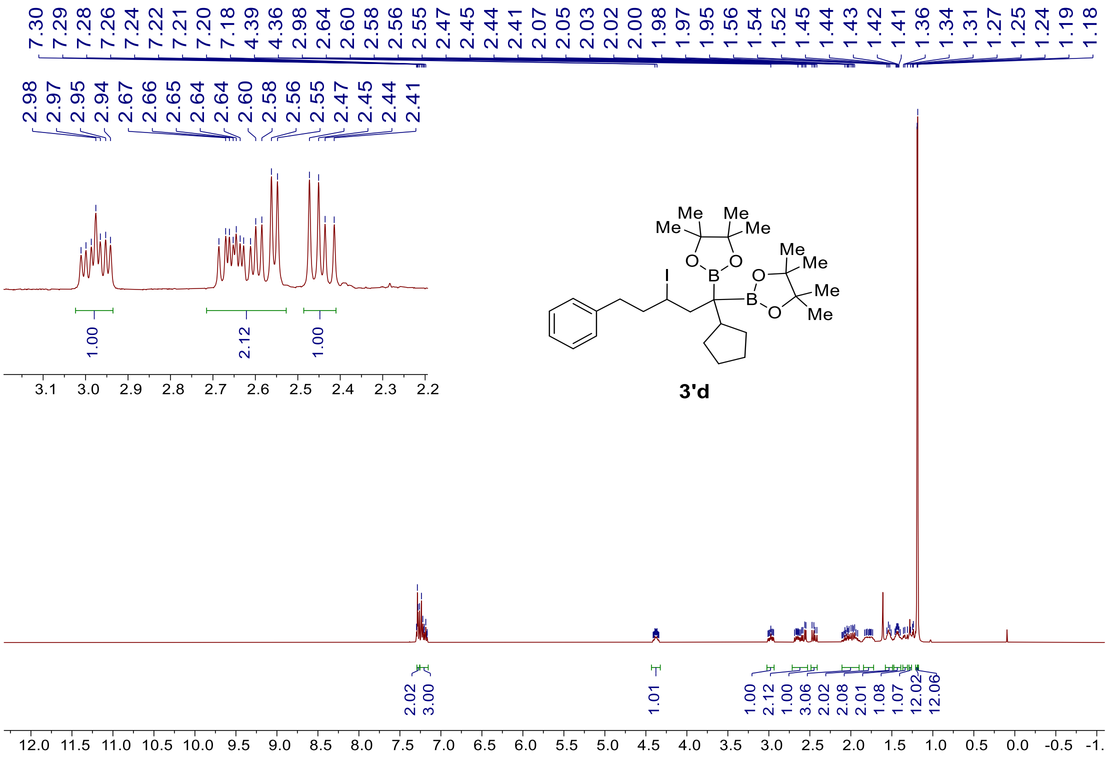

^{13}C NMR spectrum of compound **3'd** in $CDCl_3$ (101 MHz).

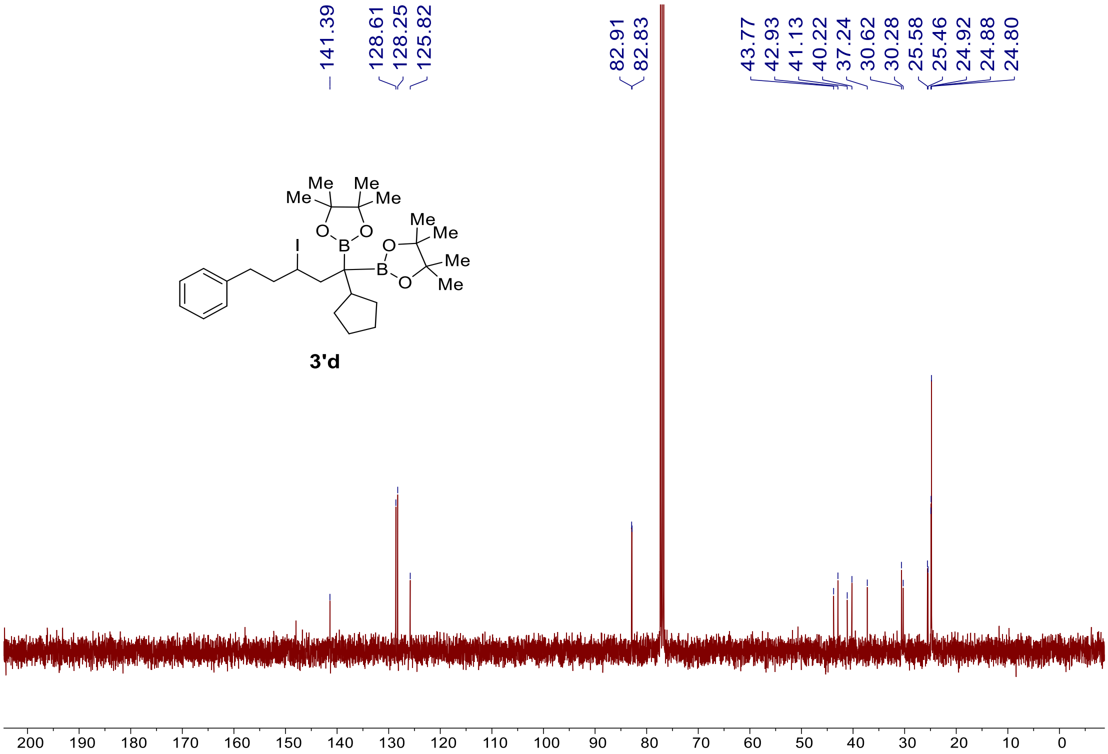

^{11}B NMR spectrum of compound **3'd** in $CDCl_3$ (128 MHz).

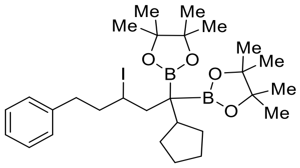

3'd

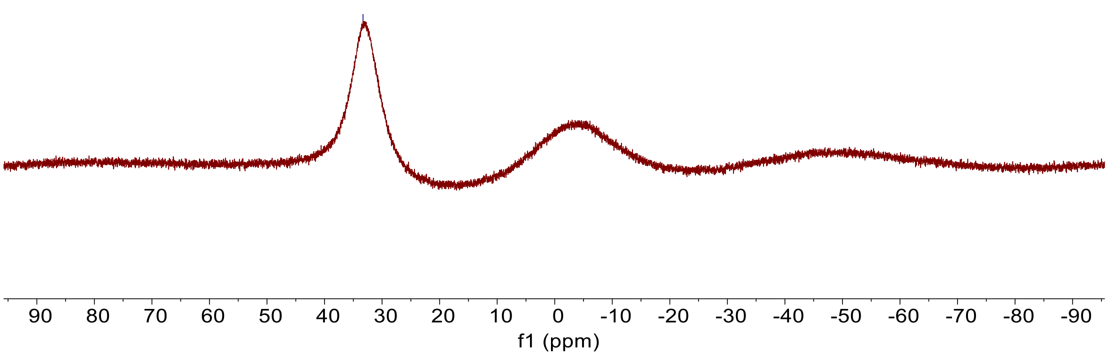

^{1}H NMR spectrum of compound **3'e** in $CDCl_3$ (400 MHz).

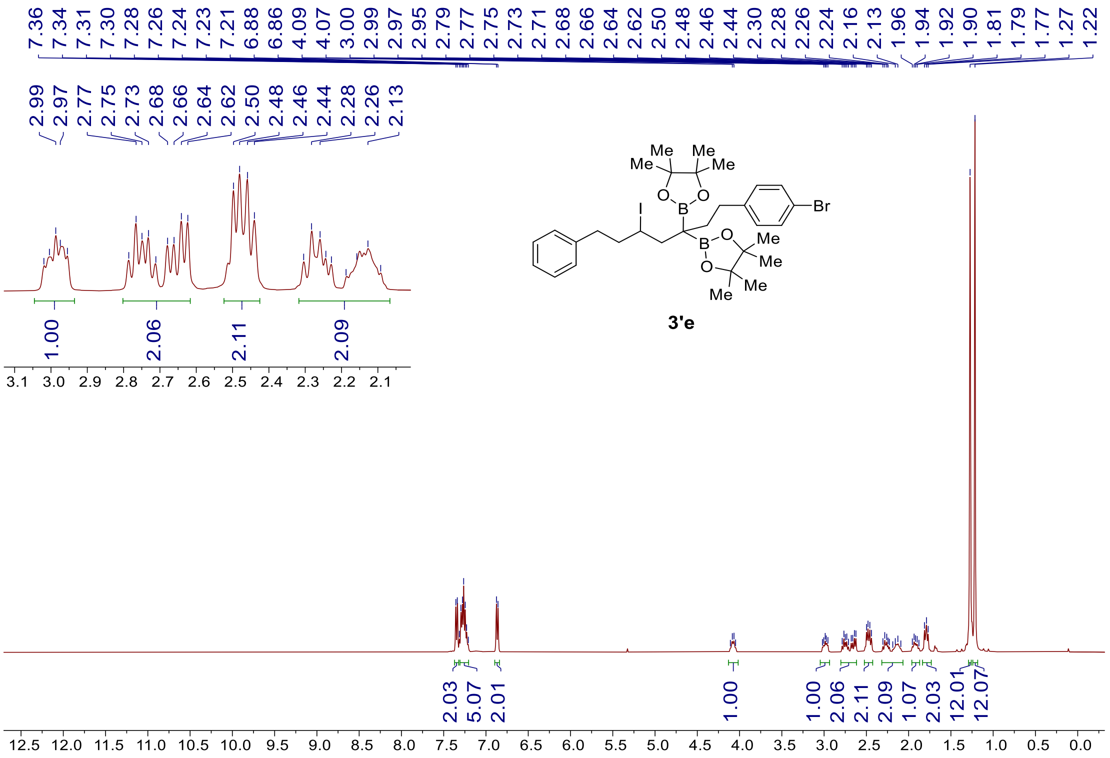

^{13}C NMR spectrum of compound **3'e** in $CDCl_3$ (101 MHz).

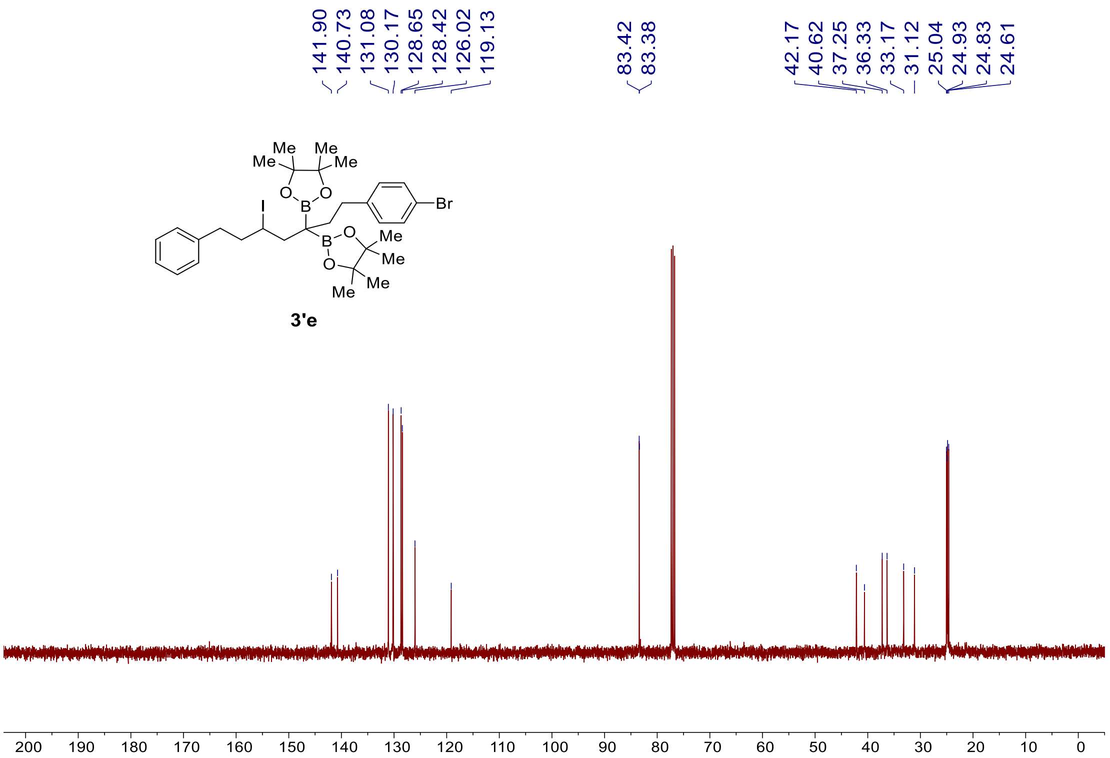

^{11}B NMR spectrum of compound **3'e** in $CDCl_3$ (128 MHz).

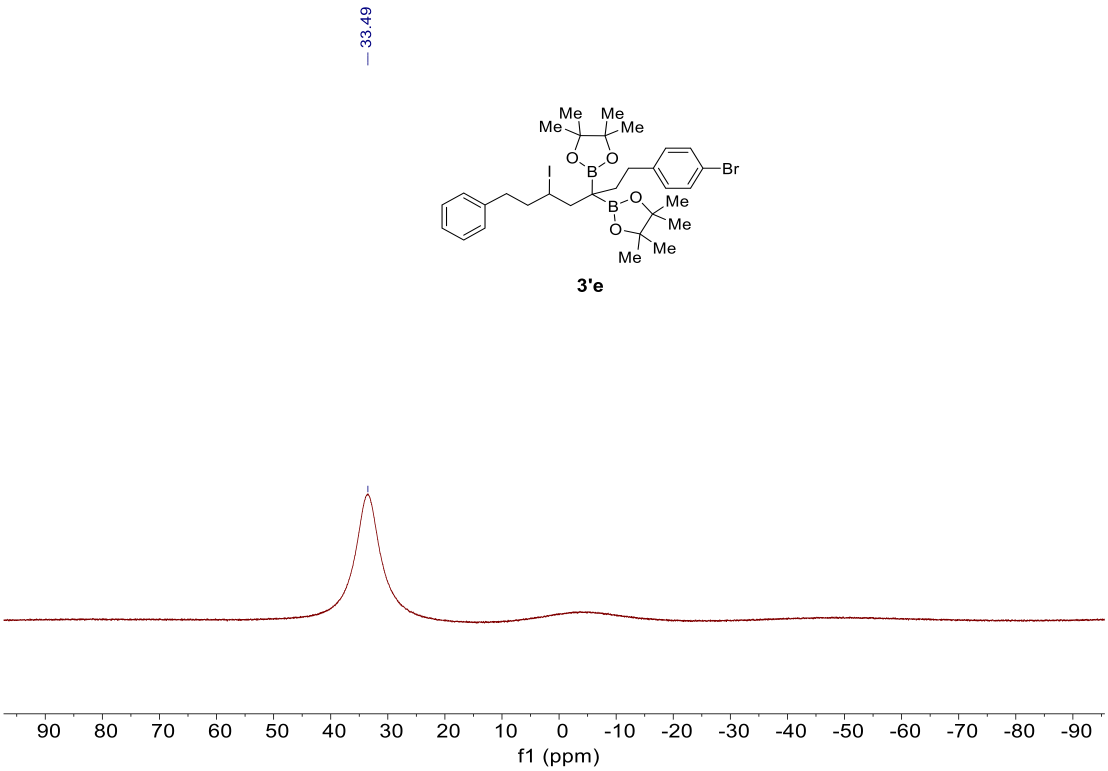

^{1}H NMR spectrum of compound **9** in $CDCl_3$ (300 MHz).

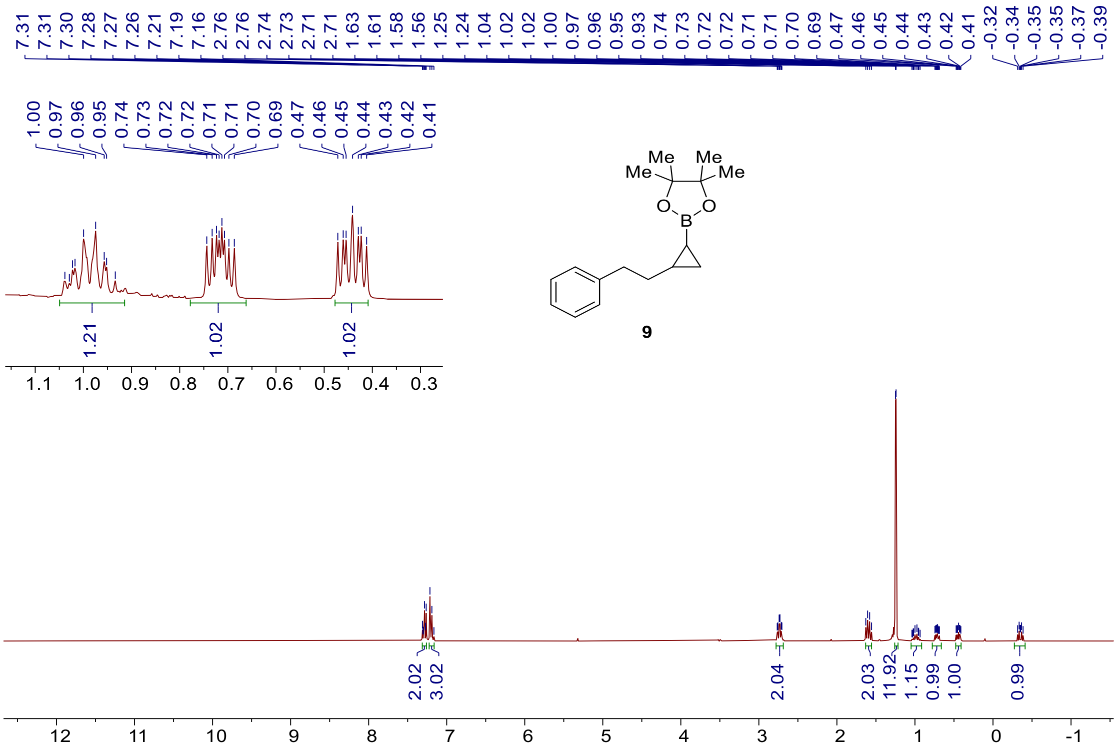

^{13}C NMR spectrum of compound **9** in $CDCl_3$ (75 MHz).

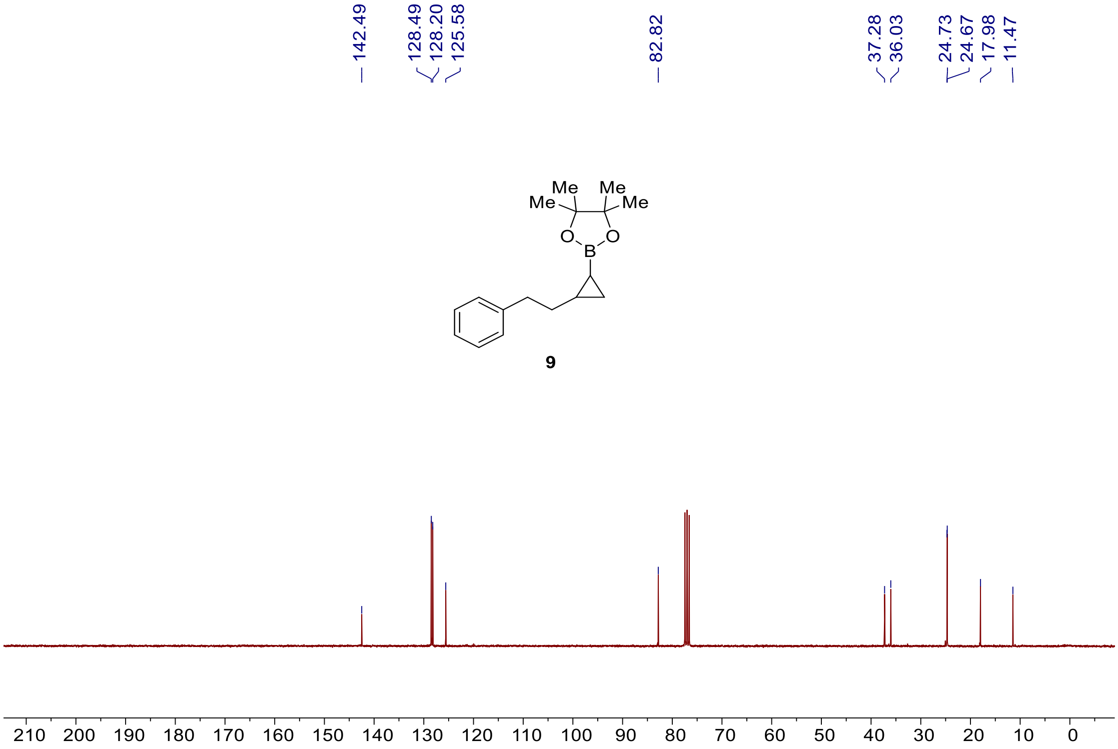

^{11}B NMR spectrum of compound **9** in $CDCl_3$ (96 MHz).

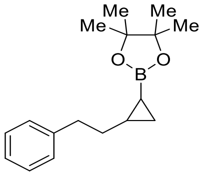

9

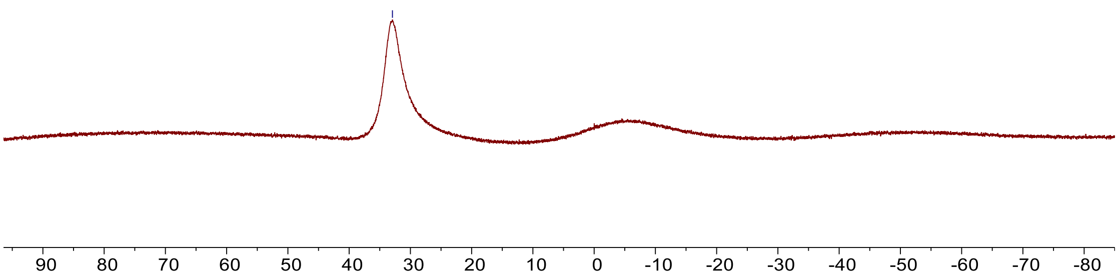

^{1}H NMR spectrum of compound **10** in $CDCl_3$ (400 MHz).

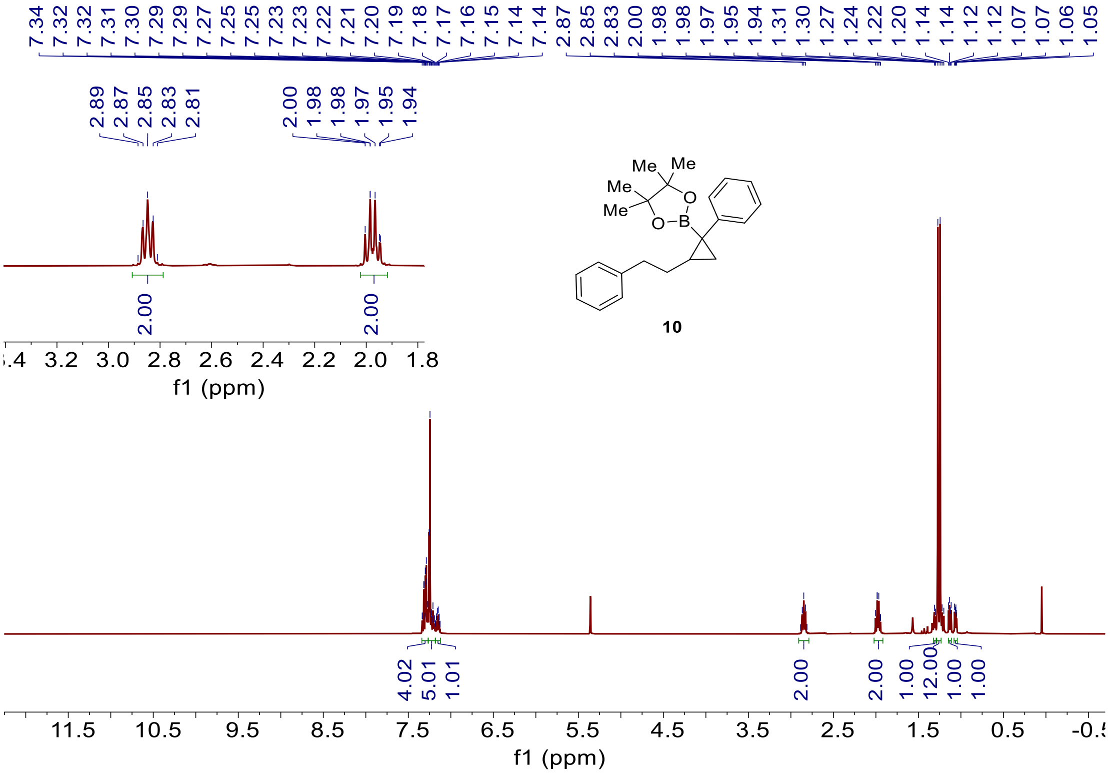

^{13}C NMR spectrum of compound **10** in $CDCl_3$ (101 MHz).

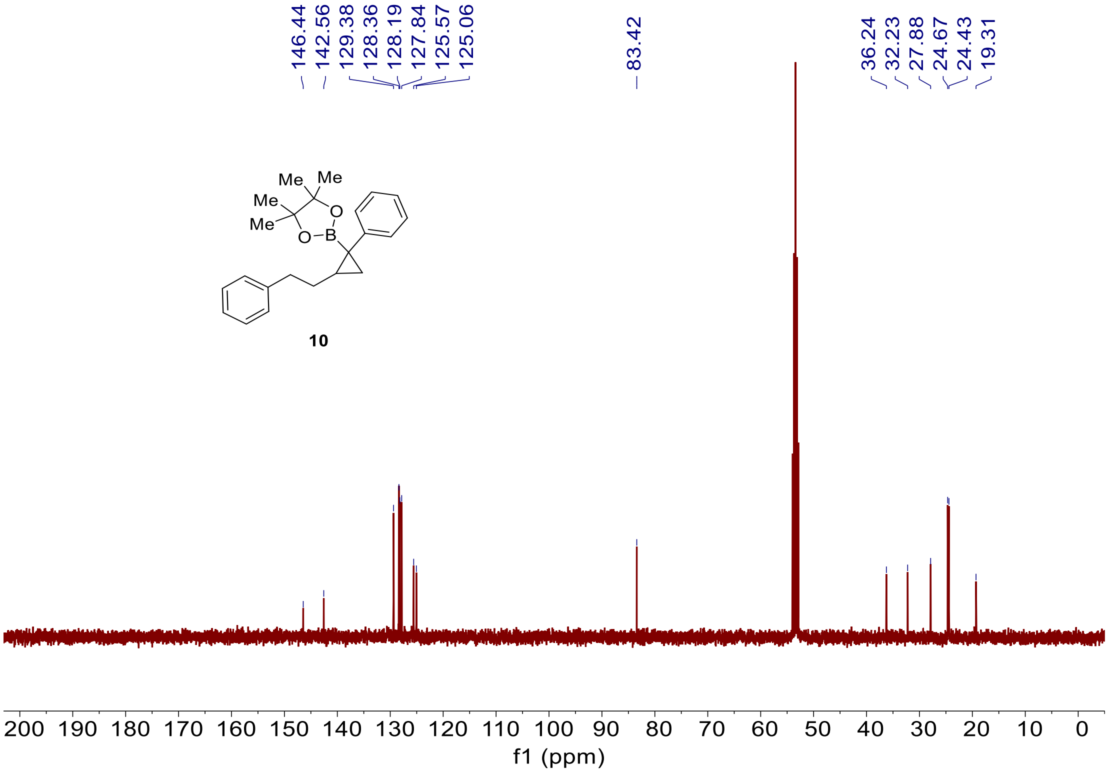

^{1}H NMR spectrum of compound **11a** in $CDCl_3$ (400 MHz).

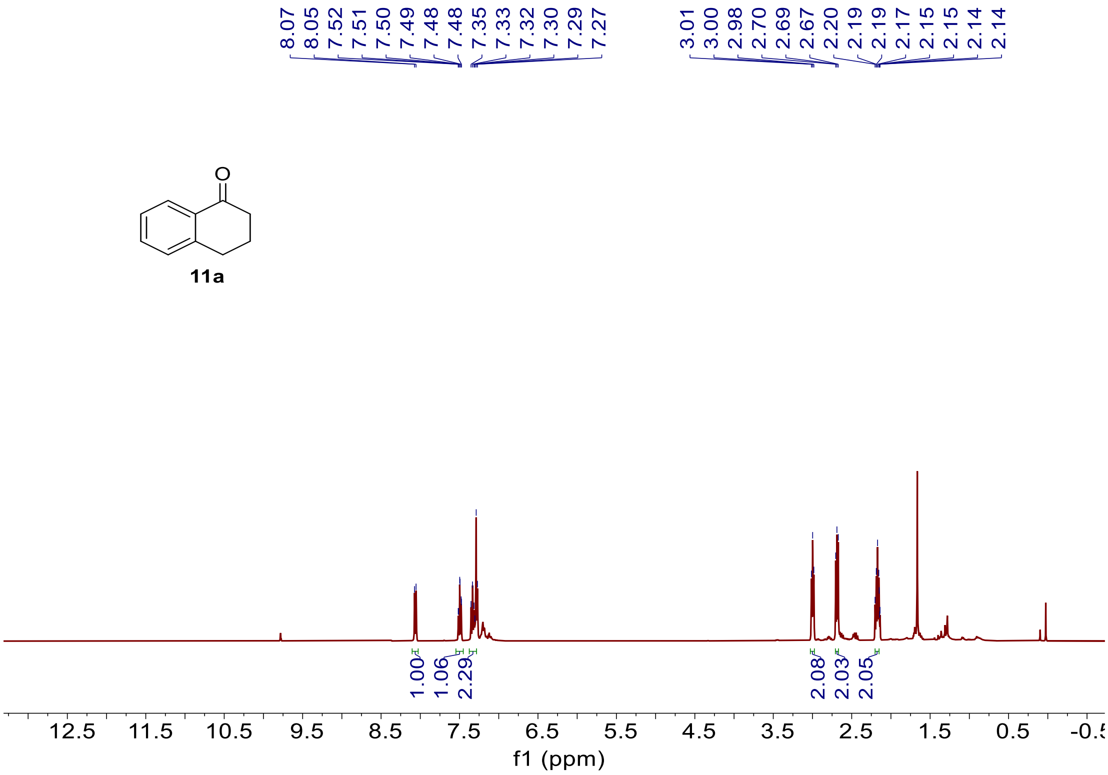

^{13}C NMR spectrum of compound **11a** in $CDCl_3$ (101 MHz).

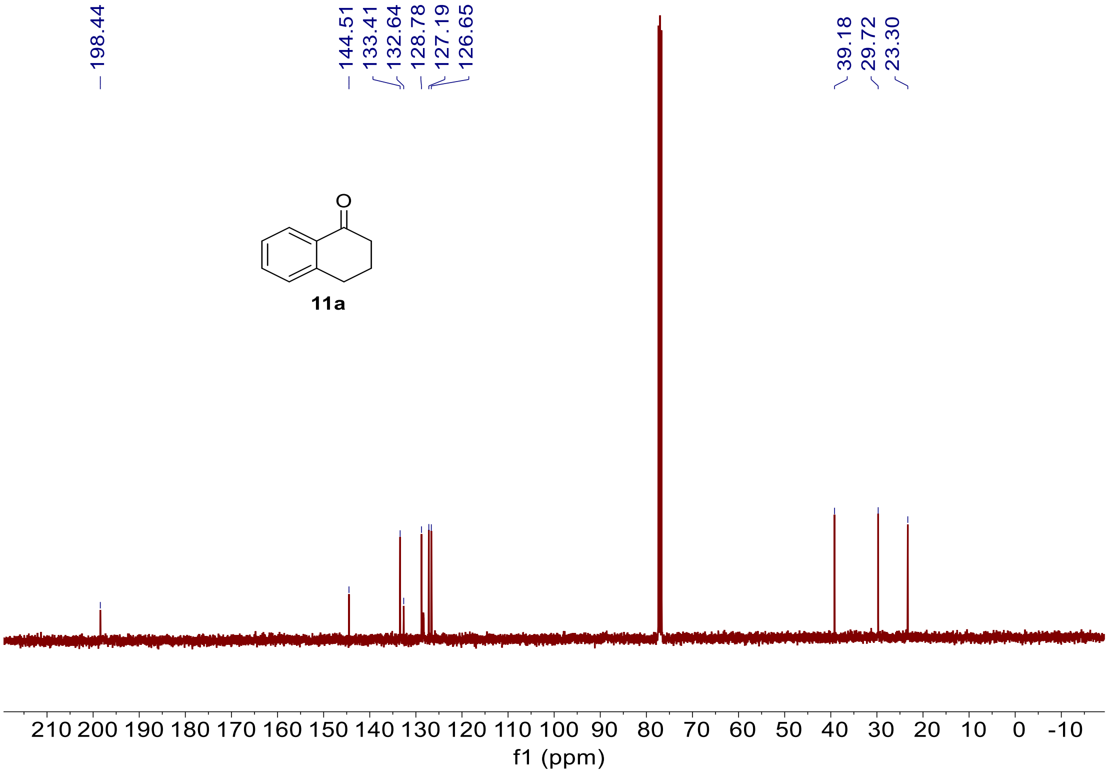

^{1}H NMR spectrum of compound **11b** in $CDCl_3$ (400 MHz).

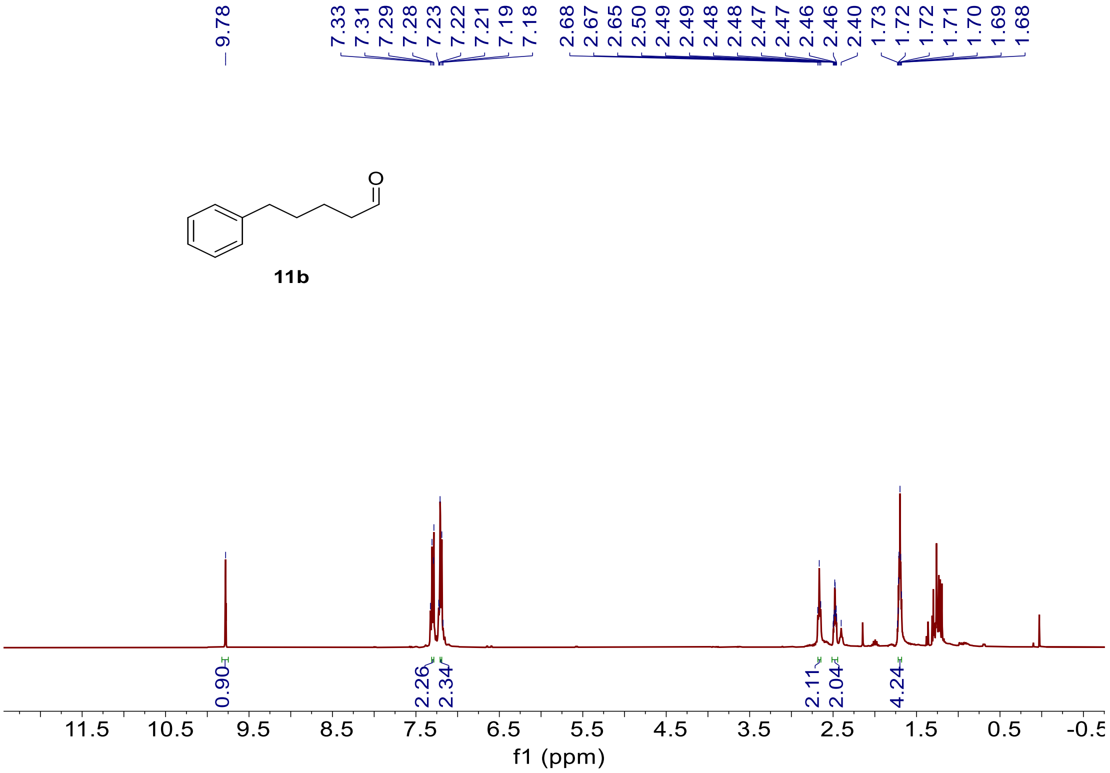

^{13}C NMR spectrum of compound **11b** in $CDCl_3$ (101 MHz).

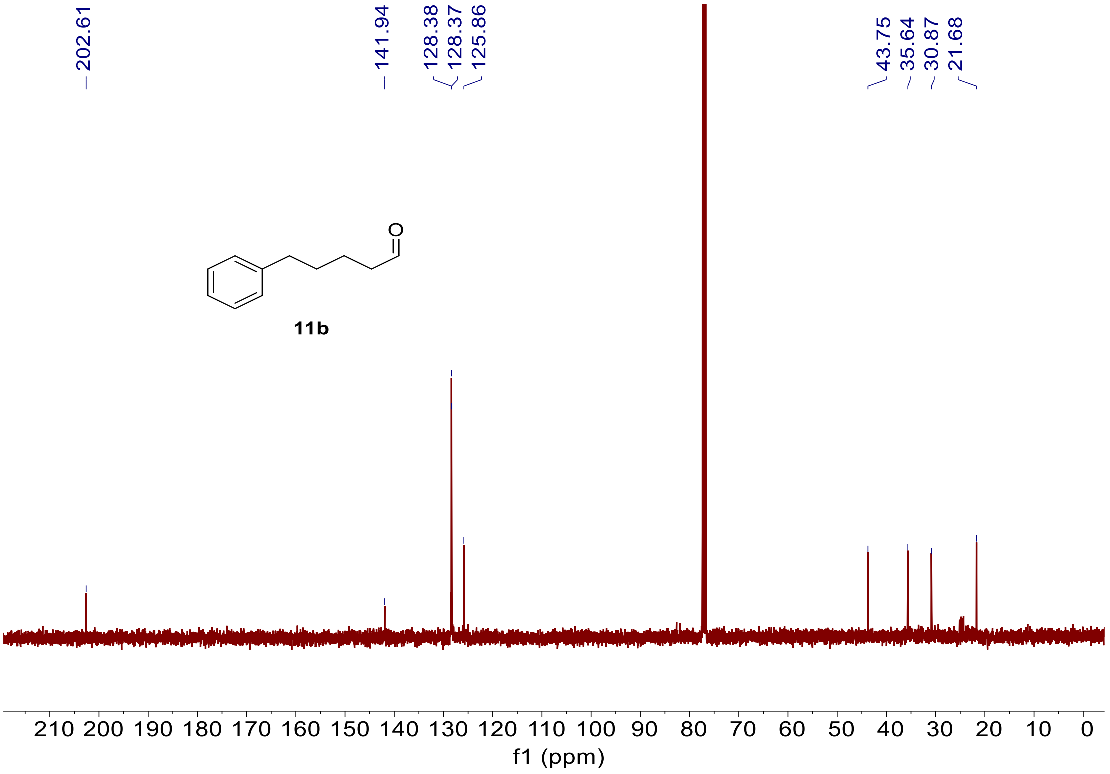

^{1}H NMR spectrum of compound **12** in $CDCl_3$ (300 MHz).

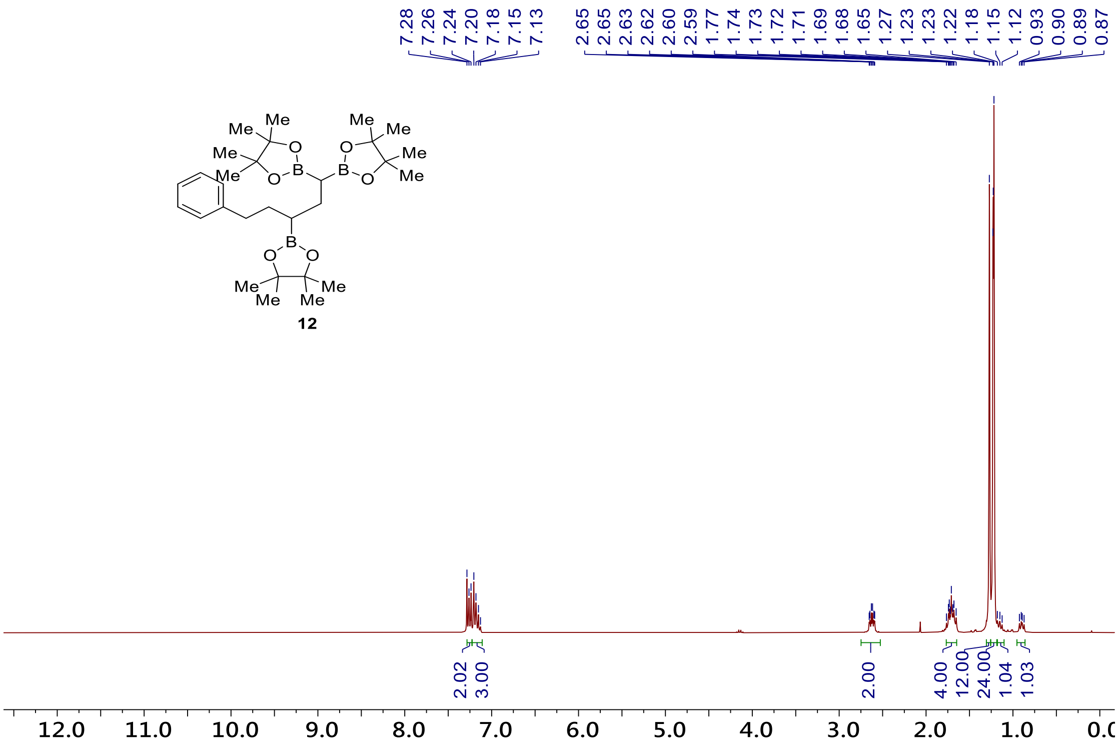

^{13}C NMR spectrum of compound **12** in $CDCl_3$ (75 MHz).

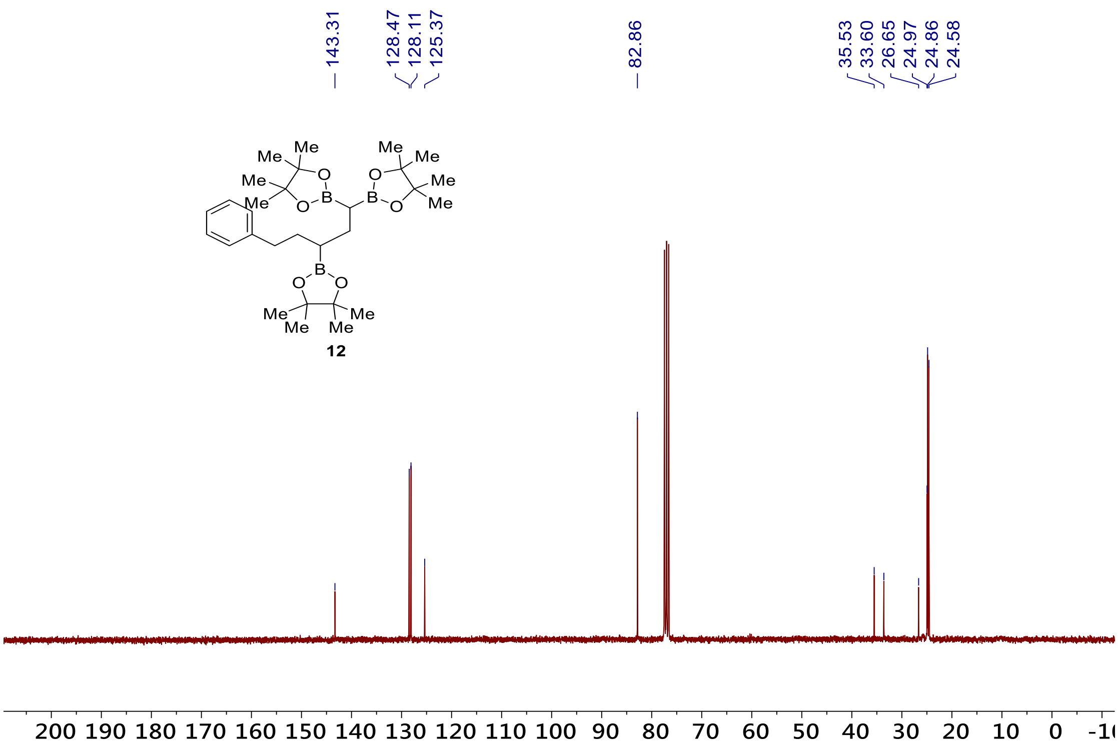

^{11}B NMR spectrum of compound **12** in $CDCl_3$ (96 MHz).

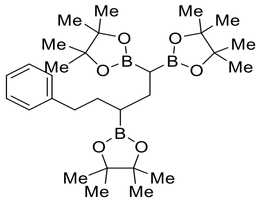

12

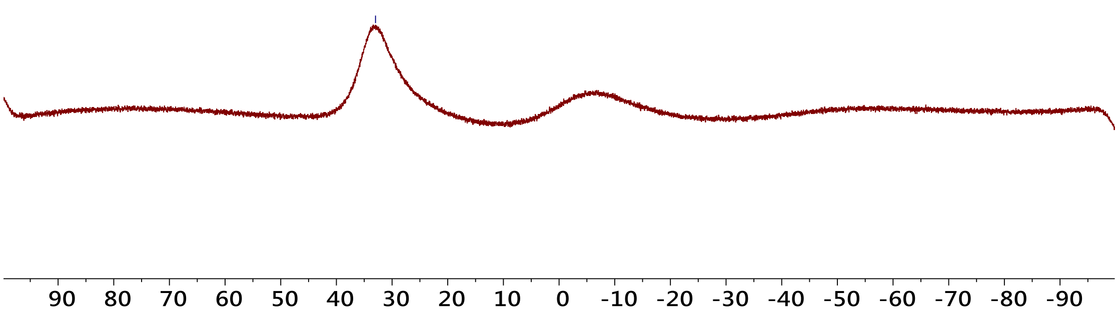

^{1}H NMR spectrum of compound **13** in $CDCl_3$ (300 MHz).

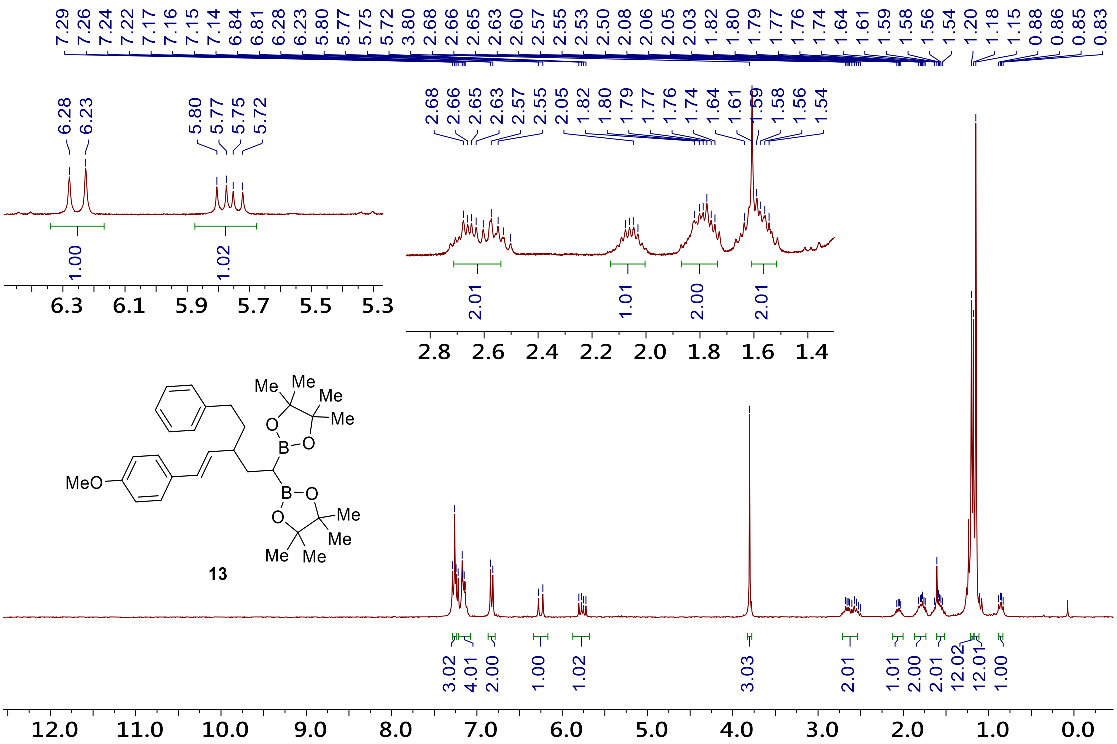

^{13}C NMR spectrum of compound **13** in $CDCl_3$ (75 MHz).

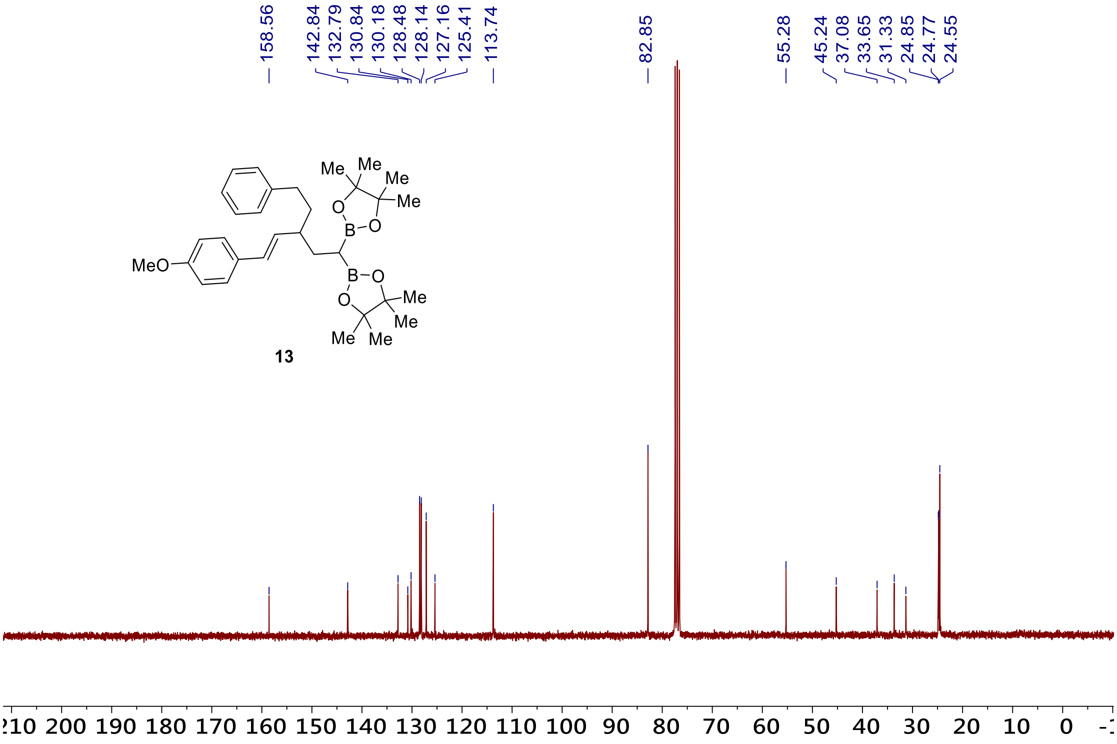

^{11}B NMR spectrum of compound **13** in $CDCl_3$ (96 MHz).

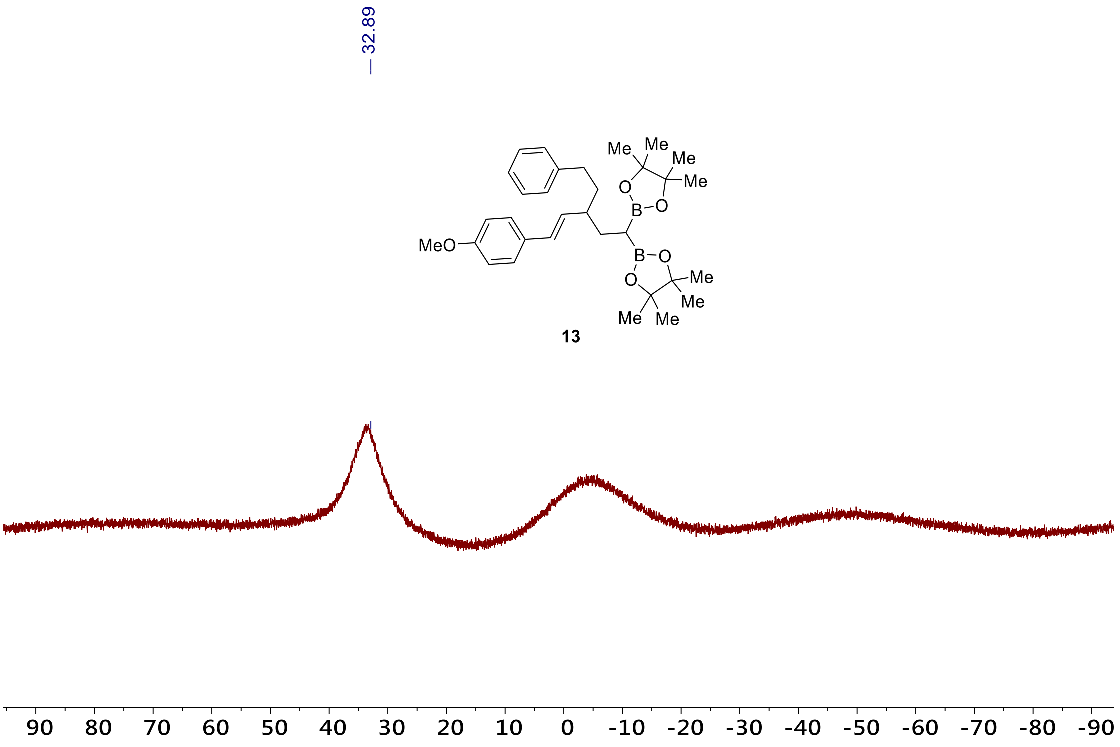

Supplement: SC-016-D5SC02670A-s001 [file SC-016-D5SC02670A-s001.pdf]
